# Hybrid Quinolinyl Phosphonates as Heterocyclic Carboxylate Isosteres: Synthesis and Biological Evaluation against Topoisomerase 1B (TOP1B)

**DOI:** 10.3390/ph14080784

**Published:** 2021-08-09

**Authors:** Asier Selas, María Fuertes, Estela Melcón-Fernández, Yolanda Pérez-Pertejo, Rosa M. Reguera, Rafael Balaña-Fouce, Birgitta R. Knudsen, Francisco Palacios, Concepcion Alonso

**Affiliations:** 1 Departamento de Química Orgánica I, Facultad de Farmacia and Centro de Investigación Lascaray, Universidad del País Vasco (UPV/EHU), Paseo de la Universidad 7, 01006 Vitoria-Gasteiz, Spain; asier.selas@ehu.eus (A.S.); maria.fuertes@ehu.eus (M.F.); francisco.palacios@ehu.eus (F.P.); 2 Departamento de Ciencias Biomédicas, Facultad de Veterinaria, Universidad de León, 24071 León, Spain; emelf@unileon.es (E.M.-F.); myperp@unileon.es (Y.P.-P.); rmregt@unileon.es (R.M.R.); 3 Department of Molecular Biology, Genetics and Interdisciplinary Nanoscience Center (iNANO), Aarhus University, 8000 Aarhus, Denmark; brk@mbg.au.dk

**Keywords:** quinolinyl phosphonates, topoisomerase 1B, enzyme inhibition, antiproliferative effect, leishmaniosis effect

## Abstract

This work describes, for the first time, the synthesis of dialkyl (2-arylquinolin-8-yl)phosphonate derivatives. The preparation was carried out through a direct and simple process as a multicomponent Povarov reaction of aminophenylphosphonates, aldehydes, and styrenes and subsequent oxidation with 2,3-dichloro-5,6-dicyano-1,4-benzoquinone (DDQ) or, alternatively, by a cycloaddition reaction between phosphonate aldimines and acetylenes. Based on phosphonate group structural characteristics, considered as phosphorous isosteres of carboxylic heterocycles, they may present interesting biological properties related to cell proliferation. In the current report, a new series of dialkyl (2-arylquinolin-8-yl)phosphonates have been synthesized and their antiproliferative effect evaluated on different human cancer and embryonic cells, as well as on *Leishmania infantum* parasites, a eukaryotic protist responsible for visceral leishmaniasis. Thereby, the antitumor effect was assessed in human lung adenocarcinoma cells (A549), human ovarian carcinoma cells (SKOV3), and human embryonic kidney cells (HEK293) versus the non-cancerous lung fibroblasts cell line (MRC5). On the other hand, the antileishmanial activity was tested against both stages of *L. infantum* cell cycle, namely free-living promastigotes and intramacrophage amastigotes, using a primary culture of Balb/c splenocytes to calculate the selectivity index. Besides the antiproliferative and antileishmanial capacities, their behavior as topoisomerase 1B inhibitors has been evaluated as a possible mechanism of action.

## 1. Introduction

Cancer is one of the most devastating diseases in spite of the substantial advances in the research of antitumor therapies. The incidence of cancer worldwide is over 18 million people and the mortality near 10 million people (in 2018) [1], and research and discovery of new effective and selective drugs/therapies remains a challenge nowadays. On the contrary, leishmaniasis is a neglected tropical disease, caused by parasitic protozoa of the *Leishmania* genus. Leishmaniasis is endemic in tropical and subtropical areas with annual case incidence ranging from 0.7 to 1 million and over 1 billion people living at risk [2]. The visceral form causes 20,000–30,000 deaths per year mostly in Sub-Saharan African countries. The current treatment of leishmaniasis is complicated and all existing drugs have significant side effects [3].

Chemotherapeutic agents that directly target DNA or modulate enzymes involved in DNA processing are of particular interest in anticancer drug research [4]. Concerning this, owing to a detrimental effect on cellular survival of DNA topoisomerase 1B (TOP1B) poisoning, this enzyme has become an interesting and broadly explored druggable target for pathologies related to proliferation phenomena, such as carcinogenesis and infection diseases produced by protist parasites. 

TOP1Bs are ATP-independent eukaryotic enzymes that relax supercoiled DNA introducing a transient break in one strand of the duplex DNA. This break allows rotation of the cut strand around the uncut strand, thus decreasing the tension in the DNA created during the replication process. Most TOP1Bs are monomeric enzymes with a catalytic tyrosine residue in the active center involved in the cleavage/binding activities. However, the TOP1B of trypanosomatids—including Leishmania—is a heterodimeric enzyme with the catalytic tyrosine located in the small subunit [5]. This structural difference has been considered an attractive target for therapeutic intervention for diseases caused by these microorganisms [6].

**Figure 1:**
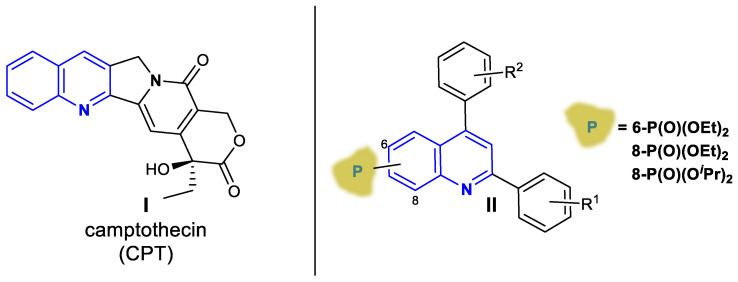
Structures of CPT **I** and hybrid quinolinyl phosphonates **II**.

Some compounds have been discovered that target this ubiquitous enzyme which modulates DNA topology in essential cellular events (transcription, replication, and chromatin assembly), as for example camptothecin (CPT, Figure 1) derivatives. However, CPT derivatives present common life-threatening side effects as hematological toxicity and diarrhea and have to deal with cancer cell resistance and structural instability as a result of lactone ring-opening in physiologic conditions. TOP1B inhibitors accepted for clinical practice are topotecan, irinotecan, and belotecan (CPT derivatives). However, these compounds still lead to toxic side effects, chemical instability, and cancer cell resistance, resulting in the limitation of their potential utility as therapeutic drugs [7].

In an attempt to avoid the aforementioned drawbacks, understanding the limitations of CPT derivatives led to the research and development of non-CPT derivatives with planar or quasi-planar nitrogen-containing fused polycyclic structures. Accordingly, several families of compounds with nitrogen-containing heterocyclic scaffolds have been successfully presented as drug candidates for TOP1B inhibitors [8]. In this sense, our group has developed the synthesis of nitrogen-containing heterocycles, such as 1,5-naphthyridine [9] derivatives, and studied their biological activity, showing good results as TOP1B inhibitors with antiproliferative [10,11] and antileishmanial [12,13] activity. In this regard, quinoline is considered a privileged scaffold in cancer drug discovery and quinoline involving central cores are of particular relevance in drugs/candidates with TOP1B inhibition-based mode of action [14,15,16,17].

Phosphorus-containing compounds play an important role in Medicinal Chemistry considering the relevance of phosphorated functionalities in their biological activity. For instance, the isosteric replacement of a carboxylic acid functionality by diarylphosphonate analogues in amino acids gives place to the corresponding α-aminodiarylphosphonates and their protein extensions (peptidyl-α-aminophosphonates), representing a highly convenient framework for improving selectivity against serine protease inhibitors [18]. Likewise, tetrahedral phosphorus is known to be an essential functionality in molecular recognition. LaVoie et al. introduced the concept of bioisosterism, when molecules modulate biological activity by virtue of subtle differences in their physicochemical properties, resulting from the substitution of some atoms in their structure [19]. In this sense, phosphorylated moieties have been employed as chemically more stable bioisosteres of phosphates in research dealing with targeting phosphorylated states of proteins, and, accordingly, phosphorylated phosphotyrosine mimetics and phosphonate nucleotides have been described to modulate biological activities of proteins that recognize phosphate groups, like kinases or phosphorylases [20]. 

Nowadays, it is also observed that the structural conjunction in molecular hybrids can give rise to potent biologically active molecules [21,22]. In previous works, our group focused on the design and preparation of phosphorus (phosphine, phosphine sulphide, and phosphine oxide) substituted quinoline derivatives as TOP1B inhibitors with antiproliferative [23] and antileishmanial [24] activity. We consider the [4 + 2] cycloaddition type Povarov reaction a highly convenient approach for the synthesis of quinoline derivatives, giving place to a wide-ranging chemical diversity arising to the large reactant scope able to get involved in the Povarov reaction [25]. In light of these considerations and following with our previous work in the synthesis of new TOP1B inhibitors, herein we report the synthesis of novel hybrid quinoline derivatives with dialkylphosphonate substituents through the Povarov reaction between aminophenylphosphonates, aromatic aldehydes, and styrenes or acetylenes, and their biological evaluation as candidates for TOP1B inhibitors with antiproliferative and antileishmanial activities.

## 2. Results and Discussion

### 2.1. Chemistry

**Scheme 1:**
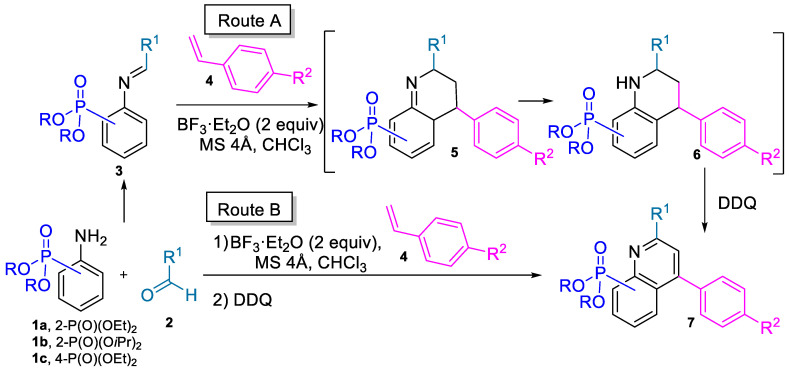
Syntheses of quinolinylphosphonate derivatives **7** from styrenes **4**.

**Chart 1:**
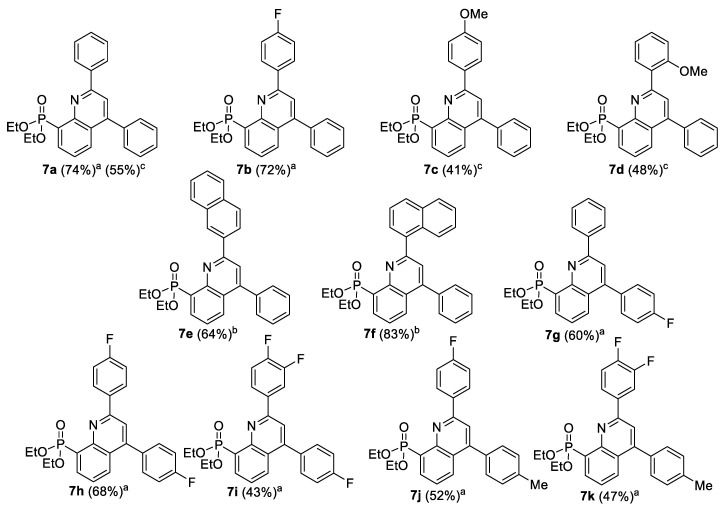
Structures of diethyl quinolin-8-ylphosphonates **7a**–**k** obtained by Povarov reaction from diethyl (2-aminophenyl)phosphonate **1a** and *^a^*by a two-step reaction with styrenes and subsequent oxidation with DDQ or *^b^*by MCR and subsequent oxidation with DDQ or *^c^*by two-step reaction with acetylenes.

The hybrid phosphonate substituted quinoline derivatives **II** (Figure 1) were synthesized by the Povarov reaction outlined in Scheme 1. First, we started with the preparation of diethyl (2-aminophenyl)phosphonate **1a** (Scheme 1, R = Et, see Supporting Material) [26]. Then, 2-(diethylphosphonate)aldimines **3** (R = Et) were prepared in situ by condensation of diethyl (2-aminophenyl)phosphonate **1a** and aromatic aldehydes **2**. These aldimines **3** were reacted with styrenes **4** by a two-step Povarov type reaction in the presence of 1 equivalent of BF_3_·Et_2_O as a Lewis acid catalyst in refluxing chloroform. For instance, when diethyl (2-(benzylideneamino)phenyl)phosphonate **3 a** (R = Et, R^1^ = H) reacted with phenyl styrene **4a** (R^2^ = H) the formation of the corresponding diethyl (2,4-diphenyl-1,2,3,4-tetrahydroquinolin-8-yl)phosphonate **6a** (R = Et, R^1^ = H, R^2^ = H) was observed in the ^1^H NMR spectrum of the crude mixture. However, attempts for the isolation of these derivatives **6** were unsuccessful and gave rise to the aromatized quinoline derivatives **7** under the purification conditions. Therefore, we explored the reaction of aldimines **3** with styrenes **4** and subsequent in situ dehydrogenation of 1,2,3,4-tetrahydroquinoline derivatives **6** with 2 equivalents of 2,3-dichloro-5,6-dicyano-1,4-benzoquinone (DDQ) in CHCl_3_ and the corresponding quinolin-8-ylphosphonates **7** were obtained in overall high yields (Scheme 1, Chart 1). The transformation of compounds **6** into compounds **7** was confirmed by ^1^H NMR spectroscopy, where signals corresponding to the four protons of tetrahydroquinoline ring of compounds **6** disappeared, while fully aromatic quinolines **7** were formed. In this sense, a wide range of compounds was obtained by using a variety of aldehydes and styrenes with both electron-withdrawing and electron-donating substituents.

The formation of compound **7** may be explained through a regio- and stereoselective [4 + 2]-cycloaddition reaction between aldimine **3a** (R = Et, R^1^ = C_6_H_5_) and phenyl styrene **4a** (R^2^ = H) followed by a prototrophic tautomerization of corresponding adduct **5** (Scheme 1, Route A) to quinoline **6** and subsequent aromatization with DDQ towards quinolines **7**.

Alternatively, the same compounds **7** can be directly obtained by a multicomponent (MCR) protocol. Thus, the three components, amines **1**, aldehydes **2**, and styrenes **4** were mixed in the presence of the Lewis acid (BF_3_·Et_2_O) in chloroform at reflux and the corresponding crude mixtures were treated with DDQ (Scheme 1, Route B).

**Scheme 2:**
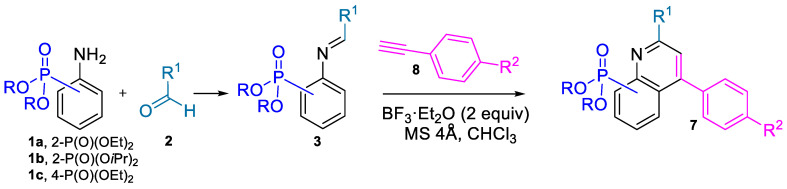
Syntheses of quinolinylphosphonate derivatives **7** from acetylenic compounds **8**.

As we previously reported, if acetylenes are used as dienophiles instead of olefins when reacting with aldimines, the corresponding aromatized compounds were directly obtained without the addition of an oxidant as DDQ [9]. For this reason, we then studied the preparation of phosphonate quinoline derivatives **7** in a direct way by means of the Povarov reaction by using aldimines **3** and acetylenes **8** in the presence of 1 equivalent of BF_3_·Et_2_O as a Lewis acid catalyst in refluxing chloroform (Scheme 2, Chart 1).

**Chart 2:**
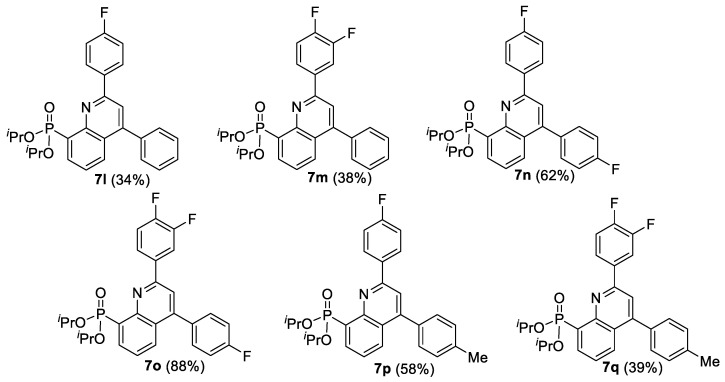
Structures of diisopropyl quinolin-8-ylphosphonates **7l**–**q** obtained by a MCR from diisopropyl (2-aminophenyl)phosphonate **1b**, aldehydes and styrenes **4** and subsequent oxidation with DDQ.

In order to increase the diversity, the preparation of isopropyl (R = *^i^*Pr) phosphonate derivatives was studied. In this case, an MCR protocol was followed by reacting diisopropyl (2-aminophenyl)phosphonate **1b** (R = *^i^*Pr) with aromatic aldehydes **2** and styrenes **4**, and after starting reagents consumption followed by the subsequent addition of DDQ and the corresponding derivatives **7l**–**q** were obtained (Chart 2).

**Chart 3:**
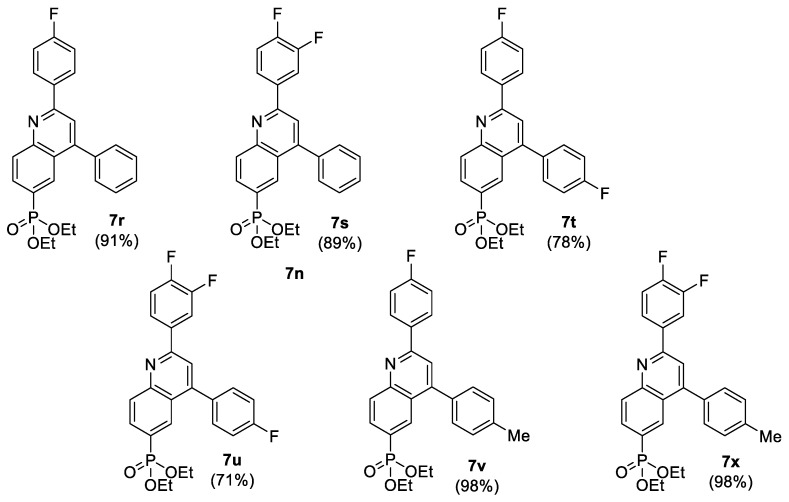
Structures of diethyl quinolin-6-ylphosphonates **7r**–**x** obtained by a two-step Povarov reaction from diethyl (4-aminophenyl)phosphonate **1c**, aldehydes **2** and styrenes **4**.

The scope of the reaction was then expanded to the preparation of quinolinyl derivatives with ethyl phosphonate group at 6-position by employing diethyl (4-aminophenyl)phosphonate **1c** (R = Et). First, aldimine **3** formation was carried out by reaction between aniline **1c** and aromatic aldehydes **2**, followed by addition of corresponding styrene **4** and subsequent oxidation with DDQ to give the corresponding derivatives **7r**–**x** (R = Et, Chart 3).

By the described methodology hybrid diethyl and di-isopropyl quinolines are obtained directly from appropriately functionalized anilines. In the next steps, the biological activity of these novel compounds as antiproliferative agents, TOP1B inhibitors, and antileishmanial agents was studied.

### 2.2. In Vitro Cytotoxicity

The cytotoxicity of the newly synthesized quinolinylphosphonates **7** was evaluated in vitro by testing their antiproliferative activities against several human cancer cell lines: A549 (human lung adenocarcinoma cells), SKOV3 (human ovarian carcinoma), and HEK293 (human embryonic kidney). In order to analyze the selective cytotoxicity facing exclusively cancerous cell lines, cytotoxicity against non-cancerous cell lines (MRC-5, lung fibroblast cells) was also evaluated. Cell counting kit (CCK-8) assay was employed to assess growth inhibition and, cell proliferation inhibitory activities of compounds that are listed in Table 1 as IC_50_ values. The tested compounds displayed a broad spectrum of antiproliferative activity against the cancer cell lines tested in culture. According to the data presented in Table 1, in general, all these phosphorated derivatives present higher selective cytotoxicity in the A549 cell line than in the other two cell lines, SKOV3 and HEK293, although slight differences are observed compared to human ovarian carcinoma (SKOV3). Moreover, MRC-5 non-malignant lung fibroblasts were tested for studying selective toxicity and none of the synthesized phosphorated compounds exhibited any toxicity toward MRC-5 cells (Table 1). 

In general ethyl phosphonates **7a**–**k** and **7r**–**x** (R = Et, entries 2–12 and 19–24) present higher cytotoxicity than isopropyl phosphonates **7l**–**q** (R = *^i^*Pr, entries 13–18). In addition, amongst the diethyl phosphonate derivatives, the derivatives with this group in position 6 show the best biological response with cytotoxic values under 7 μM (Table 1, entries 19–24). Specifically, the most effective compounds with values in the nanomolar range were the derivative **7j** (R = Et (phosphonate group at position 8), R^1^ = 4-F, R^2^ = Me, entry 11) with an IC_50_ value of 830 ± 10 nM and the derivative **7v** (R = Et (phosphonate group at position 6), R^1^ = 4-F, R^2^ = Me, entry 23) with an IC_50_ value of 840 ± 230 nM.

### 2.3. Inhibition of Human TOP1B (hTOP1B)

**Figure 2:**
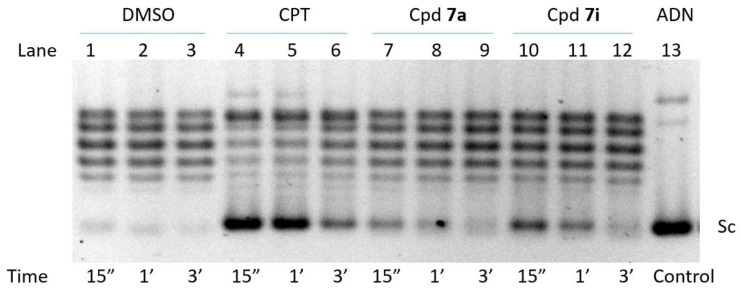
Inhibition of hTOP1B activity along the time (15′′, 1′ and 3′) by compounds **7a**, **7i** and CPT at 100 μM: lanes 1–3, DNA + hTOP1B + DMSO; lanes 4–6, DNA + hTOP1B + CPT; lanes 7–9, DNA + hTOP1B + **7a**; lanes 10–12, DNA + hTOP1B + **7i**; lane 13, control DNA. Reaction samples were mixed with the supercoiled DNA substrate before adding enzyme at 37 °C and separated by electrophoresis on a 1% agarose gel, and then stained with gel red, and photographed under UV light as described in the hTOP1B mediated DNA relaxation assay. Sc, supercoiled DNA.

The activity as topoisomerase 1B inhibitors of newly prepared quinolinylphosphonates **7** was evaluated through the assessment of conventional supercoiled DNA relaxation assay, determining their inhibitory effect on hTOP1B by quantifying the transformation of a supercoiled DNA plasmid into the relaxed form in the presence of the compounds (Table 2). In these experiments, compound samples were mixed with purified enzyme followed by the addition of supercoiled plasmid DNA and continued incubation for increasing time periods (15 s, 1 min, and 3 min). The reaction was terminated by the addition of SDS. DNA relaxation products were then resolved by electrophoresis in 1% agarose gel and visualized by gel red staining. CPT was used as a positive control (Figure 2).

The effects of CPT and DMSO (used as a solvent for the dissolutions of CPT and the new compounds) were tested as a positive and negative control, respectively, in the experiment. As expected, CPT inhibited the relaxation, as a higher amount of signal corresponding to supercoiled (unrelaxed) plasmid was observed at short reaction times (15 s and 1 min) when 100 μM CPT was added than when the enzymatic reaction was performed with the DMSO solvent alone (compare lanes 4 and 5 with lanes 1 and 2 in Figure 2). Only after a longer incubation time, more relaxation was observed in the presence of CPT (lane 6). 

By performing these experiments, the inhibitory ability of hTOP1B activity of the new compounds **7** was tested. In Figure 2 it can be seen that for example compound **7a** showed slight inhibition of hTOP1B activity at any enzymatic reaction time (lanes 7–9). Also in this Figure 2, the inhibition of hTOP1B activity of compound **7i** is evidenced by the appearance of supercoiled DNA signals (lanes 10 and 11). As a result, the behavior of all the new compounds **7** has been studied and the results have been summarized in Table 2. Some compounds, such as **7b**, **7j**, **7k**, and **7n** show inhibition at 15 s and 1 min of an enzymatic reaction, similar to that of the natural inhibitor CPT (Table 2, entries 3, 11, 12, and 15).

### 2.4. Docking Study

**Figure 3:**
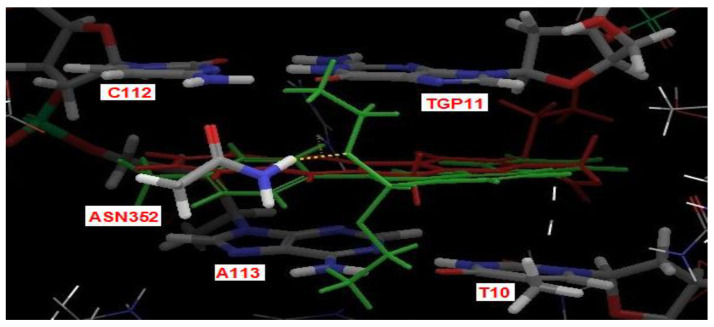
The 3D diagram representation of compound **7b** (in green), superposed with CPT (in red) docked into hTOP1B active site showing its binding interactions with the amino acids binding site.

Among all the compounds synthesized in this work, a molecular docking study of compound **7b** who showed significant inhibition of hTOP1B was carried out to investigate its possible binding pattern and its interaction with the key amino acids and DNA nucleobases in the active site of the hTOP1B. The model was derived by the docking of compound **7b** in the CPT binding site of the CPT-hTOP1B-DNA ternary complex (PDB ID: 1T8I) [27]. The most important evaluation criterion was the observation of whether the ligand was located between C112-TGP11 and A113-T10 nucleobases (Figure 3), where the DNA rupture site is located, avoiding the re-ligation of such bases, according to the concept of interfacial inhibition proposed by Pommier [7,28]. The formation of hydrogen bonds with important residues [29,30,31,32] and the existence of hydrophobic interactions with hTOP1B residues and DNA were also taken into account.

### 2.5. Antileishmanial Activity of New Quinolinylphosphonate Derivatives

The effect of the new quinolinylphosphonate derivatives **7** synthesized was assessed on both free-living promastigotes and intramacrophage amastigotes of *L. infantum* as it is described in experimental protocols. Primary cultures of intramacrophage amastigotes were obtained from splenic explants from 6-weeks infected Balb/c mice with *L. infantum-*iRFP promastigotes. The infrared fluorescence emitted by both stage forms of the parasite was used as viability criterium, using 0.5% DMSO as negative control (100% viability) and 50 mM AMB as a positive control (0% viability).

All the compounds were active against both parasite forms (Table 3). Thus, the EC_50_ values for promastigotes ranged from 0.91 ± 0.04 µM corresponding to compound **7a** (Table 3, entry 1) to 21.29 ± 2.34 µM related to compound **7x** (Table 3, entry 23). On the other hand, the EC_50_ values for amastigotes fluctuate between 4.03 ± 0.30 µM for **7a** and 32.95 ± 6.55 for **7x** (Table 3, entry 1 and 23). The selectivity of all the compounds was low, which indicates high toxicity, this parameter was calculated as the ratio between the EC_50_ obtained for amastigotes and the IC_50_ values for murine splenic cells. The higher selectivity index was obtained for **7u** compound (>3.8) (Table 3, entry 21).

### 2.6. Inhibition of Leishmanial TOP1 (LTOP1B)

The inhibition of relaxation activity of LTOP1B by the compounds was tested at 100 µM. The reactions were performed for 1 min in the way described in the experimental protocols section. The percentage of inhibition was calculated by measuring the intensity of the band corresponding to the supercoiled DNA when the different DNA topoisomers were separated in an agarose gel. At the concentration tested, none of the compounds inhibited completely the LTOP1B enzyme, being **7k** and **7o** derivatives the best inhibitors, with percentages of inhibition of 77.02 and 73.27, respectively (Table 3, entries 11 and 15).

## 3. Materials and Methods

### 3.1. Chemistry

#### 3.1.1. General Methods

All reagents from commercial suppliers were used without further purification. All solvents were freshly distilled before use from appropriate drying agents. All other reagents were recrystallized or distilled when necessary. Reactions were performed under a dry nitrogen atmosphere. Analytical TLCs were performed with silica gel 60 F_254_ plates. Visualization was accomplished by UV light. Column chromatography was carried out using silica gel 60 (230–400 mesh ASTM). Melting points were determined with an Electrothermal IA9100 Digital Melting Point Apparatus without correction. NMR spectra were obtained on a Bruker Avance 400 MHz and a Varian VXR 300 MHz spectrometer and recorded at 25 °C. Chemical shifts for ^1^H NMR spectra are reported in ppm downfield from TMS, chemical shifts for ^13^C NMR spectra are recorded in ppm relative to internal deuterated chloroform (*δ* = 77.2 ppm for ^13^C), chemical shifts for ^19^F NMR are reported in ppm downfield from fluorotrichloromethane (CFCl_3_). Coupling constants (*J*) are reported in Hertz. The terms m, s, d, t, q refer to multiplet, singlet, doublet, triplet, quartet. ^13^C NMR and ^19^F NMR were broadband decoupled from hydrogen nuclei. High-resolution mass spectra (HRMS) were measured by the EI method with an Agilent LC-Q-TOF-MS 6520 spectrometer.

#### 3.1.2. Compounds Purity Analysis

All synthesized compounds were analyzed by HPLC to determine their purity. The analyses were performed on Agilent 1260 Infinity HPLC system (C-18 column, Hypersil, BDS, 5 μm, 0.4 mm × 25 mm). All the tested compounds were dissolved in dichloromethane, and 1μL of the sample was loaded onto the column. Ethanol and heptane were used as mobile phase, and the flow rate was set at 1.0 mL/min. The maximal absorbance at the range of 190−625 nm was used as the detection wavelength. The purity of all the tested compounds is >95%, which meets the purity requirement by the Journal.

#### 3.1.3. Synthesis of Aminophenyl Phosphonates **1**

General procedure. To a suspension of 1 equiv. (75 mmol, 12.608 g) of 1,2-dinitrobenzene in 40 mL of dry MeCN, was added to the appropriate phosphite derivative (1.2 equiv) and stirred at MeCN reflux for 12 h. The crude obtained was purified by silica column chromatography (10% of ethyl acetate in hexane) and subsequently recrystallized in ethyl acetate at 4 °C, to afford the corresponding nitro derivative as a yellow solid. Subsequently, this derivative was hydrogenated with a spoon of RANEY^®^ Nickel (approx. 5 g) in 60 mL of MeOH at 80 psi at room temperature for 12 h. The reaction mixture was then filtered off through a pad of cellite and evaporated to dryness to obtain the corresponding aminophenyl phosphonate derivative 1.

*Diethyl (2-aminophenyl)phosphonate* (**1a**). General procedure was used with triethyl phosphite (90 mmol, 14.96 g, 15.44 mL). The crude obtained was purified by column chromatography to afford 15.28 g (59 mmol) of diethyl (2-nitrophenyl)phosphonate as a yellow solid (79%) mp 58–60 °C (ethyl acetate/hexane). ^1^H NMR (300 MHz, CDCl_3_) *δ*: 1.28–1.32 (m, 6 H, 2 CH_3_), 4.11–4.23 (m, 4 H, 2 CH_2_), 7.63–7.66 (m, 2 H), 7.79–7.83 (m, 1 H), 8.00–8.06 (m, 1 H) ppm; ^13^C {^1^H} NMR (75 MHz, CDCl_3_) *δ*: 16.2 (d, ^3^*J*_CP_ = 6.5 Hz, 2 CH_3_), 63.3 (d, ^2^*J*_CP_ = 6.1 Hz, 2 CH_2_), 123.3 (d, ^1^*J*_CP_ = 190.3 Hz, C), 124.1 (d, ^3^*J*_CP_ = 8.6 Hz, HC), 132.2 (d, ^3^*J*_CP_ = 13.0 Hz, HC), 133.2 (d, ^4^*J*_CP_ = 2.5 Hz, HC), 135.0 (d, ^2^*J*_CP_ = 5.9 Hz, HC), 152.0 (d, ^4^*J*_CP_ = 2.5 Hz, C) ppm; ^31^P {^1^H} NMR (120 MHz, CDCl_3_) *δ*: 12.7 ppm.

The hydrogenation of this derivative produced 13.39 g (58 mmol) of diethyl (2-aminophenyl)phosphonate **1a** (99%) as a brown oil, Rf= 0.42 (50:50 EtOAc/hexane). ^1^H NMR (300 MHz, CDCl_3_) *δ*: 1.23–1.38 (m, 6 H, 2 CH_3_), 4.00–4.17 (m, 4 H, 2 CH_2_), 5.14 (sa, NH_2_), 6.62–6.72 (m, 2 H), 7.23–7.29 (m, 1 H), 7.40–7.48 (m, 1 H) ppm; ^13^C {^1^H} NMR (75 MHz, CDCl_3_) *δ*: 16.5 (d, ^3^*J*_CP_ = 6.6 Hz, 2 CH_3_), 62.2 (d, ^2^*J*_CP_ = 4.9 Hz, 2 CH_2_), 108.3 (d, ^1^*J*_CP_ = 183.5 Hz, C), 116.4 (d, ^3^*J*_CP_ = 12.7 Hz, HC), 117.1 (d, ^3^*J*_CP_ = 13.9 Hz, HC), 133.4 (d, ^2^*J*_CP_ = 7.3 Hz, HC), 134.0 (d, ^4^*J*_CP_ = 2.1 Hz, HC), 151.4 (d, ^2^*J*_CP_ = 8.4 Hz, C) ppm; ^31^P {^1^H} NMR (120 MHz, CDCl_3_) *δ*: 22.3 ppm.

*Diisopropyl (2-aminophenyl)phosphonate* (**1b**). General procedure was used with triisopropyl phosphite (90 mmol, 18.74 g, 22.21 mL). The crude obtained was purified by column chromatography to afford 17.88 g (62.25 mmol) of diisopropyl (2-nitrophenyl)phosphonate as a brown oil (83%), Rf= 0.39 (50:50 EtOAc/hexane). ^1^H NMR (300 MHz, CDCl_3_) *δ*: 1.28 (d, ^3^*J*_HH_ = 6.2 Hz, 2 CH_3_), 1.39 (d, ^3^*J*_HH_ = 6.2 Hz, 2 CH_3_), 4.77–4.85 (m, 2 H, 2 CH), 7.65–7.67 (m, 2 H), 7.79–7.83 (m, 1 H), 8.10–8.16 (m, 1 H) ppm; ^13^C {^1^H} NMR (75 MHz, CDCl_3_) *δ*: 23.8 (d, ^3^*J*_CP_ = 4.9 Hz, 2 CH_3_), 24.1 (d, ^3^*J*_CP_ = 4.4 Hz, 2 CH_3_), 72.4 (d, ^2^*J*_CP_ = 6.2 Hz, 2 CH), 124.2 (d, ^3^*J*_CP_ = 8.6 Hz, HC), 124.6 (d, ^1^*J*_CP_ = 189.9 Hz, C), 132.1 (d, ^3^*J*_CP_ = 13.1 Hz, HC), 133.1 (d, ^4^*J*_CP_ = 2.1 Hz, HC), 135.3 (d, ^2^*J*_CP_ = 6.2 Hz, HC), 152.3 (C) ppm; ^31^P {^1^H} NMR (120 MHz, CDCl_3_) *δ*: 10.2 ppm.

The hydrogenation of this derivative produced 15.84 g (61.62 mmol) of diisopropyl (2-aminophenyl)phosphonate **1c** as a white solid (98%) mp 122–124 °C (ethyl acetate/hexane). ^1^H NMR (300 MHz, CDCl_3_) *δ*: 1.20 (d, ^3^*J*_HH_ = 6.2 Hz, 2 CH_3_), 1.34 (d, ^3^*J*_HH_ = 6.2 Hz, 2 CH_3_), 4.56–4.68 (m, 2 H, 2 CH), 5.16 (sa, 2 H, NH_2_), 6.58–6.68 (m, 2 H), 7.18–7.26 (m, 1 H), 7.39–7.47 (m, 1 H) ppm; ^13^C {^1^H} NMR (75 MHz, CDCl_3_) *δ*: 23.9 (d, ^3^*J*_CP_ = 5.0 Hz, 2 CH_3_), 24.3 (d, ^3^*J*_CP_ = 3.8 Hz, 2 CH_3_), 70.8 (d, ^2^*J*_CP_ = 5.1 Hz, 2 CH), 109.8 (d, ^1^*J*_CP_ = 184.3 Hz, C), 116.3 (d, ^3^*J*_CP_ = 10.6 Hz, HC), 116.9 (d, ^3^*J*_CP_ = 14.0 Hz, HC), 133.6 (d, ^2^*J*_CP_ = 7.2 Hz, HC), 133.7 (d, ^2^*J*_CP_ = 2.2 Hz, HC), 151.1 (d, ^4^*J*_CP_ = 8.6 Hz, C) ppm; ^31^P {^1^H} NMR (120 MHz, CDCl_3_) *δ*: 20.1 ppm.

*Diethyl (4-aminophenyl)phosphonate* (**1c**). To a suspension of 1 equiv. (25 mmol, 5.050 g) of 1-bromo-4-nitrobenzene with 6% mol of triphenylphosphine, 2% mol of palladium(**II**) acetate and 1.5 equiv. of freshly distilled triethylamine (37.50 mmol, 3.80 g, 5.23 mL) in 50 mL of deoxygenated dry EtOH, 1.2 equiv. (30 mmol, 4.143 g, 4.711 mL) of diethyl phosphite were added and stirred at EtOH reflux for 12h. The crude obtained was purified by silica column chromatography (30% of ethyl acetate in hexane) and subsequently recrystalized in ethyl acetate at 4 °C, to afford 5.249 g (20.25 mmol) of diethyl (4-nitrophenyl)phosphonate as a brown oil (81%), Rf = 0.39 (50:50 EtOAc/hexane). ^1^H NMR (300 MHz, CDCl_3_) *δ*: 1.16–1.20 (m, 6 H, 2 CH_3_), 3.92–4.09 (m, 4 H, 2 CH_2_), 7.82–7.87 (m, 2 H), 8.12–8.15 (m, 2 H) ppm; ^13^C {^1^H} NMR (75 MHz, CDCl_3_) *δ*: 16.2 (d, ^3^*J*_CP_ = 6.3 Hz, 2 CH_3_), 62.7 (d, ^2^*J*_CP_ = 5.7 Hz, 2 CH_2_), 123.2 (d, ^3^*J*_CP_ = 15.3 Hz, 2 HC), 132.9 (d, ^2^*J*_CP_ = 10.5 Hz, 2 HC), 135.7 (d, ^1^*J*_CP_ = 187.2 Hz, C), 150.1 (d, ^4^*J*_CP_ = 3.8 Hz, C) ppm;^31^P {^1^H} NMR (120 MHz, CDCl_3_) *δ*: 16.0 ppm.

Subsequently, this derivative was hydrogenated with RANEY^®^ Nickel (approx. 5 g) in 60 mL of MeOH at 80 psi at room temperature during 12 h. The reaction mixture was then filtered off through a pad of cellite and evaporate to dryness to obtain 4.55 g (19.85 mmol) of diethyl (4-aminophenyl)phosphonate **1b** as a white solid (98%) mp 125–127 °C (ethyl acetate/hexane). ^1^H NMR (300 MHz, CDCl_3_) *δ*: 1.22–1.26 (m, 2 CH_3_), 3.93–4.06 (m, 2 CH_2_), 4.27 (sa, 2 H, NH_2_), 6.61–6.64 (m, 2 H), 7.47–7.52 (m, 2 H) ppm; ^13^C {^1^H} NMR (75 MHz, CDCl_3_) *δ*: 16.3 (d, ^3^*J*_CP_ = 6.6 Hz, 2 CH_3_), 61.7 (d, ^2^*J*_CP_ = 5.2 Hz, 2 CH), 114.0 (d, ^3^*J*_CP_ = 17.2 Hz, 2 HC), 115.0 (d, ^1^*J*_CP_ = 188.4 Hz, C), 133.5 (d, ^3^*J*_CP_ = 11.3 Hz, 2 HC), 150.8 (C) ppm; ^31^P {^1^H} NMR (120 MHz, CDCl_3_) *δ*: 22.3 ppm. 

#### 3.1.4. Synthesis of Quinolinyl Phosphonates

General procedure. To a solution of the freshly distilled aldehyde (1 mmol) and aminophenyl phosphonate (1 mmol) in CHCl_3_ (20 mL), the styrene derivative (1.5 mmol) was added in presence of molecular sieves (4 Å) and one drop of BF_3_·Et_2_O (1.5 mmol, 0.19 mL). The reaction was refluxed for 16 h. The reaction mixture was washed with a 2M aqueous solution of NaOH (50 mL) and water (50 mL), extracted with dichloromethane (2 × 25 mL), and dried over anhydrous MgSO_4_. The solvent was removed under vacuum affording an oil that was purified by silica gel flash column chromatography using an elution of 20–80% ethyl acetate-hexane to afford products **7**.

*Diethyl (2,4-diphenylquinolin-8-yl)phosphonate* (**7a**). The general procedure was followed using aminophenylphosphonate **1a** (1 mmol, 0.23 g), benzaldehyde (1 mmol, 0.10 mL), styrene (1.5 mmol, 0.17 mL) and BF_3·_Et_2_O affording 0.31 g (74%) of a white solid identified as **7a**, mp 118–120 °C (ethyl acetate/hexane). ^1^H NMR (300 MHz, CDCl_3_) *δ*: 1.32–1.36 (m, 6 H, 2 CH_3_), 4.26–4.44 (m, 4 H, 2 CH_2_), 7.45–7.57 (m, 9 H), 7.92 (s, 1 H), 8.06–8.08 (d, ^3^*J*_HH_ = 8.4 Hz, 1 H) 8.37–8.43 (m, 3 H) ppm; ^13^C {^1^H} NMR (75 MHz, CDCl_3_) *δ*: 16.7 (d, ^3^*J*_CP_ = 6.6 Hz, 2 CH_3_), 62.5 (d, ^2^*J*_CP_ = 5.7 Hz, 2 CH_2_), 119.3 (HC), 125.5 (d, ^3^*J*_CP_ = 16.2 Hz, HC), 126.0 (d, ^3^*J*_CP_ = 10.7 Hz, C), 128.0 (2 HC), 128.6 (d, ^1^*J*_CP_ = 193.7 Hz, C), 128.7 (HC), 128.8 (2 HC), 128.9 (2 HC), 129.7 (2 HC), 129.9 (HC), 130.7 (HC), 136.9 (d, ^2^*J*_CP_ = 7.4 Hz, HC), 138.2 (C), 139.0 (C), 148.8 (d, ^2^*J*_CP_ = 6.7 Hz, C), 149.6 (C), 156.6 (C) ppm; ^31^P {^1^H} NMR (120 MHz, CDCl_3_) *δ*: 18.5 ppm; HRMS (EI): calculated for C_25_H_24_NO_3_P [M]^+^ 417.1494; found 417.1494. Purity 99.30% (EtOH/Heptane = 10/90, Rt = 5.627 min).

*Diethyl (4-Phenyl-2-(fluorophenyl)quinolin-8-il) phosphonate* (**7b**). The general procedure was followed using aminophenylphosphonate **1a** (1 mmol, 0.23 g), 4-fluorobenzaldehyde (1 mmol, 0.11 mL), styrene (1.5 mmol, 0.17 mL) and BF_3·_Et_2_O affording 0.313 (72%) of a yellow solid identified as **7b**, mp 116–118 °C (ethyl acetate/hexane). ^1^H-RMN (300 MHz, CDCl_3_): *δ* = 1.24–1.29 (m, 6H, 2CH_3_), 4.17–4.37 (m, 2H, CH_2_), 7.10–7.16 (m, 2 H), 7.39–7.50 (m, 6 H), 7.79 (s, 1 H), 7.97–8.00 (m, 1 H), 8.26–8.33 (m, 3 H) ppm. ^13^C {^1^H} NMR (75 MHz, CDCl_3_): *δ* = 16.4 (2 CH_3_), 62.2 (2 OCH_2_), 115.5 (d, ^2^*J*_CF_ = 21.6 Hz, 2HC), 118.7 (HC), 125.3 (d, ^3^*J*_CP_ = 16.2 Hz, HC), 125.7 (d, ^3^*J*_CP_ = 10.8 Hz, C), 128,2 (d, ^1^*J*_CP_ = 188.5 Hz, C), 128.6 (HC), 128.7 (2HC), 129.4 (2HC), 129.7 (d, ^3^*J*_CF_ = 8.5 Hz, 2HC), 130.6 (HC), 135.0 (C), 136.7 (d, ^2^*J*_CP_ = 7.4 Hz, HC), 138.0 (C), 148.6 (d, ^2^*J*_CP_ = 6.8 Hz, C), 149.6 (C), 155.3 (C), 163.9 (d, ^1^*J*_CF_ = 249.8 Hz, C-F) ppm. ^31^P {^1^H} NMR (120 MHz, CDCl_3_): *δ* = 18.5 ppm. ^19^F-RMN (282 MHz, CDCl_3_): *δ* =− 112.0 ppm. IR: *υ* = 2982, 1588, 1225, 1023, 963, 771 cm^–1^. HRMS (CI): calculated for C_25_H_23_NO_3_PF [M]^+^ 435.1400; found 435.1401. Purity 98.60% (EtOH/Heptane = 10/90, Rt = 6.210 min).

*Diethyl (2-(4-methoxyphenyl)-4-phenylquinolin-8-yl)phosphonate* (**7c**). The general procedure was followed using aminophenylphosphonate **1a** (1 mmol, 0.23 g), *p*-anisaldehyde (1 mmol, 0.12 mL), styrene (1.5 mmol, 0.17 mL) and BF_3·_Et_2_O affording 0.18 g (41%) of a white solid identified as **7c**, mp 120–122 °C (ethyl acetate/hexane). ^1^H NMR (400 MHz, CDCl_3_) *δ*: 1.23–1.27 (m, 6 H, 2 CH_3_), 3.86 (s, 3 H, OCH_3_), 4.16–4.35 (m, 4 H, 2 OCH_2_), 6.93–6.95 (m, 1 H), 7.31–7.48 (m, 7 H), 7.79–7.82 (m, 2 H), 7.98–8.00 (m, 2 H), 8.28–8.34 (m, H) ppm; ^13^C {^1^H} NMR (100 MHz, CDCl_3_) *δ* = 16.5 (d, ^3^J_CP_ = 6.7 Hz, 2 CH_3_), 55.4 (OCH_3_), 62.3 (d, ^2^*J*_CP_ = 5.7 Hz, 2 OCH_2_), 112.6 (HC), 116.3 (HC), 119.2 (HC), 120.0 (HC), 125.4 (d, ^3^*J*_CP_ = 16.3 Hz, HC), 125.9 (d, ^2^*J*_CP_ = 10.7 Hz, C), 128.5 (d, ^1^*J*_CP_ = 188.5 Hz, C), 128.6 (HC), 128.7 (2 HC), 129.5 (2 HC), 129.6 (HC), 130.6 (HC), 136.9 (d, ^2^*J*_CP_ = 7.5 Hz, HC), 138.0 (C), 140.3 (C), 148.5 (d, ^2^*J*_CP_ = 6.8 Hz, C), 149.4 (C), 156.1 (C), 160.1 (C) ppm; ^31^P {^1^H} NMR (120 MHz, CDCl_3_) *δ*= 18,7 ppm. HRMS (EI): calculated for C_26_H_26_NO_4_P [M]^+^ 447.1599; found 447.1597. Purity 99.02% (EtOH/Heptane = 10/90, Rt = 6.765 min).

*Diethyl (2-(2-methoxyphenyl)-4-phenylquinolin-8-yl)phosphonate* (**7d**). The general procedure was followed using aminophenylphosphonate **1a** (1 mmol, 0.23 g), o-anisaldehyde (1 mmol, 0.12 mL), styrene (1.5 mmol, 0.17 mL) and BF_3·_Et_2_O affording 0.21 g (48%) of a white solid identified as **7d**, mp 121–123 °C (ethyl acetate/hexane). ^1^H NMR (400 MHz, CDCl_3_) *δ*: 1.25–1.28 (m, 6 H, 2 CH_3_), 3.81 (s, 3 H, OCH_3_), 4.17–4.35 (m, 4 H, 2 OCH_2_), 6.95–6.97 (m, 2 H), 7.36–7.49 (m, 6 H), 7.77 (s, 1 H), 7.95–7.97 (d, ^3^*J*_HH_ = 8.4 Hz, 1 H), 8.26–8.31 (m, 3 H) ppm; ^13^C {^1^H} NMR (100 MHz, CDCl_3_) *δ* = 16.5 (d, ^3^*J*_CP_ = 6.6 Hz, 2 CH_3_), 55.4 (OCH_3_), 62.3 (d, ^2^J_CP_ = 5.5 Hz, 2 OCH_2_), 114.1 (3 HC), 118.6 (HC), 124.9 (d, ^3^*J*_CP_ = 16.2 Hz, HC), 125.6 (d, ^3^*J*_CP_ = 10.7 Hz, C), 128.1 (d, ^1^*J*_CP_ = 187.9 Hz, C), 128.5 (HC), 128.6 (3 HC), 129.3 (HC), 129.5 (HC), 130.6 (HC), 131.5 (C), 136.7 (d, ^2^*J*_CP_ = 7.5 Hz, HC), 138.2 (C), 148.7 (d, ^2^*J*_CP_ = 6.7 Hz, C), 149.2 (C), 156.1 (C), 161.1 (C) ppm; ^31^P {^1^H} NMR (120 MHz, CDCl_3_) *δ*= 18,8ppm. HRMS (EI): calculated for C_26_H_26_NO_4_P [M]^+^ 447.1599; found 447.1597. Purity 97.30% (EtOH/Heptane = 10/90, Rt = 5.704 min).

*Diethyl (2-(naphthalen-2-yl)-4-phenylquinolin-8-yl)phosphonate* (**7e**). The general procedure was followed using aminophenylphosphonate **1a** (1 mmol, 0.23 g), 2-naphthaldehyde (1 mmol, 0.13 mL), styrene (1.5 mmol, 0.17 mL) and BF_3·_Et_2_O affording 0.30 g (64%) of a yellow solid identified as **7e**, mp 135–137 °C (ethyl acetate/hexane). ^1^H NMR (300 MHz, CDCl_3_) *δ*: 1.34–1.39 (m, 6 H, 2 CH_3_), 4.30–4.48 (m, 4 H, 2 CH_2_), 7.53–7.58 (m, 8 H), 7.90–8.11 (m, 5 H), 8.38–8.46 (m, 1 H), 8.65–8.75 (m, 2 H) ppm; ^13^C {^1^H} NMR (75 MHz, CDCl_3_) *δ*: 16.7 (d, ^3^*J*_CP_ = 6.5 Hz, 2 CH_3_), 62.6 (d, ^2^*J*_CP_ = 5.3 Hz, 2 CH_2_), 119.5 (HC), 125.5 (HC), 125.6 (HC), 125.7 (HC), 126.1 (d, ^3^*J*_CP_ = 10.6 Hz, C), 126.5 (HC), 127.1 (HC), 127.4 (HC), 127.6 (HC), 128.6 (HC), 128.7 (d, ^1^*J*_CP_ = 187.5 Hz, C), 128.8 (HC), 128.9 (2 HC), 129.0 (HC), 129.7 (2 HC), 130.7 (HC), 133.5 (C), 134.3 (C), 136.5 (C), 137.0 (d, ^2^*J*_CP_ = 7.3 Hz, HC), 138.2 (C), 148.9 (d, ^2^*J*_CP_ = 6.3 Hz C), 149.7 (C), 156.5 (C) ppm; ^31^P {^1^H} NMR (120 MHz, CDCl_3_) *δ*: 18.6 ppm; HRMS (EI): calculated for C_29_H_26_NO_3_P [M]^+^ 467.1650; found 467.1649. Purity 99.91% (EtOH/Heptane = 10/90, Rt = 6.136 min).

*Diethyl (2-(naphthalen-1-yl)-4-phenylquinolin-8-yl)phosphonate* (**7f**). The general procedure was followed using aminophenylphosphonate **1a** (1 mmol, 0.23 g), 1-naphthaldehyde (1 mmol, 0.13 mL), styrene (1.5 mmol, 0.17 mL) and BF_3·_Et_2_O affording 0.39 g (83%) of a yellow solid identified as **7f**, mp 140–142 °C (ethyl acetate/hexane). ^1^H NMR (300 MHz, CDCl_3_) *δ*: 1.16–1.20 (m, 6 H, 2 CH_3_), 4.19–4.30 (m, 4 H, 2 CH_2_), 7.51–7.62 (m, 9 H), 7.77 (s, 1 H), 7.86–7.97 (m, 3 H), 8.15–8.17 (d, ^3^J_HH_ = 8.4 Hz, 1 H), 8.39–8.44 (m, 1 H), 8.58–8.60 (d, ^3^*J*_HH_ = 8.3 Hz, 1 H) ppm; ^13^C {^1^H} NMR (75 MHz, CDCl_3_) *δ*: 16.5 (d, ^3^*J*_CP_ = 6.1 Hz, 2 CH_3_), 62.9 (d, ^2^*J*_CP_ = 5.3 Hz, 2 CH_2_), 124.0 (HC), 125.4 (HC), 125.7 (C), 125.8 (HC), 125.9 (HC), 126.2 (HC), 126.5 (HC), 126.9 (HC), 128.4 (HC), 128.8 (HC), 128.9 (2 HC), 129.5 (d, ^1^*J*_CP_ = 191.1 Hz, C), 129.6 (HC), 129.8 (2 HC), 130.6 (HC), 131.1 (C), 134.0 (C), 136.5 ppm (d, ^2^*J*_CP_ = 6.6 Hz, HC), 137.9 (C), 138.1 (C), 148.7 ppm (d, ^2^*J*_CP_ = 5.8 Hz, C), 149.2 (C), 159.0 (C) ppm; ^31^P {^1^H} NMR (120 MHz, CDCl_3_) *δ*: 17.2 ppm; HRMS (EI): calculated for C_29_H_26_NO_3_P [M]^+^ 467.1650; found 467.1651. Purity 99.77% (EtOH/Heptane = 10/90, Rt = 7.120 min).

*Diethyl (4-(4-fluorophenyl)-2-phenylquinolin-8-yl))phosphonate* (**7g**). The general procedure was followed using aminophenylphosphonate **1a** (1 mmol, 0.23 g), benzaldehyde (1 mmol, 0.10 mL), 4-fluorostyrene (1.5 mmol, 0.18 mL) and BF_3_·Et_2_O affording 0.26 g (60%) of a white solid identified as **7g**, mp 172–174 °C (ethyl acetate/hexane). ^1^H NMR (300 MHz, CDCl_3_) *δ*: 1.32–1.37 (m, 6 H, 2 CH_3_), 4.27–4.44 (m, 4 H, 2 CH_2_), 7.26–7.29 (m, 2 H), 7.48- 7.56 (m, 6 H), 7.89 (s, 1 H), 8.01(d, ^3^*J*_HP_ = 8.4 Hz, 1 H), 8.37–8.44 (m, 3 H) ppm; ^13^C {^1^H} NMR (75 MHz, CDCl_3_) *δ*: 16.6 (d, ^3^*J*_CP_ = 6.6 Hz, 2 CH_3_), 62.5 (d, ^2^*J*_CP_ = 5.8 Hz, 2 CH_2_), 115.9 (d, ^2^*J*_CF_ = 21.6 Hz, 2 HC), 119.3 (HC), 125.6 (d, ^3^*J*_CP_ = 16.2 Hz, HC), 126.0 (d, ^3^*J*_CP_ = 10.7 Hz, C), 128.0 (2 HC), 128.5 (d, ^1^*J*_CP_ = 188.5 Hz, C), 128.9 (2 HC), 130.0 (HC), 130.4 (HC), 131.3 (HC), 131.4 (HC), 134.1 (C), 136.9 (d, ^2^*J*_CP_ = 7.4 Hz, HC), 138.8 (C), 148.5 (C), 148.7 (d, ^2^*J*_CP_ = 6.8 Hz, C), 156.6 (C), 163.1 (d, ^1^*J*_CF_ = 248.6 Hz, C-F) ppm; ^31^P {^1^H} NMR (120 MHz, CDCl_3_) *δ*: 18.4 ppm; ^19^F NMR (282 MHz, CDCl_3_) *δ*: −113.3 ppm. HRMS (EI): calculated for C_25_H_23_FNO_3_P [M]^+^ 435.1400; found 435.1402. Purity 95.92% (EtOH/Heptane = 10/90, Rt = 6.038 min).

*Diethyl (2,4-bis(4-fluorophenyl)quinolin-8-yl)phosphonate* (**7h**). The general procedure was followed using aminophenylphosphonate **1a** (1 mmol, 0.23 g), 4-fluorobenzaldehyde (1 mmol, 0.11 mL), 4-fluorostyrene (1.5 mmol, 0.18 mL) and BF_3·_Et_2_O affording 0.29 (68%) of a white solid identified as **7h**, mp 121–123 °C (ethyl acetate/hexane). ^1^H NMR (300 MHz, CDCl_3_) *δ*: 1.32–1.36 (m, 6 H, 2 CH_3_), 4.25–4.42 (m, 4 H, 2 CH_2_), 7.18–7.28 (m, 4 H), 7.47–7.55 (m, 3 H), 7.83 (s, 1 H), 8.02 (d, ^3^*J*_HP_ = 8.4 Hz, 1 H), 8.33–8.41 (m, 3 H) ppm; ^13^C {^1^H} NMR (75 MHz, CDCl_3_) *δ*: 16.6 (d, ^3^*J*_CP_ = 6.4 Hz, 2 CH_3_), 62.5 (d, ^2^J_CP_ = 5.5 Hz, 2 CH_2_), 115.8 (d, ^2^*J*_CF_ = 21.6 Hz, 2 HC), 116.1 (d, ^2^*J*_CF_ = 21.6 Hz, HC), 118.9 (HC), 125.7 (d, ^3^*J*_CP_ = 15.8 Hz, HC), 125.9 (C), 128.7 (d, ^1^*J*_CP_ = 188.8 Hz, C), 129.9 (d, ^3^*J*_CF_ = 8.3 Hz, 2 HC), 130.4 (HC), 131.4 (d, ^3^*J*_CF_ = 8.2 Hz, 2 HC), 134.0 (C), 135.0 (C), 137.0 (d, ^2^*J*_CP_ = 7.4 Hz, HC), 148.7 (C), 155.5 (C), 163.2 (d, ^1^*J*_CF_ = 248.6 Hz, C-F), 164.2 (d, ^1^*J*_CF_ = 249.8 Hz, C-F) ppm; ^31^P {^1^H} NMR (120 MHz, CDCl_3_) *δ*: 18.3 ppm; ^19^F NMR (282 MHz, CDCl_3_) *δ*: −111.9 and −113.1 (m) ppm.HRMS (EI): calculated for C_25_H_22_F_2_NO_3_P [M]^+^ 453.1305; found 453.1307. Purity 99.60% (EtOH/Heptane = 10/90, Rt = 5.720 min).

*Diethyl (2-(3,4-difluorophenyl)-4-(4-fluorophenyl)quinolin-8-yl)phosphonate* (**7i**). The general procedure was followed using aminophenylphosphonate **1a** (1 mmol, 0.23 g), 3,4-difluorobenzaldehyde (1 mmol, 0.11 mL), 4-fluorostyrene (1.5 mmol, 0.18 mL) and BF_3·_Et_2_O affording 0.20 g (43%) of a white solid identified as **7i**, mp 159–161 °C (ethyl acetate/hexane). ^1^H NMR (300 MHz, CDCl_3_) *δ*: 1.33–1.38 (m, 6 H, 2 CH_3_), 4.21–4.44 (m, 4 H, 2 CH_2_), 7.23–7.58 (m, 6 H), 7.81 (s, 1 H), 8.01–8.11 (m, 2 H), 8.25–8.44 (m, 2 H) ppm; ^13^C {^1^H} NMR (75 MHz, CDCl_3_) *δ*: 16.7 (d, ^3^*J*_CP_ = 6.5 Hz, 2 CH_3_), 62.5 (d, ^2^*J*_CP_ = 5.8 Hz, 2 CH_2_), 116.1 (d, ^2^*J*_CF_ = 21.7 Hz, 2 HC), 117.0 (d, ^2^*J*_CF_ = 18.7 Hz, HC), 117.7 (d, ^2^*J*_CF_ = 17.4 Hz, HC), 118.7 (HC), 120.5 (C), 124.0 (HC), 126.0 (d, ^3^*J*_CP_ = 16.0 Hz, HC), 128.7 (d, ^1^*J*_CP_ = 188.8 Hz, C), 130.5 (HC), 131.3 (HC), 131.4 (HC), 133.9 (C), 136.0 (C), 137.4 (HC), 148.6 (d, ^2^*J*_CP_ = 6.4 Hz, C), 149.1 (C), 151.0 (dd, ^2^*J*_CF_ = 12.5 Hz, ^1^*J*_CF_ = 239.2 Hz, C), 151.8 (dd, ^2^*J*_CF_ = 12.5 Hz, ^1^*J*_CF_ = 247.6 Hz, C), 154.2 (C), 163.2 (d, ^1^*J*_CF_ = 249.0 Hz, C-F) ppm; ^31^P {^1^H} NMR (120 MHz, CDCl_3_) *δ*: 18.3 ppm; ^19^F NMR (282 MHz, CDCl_3_) *δ*: −112.9, −136.4 and −137.3 (m) ppm. HRMS (EI): calculated for C_25_H_21_F_3_NO_3_P [M]^+^ 471.1211; found 471.1215. Purity 99.47% (EtOH/Heptane = 10/90, Rt = 5.892 min).

*Diethyl (2-(4-fluorophenyl)-4-(p-tolyl)quinolin-8-yl)phosphonate* (**7j**). The general procedure was followed using aminophenylphosphonate **1a** (1 mmol, 0.23 g), 4-fluorobenzaldehyde (1 mmol, 0.11 mL), 4-methylstyrene (1.5 mmol, 0.20 mL) and BF_3·_Et_2_O affording 0.23 g (52%) of a white solid identified as **7j**, mp 139–141 °C (ethyl acetate/hexane). ^1^H NMR (300 MHz, CDCl_3_) *δ*: 1.32–1.36 (m, 6 H, 2 CH_3_), 2.48 (s, 3 H, CH_3_), 4.22–4.44 (m, 4 H, 2 CH_2_), 7.17–7.23 (m, 2 H), 7.35–7.43 (m, 4 H), 7.47–7.53 (m, 1 H), 7.86 (s, 1 H), 8.08–8.11 (m, 1 H), 8.32–8.40 (m, 3 H) ppm; ^13^C {^1^H} NMR (75 MHz, CDCl_3_) *δ*: 16.6 (d, ^3^*J*_CP_ = 6.5 Hz, 2 CH_3_), 21.5 (CH_3_), 62.5 (m, 2 CH_2_), 115.9 (d, ^2^*J*_CF_ = 21.7 Hz, 2 HC), 118.9 (HC), 125.5 (d, ^3^*J*_CP_ = 18.0 Hz, HC), 126.0 (d, ^3^*J*_CP_ = 10.7 Hz, C), 128.2 (d, ^1^*J*_CP_ = 166.3 Hz, C), 129.6 (3 HC), 129.8 (HC), 129.9 (HC), 130.9 (2 HC), 135.1 (2 C), 136.9 (HC), 138.9 (C), 148.7 (C), 150.1 (d, ^2^*J*_CP_ = 17.2 Hz, C), 155.4 (C), 164.2 (d, ^1^*J*_CF_ = 249.8 Hz, C-F) ppm; ^31^P {^1^H} NMR (120 MHz, CDCl_3_) *δ*: 18.6 ppm; ^19^F NMR (282 MHz, CDCl_3_) *δ*: −112.0 ppm. HRMS (EI): calculated for C_26_H_25_FNO_3_P [M]^+^ 449.1556; found 449.1562. Purity 97.07% (EtOH/Heptane = 10/90, Rt = 5.166 min). 

*Diethyl (2-(3,4-difluorophenyl)-4-(p-tolyl)quinolin-8-yl)phosphonate* (**7k**). The general procedure was followed using aminophenylphosphonate **1a** (1 mmol, 0.23 g), 3,4-difluorobenzaldehyde (1 mmol, 0.11 mL), 4-methylstyrene (1.5 mmol, 0.20 mL) and BF_3·_Et_2_O affording 0.32 g (47%) of a white solid identified as **7k**, mp 133–135 °C (ethyl acetate/hexane). ^1^H NMR (300 MHz, CDCl_3_) *δ*: 1.34–1.39 (m, 6 H, 2 CH_3_), 2.49 (s, 1 H, CH_3_), 4.21–4.43 (m, 4 H, 2 CH_2_), 7.28–7.31 (m, 1 H), 7.36–7.43 (m, 4 H), 7.49–7.56 (m, 1 H), 7.82 (s, 1 H), 8.07–8.12 (m, 2 H), 8.26–8.43 (m, 2 H) ppm; ^13^C {^1^H} NMR (75 MHz, CDCl_3_) *δ*: 16.7 (d, ^3^*J*_CP_ = 6.5Hz, 2 CH_3_), 21.5 (CH_3_), 62.4 (d, ^2^*J*_CP_ = 5.8 Hz, 2 CH_2_), 117.0 (d, ^2^*J*_CF_ = 18.9 Hz, HC), 117.6 (d, ^2^*J*_CF_ = 17.4 Hz, HC), 118.6 (HC), 123.9 (dd, ^3^*J*_CF_ = 6.5 Hz, ^4^*J*_C-F_ = 3.3 Hz, HC), 125.8 (d, ^3^*J*_CP_ = 16.2 Hz, HC), 126.2 (d, ^3^*J*_CP_ = 10.6 Hz, C), 128.5 (d, ^1^*J*_CP_ = 188.5 Hz, C), 129.5 (2 HC), 129.6 (2 HC), 130.9 (C), 135.0 (C), 136.3 (C), 137.2 (d, ^2^*J*_CP_ = 7.6 Hz, HC), 139.0 (C), 148.7 (d, ^2^*J*_CP_ = 6.6 Hz, C), 150.2 (C), 150.8 (dd, ^1^*J*_CF_ = 247.4 Hz, ^2^*J*_CF_ = 12.1 Hz, C), 151.7 (dd, ^1^*J*_CF_ = 250.7 Hz, ^2^*J*_CF_ = 12.3 Hz, C), 153.5 (C) ppm. ^31^P {^1^H} NMR (120 MHz, CDCl_3_) *δ*: 18.5 ppm; ^19^F NMR (282 MHz, CDCl_3_) *δ*: −136.65 and −137.50 (m) ppm. HRMS (EI): calculated for C_26_H_24_F_2_NO_3_P [M]^+^ 467.1462; found 467.1465. Purity 98.21% (EtOH/Heptane = 10/90, Rt = 5.307 min).

*Diisopropyl (2-(4-fluorophenyl)-4-phenylquinolin-8-yl)phosphonate* (**7l**). The general procedure was followed using aminophenylphosphonate **1b** (1 mmol, 0.23 g), 4-fluorobenzaldehyde (1 mmol, 0.11 mL), styrene (1.5 mmol, 0.17 mL) and BF_3·_Et_2_O affording 0.16 g (34%) of a white solid identified as **7l**, mp 107–109 °C (ethyl acetate/hexane). ^1^H NMR (300 MHz, CDCl_3_) *δ*: 1.15 (d, ^3^J_HH_ = 6.2 Hz, 2 CH_3_), 1.33 (d, ^3^J_HH_ = 6.2 Hz, 2 CH_3_), 4.90–4.98 (m, 2 H, 2 CH), 7.10–7.14 (m, 2 H), 7.39–7.50 (m, 6 H), 7.77 (s, 1 H), 7.96–7.98 (m, 1 H), 8.27–8.33 (m, 3 H) ppm; ^13^C {^1^H} NMR (75 MHz, CDCl_3_) *δ*: 23.9 (d, ^3^*J*_CP_ = 5.1 Hz, 2 CH_3_), 24.3 (d, ^3^*J*_CP_ = 3.8 Hz, 2 CH_3_), 70.7 (d, ^2^*J*_CP_ = 6.0 Hz, 2 CH), 115.6 (d, ^2^*J*_CF_ = 21.6 Hz, 2 HC), 118.6 (HC), 125.3 (d, ^3^*J*_CP_ = 16.2 Hz, HC), 125.7 (d, ^3^*J*_CP_ = 10.8 Hz, C), 128.6 (HC), 128.7 (2 HC), 129.4 (d, ^1^*J*_CP_ = 189.7 Hz, C), 129.5 (2 HC), 129.7 (d, ^3^*J*_CF_ = 8.4 Hz, 2 HC), 130.4 (HC), 135.1 (C), 136.8 (d, ^2^*J*_CP_ = 7.5 Hz, HC), 138.0 (C), 148.5 (d, ^2^*J*_CP_ = 6.7 Hz, C), 149.5 (C), 155.1 (C), 164.0 (d, ^1^*J*_CF_ = 249.6 Hz, C-F) ppm; ^31^P {^1^H} NMR (120 MHz, CDCl_3_) *δ*: 16.3 ppm; ^19^F NMR (282 MHz, CDCl_3_) *δ*: −112.2 ppm. HRMS (EI): calculated for C_27_H_27_FNO_3_P [M]^+^ 463.1713; found 463.1720. Purity 99.78% (EtOH/Heptane = 10/90, Rt = 4.631 min).

*Diisopropyl (2-(3,4-difluorophenyl)-4-phenylquinolin-8-yl)phosphonate* (**7m**). The general procedure was followed using aminophenylphosphonate **1b** (1 mmol, 0.23 g), 3,4-difluorobenzaldehyde (1 mmol, 0.11 mL), styrene (1.5 mmol, 0.17 mL) and BF_3·_Et_2_O affording 0.18 g (38%) of a white solid identified as **7m**, mp 127–129 °C (ethyl acetate/hexane). ^1^H NMR (300 MHz, CDCl_3_) *δ*: 1.15 (d, ^3^*J*_HH_ = 6.2 Hz, 2 CH_3_), 1.35 (d, ^3^*J*_HH_ = 6.2 Hz, 2 CH_3_), 4.88–4.96 (m, 2 H, 2 CH), 7.17–7.23 (m, 1 H), 7.40–7.49 (m, 6 H), 7.74 (s, 1 H), 7.95–7.98 (m, 1 H), 8.00–8.03 (m, H), 8.23–8.28 (m, H), 8.30–8.37 (m, H) ppm; ^13^C {^1^H} NMR (75 MHz, CDCl_3_) *δ*: 23.9 (d, ^3^*J*_CP_ = 5.0 Hz, 2 CH_3_), 24.3 (d, ^3^*J*_CP_ = 4.0 Hz, 2 CH_3_), 70.7 (d, ^2^*J*_CP_ = 6.0 Hz, 2 CH), 116.8 (d, ^2^*J*_CF_ = 18.7 Hz, HC), 117.3 (d, ^2^*J*_CF_ = 17.5 Hz, HC), 118.2 (HC), 123.6 (dd, ^3^*J*_CF_ = 6.6 Hz, ^4^*J*_CF_ = 3.5 Hz, HC), 125.6 (d, ^3^*J*_CP_ = 16.3 Hz, HC), 125.9 (d, ^3^*J*_CP_ = 10.6 Hz, C), 128.7 (2 HC), 129.5 (2 HC), 129.5 (d, ^1^*J*_CP_ = 189.3 Hz, C), 130.4 (HC), 130.6 (HC), 136.1 (dd, ^3^*J*_CF_ = 6.7 Hz, ^4^*J*_CF_ = 3.6 Hz, C), 137.1 (d, ^3^*J*_CF_ = 7.7 Hz, HC), 137.8 (C), 148.4 (d, ^2^*J*_CP_ = 6.4 Hz, C), 149.8 (C), 150.8 (dd, ^2^*J*_CF_ = 12.7 Hz, ^1^*J*_CF_ = 247.7 Hz, C), 151.6 (dd, ^2^*J*_CF_ = 12.7 Hz, ^1^*J*_CF_ = 251.7 Hz, C), 153.7 (C) ppm; ^31^P {^1^H} NMR (120 MHz, CDCl_3_) *δ*: 16.2 ppm; ^19^F NMR (282 MHz, CDCl_3_) *δ*: −136.7 and −137.7 ppm. HRMS (EI): calculated for C_27_H_26_F_2_NO_3_P [M]^+^ 481.1618; found 481.1625. Purity 99.99% (EtOH/Heptane = 10/90, Rt = 4.799 min).

*Diisopropyl (2,4-bis(4-fluorophenyl)quinolin-8-yl)phosphonate* (**7n**). The general procedure was followed using aminophenylphosphonate **1b** (1 mmol, 0.23 g), 4-fluorobenzaldehyde (1 mmol, 0.11 mL), 4-fluorostyrene (1.5 mmol, 0.18 mL) and BF_3·_Et_2_O affording 0.30 g (62%) of a white solid identified as **7n**, mp 113–115 °C (ethyl acetate/hexane). ^1^H NMR (300 MHz, CDCl_3_) *δ*: 1.15 (d, ^3^*J*_HH_ = 6.2 Hz, 2 CH_3_), 1.35 (d, ^3^*J*_HH_ = 6.1 Hz, 2 CH_3_), 4.90–4.99 (m, 2 H, 2 CH), 7.11–7.20 (m, 4 H), 7.40–7.46 (m, 3 H), 7.75 (s, 1 H), 7.91–7.93 (m, 1 H), 8.29–8.35 (m, 3 H) ppm; ^13^C {^1^H} NMR (75 MHz, CDCl_3_) *δ*: 23.9 (d, ^3^*J*_CP_ = 5.1 Hz, 2 CH_3_), 24.3 (d, ^3^*J*_CP_ = 3.8 Hz, 2 CH_3_), 70.7 (d, ^2^J_CP_ = 6.0 Hz, 2 CH), 115.6 (d, ^2^*J*_CF_ = 21.5 Hz, 2 HC), 115.8 (d, ^2^*J*_CF_ = 21.6 Hz, 2 HC), 118.6 (HC), 125.5 (d, ^3^*J*_CP_ = 16.1 Hz, HC), 125.7 (d, ^3^*J*_CP_ = 10.6 Hz, C), 129.6 (d, ^1^*J*_CP_ = 189.7 Hz, C), 131.2 (d, ^3^*J*_CF_ = 8.1 Hz, 2 HC), 134.0 (C), 134.9 (C), 136.8 (d, ^2^*J*_CP_ = 7.4 Hz, HC), 148.4 (C), 148.5 (d, ^2^*J*_CP_ = 6.8 Hz, C), 150.1 (C), 163.0 (d, ^1^*J*_CF_ = 248.3 Hz, C-F), 164.0 (d, ^1^*J*_CF_ = 249.7 Hz, C-F) ppm; ^31^P {^1^H} NMR (120 MHz, CDCl_3_) *δ*: 16.1 ppm; ^19^F NMR (282 MHz, CDCl_3_) *δ*: −112.1 and −113.3 ppm. HRMS (EI): calculated for C_27_H_26_F_2_NO_3_P [M]^+^ 481.1618; found 481.1632. Purity 99.99% (EtOH/Heptane = 10/90, Rt = 4.696 min).

*Diisopropyl (2-(3,4-difluorophenyl)-4-(4-fluorophenyl)quinolin-8-yl)phosphonate* (**7o**). The general procedure was followed using aminophenylphosphonate **1b** (1 mmol, 0.23 g), 3,4-difluorobenzaldehyde (1 mmol, 0.11 mL), 4-fluorostyrene (1.5 mmol, 0.18 mL) and BF_3·_Et_2_O affording 0.44 g (88%) of a white solid identified as **7o**, mp 144–146 °C (ethyl acetate/hexane). ^1^H NMR (300 MHz, CDCl_3_) *δ*: 1.06 (d, ^3^J_HH_ = 6.2 Hz, 2 CH_3_), 1.25 (d, ^3^*J*_HH_ = 6.2 Hz, 2 CH_3_), 4.79–4.87 (m, 2 H, 2 CH), 7.17–7.23 (m, 1 H), 7.40–7.49 (m, 6 H), 7.74 (s, 1 H), 7.95–7.98 (m, 1 H), 8.00–8.03 (m, H), 8.23–8.28 (m, H), 8.30–8.37 (m, H) ppm; ^13^C {^1^H} NMR (75 MHz, CDCl_3_) *δ*: 23.8 (d, ^3^*J*_CP_ = 5.0 Hz, 2 CH_3_), 24.1 (d, ^3^*J*_CP_ = 3.9 Hz, 2 CH_3_), 70.7 (d, ^2^*J*_CP_ = 6.0 Hz, 2 CH), 115.7 (d, ^3^*J*_CF_ = 20.6 Hz, 2 HC), 116.8 (d, ^2^*J*_CF_ = 18.7 Hz, HC), 117.2 (d, ^2^J_CF_ = 17.5 Hz, HC), 118.2 (HC), 123.6 (dd, ^3^*J*_CF_ = 6.5 Hz, ^4^*J*_CF_ = 3.3 Hz, HC), 125.7 (d, ^3^*J*_CP_ = 17.6 Hz, HC), 125.7 (C), 129.4 (d, ^1^*J*_CP_ = 189.5 Hz, C), 129.9 (HC), 131.1 (HC), 131.2 (2 HC), 133.6 (C), 135.8 (dd, ^3^*J*_CF_ = 5.7 Hz, ^4^*J*_CF_ = 3.5 Hz, C), 136.9 (d, ^3^*J*_CF_ = 7.6 Hz, HC), 148.3 (d, ^2^*J*_CP_ = 6.5 Hz, C), 148.6 (C), 150.6 (dd, ^2^*J*_CF_ = 12.6 Hz, ^1^*J*_CF_ = 247.8 Hz, C), 151.5 (dd, ^2^*J*_CF_ = 12.7 Hz, ^1^*J*_CF_ = 251.6 Hz, C), 153.6 (C), 162.9 (d, ^1^*J*_CF_ = 248.9 Hz, C-F), ppm; ^31^P {^1^H} NMR (120 MHz, CDCl_3_) *δ*: 15.9 ppm; ^19^F NMR (282 MHz, CDCl_3_) *δ*: −112.9, −136.6 and −137.7 (m) ppm. HRMS (EI): calculated for C_27_H_25_F_3_NO_3_P [M]^+^ 499.1524; found 499.1534. Purity 99.72% (EtOH/Heptane = 10/90, Rt = 4.826 min).

*Diisopropyl (2-(4-fluorophenyl)-4-(p-tolyl)quinolin-8-yl)phosphonate* (**7p**). The general procedure was followed using aminophenylphosphonate **1b** (1 mmol, 0.23 g), 4-fluorobenzaldehyde (1 mmol, 0.11 mL), 4-methylstyrene (1.5 mmol, 0.20 mL) and BF_3·_Et_2_O affording 0.28 g (58%) of a white solid identified as **7p**, mp 121–123 °C (ethyl acetate/hexane). ^1^H NMR (300 MHz, CDCl_3_) *δ*: 1.14 (d, ^3^*J*_HH_ = 6.1 Hz, 2 CH_3_), 1.33 (d, ^3^*J*_HH_ = 6.2 Hz, 2 CH_3_), 2.40 (CH_3_), 4.89–4.97 (m, 2 H, 2 CH), 7.09–7.13 (m, 2 H), 7.27–7.34 (m, 4 H), 7.37–7.42 (m, 1 H), 7.75 (s, 1 H), 7.98–8.00 (m, 1 H), 8.26–8.32 (m, 3 H) ppm; ^13^C {^1^H} NMR (75 MHz, CDCl_3_) *δ*: 21.3 (CH_3_), 23.9 (d, ^3^*J*_CP_ = 5.1 Hz, 2 CH_3_), 24.3 (d, ^3^*J*_CP_ = 3.8 Hz, 2 CH_3_), 70.6 (d, ^2^*J*_CP_ = 6.0 Hz, 2 CH), 115.5 (d, ^2^*J*_CF_ = 21.6 Hz, 2 HC), 118.5 (HC), 125.2 (d, ^3^*J*_CP_ = 16.2 Hz, HC), 125.8 (d, ^3^*J*_CP_ = 10.7 Hz, C), 129.4 (2 HC), 129.4 (2 HC), 129.7 (d, ^3^*J*_CF_ = 7.4 Hz, 2 HC), 130.0 (d, ^1^*J*_CP_ = 189.3 Hz, C), 130.5 (d, ^4^*J*_CP_ = 5.0 Hz, HC), 135.1 (d, ^4^*J*_CP_ = 4.7 Hz, C), 135.1 (C), 136.7 (d, ^2^*J*_CP_ = 7.5 Hz, HC), 138.6 (C), 148.6 (d, ^2^*J*_CP_ = 6.6 Hz, C), 149.6 (C), 155.1 (C), 164.0 (d, ^1^*J*_CF_ = 249.5 Hz, C) ppm; ^31^P {^1^H} NMR (120 MHz, CDCl_3_) *δ*: 16.4 ppm; ^19^F NMR (282 MHz, CDCl_3_) *δ*: −112.2 ppm. HRMS (EI): calculated for C_28_H_29_FNO_3_P [M]^+^ 477.1869; found 477.1876. Purity 99.48% (EtOH/Heptane = 10/90, Rt = 4.343 min).

*Diisopropyl (2-(3,4-difluorophenyl)-4-(p-tolyl)quinolin-8-yl)phosphonate* (**7q**). The general procedure was followed using aminophenylphosphonate **1b** (1 mmol, 0.23 g), 3,4-difluorobenzaldehyde (1 mmol, 0.11 mL), 4-methylstyrene (1.5 mmol, 0.20 mL) and BF_3·_Et_2_O affording 0.19 g (39%) of a white solid identified as **7q**, mp 140–142 °C (ethyl acetate/hexane). ^1^H NMR (300 MHz, CDCl_3_) *δ*: 1.15 (d, ^3^*J*_HH_ = 6.2 Hz, 2 CH_3_), 1.34 (d, ^3^*J*_HH_ = 6.2 Hz, 2 CH_3_), 2.39 (CH_3_), 4.88–4.96 (m, 2 H, 2 CH), 7.16–7.23 (m, 1 H), 7.26–7.33 (m, 4 H), 7.40–7.44 (m, 1 H), 7.72 (s, 1 H), 7.99–8.02 (m, 2 H), 8.23–8.36 (m, 2 H) ppm; ^13^C {^1^H} NMR (75 MHz, CDCl_3_) *δ*: 21.2 (CH_3_), 23.9 (d, ^3^*J*_CP_ = 5.0 Hz, 2 CH_3_), 24.3 (d, ^3^*J*_CP_ = 4.8 Hz, 2 CH_3_), 70.7 (d, ^2^*J*_CP_ = 5.9 Hz, 2 CH), 116.8 (d, ^2^*J*_CF_ = 18.7 Hz, HC), 117.3 (d, ^2^*J*_CF_ = 17.5 Hz, HC), 118.2 (HC), 123.6 (dd, ^3^*J*_CF_ = 6.6 Hz, ^4^*J*_CF_ = 3.4 Hz, HC), 125.5 (d, ^3^*J*_CP_ = 16.2 Hz, HC), 126.0 (d, ^3^*J*_CP_ = 10.4 Hz, C), 129.4 (2 HC), 129.4 (2 HC), 129.5 (d, ^1^*J*_CP_ = 188.1 Hz, C), 130.5 (HC), 134.9 (HC), 136.2 (dd, ^3^*J*_CF_ = 5.8 Hz, ^4^*J*_CF_ = 3.6 Hz, C), 137.1 (d, ^3^*J*_CF_ = 7.7 Hz, HC), 138.7 (C), 148.4 (d, ^2^*J*_CP_ = 6.4 Hz, C), 149.9 (C), 150.8 (dd, ^2^*J*_CF_ = 12.6 Hz, ^1^*J*_CF_ = 247.6 Hz, C), 151.6 (dd, ^2^*J*_CF_ = 12.7 Hz, ^1^*J*_CF_ = 251.3 Hz, C), 153.7 (C) ppm; ^31^P {^1^H} NMR (120 MHz, CDCl_3_) *δ*: 16.3 ppm; ^19^F NMR (282 MHz, CDCl_3_) *δ*: −136.8 and −137.7 ppm. HRMS (EI): calculated for C_28_H_28_F_2_NO_3_P [M]^+^ 495.1775; found 495.1784. Purity 99.99% (EtOH/Heptane = 10/90, Rt = 4.450 min).

*Diethyl (2-(4-fluorophenyl)-4-phenylquinolin-6-yl)phosphonate* (**7r**). The general procedure was followed using aminophenylphosphonate **1c** (1 mmol, 0.23 g), 4-fluorobenzaldehyde (1 mmol, 0.11 mL), styrene (1.5 mmol, 0.17 mL) and BF_3·_Et_2_O affording 0.40 g (91%) of a white solid identified as **7r**, mp 137–139 °C (ethyl acetate/hexane). ^1^H NMR (300 MHz, CDCl_3_) *δ*: 1.29–1.33 (m, 6 H, 2 CH_3_), 4.06–4.19 (m, 4 H, 2 CH_2_), 7.19–7.26 (m, 2 H), 7.53–7.60 (m, 5 H), 7.84 (s, 1 H), 8.00–8.05 (m, 1 H), 8.20–8.24 (m, 2 H), 8.26–8.29 (m, 1 H), 8.45–8.49 (m, 1 H) ppm; ^13^C {^1^H} NMR (75 MHz, CDCl_3_) *δ*: 16.3 (d, ^3^*J*_CP_ = 6.5 Hz, 2 CH_3_), 62.3 (d, ^2^*J*_CP_ = 5.5 Hz, 2 CH_2_), 115.9 (d, ^2^*J*_CF_ = 21.7 Hz, 2 HC), 119.8 (HC), 124.9 (d, ^3^*J*_CP_ = 17.3 Hz, C), 126.2 (d, ^1^*J*_CP_ = 189.7 Hz, C), 128.9 (2 HC), 128.9 (HC), 129.5 (2 HC), 129.6 (d, ^3^*J*_CF_ = 8.5 Hz, 2 HC), 130.4 (d, ^2^*J*_CP_ = 3.8 Hz, HC), 130.6 (HC), 131.6 (d, ^2^*J*_CP_ = 11.9 Hz, HC), 135.2 (C), 137.4 (C), 150.2 (d, ^4^*J*_CP_ = 3.0 Hz, C), 150.3 (C), 157.6 (C), 164.1 (d, ^1^*J*_CF_ = 250.2 Hz, C-F) ppm; ^31^P {^1^H} NMR (120 MHz, CDCl_3_) *δ*: 19.3 ppm; ^19^F NMR (282 MHz, CDCl_3_) *δ*: −111.8 ppm. HRMS (EI): calculated for C_25_H_23_FNO_3_P [M]^+^ 435.1400; found 435.1415. Purity 95.42% (EtOH/Heptane = 10/90, Rt = 5.523 min).

*Diethyl (2-(3,4-difluorophenyl)-4-phenylquinolin-6-yl)phosphonate* (**7s**). The general procedure was followed using aminophenylphosphonate **1c** (1 mmol, 0.23 g), 3,4-difluorobenzaldehyde (1 mmol, 0.11 mL), styrene (1.5 mmol, 0.17 mL) and BF_3·_Et_2_O affording 0.40 g (89%) of a white solid identified as **7s**, mp 134–136 °C (ethyl acetate/hexane). ^1^H NMR (300 MHz, CDCl_3_) *δ*: 1.28–1.33 (m, 6 H, 2 CH_3_), 4.03–4.21 (m, 4 H, 2 CH_2_), 7.29–7.32 (m, 1 H), 7.50–7.59 (m, 5 H), 7.80 (s, 1 H), 7.90–7.93 (m, 1 H), 7.99–8.14 (m, 2 H), 8.24–8.28 (m, 1 H), 8.44–8.50 (m, 1 H) ppm; ^13^C {^1^H} NMR (75 MHz, CDCl_3_) *δ*: 16.2 (d, ^3^*J*_CP_ = 6.5 Hz, 2 CH_3_), 62.3 (d, ^2^*J*_CP_ = 5.5 Hz, 2 CH_2_), 116.7 (d, ^2^*J*_CF_ = 18.5 Hz, HC), 117.6 (d, ^2^*J*_CF_ = 17.6 Hz, HC), 119.4 (HC), 123.7 (dd, ^3^*J*_CF_ = 6.7 Hz, ^4^*J*_CF_ = 3.5 Hz, HC), 125.1 (d, ^3^*J*_CP_ = 17.3 Hz, C), 126.7 (d, ^1^*J*_CP_ = 189.5 Hz, C), 128.9 (2 HC), 129.0 (HC), 129.4 (2 HC), 130.6 (d, ^2^*J*_CP_ = 24.5 Hz, C), 130.6 (HC), 131.6 (d, ^2^*J*_CP_ = 11.7 Hz, HC), 136.0 (dd, ^3^*J*_CF_ = 5.7 Hz, ^4^*J*_CF_ = 3.7 Hz, C), 137.2 (C), 150.1 (d, ^4^*J*_CP_ = 3.0 Hz, C), 150.6 (C), 150.7 (dd, ^2^*J*_CF_ = 12.7 Hz, ^1^*J*_CF_ = 248.4 Hz, C), 151.6 (dd, ^2^*J*_CF_ = 12.7 Hz, ^1^*J*_CF_ = 251.9 Hz, C), 156.2 (C) ppm; ^31^P {^1^H} NMR (120 MHz, CDCl_3_) *δ*: 19.1 ppm; ^19^F NMR (282 MHz, CDCl_3_) *δ*: −136.2 and −137.2 ppm. HRMS (EI): calculated for C_25_H_22_F_2_NO_3_P [M]^+^ 453.1305; found 453.1317. Purity 98.94% (EtOH/Heptane = 10/90, Rt = 5.605 min).

*Diethyl (2,4-bis(4-fluorophenyl)quinolin-6-yl)phosphonate* (**7t**). The general procedure was followed using aminophenylphosphonate **1c** (1 mmol, 0.23 g), 4-fluorobenzaldehyde (1 mmol, 0.11 mL), 4-fluorostyrene (1.5 mmol, 0.18 mL) and BF_3·_Et_2_O affording 0.35 g (78%) of a white solid identified as **7t**, mp 161–164 °C (ethyl acetate/hexane). ^1^H NMR (300 MHz, CDCl_3_) *δ*: 1.21–1.25 (m, 6 H, 2 CH_3_), 3.97–4.13 (m, 4 H, 2 CH_2_), 7.12–7.21 (m, 4 H), 7.43–7.47 (m, 2 H), 7.73 (s, 1 H), 7.91–7.96 (m, 1 H), 8.12–8.15 (m, 2 H), 8.18–8.21 (m, 1 H), 8.32–8.36 (m, 1 H) ppm; ^13^C {^1^H} NMR (75 MHz, CDCl_3_) *δ*: 16.3 (d, ^3^*J*_CP_ = 6.5 Hz, 2 CH_3_), 62.3 (d, ^2^*J*_CP_ = 5.5 Hz, 2 CH_2_), 115.9 (d, ^2^*J*_CF_ = 21.7 Hz, 2 HC), 116.1 (d, ^2^*J*_CF_ = 21.5 Hz, HC), 119.8 (HC), 124.8 (d, ^3^*J*_CP_ = 17.3 Hz, C), 126.5 (d, ^1^*J*_CP_ = 189.7 Hz, C), 129.6 (d, ^3^*J*_CF_ = 8.5 Hz, 2 HC), 130.5 (d, ^2^*J*_CP_ = 2.5 Hz, HC), 130.6 (d, ^2^*J*_CP_ = 1.8 Hz, HC), 131.2 (d, ^3^*J*_CF_ = 8.3 Hz, 2 HC), 131.4 (d, ^3^*J*_CP_ = 11.9 Hz, HC), 133.4 (C), 135.0 (C), 149.2 (C), 150.2 (C), 157.7 (C), 163.2 (d, ^1^*J*_CF_ = 249.1 Hz, C-F), 164.1 (d, ^1^*J*_CF_ = 250.2 Hz, C-F) ppm; ^31^P {^1^H} NMR (120 MHz, CDCl_3_) *δ*: 19.1 ppm; ^19^F NMR (282 MHz, CDCl_3_) *δ*: −114.5 and −116.4 ppm. HRMS (EI): calculated for C_25_H_22_F_2_NO_3_P [M]^+^ 453.1305; found 453.1317. Purity 95.51% (EtOH/Heptane = 10/90, Rt = 5.479 min).

*Diethyl (2-(3,4-difluorophenyl)-4-(4-fluorophenyl)quinolin-6-yl)phosphonate* (**7u**). The general procedure was followed using aminophenylphosphonate **1c** (1 mmol, 0.23 g), 3,4-difluorobenzaldehyde (1 mmol, 0.11 mL), 4-fluorostyrene (1.5 mmol, 0.18 mL) and BF_3·_Et_2_O affording 0.33 g (71%) of a white solid identified as **7u**, mp 130–132 °C (ethyl acetate/hexane). ^1^H NMR (300 MHz, CDCl_3_) *δ*: 1.22–1.25 (m, 6 H, 2 CH_3_), 3.97–4.14 (m, 4 H, 2 CH_2_), 7.17–7.27 (m, 3 H), 7.42–7.47 (m, 2 H), 7.71 (s, 1 H), 7.84–7.88 (m, 1 H), 7.92–7.98 (m, 1 H), 8.02–8.07 (m, 1 H), 8.17–8.21 (m, 1 H), 8.32–8.37 (m, 1 H) ppm; ^13^C {^1^H} NMR (75 MHz, CDCl_3_) *δ*: 16.3 (d, ^3^*J*_CP_ = 6.4 Hz, 2 CH_3_), 62.3 (d, ^2^*J*_CP_ = 5.1 Hz, 2 CH_2_), 116.1 (d, ^2^*J*_CF_ = 21.7 Hz, 2 HC), 116.8 (d, ^2^*J*_CF_ = 19.3 Hz, HC), 117.6 (d, ^2^*J*_CF_ = 17.4 Hz, HC), 119.5 (HC), 123.7 (dd, ^4^*J*_CF_ = 3.5 Hz, ^3^*J*_CF_ = 8.7 Hz, C), 125.0 (d, ^3^*J*_CP_ = 17.2 Hz, HC), 126.9 (d, ^1^*J*_CP_ = 189.8 Hz, C), 130.5 (d, ^3^*J*_CF_ = 24.0 Hz, HC), 130.7 (HC), 131.2 (d, ^3^*J*_CF_ = 8.3 Hz, 2 HC), 131.4 (d, ^3^*J*_CP_ = 11.8 Hz, HC), 133.2 (C), 136.0 (C), 149.5 (C), 150.1 (d, ^4^*J*_CP_ = 3.0 Hz, C), 150.7 (dd, ^2^*J*_CF_ = 12.7 Hz, ^1^*J*_CF_ = 248.6 Hz, C), 151.8 (dd, ^2^*J*_CF_ = 12.4 Hz, ^1^*J*_CF_ = 252.0 Hz, C), 156.3 (C), 163.2 (d, ^1^*J*_CF_ = 249.3 Hz, C-F) ppm; ^31^P {^1^H} NMR (120 MHz, CDCl_3_) *δ*: 19.0 ppm; ^19^F NMR (282 MHz, CDCl_3_) *δ*: −112.6, −136.1 and −137.1 (m) ppm. HRMS (EI): calculated for C_25_H_21_F_3_NO_3_P [M]^+^ 471.1211; found 471.1224. Purity 99.96% (EtOH/Heptane = 10/90, Rt = 7.363 min).

*Diethyl (2-(4-fluorophenyl)-4-(p-tolyl)quinolin-6-yl)phosphonate* (**7v**). The general procedure was followed using aminophenylphosphonate **1c** (1 mmol, 0.23 g), 4-fluorobenzaldehyde (1 mmol, 0.11 mL), 4-methylstyrene (1.5 mmol, 0.20 mL) and BF_3·_Et_2_O affording 0.44 g (98%) of a white solid identified as **7v**, mp 153–155 °C (ethyl acetate/hexane). ^1^H NMR (300 MHz, CDCl_3_) *δ*: 1.21–1.25 (m, 6 H, 2 CH_3_), 2.40 (s, CH_3_), 3.96–4.12 (m, 4 H, 2 CH_2_), 7.11–7.16 (m, 2 H), 7.28–7.30 (m, 2 H), 7.35–7.37 (m, 2 H), 7.75 (s, 1 H), 7.90–7.95 (m, 1 H), 8.11–8.15 (m, 2 H), 8.16–8.19 (m, 1 H), 8.40–8.44 (m, 1 H) ppm; ^13^C {^1^H} NMR (75 MHz, CDCl_3_) *δ*: 16.3 (d, ^3^*J*_CP_ = 6.5 Hz, 2 CH_3_), 21.3 (CH_3_), 62.2 (d, ^2^*J*_CP_ = 5.4 Hz, 2 CH_2_), 115.8 (d, ^2^*J*_CF_ = 21.6 Hz, 2 HC), 119.71 (HC), 125.0 (d, ^3^*J*_CP_ = 17.3 Hz, C), 126.1 (d, ^1^*J*_CP_ = 189.3 Hz, C), 129.3 (2 HC), 129.6 (3 HC), 129.7 (d, ^3^*J*_CF_ = 8.5 Hz, 2 HC), 130.4 (d, ^2^*J*_CP_ = 12.8 Hz, HC), 131.9 (d, ^2^*J*_CP_ = 12.0 Hz, HC), 134.5 (C), 135.2 (C), 138.9 (C), 150.2 (C), 150.4 (C), 157.7 (C), 164.0 (d, ^1^*J*_CF_ = 249.9 Hz, C-F) ppm; ^31^P {^1^H} NMR (120 MHz, CDCl_3_) *δ*: 19.4 ppm; ^19^F NMR (282 MHz, CDCl_3_) *δ*: −111.9 ppm. HRMS (EI): calculated for C_26_H_25_FNO_3_P [M]^+^ 449.1556; found 449.1573. Purity 99.23% (EtOH/Heptane = 10/90, Rt = 5.138 min).

*Diethyl (2-(3,4-difluorophenyl)-4-(p-tolyl)quinolin-6-yl)phosphonate* (**7x**). The general procedure was followed using aminophenylphosphonate **1c** (1 mmol, 0.23 g), 3,4-difluorobenzaldehyde (1 mmol, 0.11 mL), 4-methylstyrene (1.5 mmol, 0.20 mL) and BF_3·_Et_2_O affording 0.46 g (98%) of a white solid identified as **7x**, mp 158–160 °C (ethyl acetate/hexane). ^1^H NMR (300 MHz, CDCl_3_) *δ*: 1.21–1.25 (m, 6 H, 2 CH_3_), 2.40 (s, CH_3_), 3.96–4.13 (m, 4 H, 2 CH_2_), 7.19–7.25 (m, 1 H), 7.28–7.30 (m, 2 H), 7.34–7.36 (m, 2 H), 7.72 (s, 1 H), 7.83–7.87 (m, 1 H), 7.91–7.96 (m, 1 H), 8.01–8.07 (m, 1 H), 8.16–8.19 (m, 1 H), 8.40–8.44 (m, 1 H) ppm; ^13^C {^1^H} NMR (75 MHz, CDCl_3_) *δ*: 16.3 (d, ^3^*J*_CP_ = 6.5 Hz, 2 CH_3_), 21.3 (CH_3_), 62.3 (d, ^2^*J*_CP_ = 5.4 Hz, 2 CH_2_), 116.8 (d, ^2^*J*_CF_ = 19.3 Hz, HC), 117.6 (d, ^2^*J*_CF_ = 17.6 Hz, HC), 119.4 (HC), 123.7 (dd, ^4^*J*_CF_ = 3.5 Hz, ^3^*J*_CF_ = 6.6 Hz, HC), 125.2 (d, ^3^*J*_CP_ = 17.4 Hz, C), 126.5 (d, ^1^*J*_CP_ = 189.4 Hz, C), 129.3 (2 HC), 129.6 (2 HC), 130.5 (d, ^2^*J*_CP_ = 11.7 Hz, HC), 130.6 (d, ^3^*J*_CP_ = 7.4 Hz, HC), 131.9 (d, ^2^*J*_CP_ = 11.9 Hz, HC), 134.3 (C), 136.2 (dd, ^4^*J*_CF_ = 3.6 Hz, ^3^*J*_CF_ = 6.7 Hz, C), 139.1 (C), 150.1 (d, ^4^*J*_CP_ = 3.0 Hz, C), 150.7 (dd, ^2^*J*_CF_ = 13.8 Hz, ^1^*J*_CF_ = 248.1 Hz, C), 150.7 (C), 151.6 (dd, ^2^*J*_CF_ = 12.7 Hz, ^1^*J*_CF_ = 250.4 Hz, C), 156.3 (C) ppm; ^31^P {^1^H} NMR (120 MHz, CDCl_3_) *δ*: 19.2 ppm; ^19^F NMR (282 MHz, CDCl_3_) *δ*: −136.4 and −137.2 ppm. HRMS (EI): calculated for C_26_H_24_F_2_NO_3_P [M]^+^ 467.1462; found 467.1472. Purity 99.47% (EtOH/Heptane = 10/90, Rt = 6.241 min).

### 3.2. Biology

#### 3.2.1. Materials

Reagents and solvents were used as purchased without further purification. CPT was purchased from Sigma-Aldrich. All stock solutions of the investigated compounds were prepared by dissolving the powdered materials in an appropriate amount of DMSO. The final concentration of DMSO never exceeded 5% (*v*/*v*) in reactions. Under these conditions, DMSO was also used in the controls and was not seen to affect hTOP1B activity. The stock solution was stored at 5 °C until it was used.

#### 3.2.2. Expression and Purification of Human Topoisomerase 1B

The yeast *Saccharomyces cerevisiae* TopI null strain RS190, which was used for expression of recombinant human Top 1 (hTOP1B) was a kind gift from R. Sternglanz (the State University of New York, Stony Brook, NY). Plasmid pHT143, for the expression of recombinant hTOP1B under the control of an inducible GAL promoter, was described [33]. The plasmids pHT143 were transformed into the yeast *S. cerevisiae* strain RS190. The proteins were expressed and purified by affinity chromatography essentially as described [34]. The protein concentrations were estimated from Coomassie blue-stained SDS/polyacrylamide gels by comparison to serial dilutions of bovine serum albumin (BSA).

#### 3.2.3. Cytotoxicity Assays

Cells were cultured according to the supplier’s instructions, A549 (ATCC^®^ CCL-185), SKOV3 (ATCC^®^ HTB-77), HEK293 (CLS GmbH), MRC-5 (ATCC^®^ CCL-171™). Cells were seeded in 96-well plates at a density of 2–2.5 × 10^3^ cells per well and incubated overnight in 0.1 mL of media supplied with 10% Fetal Bovine Serum (Lonza, Basel, Switzerland) in a 5% CO_2_ incubator at 37 °C. On day 2, drugs (concentration between 0.5–50 μM) were added and samples were incubated for 48 h. After treatment, 10 μL of cell counting kit-8 was added to each well for an additional 2 h incubation at 37 °C. The absorbance of each well was determined by an Automatic Elisa Reader System at 450 nm wavelength. CPT was purchased from Sigma-Aldrich and used as a positive control.

#### 3.2.4. hTOP1B DNA Relaxation Assays 

TOP1B activity was assayed using a DNA relaxation assay by incubating 110 ng/μL of hTOP1B with 0.5 μg of negatively supercoiled pUC18 in 20 μL of reaction buffer (20 mM Tris–HCl, 0.1 mM Na_2_EDTA, 10 mM MgCl_2_, 50 μg/mL acetylated BSA and 150 mM KCl, pH 7.5). The effect of the synthesized tetracyclic **7** derivatives on topoisomerase activity was measured by adding the compounds, at different time points as indicated in the text. The reactions were performed at 37 °C, stopped by the addition of 0.5% SDS after indicated time intervals. The samples were protease digested, electrophoresed in a horizontal 1% agarose gel in 1× TBE (50 mM Tris, 45 mM boric acid, 1 mM EDTA) at 26V for 20 h. The gel was stained with gel red (Biotium, CA, USA, 5 μg/mL), destained with water and photographed under UV illumination.

Since all drugs were dissolved in dimethyl sulfoxide (DMSO), a positive control sample containing the same DMSO concentration as the samples incubated with the drugs was included in all experiments. As a control for drug inhibition, the well-known hTOP1B specific drug CPT was included.

#### 3.2.5. Docking Study 

First, we proceeded with the choice of the most suitable hTOP1B/DNA complex for the docking in the Protein Data Bank (PDB). The Xray structure code 1T8I [27] (3.00 Å resolution) was chosen, a TOP1B of human origin covalently bounded to DNA and containing the anti-cancer agent CPT as a ligand. Maestro [35] graphic interface was used, and the Glide 6.9 application [36] in XP mode (extra precision) [37] was chosen for the docking. The grid was set up in a box of 20 × 20 × 20 Å, centered in the geometric center of CPT. The DNA-binding region in the active site was selected as the target for the screening. The hTOP1B/DNA complex was prepared by reconstructing the phosphoester bond to nucleobase C12 in the 1T8I structure, and the 5′-SH of nucleobase G11 of the cleaved strand was converted to a 5′-OH by changing the sulfur atom by oxygen. The hydrogen atoms were added. The binding orders and the protonation states of waste and DNA were corrected. The complex was optimized and minimized using the Protein Preparation Wizard panel of Schrödinger Suites 2015.1 [38]. Likewise, the structure of the ligand to interact with the protein and the ligand initially present in the complex, CPT, were prepared as previously indicated and used for the different docking processes.

#### 3.2.6. In Vitro *L. infantum* Promastigotes Assays 

A genetically modified strain of *L infantum* BCN 150, expressing iRFP protein from *Rhodopseudomonas palustris* bacteriophytochrome (*iRFP-L. infantum*), was used to obtain the EC_50_ values of the different compounds against promastigotes, through the detection of the near-infrared radiation emitted by living parasites exposed to tested molecules [39]. Thus, the viability of promastigotes was calculated after recording their fluorescence at 708 nm in an Odyssey (Li-Cor, NE, USA) infrared imaging.

*iRFP-L. infantum* promastigotes were cultured using M199 medium (Gibco, Thermo Fisher Scientific, MA, USA), supplemented with 25 mM 4-(2-hydroxyethyl)-1-piperazineethanesulfonic acid (HEPES) pH 6.9, 7.6 mM hemin, 10 mM glutamine, 0.1 mM adenosine, 0.01 mM folic acid, 1× RPMI 1640 vitamin mix (Sigma, Darmstadt, Germany), 10% (*v*/*v*) heat-inactivated foetal bovine serum (Gibco, Thermo Fisher Scientific, MA, USA), 50 U/mL penicillin and 50 µg/mL streptomycin. The assay was carried out, seeding 1 × 10^6^ cells/mL of iRFP*-L. infantum* promastigotes into 96-well optical bottom black plates (Thermo Scientific, MA, USA) along with different concentrations of compound to each well. The final concentration of DMSO, the solvent used to prepare the stock solutions of compounds, was never higher than 0.1%. All of the compounds’ concentrations and controls were assayed by triplicate.

Plots of the infrared fluorescence emitted by viable promastigotes against the different concentrations of tested compounds after 96 h of exposure (fitted by nonlinear analysis using the Sigma Plot 10.1 statistical package), were used to calculate the EC_50_ values.

#### 3.2.7. Ex Vivo Murine Splenic Explant Cultures

All protocols described in this work comply with European Union Legislation (2010/63/UE) and Spanish Act (RD 53/2013).

The EC_50_ values of compounds against the amastigote forms were calculated using a cell suspension obtained from spleens extracted from BALB/c mice. Mice had been infected previously with *iRFP L. infantum* metacyclic promastigotes and sacrificed five weeks after infection. To obtain the cell suspension containing amastigotes, spleens washed with cold phosphate-buffered saline (PBS), were cut into pieces, and incubated with 5 mL of 2 mg/mL collagenase D (Sigma, Darmstadt, Germany), which was prepared in buffer (10 mM HEPES, pH 7.4, 150 mM NaCl, 5 mM KCl, 1 mM MgCl_2_ and 1.8 mM CaCl_2_) for 20 min. Subsequently, these cells were passed through a 100 µm cell strainer, collected by centrifugation (500× *g* for 7 min at 4 °C), washed twice with PBS, and suspended in RPMI medium (Gibco, Thermo Fisher Scientific, MA, USA) supplemented with 10 mM HEPES, 1 mM sodium pyruvate, 1xRPMI 1640 vitamin mix, 10% (*v*/*v*) FBS, 50 U/mL penicillin and 50 µg/mL streptomycin, at 37 °C under a 5% CO_2_ atmosphere. 

To assay the effect of the different compounds against amastigotes, which infect splenic cells, these cells were seeded to confluence into 384-well black optical bottom plates (Thermo Scientific, Waltham, MA, USA) in addition to different concentrations of compound to each well. The viability of amastigotes was calculated by recording their fluorescence at 708 nm in an Odyssey (Li-Cor, NE, USA) infrared imaging system, after 96 h of exposure. Plots of the infrared fluorescence against the concentrations of tested compounds were fitted by nonlinear analysis using the Sigma Plot 10.1 statistical package and used to calculate EC_50_ values. As well as promastigotes viability, the assays were performed in triplicate.

#### 3.2.8. Selectivity Index (SI) Determination

The viability of splenocytes cells against the different compounds was measured using the Alamar Blue staining method (Invitrogen, MA, USA). For this purpose, these cells were obtained following the protocol indicated in the previous section, from uninfected mice. Plots of absorbance of viable cells (obtained following manufacturer’s recommendations of Alamar Blue) vs compounds concentration were used to calculate CC_50_ values. Plots were fitted by nonlinear analysis using the Sigma Plot 10.1 statistical package. The assays were performed by triplicate.

The selectivity index for each compound was calculated as the ratio between the IC_50_ value for splenic cells and the EC_50_ value for amastigotes.

#### 3.2.9. Purification of Leishmanial Topoisomerase 1B 

Purification of leishmanial DNA topoisomerase 1B (LTOP1B) was carried out as reported previously [5]. Shortly, LTOP1B was purified using the deficient in TOP1 activity yeast strain, EKY3 MAT α ura3–52 his3Δ20ting, from the union of the bicistronic expression vector pESC-URA to both subunits of LTOP1B. The yeast cells were grown in a yeast synthetic drop-out medium without uracil (Sigma) supplemented with 2% raffinose (w/v) to OD_600_: 0.8–1 and induced for 10 h with 2% galactose (*w*/*v*). After that, the cells were harvested, washed with cold TEEG buffer (50 mM Tris–HCl pH 7.4, 1 mM EDTA, 1 mM EGTA, 10% glycerol), resuspended in 15 mL of 1× TEEG buffer supplemented with 0.2 M KCl and a protease inhibitors cocktail (Thermo Scientific, Waltham, MA, USA) and lysed. Protein extract obtained was loaded on a 5 mL Heparin Sepharose column (GE Healthcare, MA, USA). LTOP1B enzyme was eluted at 4 °C with a discontinuous gradient of KCl (0.2, 0.4, 0.6 M) in TEEG buffer.

#### 3.2.10. LTOP1B Relaxation Activity Assay

Leishmanial TOP1B activity was tested by the relaxation of supercoiled plasmid DNA assay. Each reaction contained: one unit of purified LTOP1B enzyme, 100 µM of each compound, 0.5 μg of supercoiled pSK DNA, 10 mM Tris-HCl buffer pH 7.5, 5 mM MgCl_2_, 0.1 mM EDTA, 15 µg/mL bovine serum albumin, and 150 mM KCl. The final volume of each reaction was 20 µL. Reaction mixtures were incubated for 1 min at 37 °C and stopped by the addition of a 4 µL loading buffer (5% sarkosyl, 0.12% bromophenol blue, 25% glycerol). The DNA topoisomers were resolved in 1% agarose gels by electrophoresis in 0.1 M Tris borate EDTA buffer (pH 8.0) at 2 V/cm for 16 h and visualized with UV illumination after ethidium bromide (0.5 μg/mL) staining. The percentage of inhibition was calculated by measuring the intensity of the band corresponding to the supercoiled DNA after separating and staining the different DNA topoisomers. This analysis was performed using the GeneTools from Syngene software (Version 4.3.8, MD, USA).

## 4. Conclusions

In conclusion, in this work, the preparation of 23 quinoline derivatives **7** with aromatic and phosphonate substituents at different positions of the quinoline skeleton has been carried out. The synthesis was performed by a [4 + 2] cycloaddition reaction from the corresponding phosphorated anilines, aromatic aldehydes, and olefins, by a two-step or MCR protocol, and subsequent oxidation of the quinoline derivatives with DDQ or directly by a [4 + 2] cycloaddition reaction with acetylenes used as dienophiles. The compounds showed an interesting biological activity, with cytotoxicity against cancer cells (A549, SKOV3, and HEK293) while they presented no cytotoxicity against the non-cancerous cell line (MRC5). When tested against the A549 cancer cell line, the IC_50_ values observed are generally lower than 3 micromolar, with compounds **7j** and **7v** being the most cytotoxic with IC_50_ values in the nanomolar range.

Regarding the behavior against the biological target, TOP1B, we may say that compounds **7b**, **7j**, and **7k** showed an inhibitory effect against human TOP1B similar to that observed for the natural inhibitor, CPT. On the other hand, compounds **7k** and **7o** showed inhibitory effects on LTOP1B. These data could be useful for the design of new molecules with selective inhibitory activity against LTOP1B, although the overall low SI of the tested drugs suggests that additional targets could be involved in their mode of action.

## Figures and Tables

**Table 1:** Antiproliferative activity of compounds **7**.

Entry	Comp.	PO(OR)_2_	R^1^	R^2^	Cytotoxicity IC_50_ (µM) *^a^*			
					Lung A549	Ovarian SKOV3	Kidney HEK293	MRC-5
1	CPT				(1.0 ± 0.06)·10^−3^	(5.5 ± 0.01)·10^−3^	-	(1.7 ± 0.96)·10^−5^
2	**7a**	8-(PO(OEt)_2_)	C_6_H_5_	H	2.26 ± 0.59	9.30 ± 0.76	27.29 ± 2.22	>50
3	**7b**	8-(PO(OEt)_2_)	4-F-C_6_H_4_	H	2.16 ± 0.29	5.29 ± 1.42	43.04 ± 4.65	>50
4	**7c**	8-(PO(OEt)_2_)	4-OMe-C_6_H_4_	H	12.59 ± 1.30	25.04 ± 3.60	38.29 ± 8.90	>50
5	**7d**	8-(PO(OEt)_2_)	2-OMe-C_6_H_4_	H	12.26 ± 4.10	19.38 ± 1.60	>50	>50
6	**7e**	8-(PO(OEt)_2_)	2-naphthyl	H	3.18 ± 0.27	1.33 ± 0.76	48.14 ± 6.39	>50
7	**7f**	8-(PO(OEt)_2_)	1-naphthyl	H	2.66 ± 0.16	4.54 ± 0.52	35.67 ± 3.13	>50
8	**7g**	8-(PO(OEt)_2_)	C_6_H_5_	F	2.27 ± 0.32	9.29 ± 1.79	24.43 ± 3.46	>50
9	**7h**	8-(PO(OEt)_2_)	4-F-C_6_H_4_	F	3.07 ± 0.22	9.79 ± 0.59	15.66 ± 1.74	>50
10	**7i**	8-(PO(OEt)_2_)	3,4-F_2_-C_6_H_3_	F	4.64 ± 0.30	37.59 ± 9.10	>50	>50
11	**7j**	8-(PO(OEt)_2_)	4-F-C_6_H_4_	Me	0.83 ± 0.01	10.24 ± 0.38	>50	>50
12	**7k**	8-(PO(OEt)_2_)	3,4-F_2_-C_6_H_3_	Me	2.72 ± 0.37	10.59 ± 1.13	22.26 ± 1.36	>50
13	**7l**	8-(PO(O*^i^*Pr)_2_)	4-F-C_6_H_4_	H	5.01 ± 2.70	18.73 ± 1.98	10.12 ± 1.26	>50
14	**7m**	8-(PO(O*^i^*Pr)_2_)	3,4-F_2_-C_6_H_3_	H	18.39 ± 1.4	12.13 ± 1.7	>50	>50
15	**7n**	8-(PO(O*^i^*Pr)_2_)	4-F-C_6_H_4_	F	10.55 ± 1.73	19.82 ± 3.83	15.01 ± 0.99	>50
16	**7o**	8-(PO(O*^i^*Pr)_2_)	3,4-F_2_-C_6_H_3_	F	6.79 ± 3.87	22.12 ± 4.47	21.04 ± 4.05	>50
17	**7p**	8-(PO(O*^i^*Pr)_2_)	4-F-C_6_H_4_	Me	6.84 ± 1.74	12.13 ± 1.68	14.38 ± 1.69	>50
18	**7q**	8-(PO(O*^i^*Pr)_2_)	3,4-F_2_-C_6_H_3_	Me	8.24 ± 2.42	4.67 ± 1.50	12.32 ± 0.71	>50
19	**7r**	6-(PO(OEt)_2_)	4-F-C_6_H_4_	H	2.80 ± 1.24	3.03 ± 1.24	36.86 ± 8.06	>50
20	**7s**	6-(PO(OEt)_2_)	3,4-F_2_-C_6_H_3_	H	6.19 ± 0.67	>50	19.18 ± 1.13	>50
21	**7t**	6-(PO(OEt)_2_)	4-F-C_6_H_4_	F	2.85 ± 1.26	>50	>50	>50
22	**7u**	6-(PO(OEt)_2_)	3,4-F_2_-C_6_H_3_	F	4.33 ± 1.11	>50	>50	>50
23	**7v**	6-(PO(OEt)_2_)	4-F-C_6_H_4_	Me	0.84 ± 0.23	41.61 ± 10.01	>50	>50
24	**7x**	6-(PO(OEt)_2_)	3,4-F_2_-C_6_H_3_	Me	3.05 ± 0.86	>50	>50	>50

*^a^* The cytotoxicity IC_50_ values listed are the concentrations corresponding to 50% growth inhibition.

**Table 2:** Human TOP1B inhibitory activity of compounds **7**.

Entry	Comp.	PO(OR)_2_	R^1^	R^2^	% Inhibition *^a^*		
					15 s	1 min	3 min
1	CPT				80	76	3
2	**7a**	8-(PO(OEt)_2_)	C_6_H_5_	H	20	28	1
3	**7b**	8-(PO(OEt)_2_)	4-F-C_6_H_4_	H	54	61	21
4	**7c**	8-(PO(OEt)_2_)	4-OMe-C_6_H_4_	H	11	9	4
5	**7d**	8-(PO(OEt)_2_)	2-OMe-C_6_H_4_	H	22	16	3
6	**7e**	8-(PO(OEt)_2_)	2-naphthyl	H	17	36	4
7	**7f**	8-(PO(OEt)_2_)	1-naphthyl	H	27	28	8
8	**7g**	8-(PO(OEt)_2_)	C_6_H_5_	F	28	36	3
9	**7h**	8-(PO(OEt)_2_)	4-F-C_6_H_4_	F	27	19	4
10	**7i**	8-(PO(OEt)_2_)	3,4-F_2_-C_6_H_3_	F	47	37	2
11	**7j**	8-(PO(OEt)_2_)	4-F-C_6_H_4_	Me	65	67	3
12	**7k**	8-(PO(OEt)_2_)	3,4-F_2_-C_6_H_3_	Me	63	95	5
13	**7l**	8-(PO(O*^i^*Pr)_2_)	4-F-C_6_H_4_	H	18	13	2
14	**7m**	8-(PO(O*^i^*Pr)_2_)	3,4-F_2_-C_6_H_3_	H	26	32	3
15	**7n**	8-(PO(O*^i^*Pr)_2_)	4-F-C_6_H_4_	F	59	57	7
16	**7o**	8-(PO(O*^i^*Pr)_2_)	3,4-F_2_-C_6_H_3_	F	29	27	3
17	**7p**	8-(PO(O*^i^*Pr)_2_)	4-F-C_6_H_4_	Me	32	46	4
18	**7q**	8-(PO(O*^i^*Pr)_2_)	3,4-F_2_-C_6_H_3_	Me	51	35	6
19	**7r**	6-(PO(OEt)_2_)	4-F-C_6_H_4_	H	9	15	3
20	**7s**	6-(PO(OEt)_2_)	3,4-F_2_-C_6_H_3_	H	9	13	2
21	**7t**	6-(PO(OEt)_2_)	4-F-C_6_H_4_	F	21	18	5
22	**7u**	6-(PO(OEt)_2_)	3,4-F_2_-C_6_H_3_	F	27	21	14
23	**7v**	6-(PO(OEt)_2_)	4-F-C_6_H_4_	Me	22	30	12
24	**7x**	6-(PO(OEt)_2_)	3,4-F_2_-C_6_H_3_	Me	18	25	5

*^a^* The activity of the compounds inhibiting hTOP1B relaxation was expressed quantitatively by comparison with the inhibitory activity observed for CPT.

**Table 3:** Bioactivity of phosphonate derivative compounds **7** on both forms of Leishmania parasites.

Entry	Comp.	PO(OR)_2_	R^1^	R^2^	EC_50_ L. Infantum		IC_50_ Splenocites	SI	LTOP1B Inhibition
					Promastigotes	Amastigotes			
1	**7a**	8-(PO(OEt)_2_)	C_6_H_5_	H	0.91 ± 0.04	4.03 ± 0.30	3.61 ± 0.45	0.9	58.87
2	**7b**	8-(PO(OEt)_2_)	4-F-C_6_H_4_	H	7.63 ± 0.76	14.56 ± 1.30	12.24 ± 1.77	0.8	24.43
3	**7c**	8-(PO(OEt)_2_)	4-OMe-C_6_H_4_	H	9.46 ± 1.67	8.23 ± 1.70	15.90 ± 1.65	1.9	45.45
4	**7d**	8-(PO(OEt)_2_)	2-OMe-C_6_H_4_	H	11.37 ± 0.62	19.46 ± 2.38	30.09 ± 6.05	1.5	46.41
5	**7e**	8-(PO(OEt)_2_)	2-naphthyl	H	20.38 ± 1.57	20.44 ± 5.62	51.62 ± 1.52	2.5	18.36
6	**7f**	8-(PO(OEt)_2_)	1-naphthyl	H	20.55 ± 2.19	19.66 ± 2.39	12.22 ± 1.49	0.6	7.5
7	**7g**	8-(PO(OEt)_2_)	C_6_H_5_	F	16.05 ± 1.94	11.89 ± 5.17	33.86 ± 7.98	2.8	16.49
8	**7h**	8-(PO(OEt)_2_)	4-F-C_6_H_4_	F	8.43 ± 0.92	24.17 ± 2.37	20.26 ± 7.49	0.8	22.44
9	**7i**	8-(PO(OEt)_2_)	3,4-F_2_-C_6_H_3_	F	6.35 ± 0.16	13.43 ± 5.53	20.64 ± 3.11	1.5	54.01
10	**7j**	8-(PO(OEt)_2_)	4-F-C_6_H_4_	Me	7.29 ± 0.94	9.80 ± 0.48	14.54 ± 1.56	1.5	41.21
11	**7k**	8-(PO(OEt)_2_)	3,4-F_2_-C_6_H_3_	Me	2.59 ± 0.48	26.23 ± 3.65	24.07 ± 7.79	0.9	77.02
12	**7l**	8-(PO(O*^i^*Pr)_2_)	4-F-C_6_H_4_	H	9.57 ± 0.4	19.47 ± 2.23	22.1 ± 4.7	1.1	3.55
13	**7m**	8-(PO(O*^i^*Pr)_2_)	3,4-F_2_-C_6_H_3_	H	11.18 ± 0.74	10.20 ± 1.34	19.79 ± 3.76	1.9	5.41
14	**7n**	8-(PO(O*^i^*Pr)_2_)	4-F-C_6_H_4_	F	4.86 ± 0.5	5.52 ± 1.11	15.29 ± 1.72	2.8	43.31
15	**7o**	8-(PO(O*^i^*Pr)_2_)	3,4-F_2_-C_6_H_3_	F	19.26 ± 1.79	19.65 ± 1.73	18.60 ± 1.10	0.9	73.27
16	**7p**	8-(PO(O*^i^*Pr)_2_)	4-F-C_6_H_4_	Me	5.01 ± 0.35	24.32 ± 2.53	21.23 ± 3.5	0.9	41.62
17	**7q**	8-(PO(O*^i^*Pr)_2_)	3,4-F_2_-C_6_H_3_	Me	7.46 ± 0.76	13.91 ± 3.65	28.33 ± 9.16	2	52.65
18	**7r**	6-(PO(OEt)_2_)	4-F-C_6_H_4_	H	15.22 ± 1.14	29.23 ± 14	28.07 ± 1.23	1	1.94
19	**7s**	6-(PO(OEt)_2_)	3,4-F_2_-C_6_H_3_	H	14.72 ± 1.78	14.20± 2.83	26.23 ± 0.51	1.8	14.56
20	**7t**	6-(PO(OEt)_2_)	4-F-C_6_H_4_	F	16.73 ± 0.72	31.46 ± 2.71	44.97 ± 10.24	1.4	37.86
21	**7u**	6-(PO(OEt)_2_)	3,4-F_2_-C_6_H_3_	F	7.07 ± 0.61	26.36 ± 2.2	>100	>3.8	50.24
22	**7v**	6-(PO(OEt)_2_)	4-F-C_6_H_4_	Me	7.02 ± 0.94	17.56 ± 7.81	11.18 ± 4.42	0.6	43.25
23	**7x**	6-(PO(OEt)_2_)	3,4-F_2_-C_6_H_3_	Me	21.29 ± 2.34	32.95 ± 6.55	32.33 ± 3.36	1	43.84

Antileishmanial effects (EC_50_ ± SD) on promastigotes and amastigotes of *L. infantum*. Cytotoxicity effects (IC_50_ ± SD) on murine splenocytes. SI: Selective Index. Inhibition of leishmanial TOP1B enzyme (LTOP1B inhibition ± SD) at 100 µM of every compound.

## Data Availability

Data is contained within the article and Supplementary Materials.

## Supplementary Materials

The following are available online at https://www.mdpi.com/article/10.3390/ph14080784/s1, 1H NMR, 13C NMR, 31P NMR and 19F NMR spectra of compounds and Table S1.

## Abbreviations

AMB	Amphotericin B
CCK8	Cell counting kit
CPT	Camptothecin
DDQ	Dichloro-5,6-dicyanobenzoquinone
MCR	Multicomponent reaction
SDS	Sodium dodecyl sulfate; SI, selectivity index
TOP1B	DNA topoisomerase 1B
TLC	Thin layer chromatography
