# Supplementary material for: Hybrid Quinolinyl Phosphonates as Heterocyclic Carboxylate Isosteres: Synthesis and Biological Evaluation against Topoisomerase 1B (TOP1B)
Source: Pharmaceuticals (Basel). 2021 Aug 9;14(8):784. doi: 10.3390/ph14080784 (PMC8399847; doi:10.3390/ph14080784)

Hybrid Quinolinyl Phosphonates as Heterocyclic Carboxylate Isosteres: Synthesis and Biological Evaluation against Topoisomerase I (TopI)

Asier Selas,[a] *María Fuertes,*[a] *Estela Melcón-Fernández,*[b] *Yolanda Pérez-Pertejo,*[b] *Rosa M. Reguera,*[b] *Rafael Balaña-Fouce,*[b,]* *Birgitta R. Knudsen,*[c] *Francisco Palacios,*[a] *and Concepcion Alonso*[a,]*

[a]Departamento de Química Orgánica I, Facultad de Farmacia and Centro de Investigación Lascaray (Lascaray Research Center). Universidad del País Vasco/Euskal Herriko Unibertsitatea (UPV/EHU). Paseo de la Universidad 7, 01006 Vitoria-Gasteiz, Spain.

[b]Departamento de Ciencias Biomédicas; Universidad de León, Campus de Vegazana s/n; 24071 León (SPAIN), Phone 34 987 291590.

[c]Department of Molecular Biology and Genetics and Interdisciplinary Nanoscience Center (iNANO), Aarhus University, Aarhus 8000, Denmark.

Page	
S2 to S106	Copies of 1H NMR, 13C NMR, 31P NMR and 19F NMR spectra of compounds **1** and **7**.
S107	**Table S1.** Human TopI inhibitory activity of compounds **7**.
S10 to S119	Copies of HPLC chromatograms of compounds **7**.
S120 to S121	Electrophoresis relaxation assays relative to LTOP1B

^{1}H-RMN

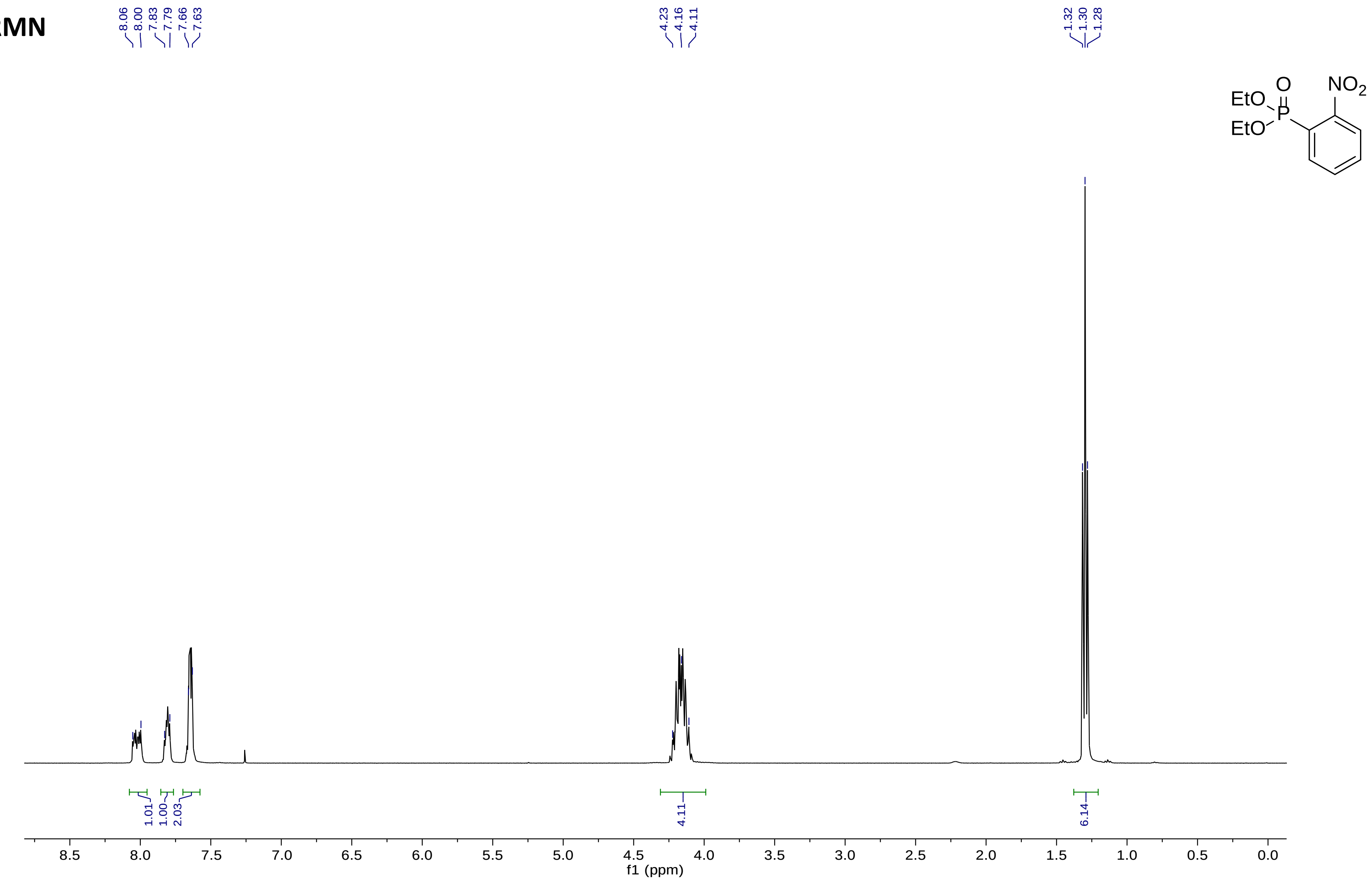

8.06
8.00
7.83
7.79
7.66
7.63
4.23
4.16
4.11
1.32
1.30
1.28
1.01
1.00
2.03
4.11
6.14
8.5 8.0 7.5 7.0 6.5 6.0 5.5 5.0 4.5 4.0 3.5 3.0 2.5 2.0 1.5 1.0 0.5 0.0
f1 (ppm)
EtO
EtO
P
O
NO2

^{13}C-RMN

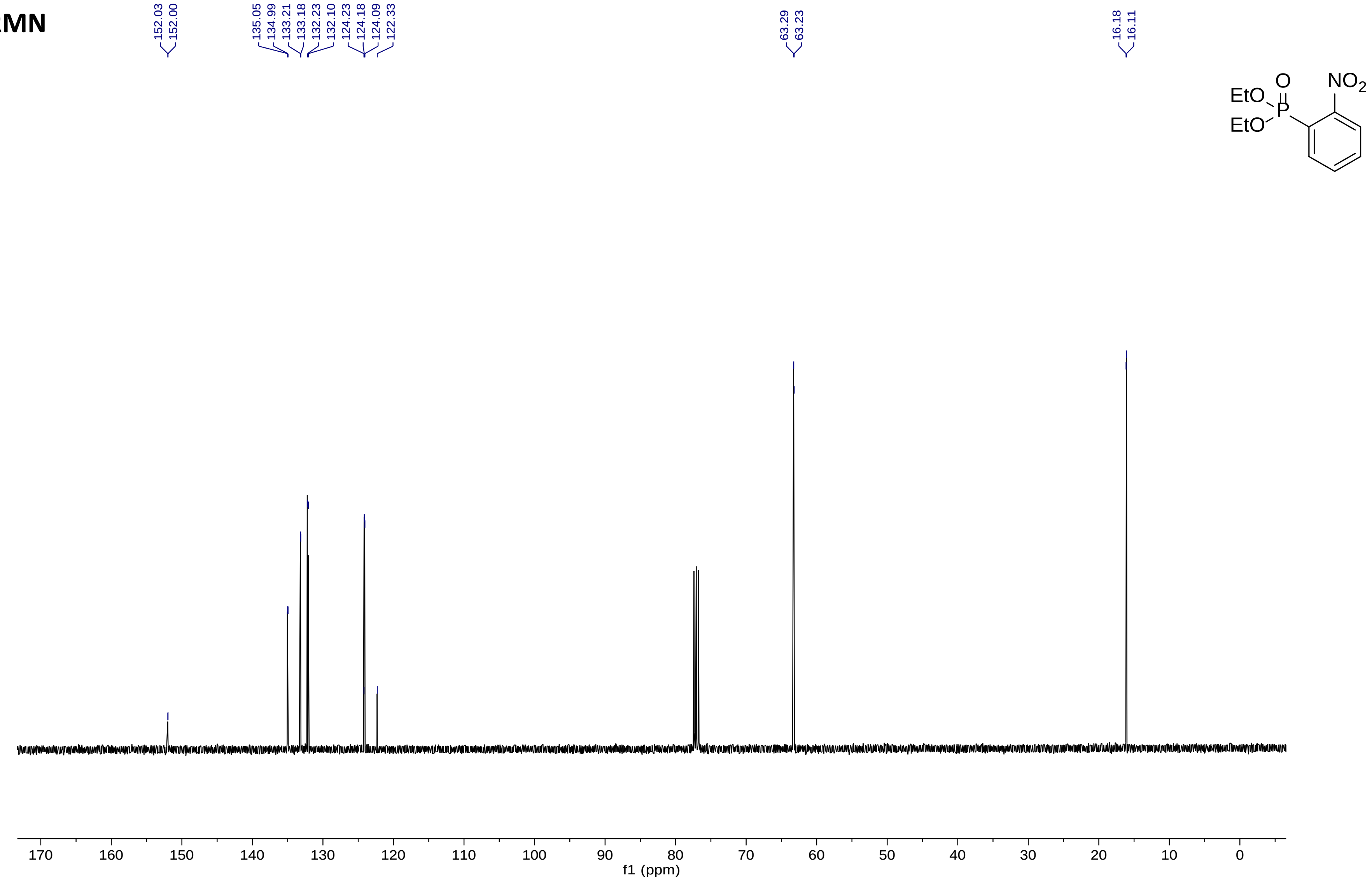
152.03
152.00
135.05
134.99
133.21
133.18
132.23
132.10
124.23
124.18
124.09
122.33
63.29
63.23
16.18
16.11
EtO
EtO
O
P
NO2
170 160 150 140 130 120 110 100 90 80 70 60 50 40 30 20 10 0
f1 (ppm)

^{31}P-RMN

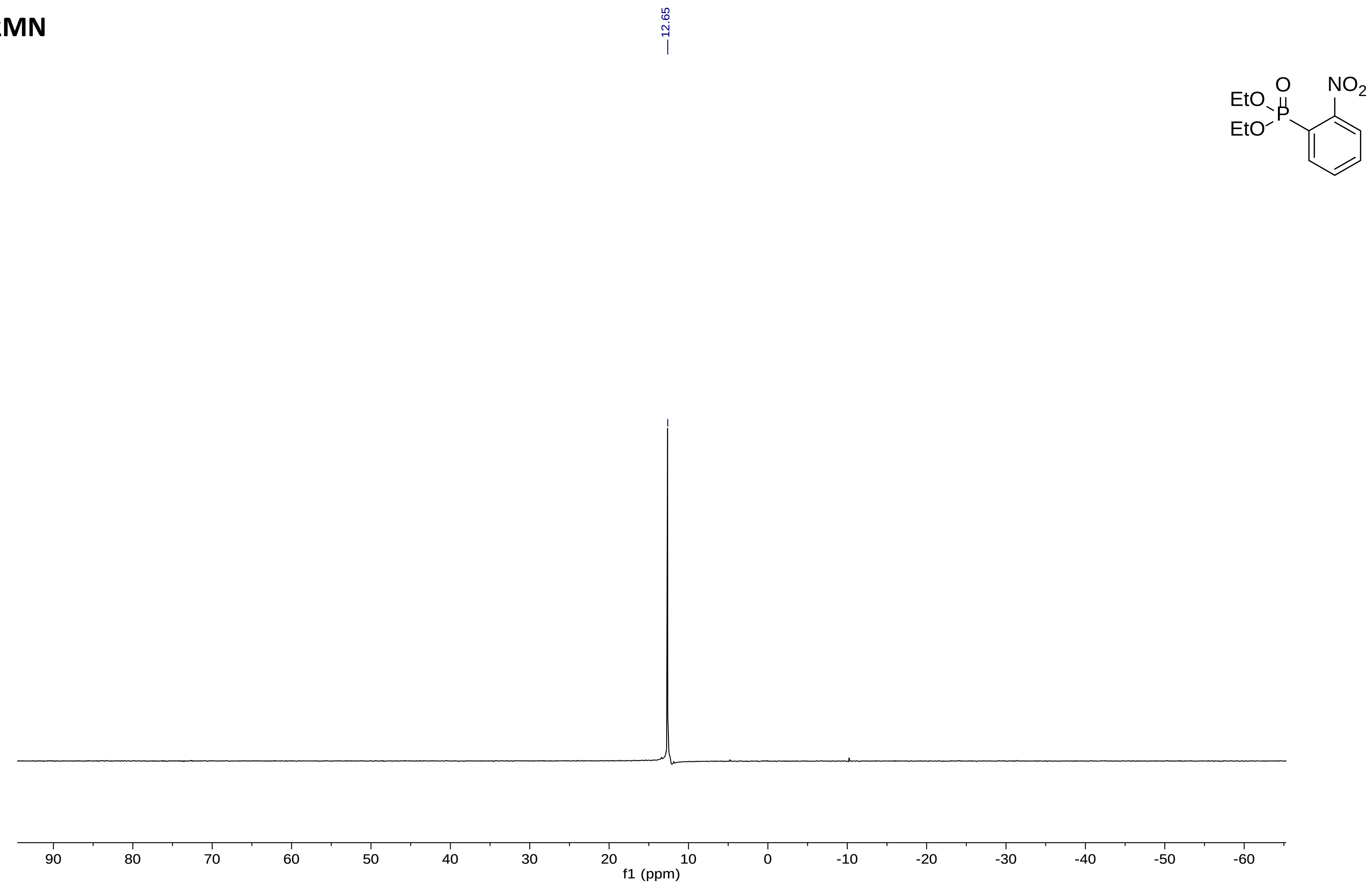

12.65
90
80
70
60
50
40
30
20
10
0
-10
-20
-30
-40
-50
-60
f1 (ppm)
EtO
EtO
P
O
NO2

^{1}H-RMN

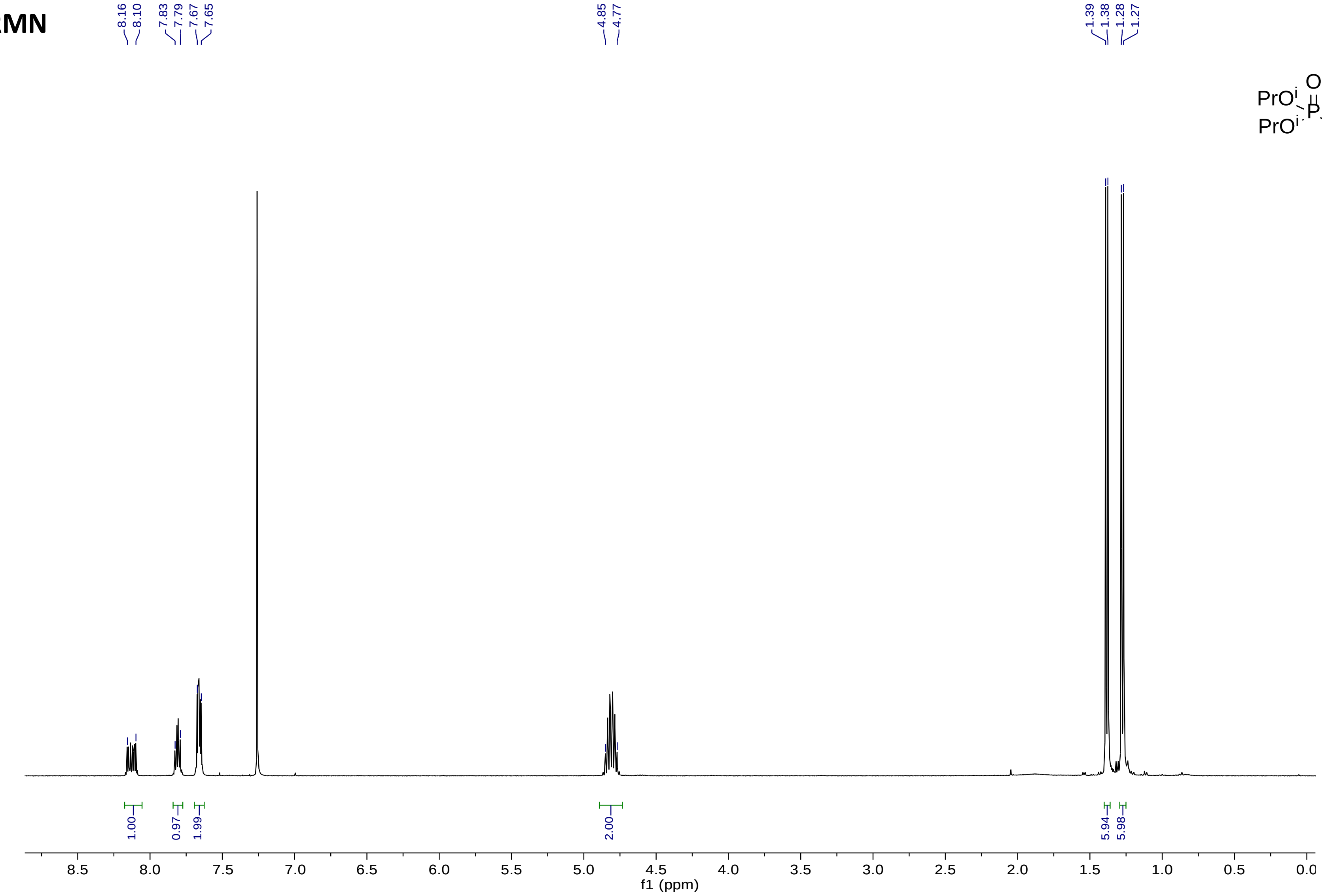

8.16
8.10
7.83
7.79
7.67
7.65
4.85
4.77
1.39
1.38
1.28
1.27
1.00
0.97
1.99
2.00
5.94
5.98
8.5
8.0
7.5
7.0
6.5
6.0
5.5
5.0
4.5
4.0
3.5
3.0
2.5
2.0
1.5
1.0
0.5
0.0
f1 (ppm)

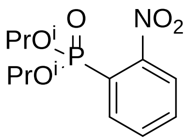

PrO^{i}
PrO^{i}
O
P
NO$_2$

^{13}C-RMN

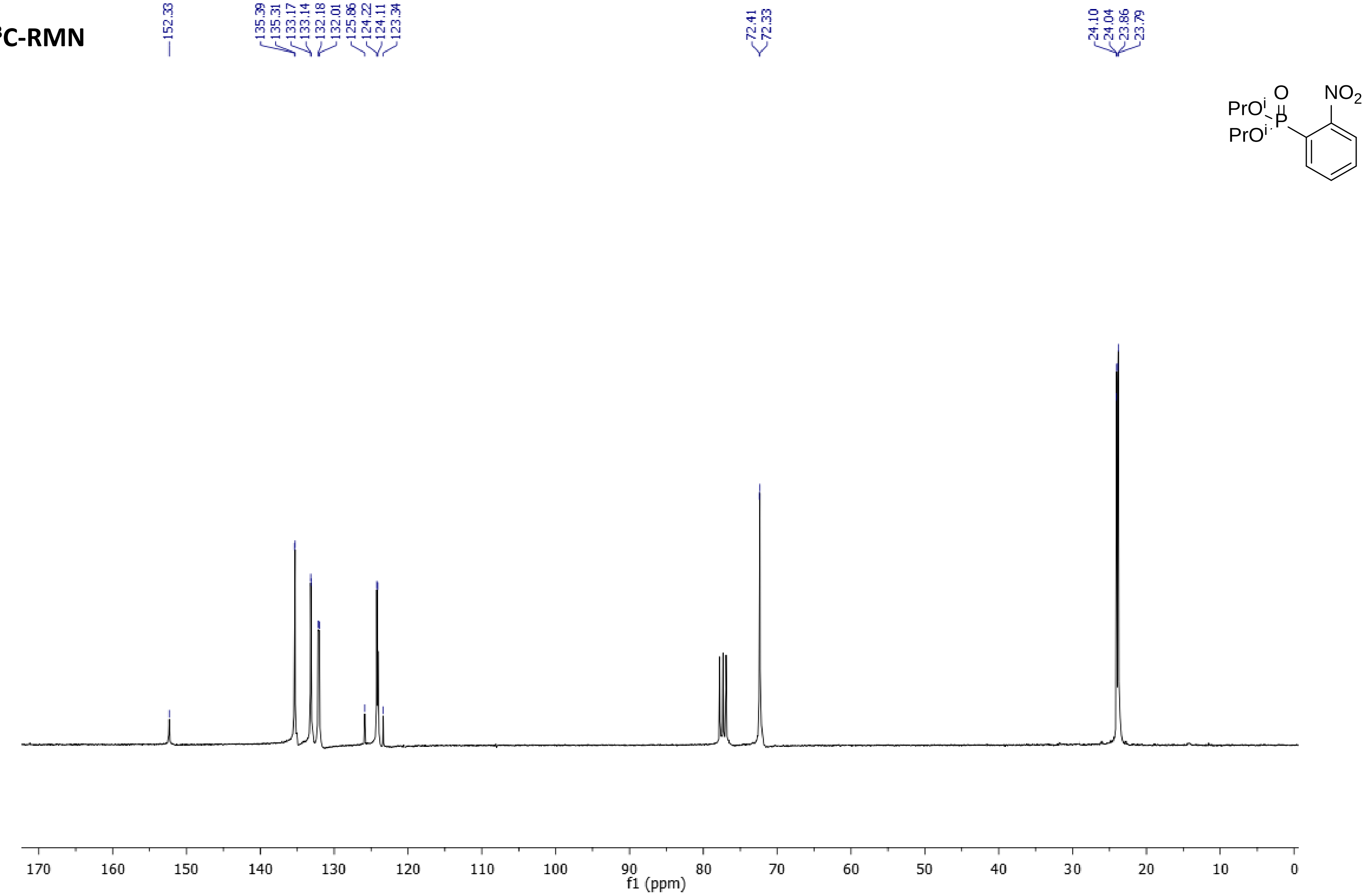

152.33
135.39
135.31
133.17
133.14
132.18
132.01
125.86
124.22
124.11
123.34
72.41
72.33
24.10
24.04
23.86
23.79
170 160 150 140 130 120 110 100 90 80 70 60 50 40 30 20 10 0
f1 (ppm)
PrOi
PrOi
O
P
NO2

^{31}P-RMN

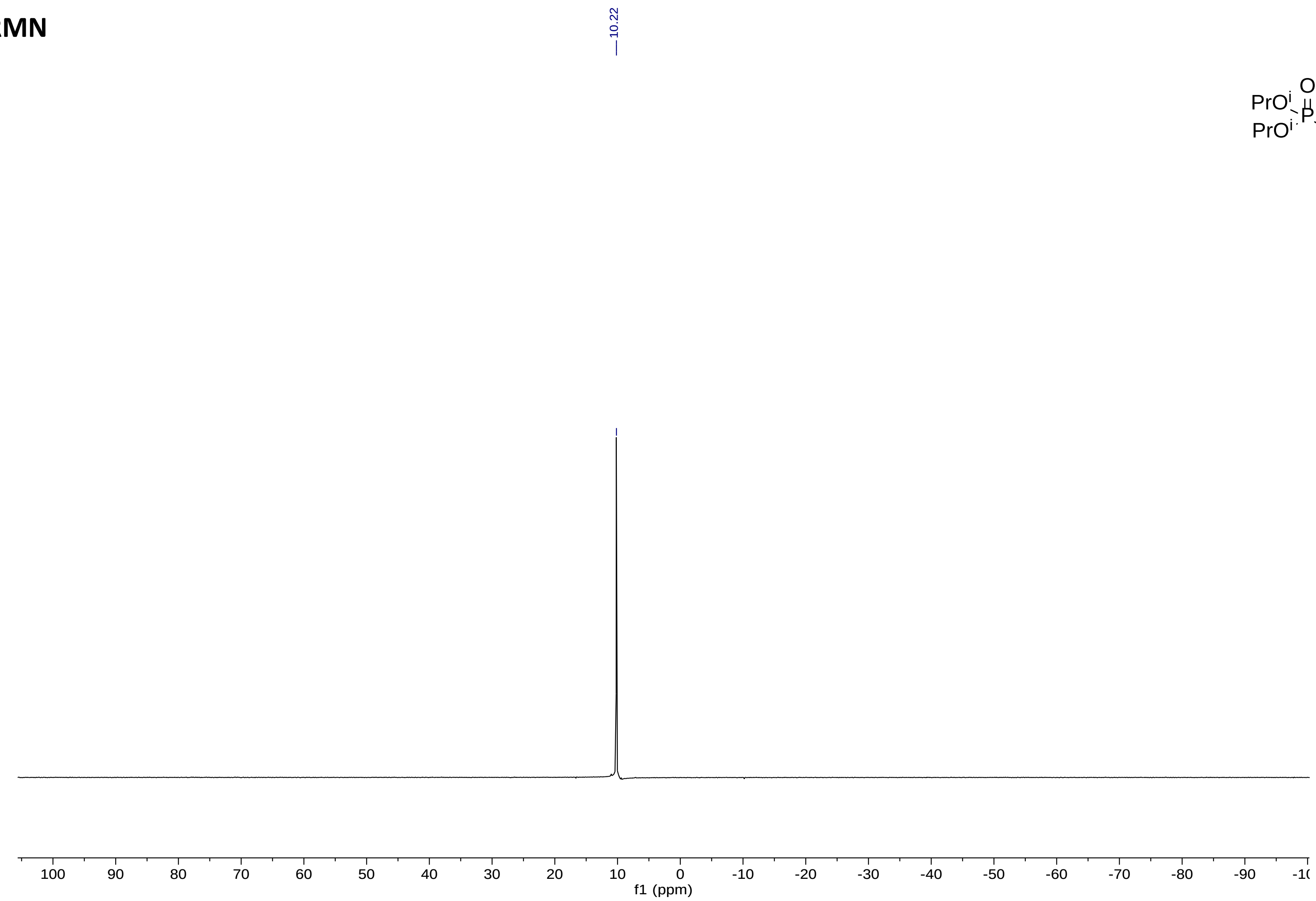
10.22
100 90 80 70 60 50 40 30 20 10 0 -10 -20 -30 -40 -50 -60 -70 -80 -90
f1 (ppm)

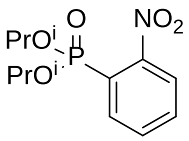
PrO
PrO
O
P
NO2

^{1}H-RMN

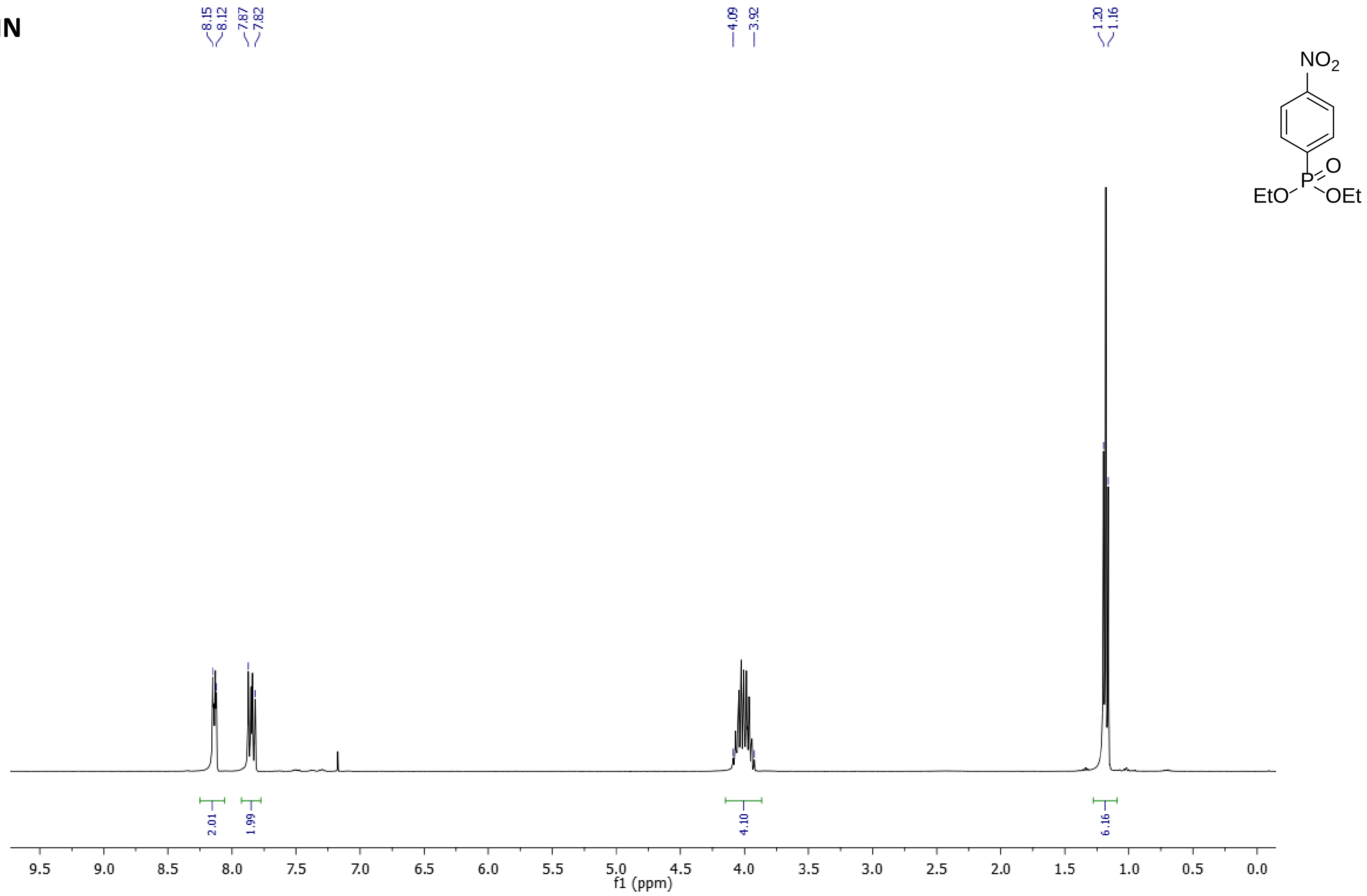

8.15
8.12
7.87
7.82
4.09
3.92
1.20
1.16
NO_2
O
P
EtO
OEt
2.01
1.99
4.10
6.16
9.5
9.0
8.5
8.0
7.5
7.0
6.5
6.0
5.5
5.0
4.5
4.0
3.5
3.0
2.5
2.0
1.5
1.0
0.5
0.0
f1 (ppm)

^{13}C-RMN

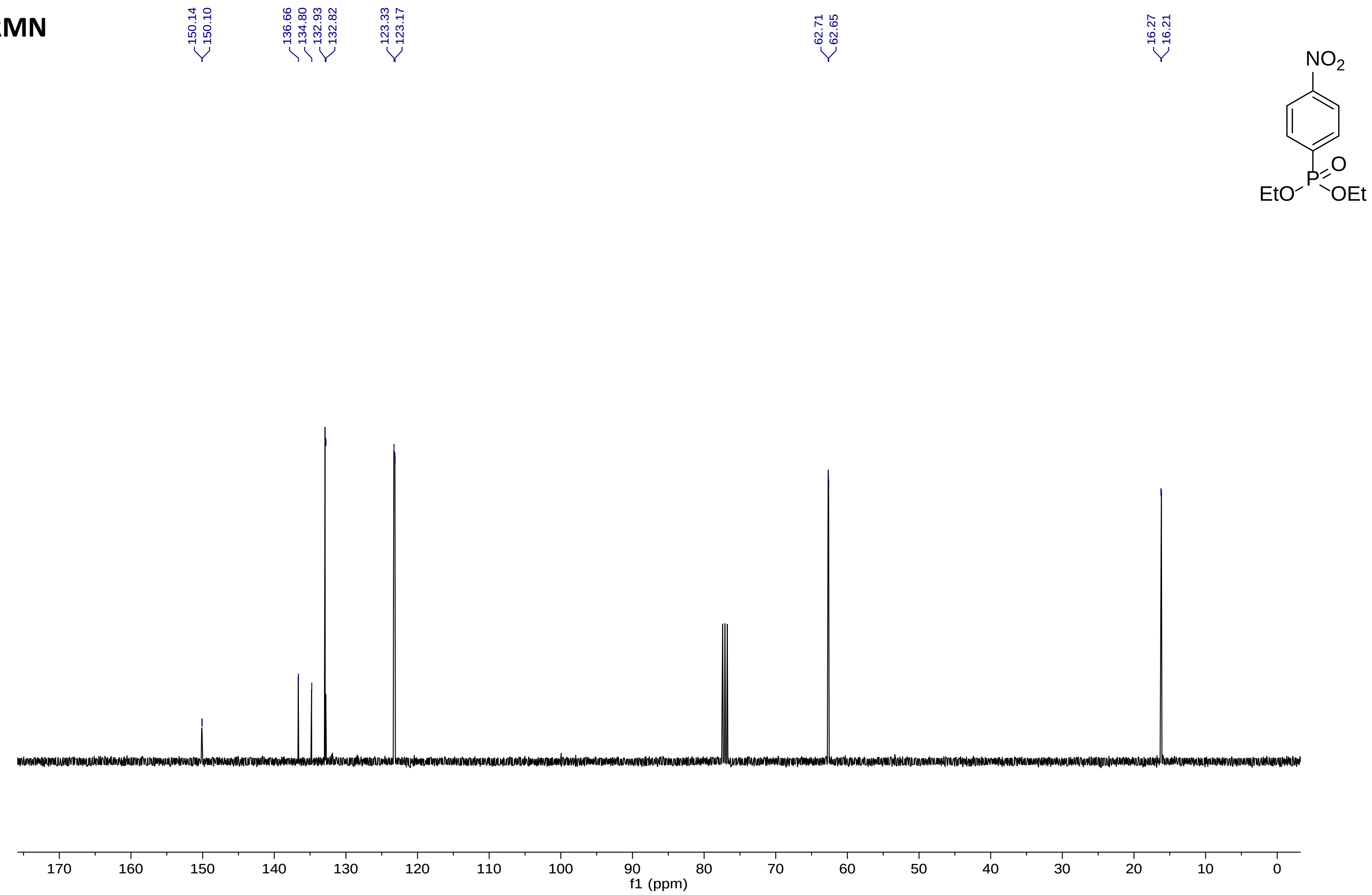

150.14
150.10
136.66
134.80
132.93
132.82
123.33
123.17
62.71
62.65
16.27
16.21
NO2
O
P
EtO
OEt
170 160 150 140 130 120 110 100 90 80 70 60 50 40 30 20 10 0
f1 (ppm)

^{31}P-RMN

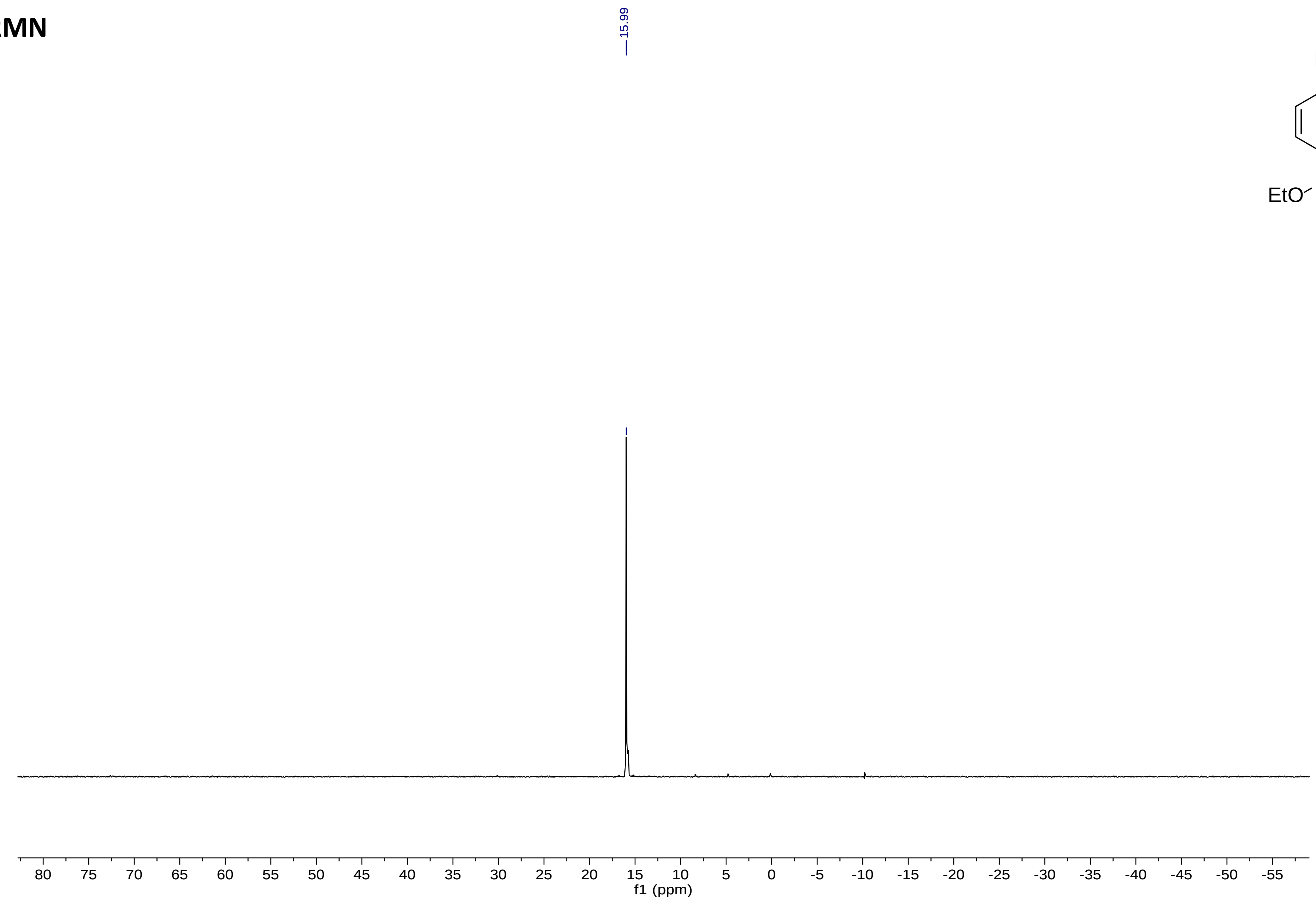

15.99
80
75
70
65
60
55
50
45
40
35
30
25
20
15
10
5
0
-5
-10
-15
-20
-25
-30
-35
-40
-45
-50
-55
f1 (ppm)

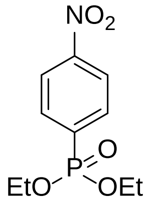

NO2
O
P
EtO
OEt

^{1}H-RMN

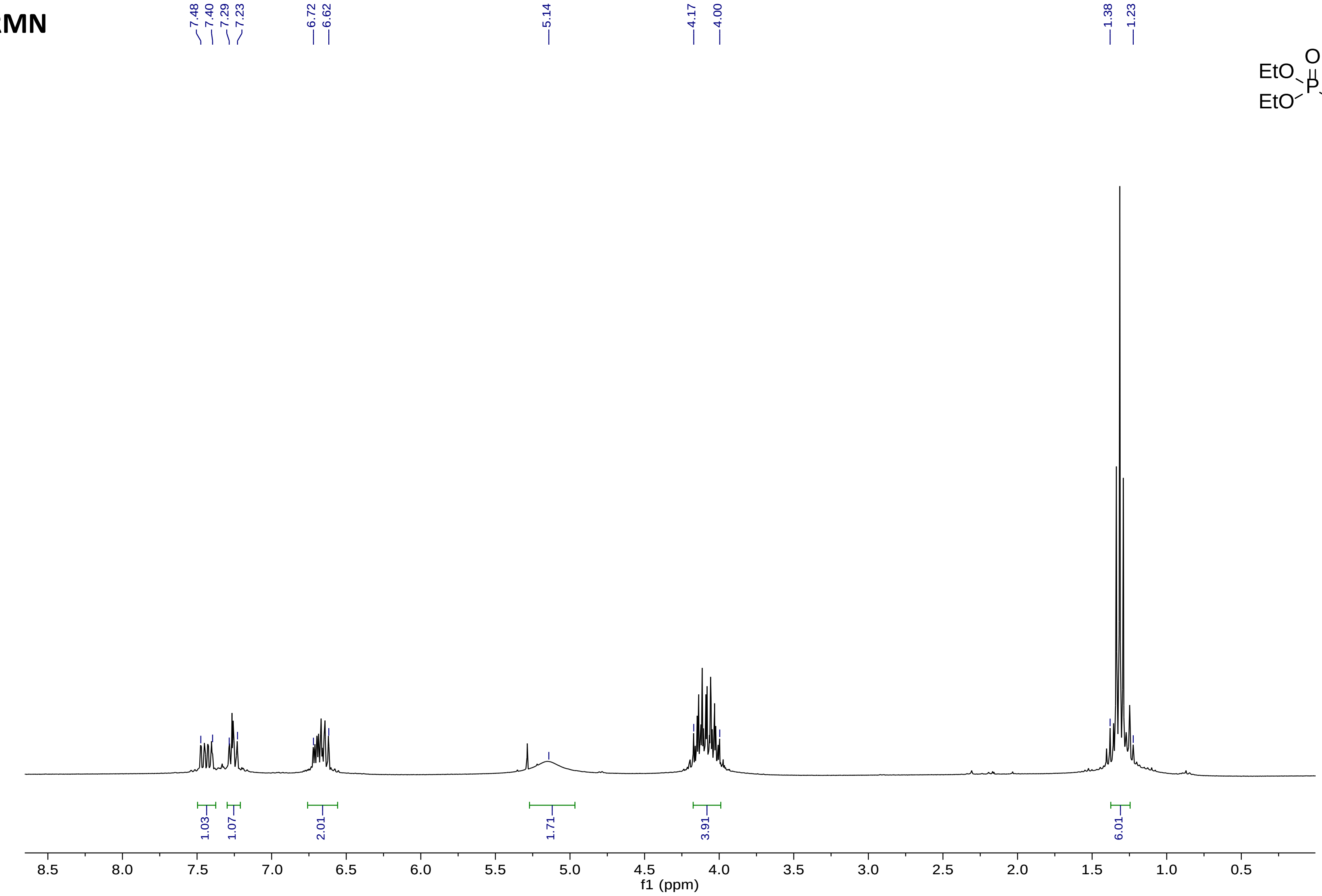
7.48
7.40
7.29
7.23
6.72
6.62
5.14
4.17
4.00
1.38
1.23
1.03
1.07
2.01
1.71
3.91
6.01
8.5
8.0
7.5
7.0
6.5
6.0
5.5
5.0
4.5
4.0
3.5
3.0
2.5
2.0
1.5
1.0
0.5
f1 (ppm)

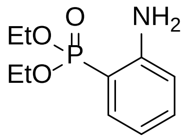
EtO
EtO
P
O
NH2

1a

^{13}C-RMN

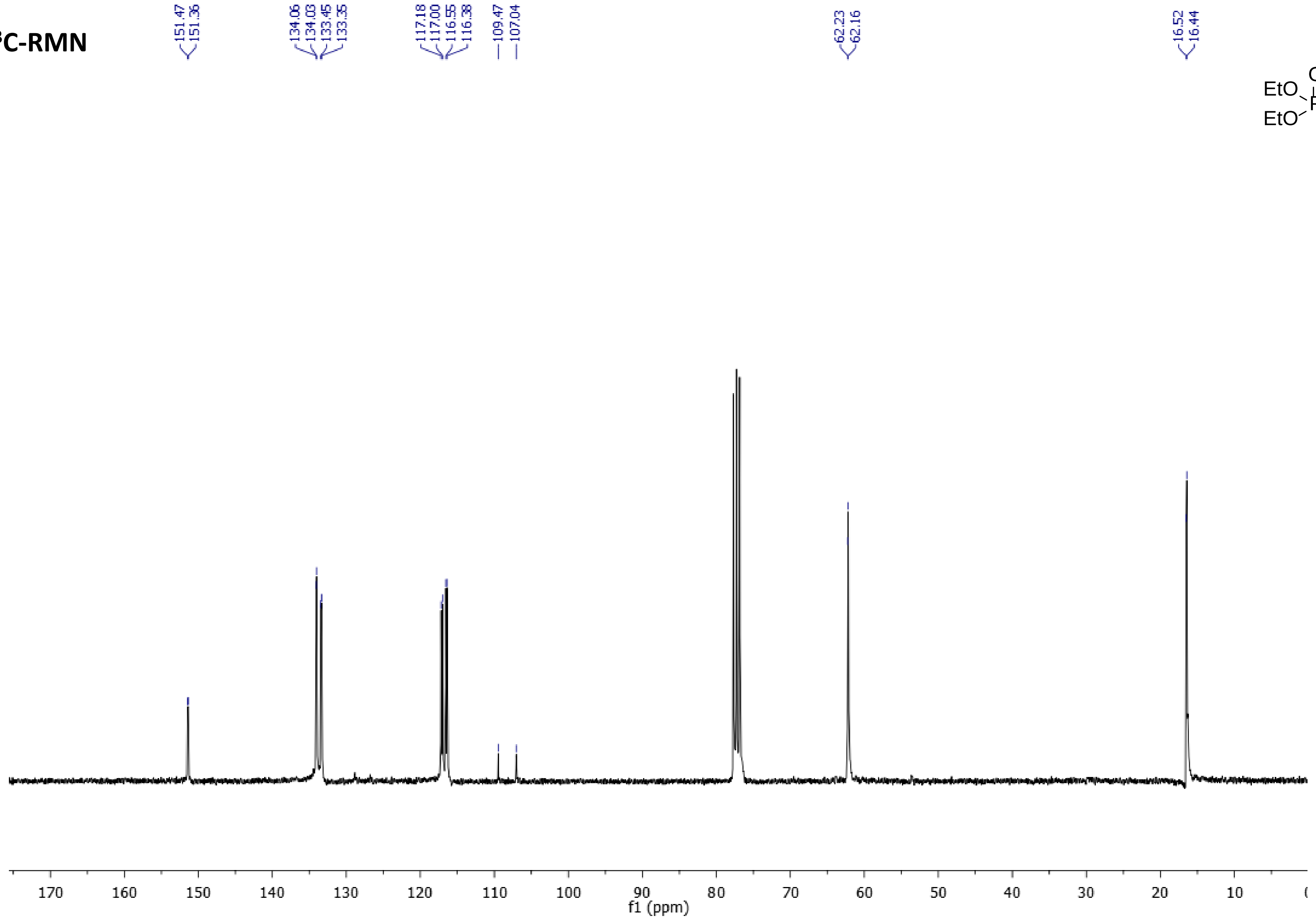
151.47
151.36
134.06
134.03
133.45
133.35
117.18
117.00
116.55
116.38
109.47
107.04
62.23
62.16
16.52
16.44
170
160
150
140
130
120
110
100
90
80
70
60
50
40
30
20
10
f1 (ppm)

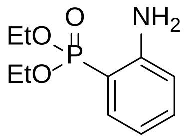
EtO
EtO
O
P
NH2

1a

^{31}P-RMN

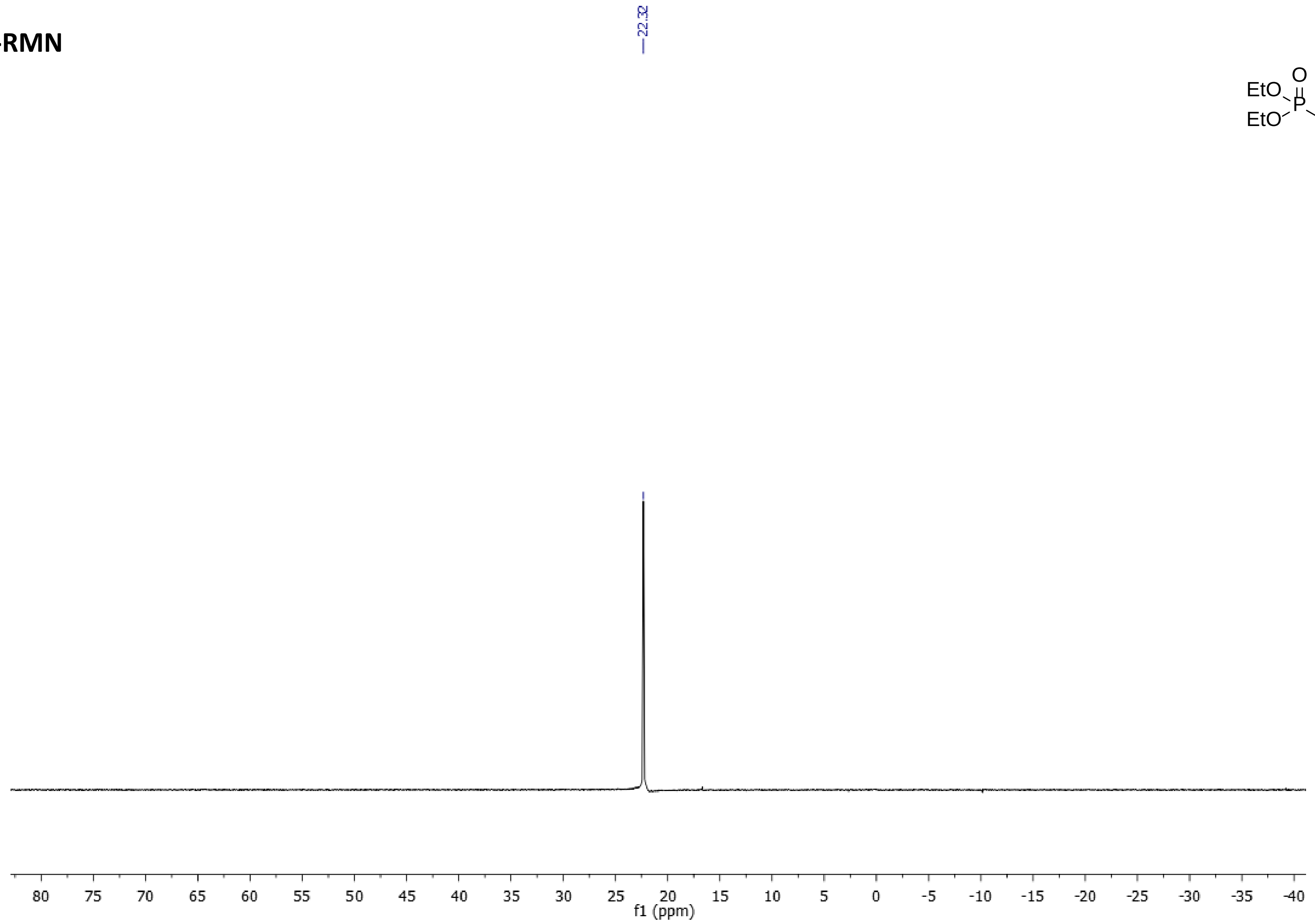

22.32
80 75 70 65 60 55 50 45 40 35 30 25 20 15 10 5 0 -5 -10 -15 -20 -25 -30 -35 -40
f1 (ppm)

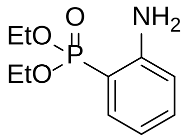

O
NH2
EtO
P
EtO

1a

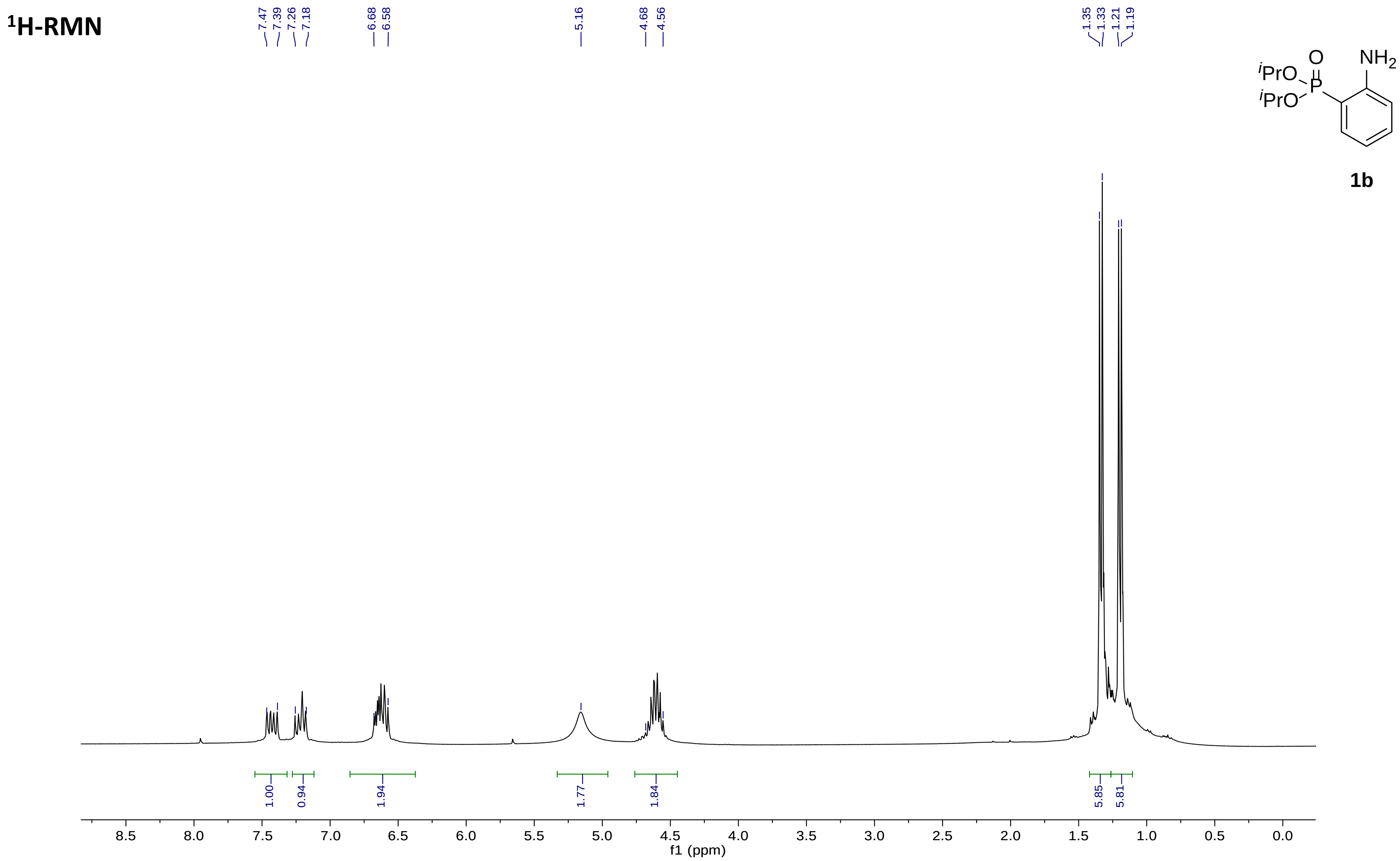

1H-RMN
7.47
7.39
7.26
7.18
6.68
6.58
5.16
4.68
4.56
1.35
1.33
1.21
1.19
iPrO
iPrO
O
P
NH2
1.00
0.94
1.94
1.77
1.84
5.85
5.81
8.5 8.0 7.5 7.0 6.5 6.0 5.5 5.0 4.5 4.0 3.5 3.0 2.5 2.0 1.5 1.0 0.5 0.0
f1 (ppm)

1b

^{13}C-RMN

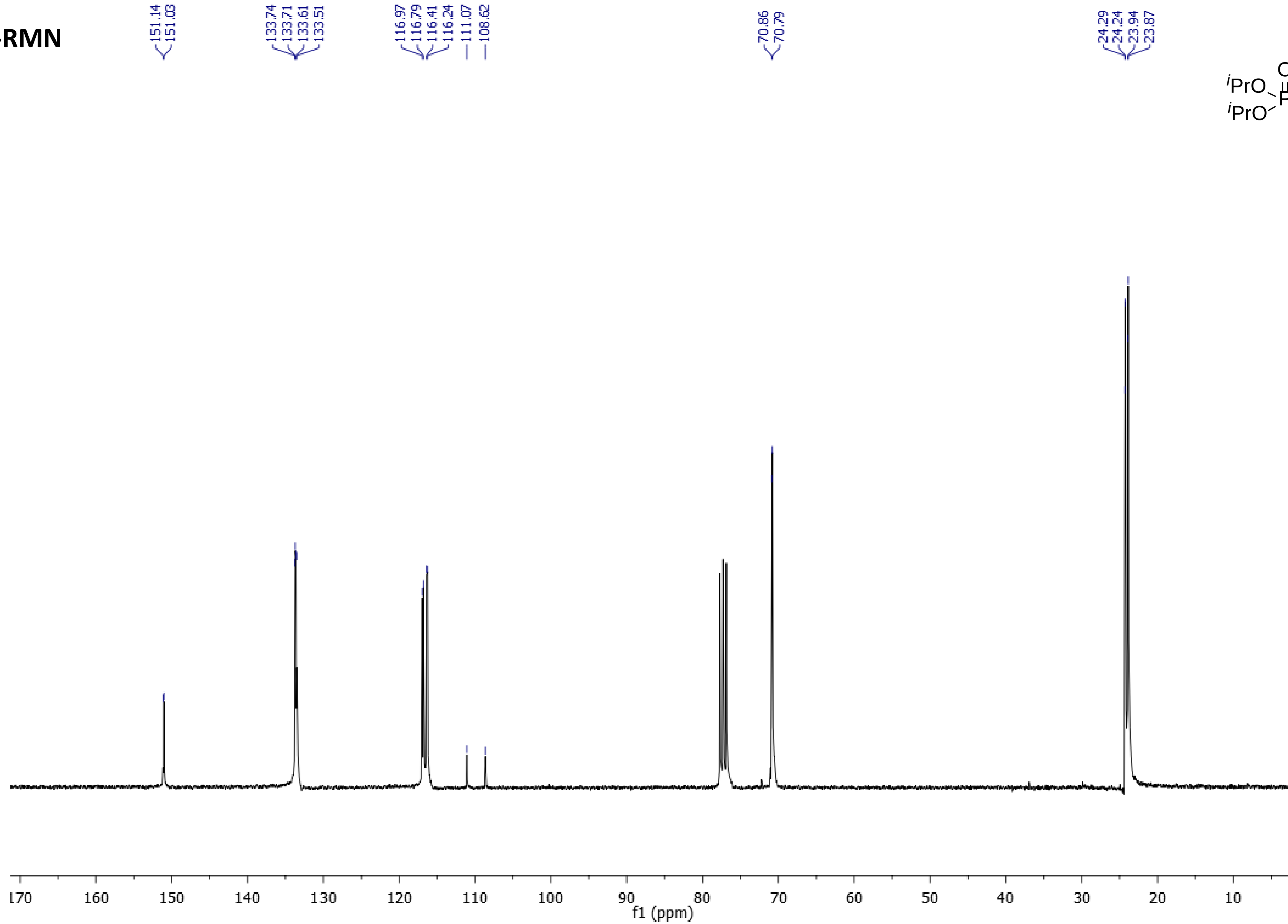

151.14
151.03
133.74
133.71
133.61
133.51
116.97
116.79
116.41
116.24
111.07
108.62
70.86
70.79
24.29
24.24
23.94
23.87
170 160 150 140 130 120 110 100 90 80 70 60 50 40 30 20 10 0
f1 (ppm)

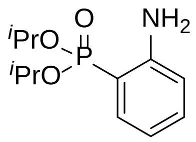

^{i}PrO
^{i}PrO
O
P
NH_2

1b

^{31}P-RMN

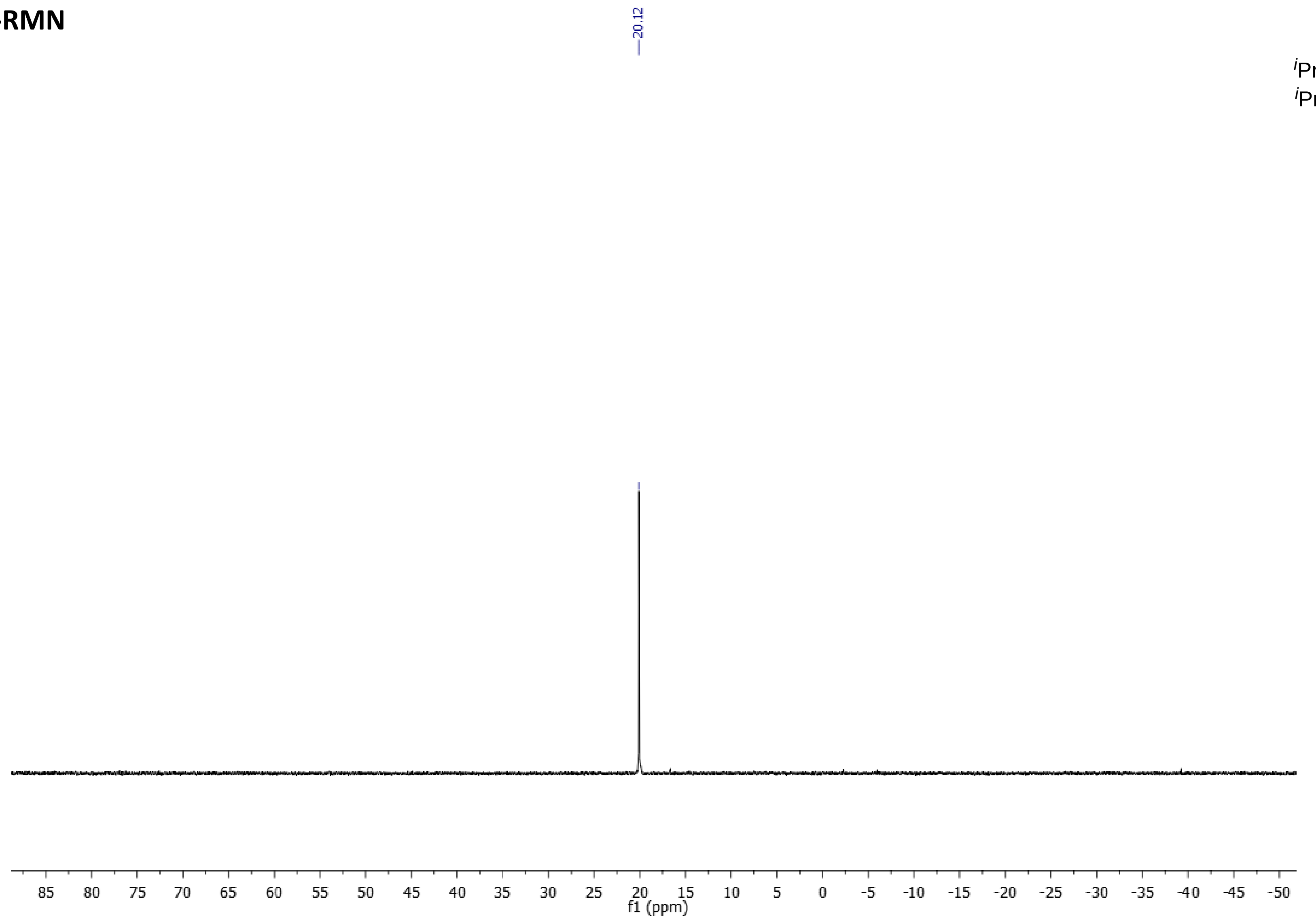
20.12
85
80
75
70
65
60
55
50
45
40
35
30
25
20
15
10
5
0
-5
-10
-15
-20
-25
-30
-35
-40
-45
-50
f1 (ppm)

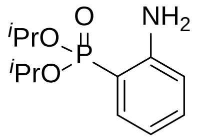
O
NH2
iPrO
iPrO
P

1b

^{1}H-RMN

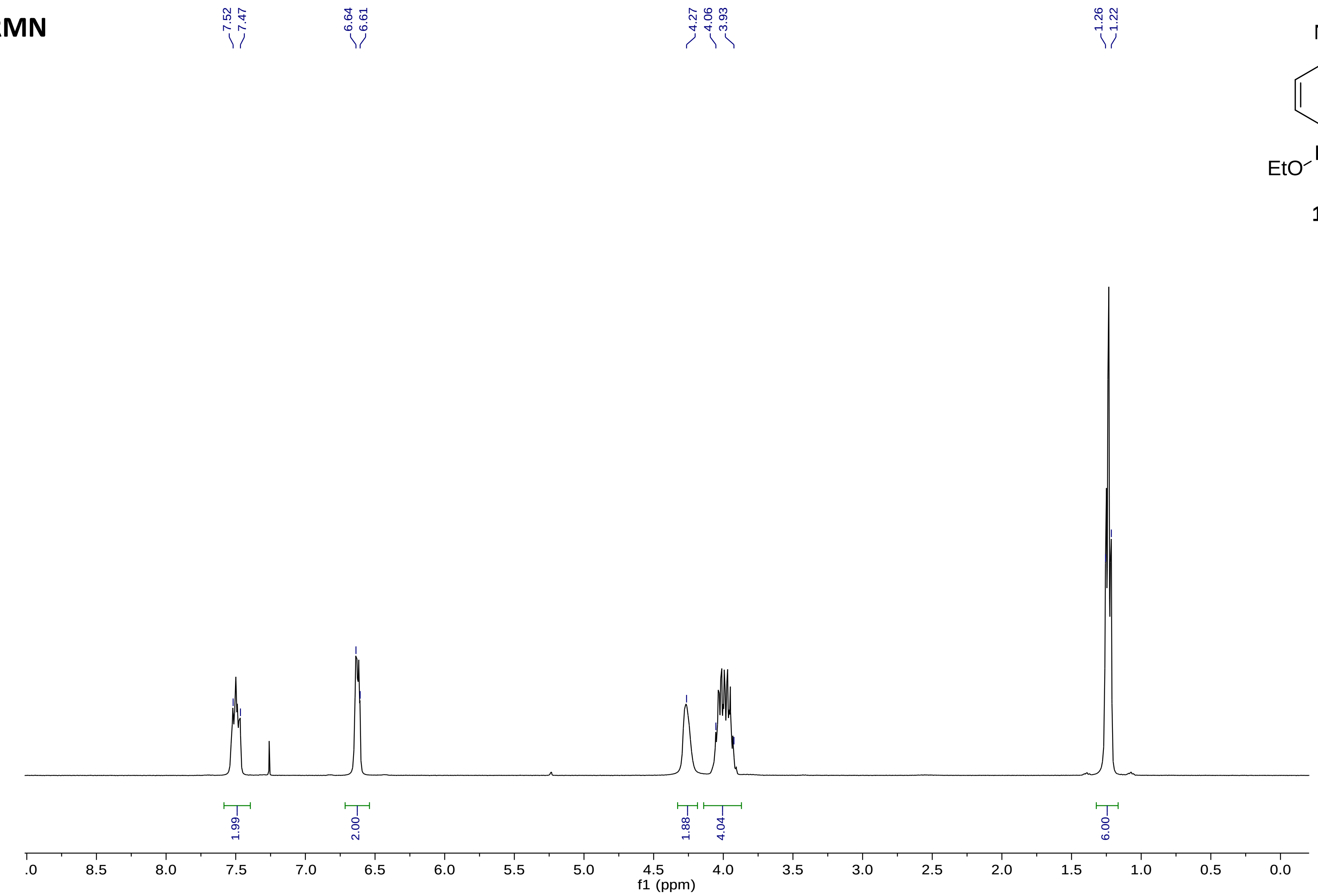

7.52
7.47
6.64
6.61
4.27
4.06
3.93
1.26
1.22
1.99
2.00
1.88
4.04
6.00
.0
8.5
8.0
7.5
7.0
6.5
6.0
5.5
5.0
4.5
4.0
3.5
3.0
2.5
2.0
1.5
1.0
0.5
0.0
f1 (ppm)

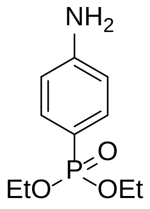

NH2
O
P
EtO
OEt

1c

^{13}C-RMN

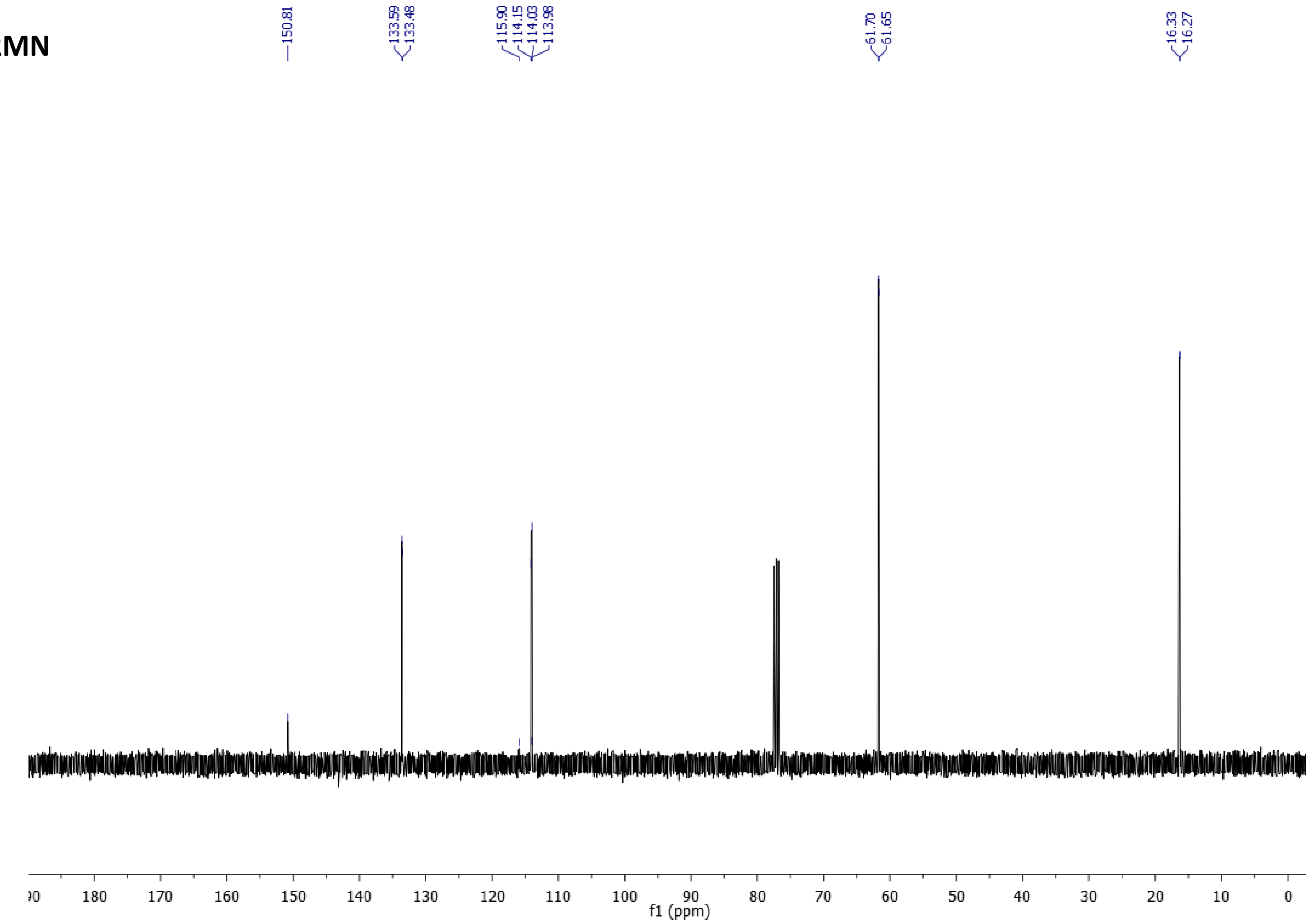
150.81
133.59
133.48
115.90
114.15
114.03
113.98
61.70
61.65
16.33
16.27
f1 (ppm)

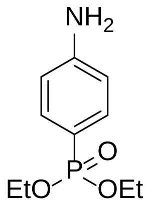
NH2
O
P
EtO
OEt

1c

^{31}P-RMN

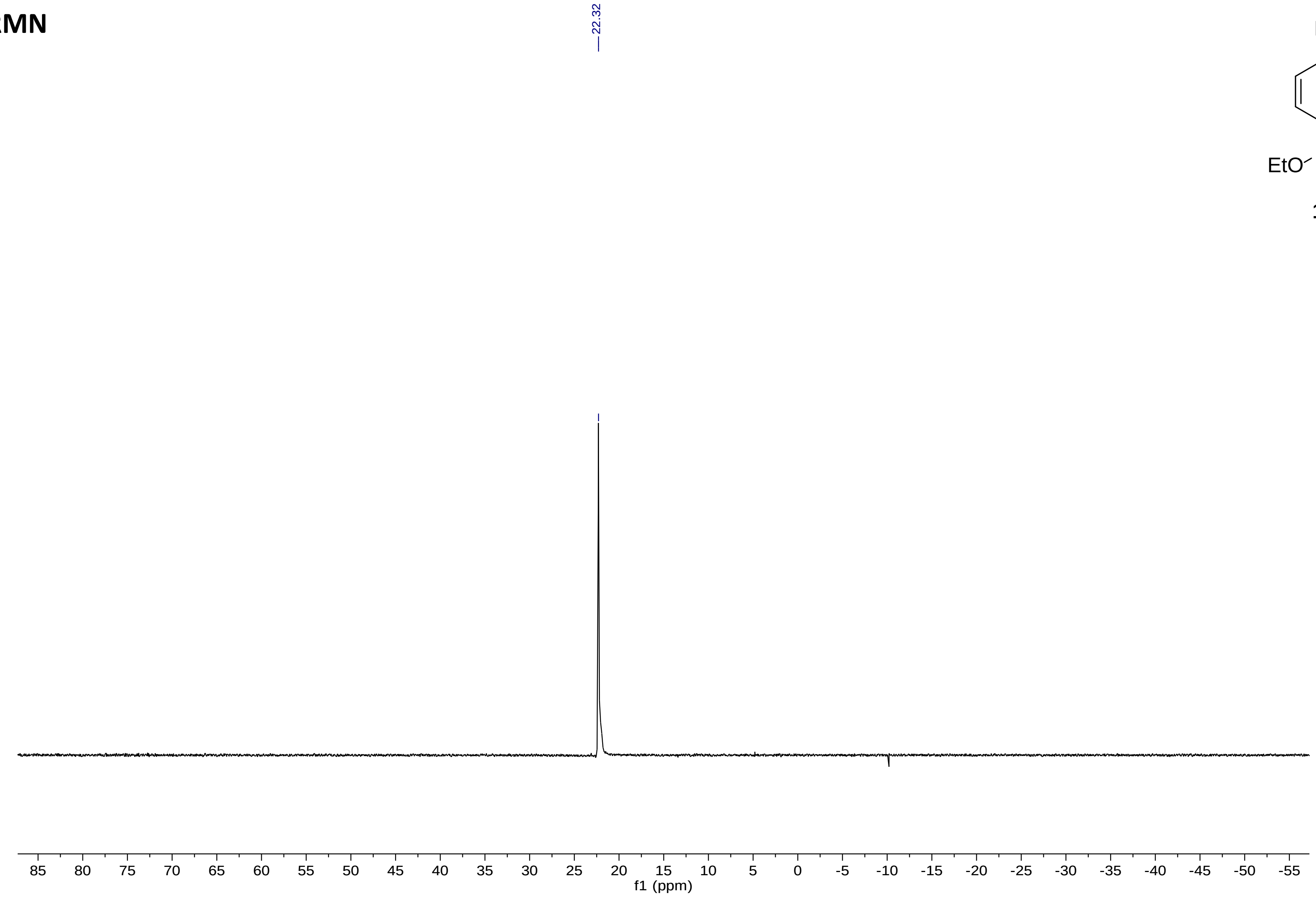
22.32
85 80 75 70 65 60 55 50 45 40 35 30 25 20 15 10 5 0 -5 -10 -15 -20 -25 -30 -35 -40 -45 -50 -55
f1 (ppm)

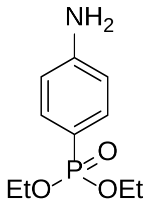
NH2
O
P
EtO
OEt

1c

^{1}H-RMN

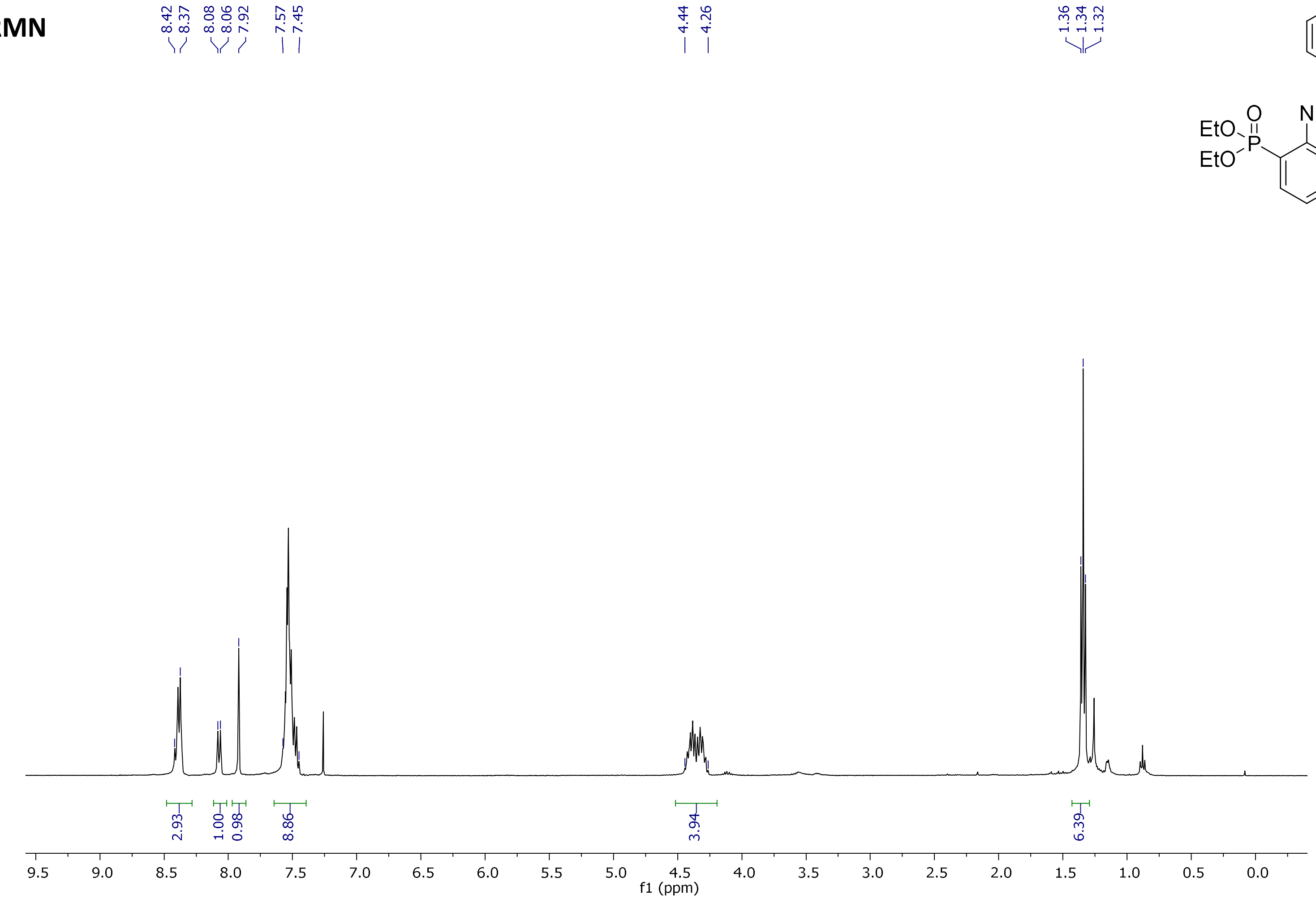

8.42
8.37
8.08
8.06
7.92
7.57
7.45
4.44
4.26
1.36
1.34
1.32
2.93
1.00
0.98
8.86
3.94
6.39
9.5 9.0 8.5 8.0 7.5 7.0 6.5 6.0 5.5 5.0 4.5 4.0 3.5 3.0 2.5 2.0 1.5 1.0 0.5 0.0
f1 (ppm)

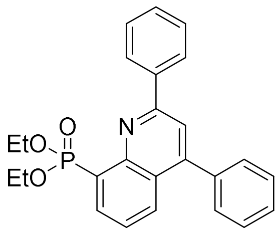

EtO
EtO
O
P
N

7a

^{13}C-RMN

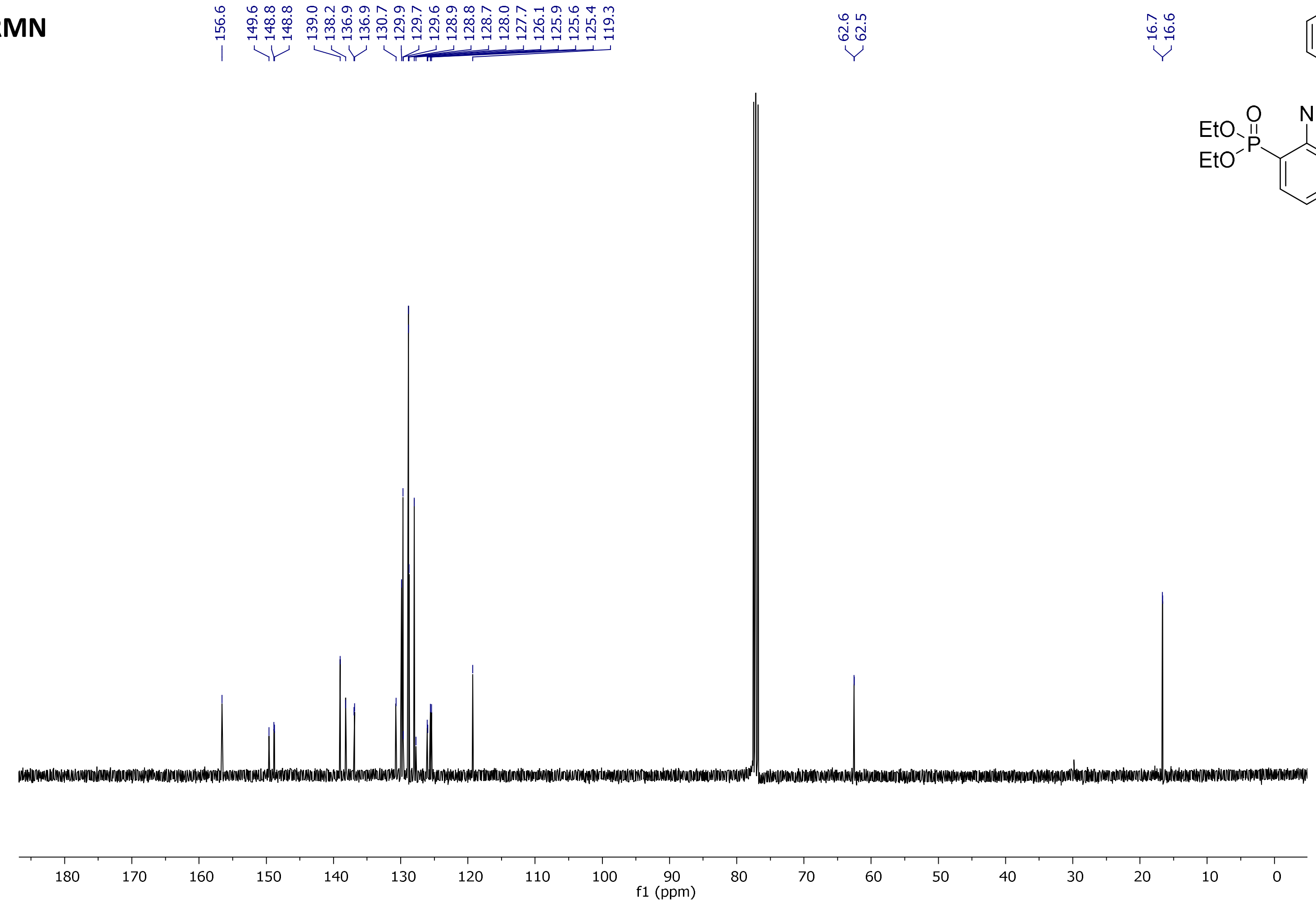

156.6
149.6
148.8
148.8
139.0
138.2
136.9
136.9
130.7
129.9
129.7
129.6
128.9
128.8
128.7
128.0
127.7
126.1
125.9
125.6
125.4
119.3
62.6
62.5
16.7
16.6
180
170
160
150
140
130
120
110
100
90
80
70
60
50
40
30
20
10
0
f1 (ppm)

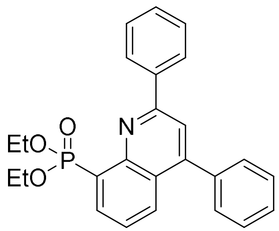

EtO
EtO
O
P
N

7a

^{31}P-RMN

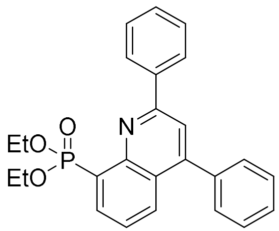

7a

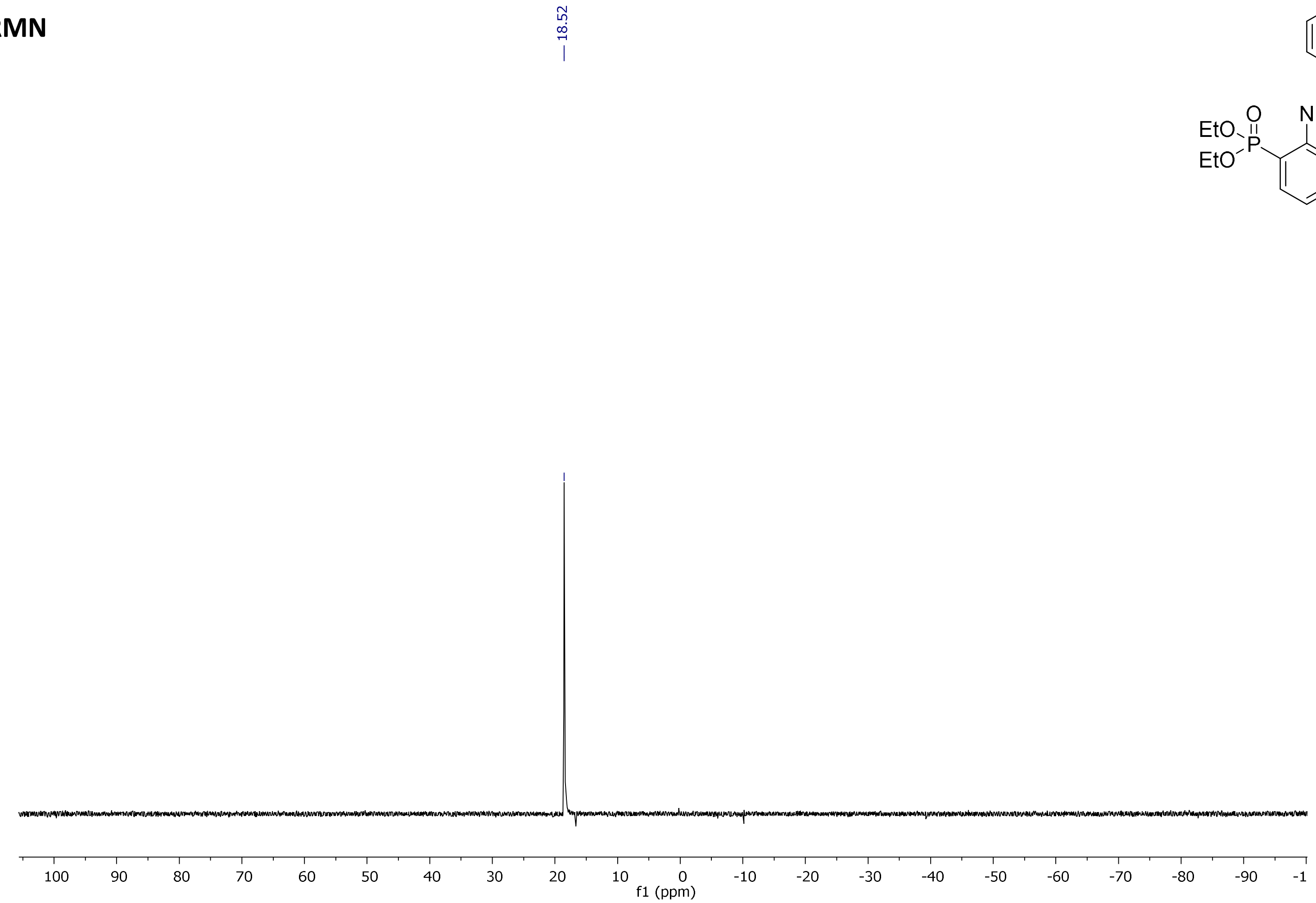

18.52
100
90
80
70
60
50
40
30
20
10
0
-10
-20
-30
-40
-50
-60
-70
-80
-90
-1
f1 (ppm)

^{1}H-RMN

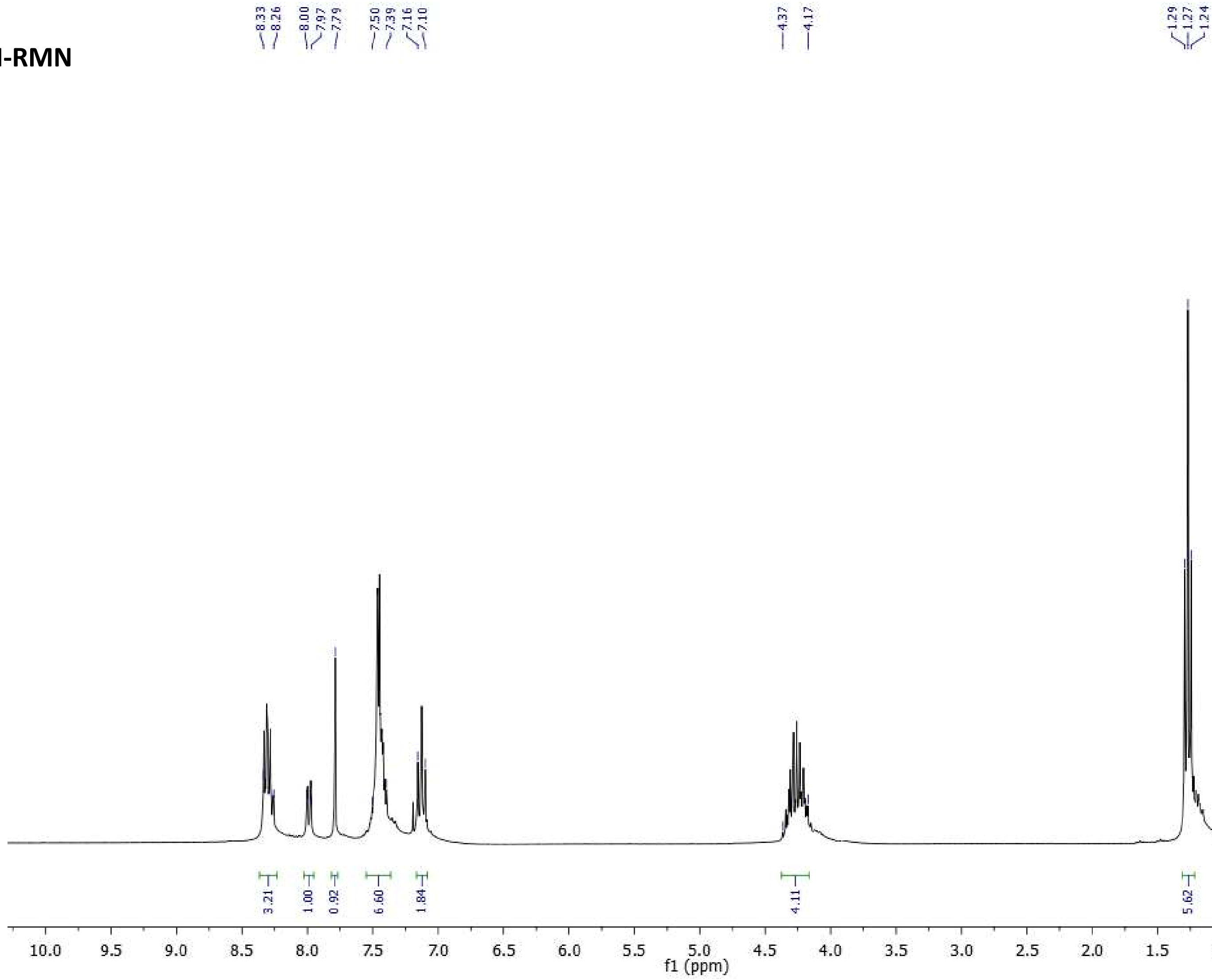

8.33
8.26
8.00
7.97
7.79
7.50
7.39
7.16
7.10
4.37
4.17
1.29
1.27
1.24
3.21
1.00
0.92
6.60
1.84
4.11
5.62
10.0 9.5 9.0 8.5 8.0 7.5 7.0 6.5 6.0 5.5 5.0 4.5 4.0 3.5 3.0 2.5 2.0 1.5 1.0 0.5 0.0
f1 (ppm)

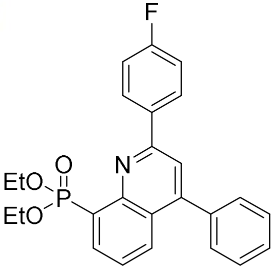

F
N
O
EtO
EtO
P

7b

^{13}C-RMN

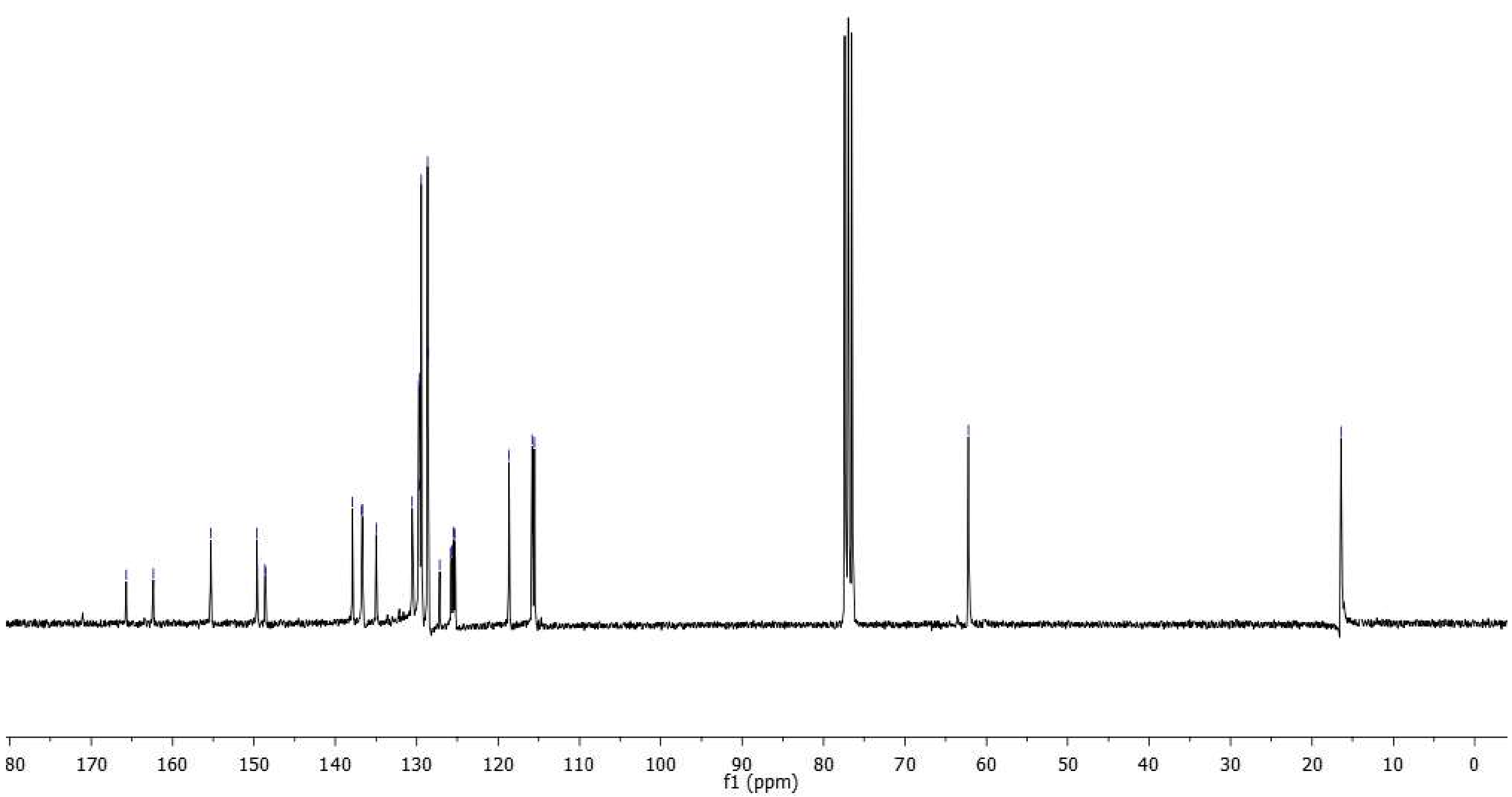

165.67
162.36
155.31
149.61
148.64
148.55
137.91
136.78
136.68
134.97
130.55
129.74
129.68
129.63
129.44
128.66
128.62
127.18
125.78
125.64
125.48
125.26
118.69
115.80
115.51
62.22
16.41
80 170 160 150 140 130 120 110 100 90 80 70 60 50 40 30 20 10 0
f1 (ppm)

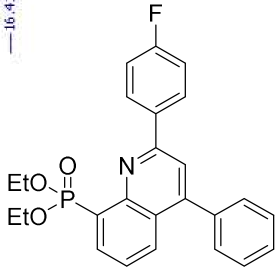

F
N
O
EtO
EtO
P

7b

^{31}P-RMN

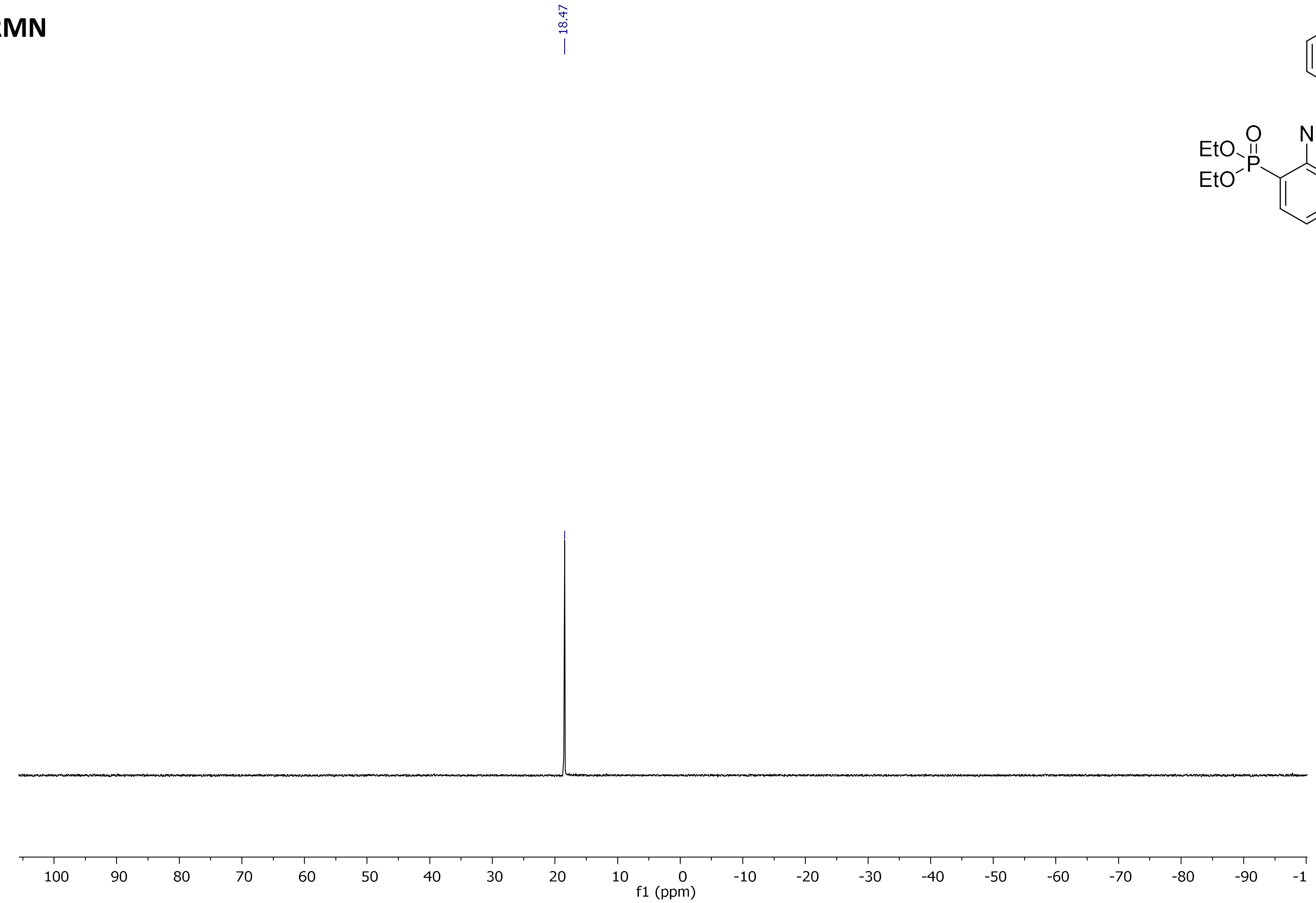

18.47
100 90 80 70 60 50 40 30 20 10 0 -10 -20 -30 -40 -50 -60 -70 -80 -90 -1
f1 (ppm)

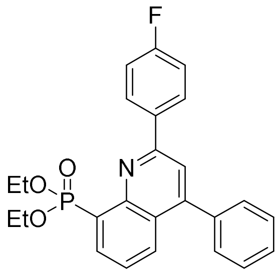

F
N
O
EtO
P
EtO

7b

^{19}F-RMN

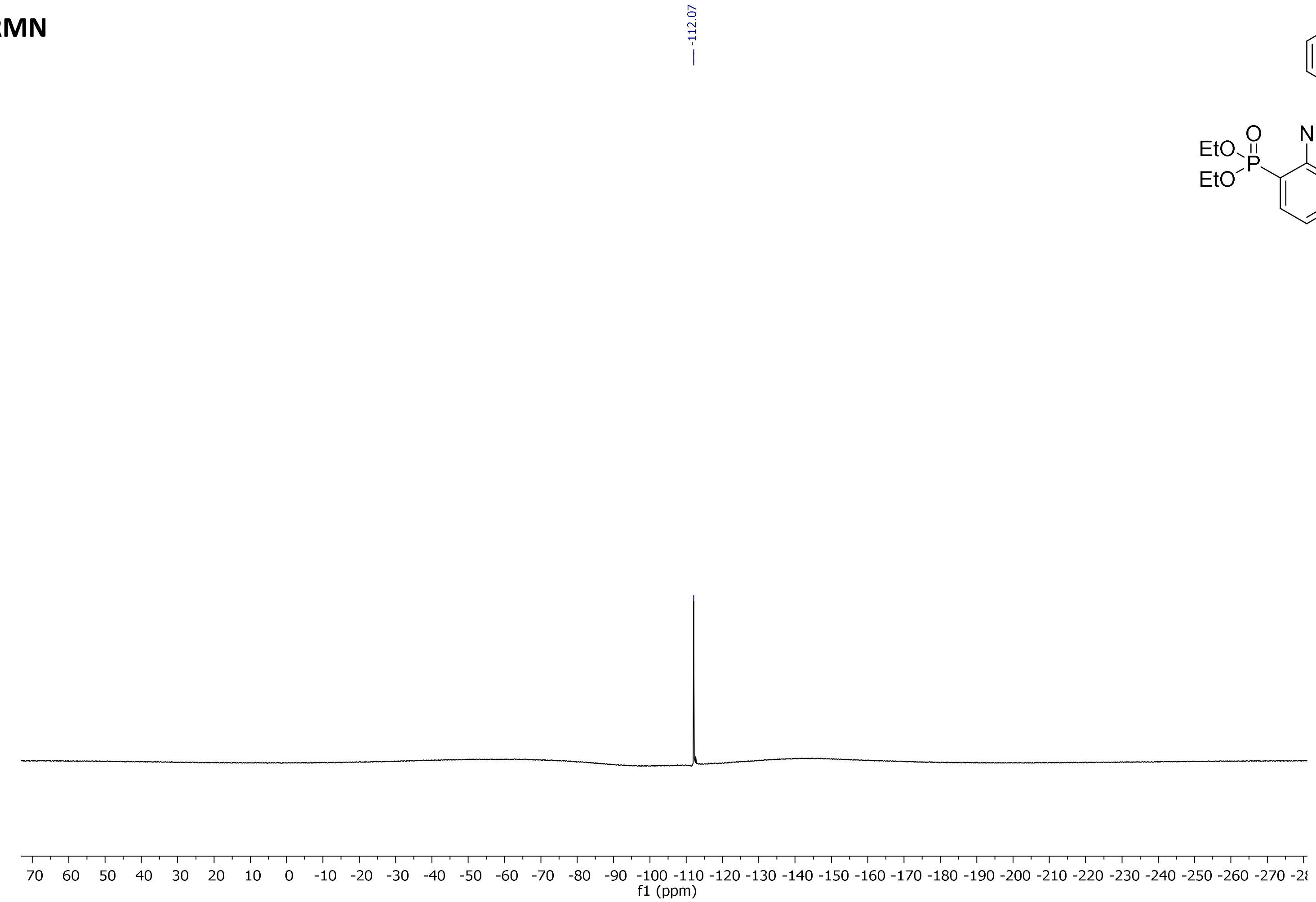

-112.07
70 60 50 40 30 20 10 0 -10 -20 -30 -40 -50 -60 -70 -80 -90 -100 -110 -120 -130 -140 -150 -160 -170 -180 -190 -200 -210 -220 -230 -240 -250 -260 -270
f1 (ppm)

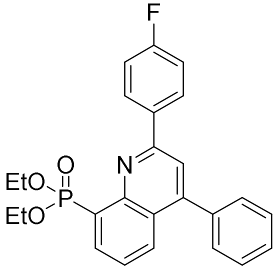

F
N
O
EtO
P
EtO

7b

^{1}H-RMN

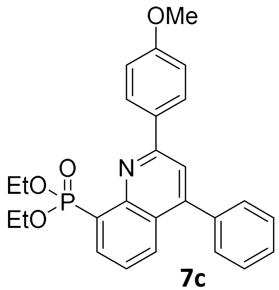

OMe
N
EtO
EtO
O
P

7c

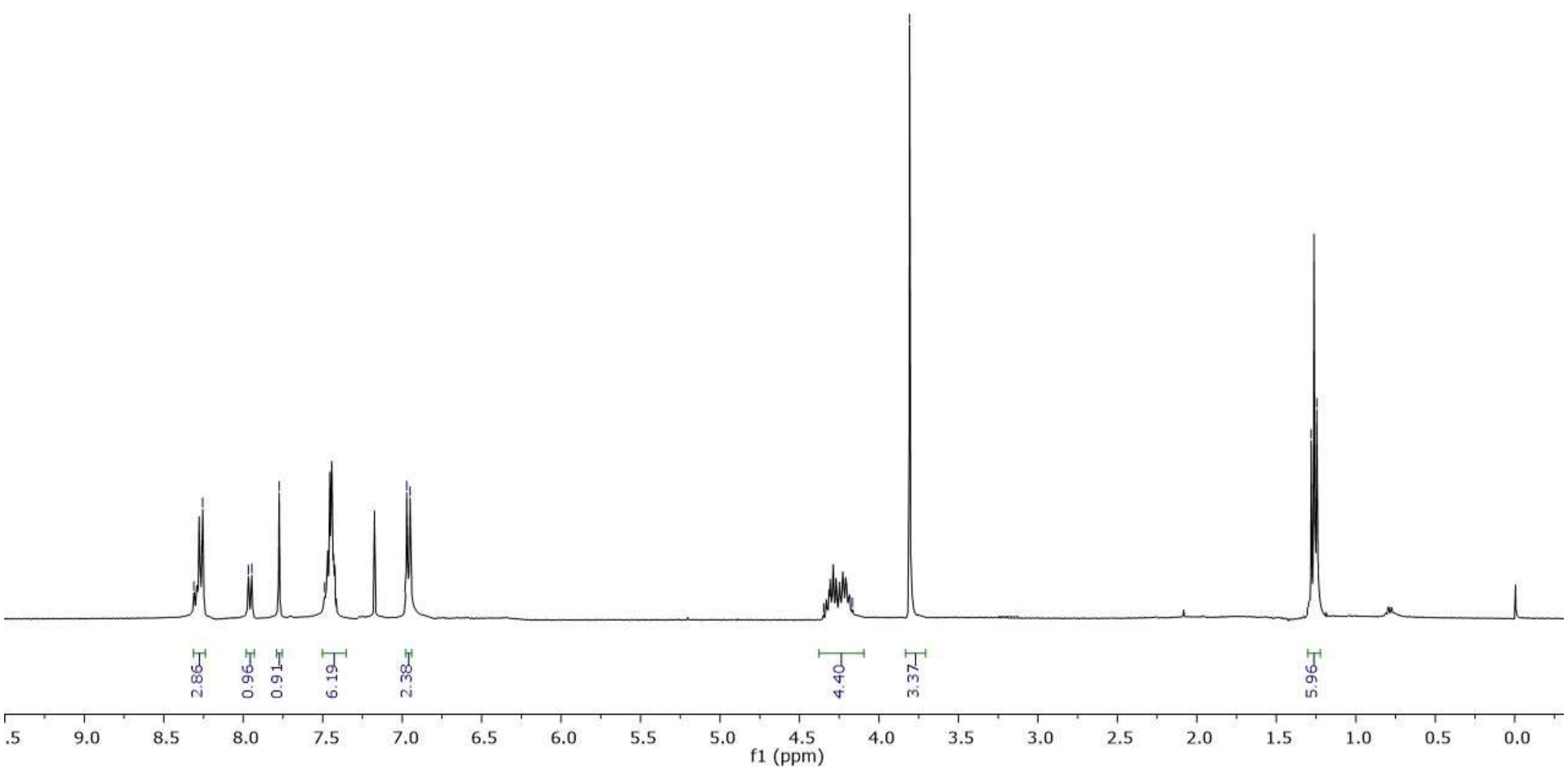

8.31
8.26
7.97
7.95
7.77
7.49
7.36
6.97
6.95
4.35
4.17
3.81
1.28
1.25
2.86
0.96
0.91
6.19
2.38
4.40
3.37
5.96
.5
9.0
8.5
8.0
7.5
7.0
6.5
6.0
5.5
5.0
4.5
4.0
3.5
3.0
2.5
2.0
1.5
1.0
0.5
0.0
f1 (ppm)

^{13}C-RMN

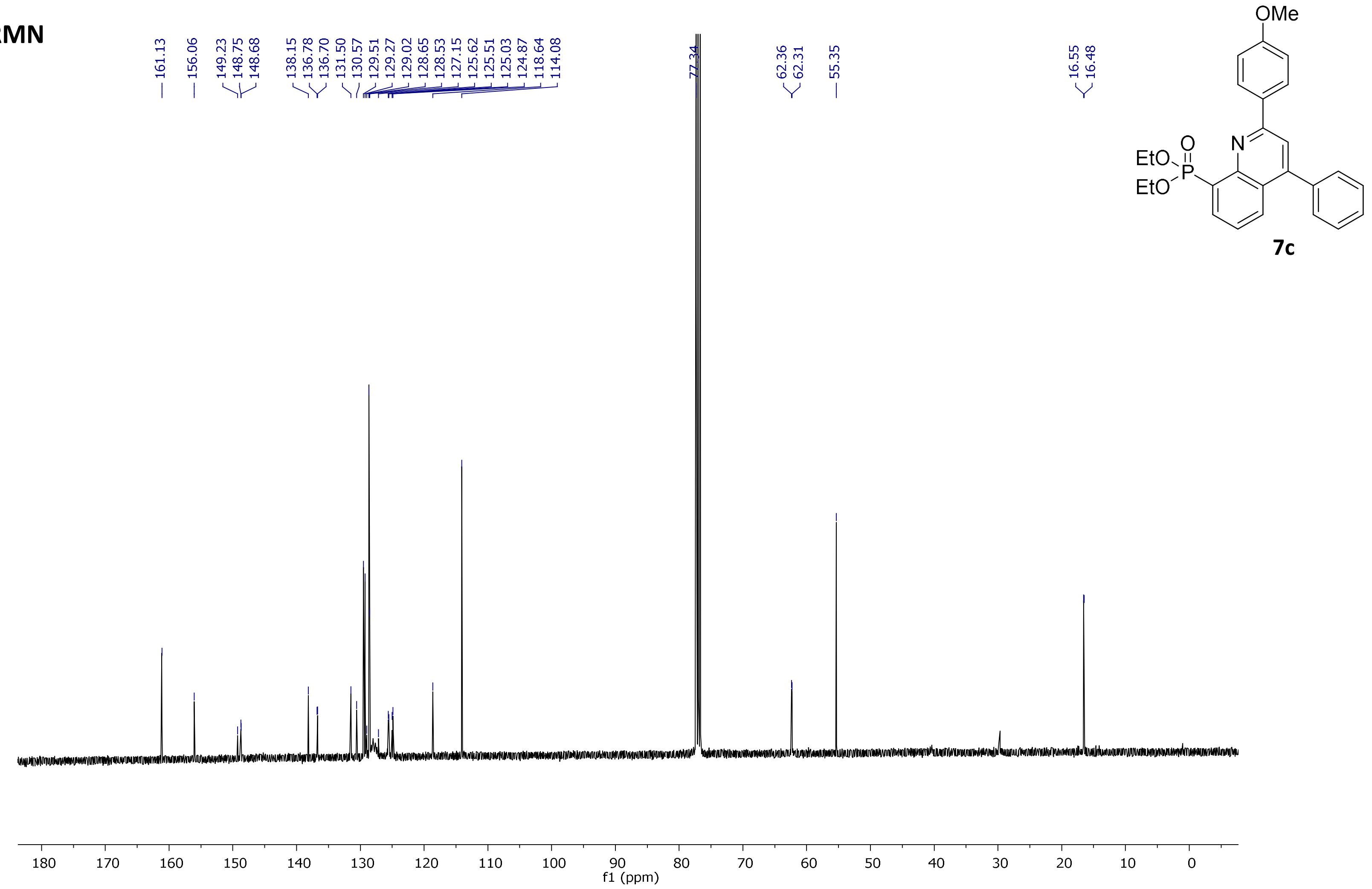

161.13
156.06
149.23
148.75
148.68
138.15
136.78
136.70
131.50
130.57
129.51
129.27
129.02
128.65
128.53
127.15
125.62
125.51
125.03
124.87
118.64
114.08
77.34
62.36
62.31
55.35
16.55
16.48
OMe
N
EtO
EtO
O
P
180
170
160
150
140
130
120
110
100
90
80
70
60
50
40
30
20
10
0
f1 (ppm)

7c

^{31}P-RMN

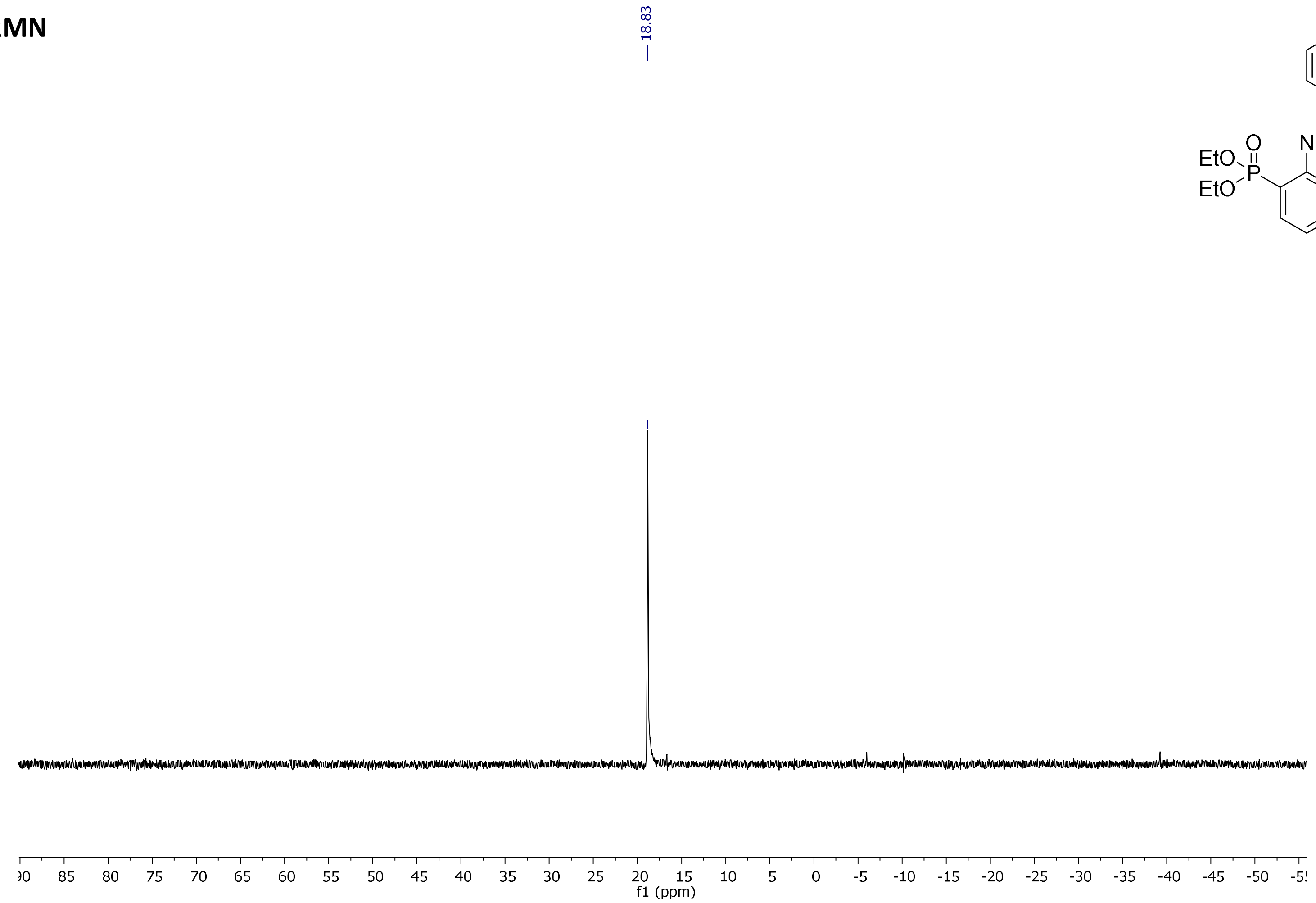

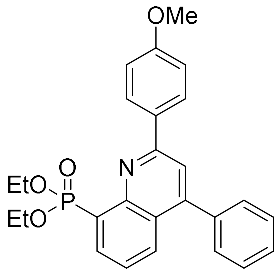

7c

^{1}H-RMN

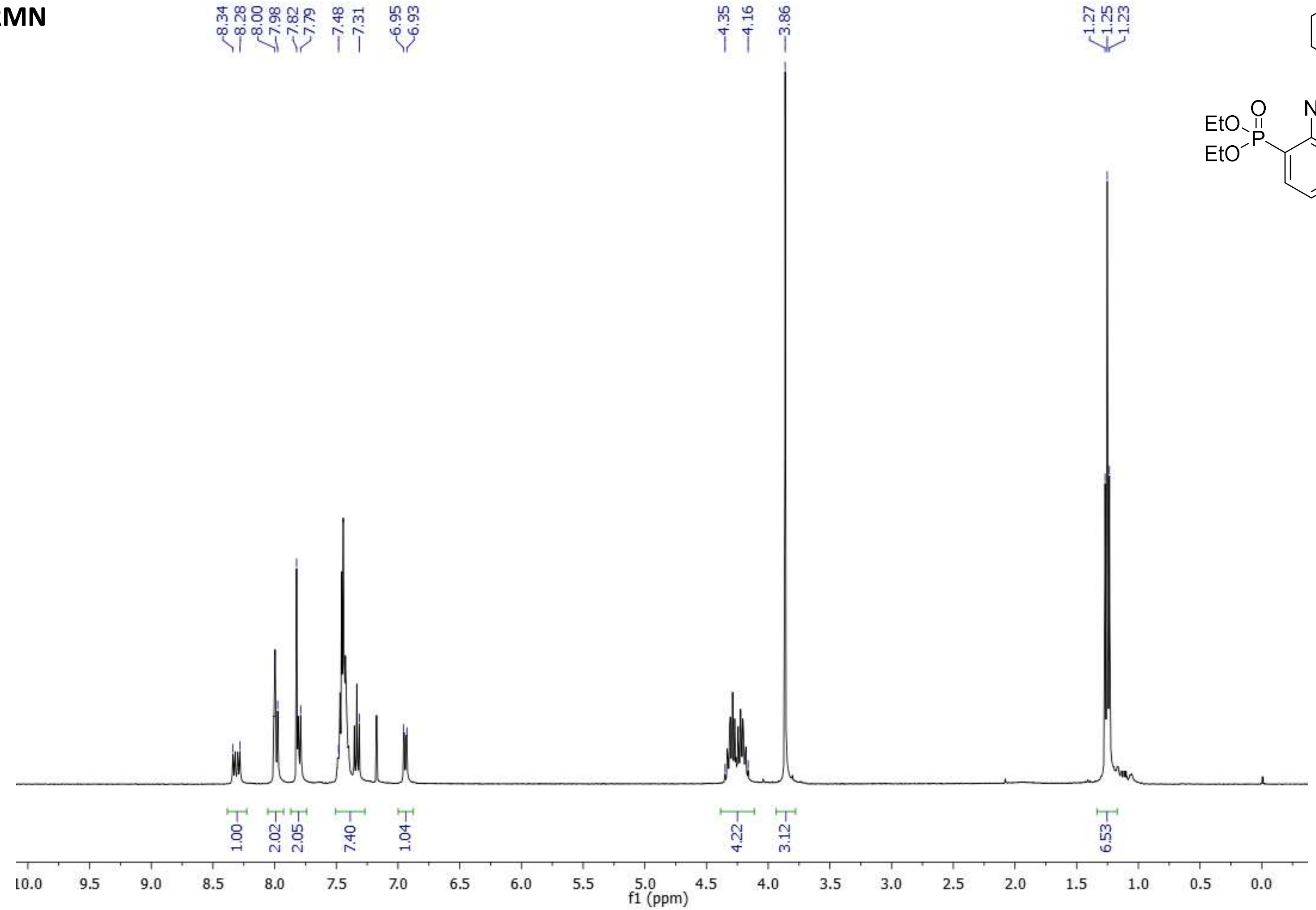

8.34
8.28
8.00
7.98
7.82
7.79
7.48
7.31
6.95
6.93
4.35
4.16
3.86
1.27
1.25
1.23
1.00
2.02
2.05
7.40
1.04
4.22
3.12
6.53
10.0 9.5 9.0 8.5 8.0 7.5 7.0 6.5 6.0 5.5 5.0 4.5 4.0 3.5 3.0 2.5 2.0 1.5 1.0 0.5 0.0
f1 (ppm)

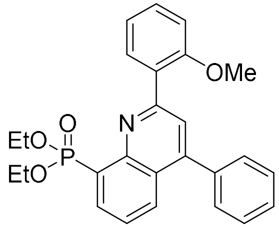

OMe
N
O
EtO
P
EtO

7d

^{13}C-RMN

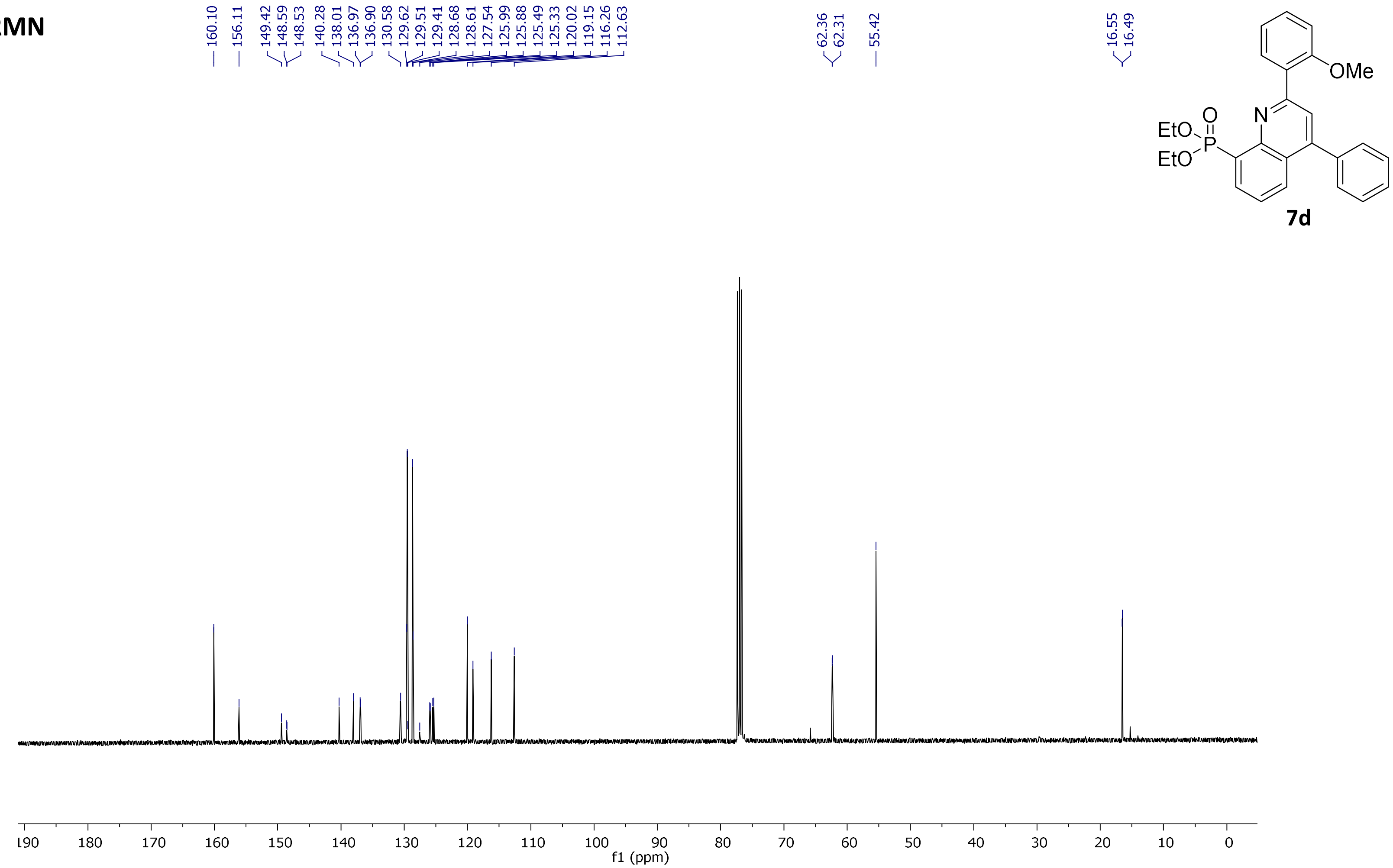

160.10
156.11
149.42
148.59
148.53
140.28
138.01
136.97
136.90
130.58
129.62
129.51
129.41
128.68
128.61
127.54
125.99
125.88
125.49
125.33
120.02
119.15
116.26
112.63
62.36
62.31
55.42
16.55
16.49
OMe
N
O
EtO
P
EtO
190 180 170 160 150 140 130 120 110 100 90 80 70 60 50 40 30 20 10 0
f1 (ppm)

7d

^{31}P-RMN

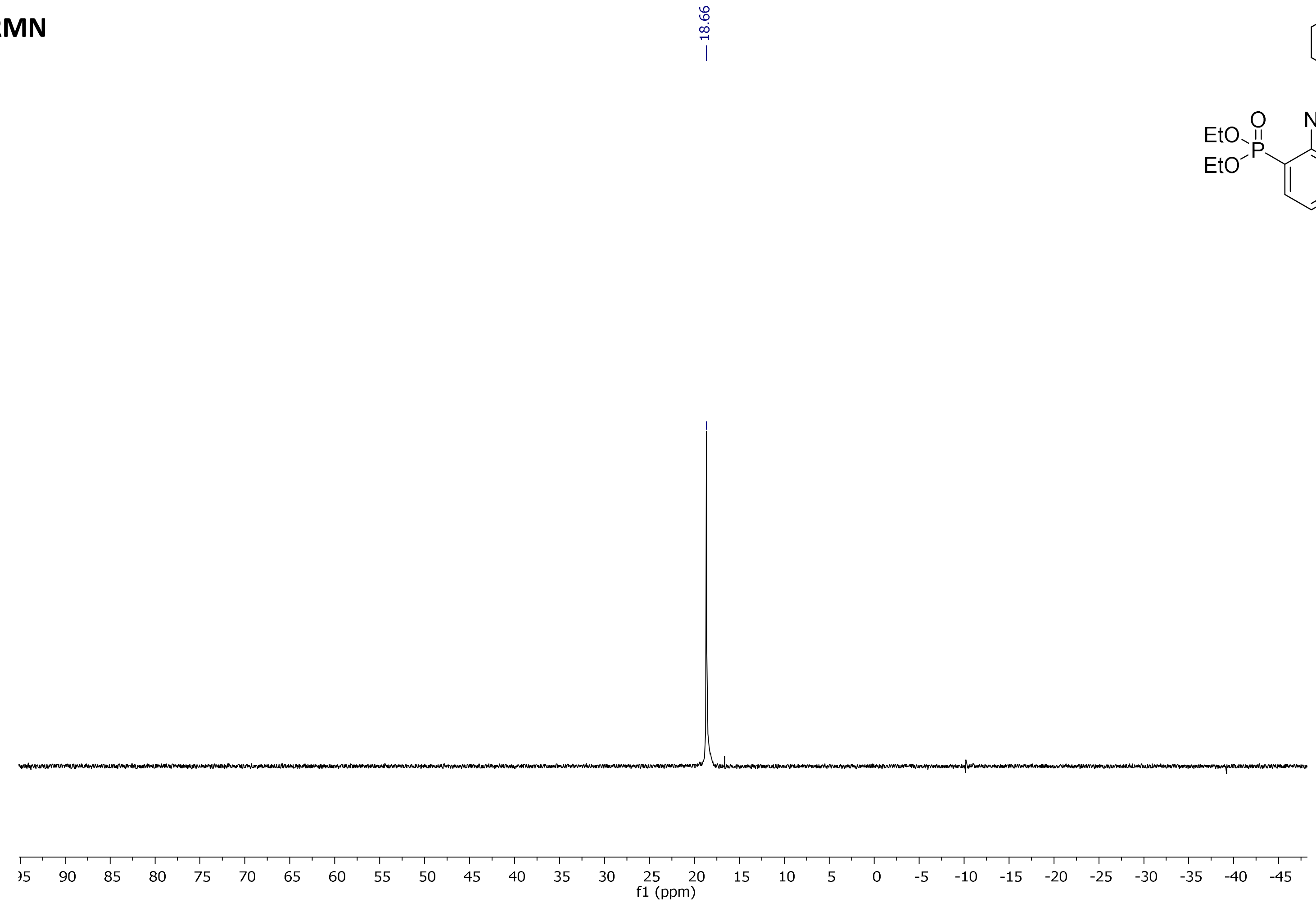

18.66
95 90 85 80 75 70 65 60 55 50 45 40 35 30 25 20 15 10 5 0 -5 -10 -15 -20 -25 -30 -35 -40 -45
f1 (ppm)

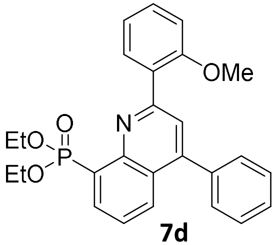

OMe
N
O
EtO
P
EtO

7d

^{1}H-RMN

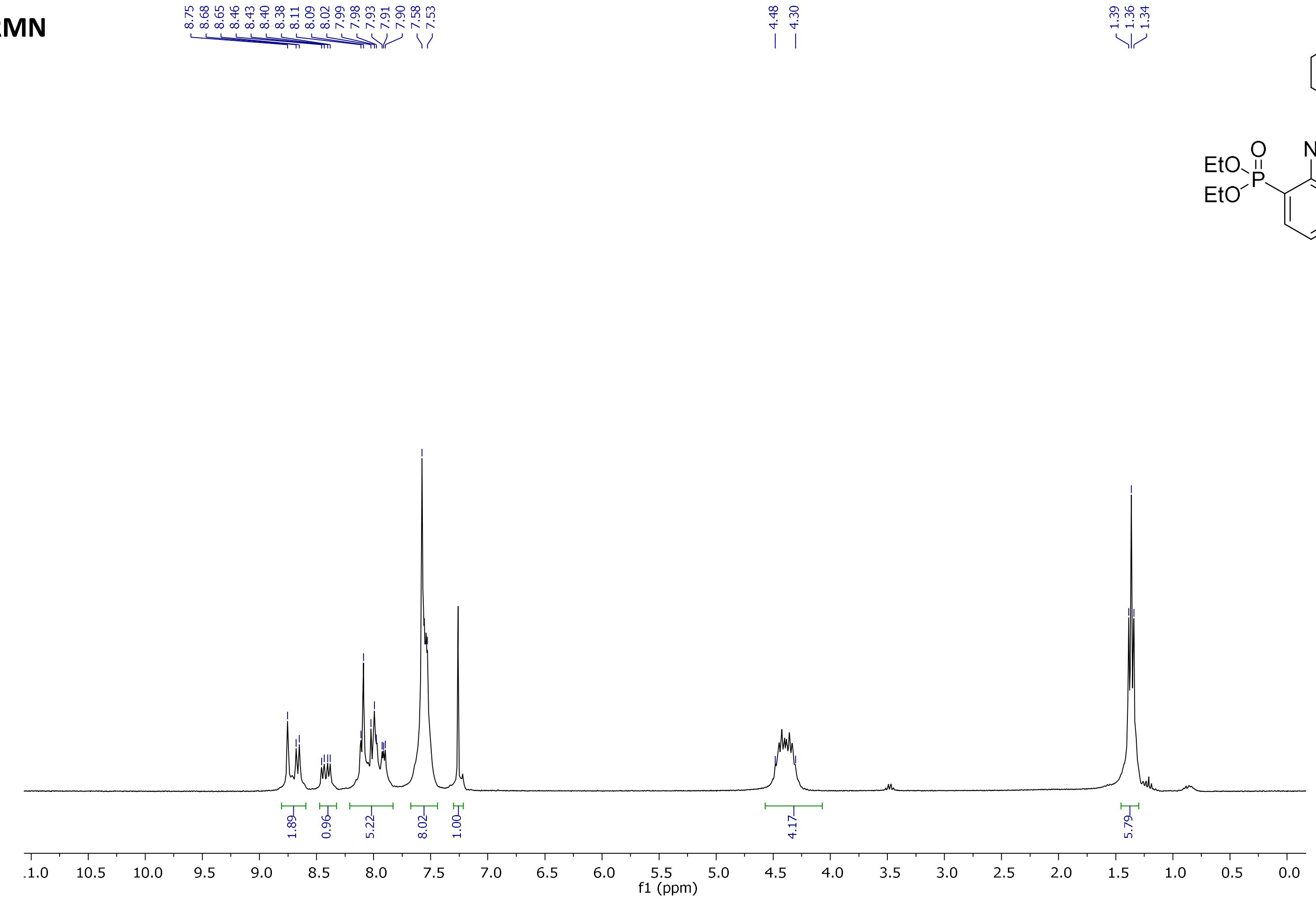

8.75 8.68 8.65 8.46 8.43 8.40 8.38 8.11 8.09 8.02 7.99 7.98 7.93 7.91 7.90 7.58 7.53
4.48 4.30
1.39 1.36 1.34
1.89 0.96 5.22 8.02 1.00 4.17 5.79
11.0 10.5 10.0 9.5 9.0 8.5 8.0 7.5 7.0 6.5 6.0 5.5 5.0 4.5 4.0 3.5 3.0 2.5 2.0 1.5 1.0 0.5 0.0
f1 (ppm)

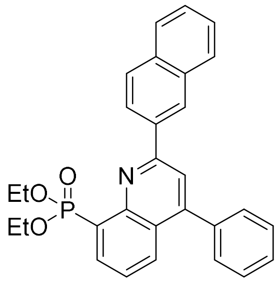

EtO
EtO
O
P
N

7e

^{13}C-RMN

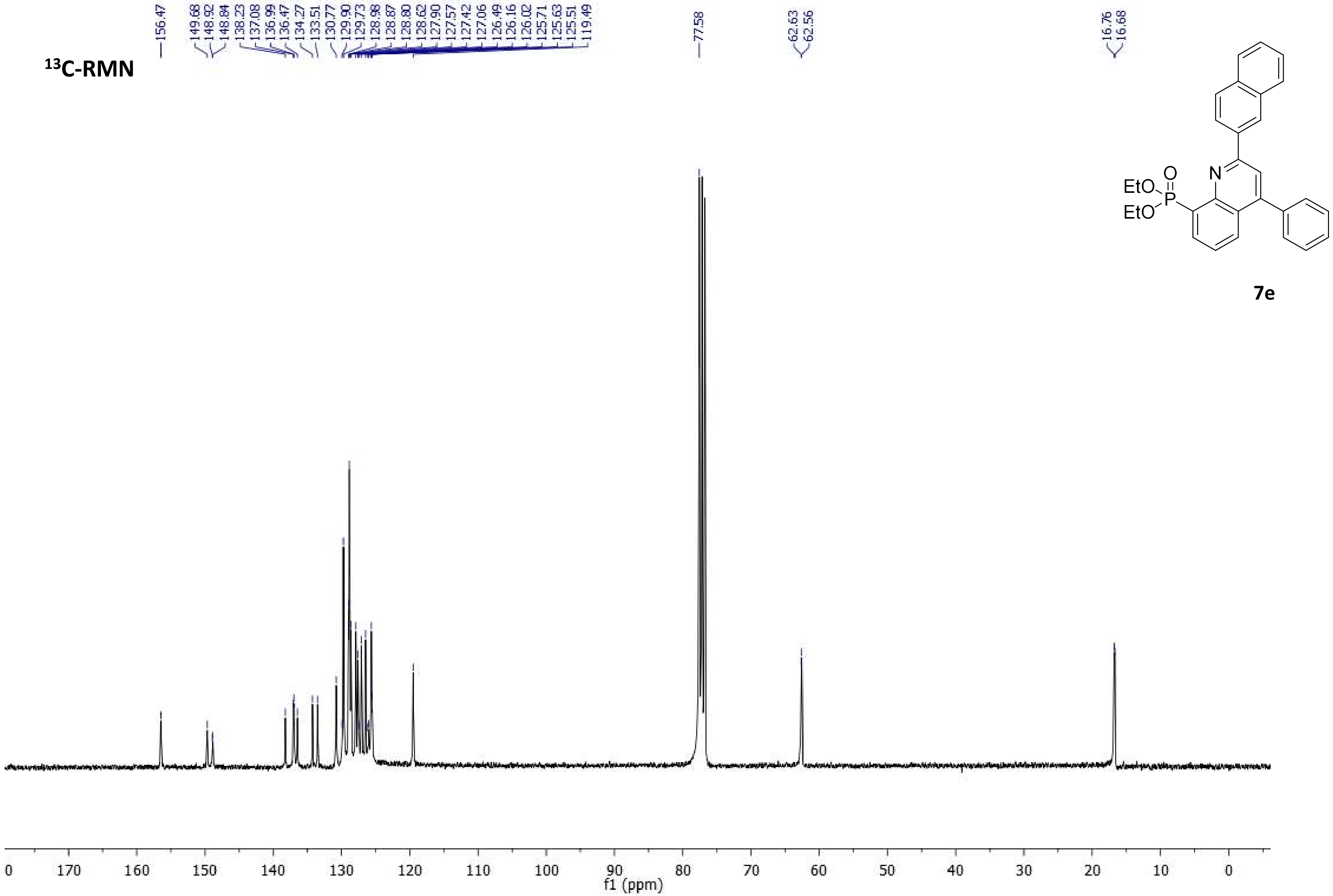

156.47
149.68
148.92
148.84
138.23
137.08
136.99
136.47
134.27
133.51
130.77
129.90
129.73
128.98
128.87
128.80
128.62
127.90
127.57
127.42
127.06
126.49
126.16
126.02
125.71
125.63
125.51
119.49
77.58
62.63
62.56
16.76
16.68
f1 (ppm)
EtO
EtO
P
O
N

7e

^{31}P-RMN

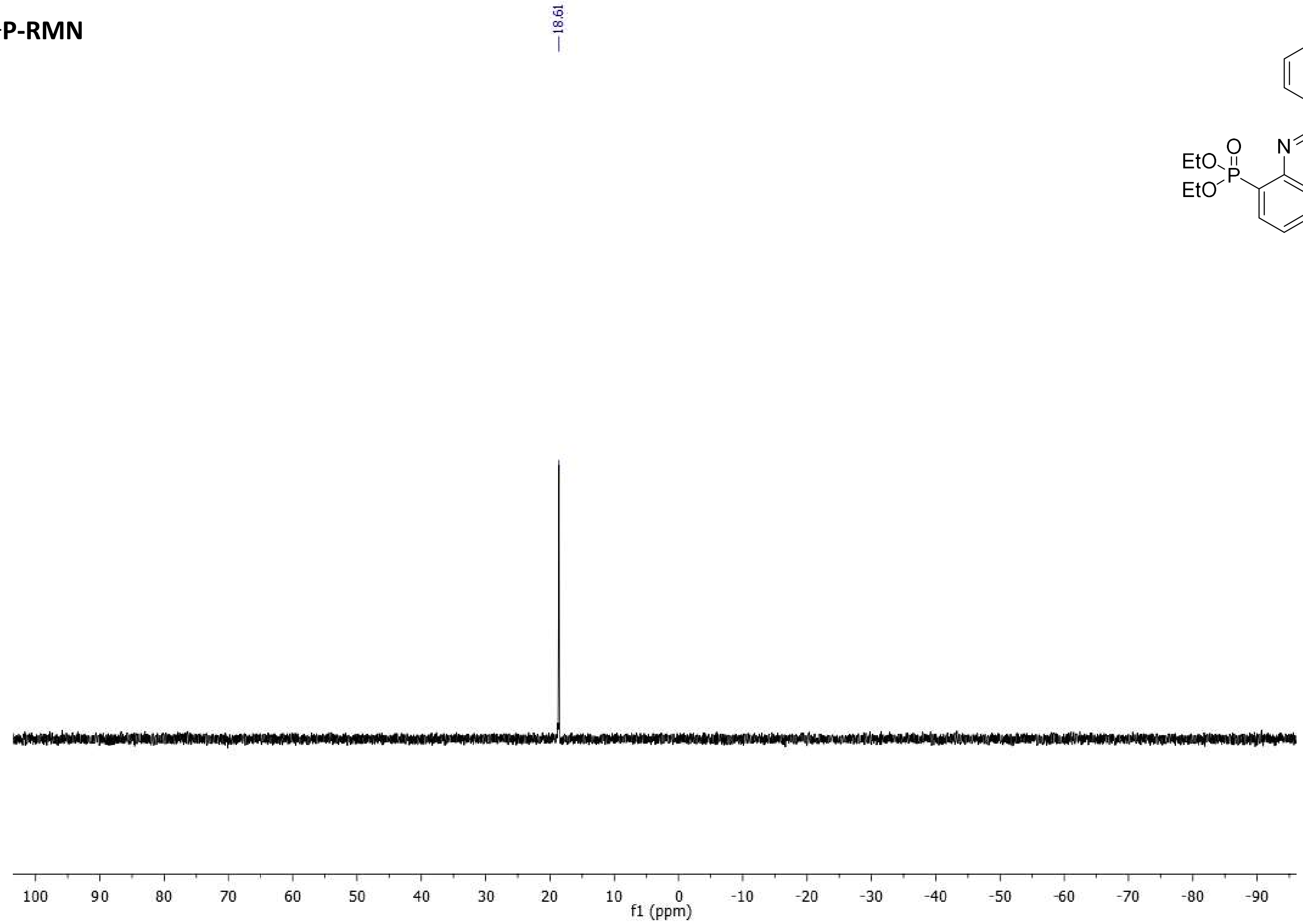

18.61
100
90
80
70
60
50
40
30
20
10
0
-10
-20
-30
-40
-50
-60
-70
-80
-90
f1 (ppm)

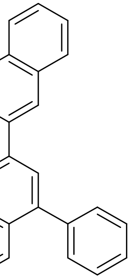

EtO
EtO
O
P
N

7e

^{1}H-RMN

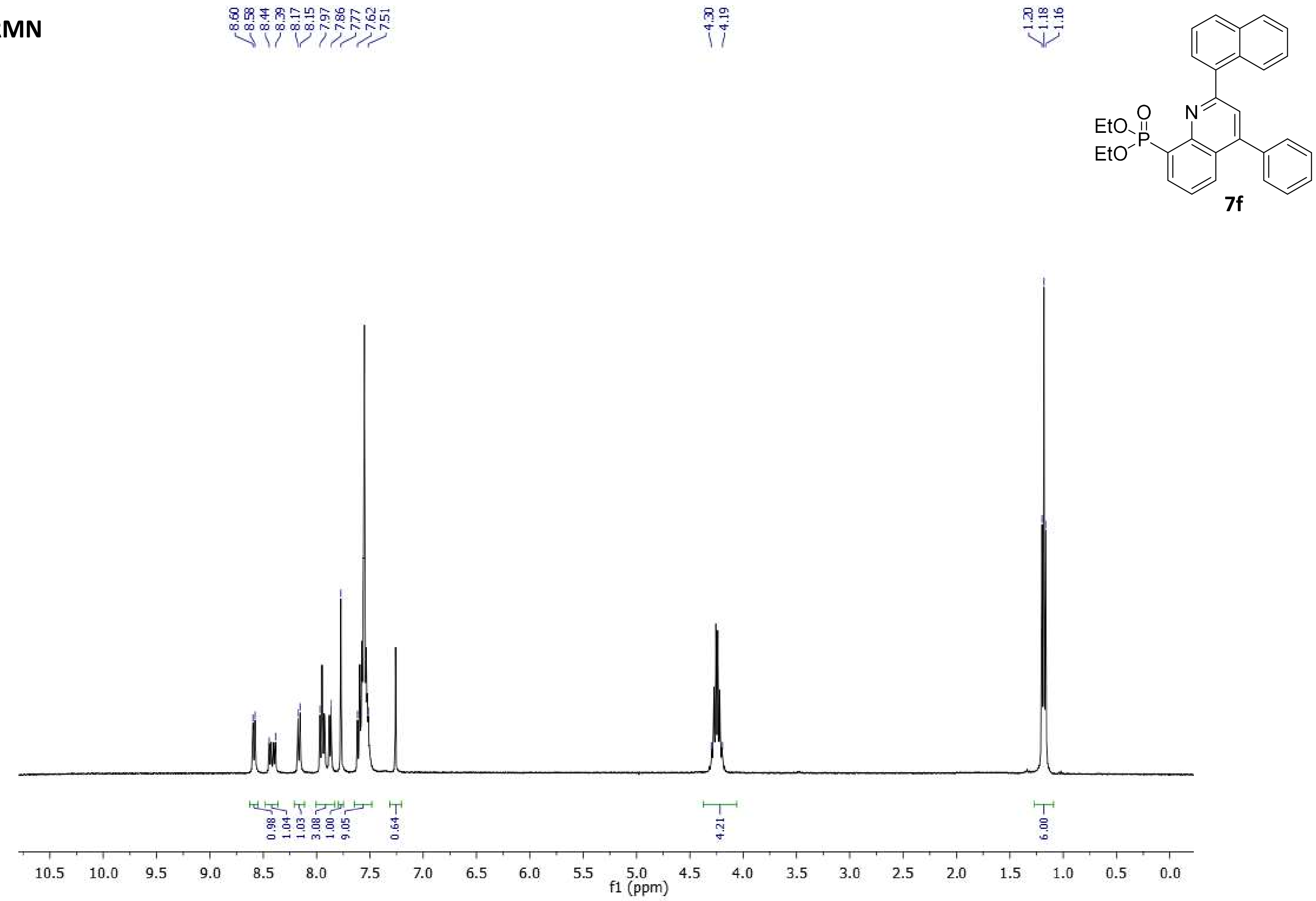

8.60
8.58
8.44
8.39
8.17
8.15
7.97
7.86
7.77
7.62
7.51
4.30
4.19
1.20
1.18
1.16
EtO
EtO
O
P
N
7f
0.98
1.04
1.03
3.08
1.00
9.05
0.64
4.21
6.00
10.5
10.0
9.5
9.0
8.5
8.0
7.5
7.0
6.5
6.0
5.5
5.0
4.5
4.0
3.5
3.0
2.5
2.0
1.5
1.0
0.5
0.0
f1 (ppm)

^{13}C-RMN

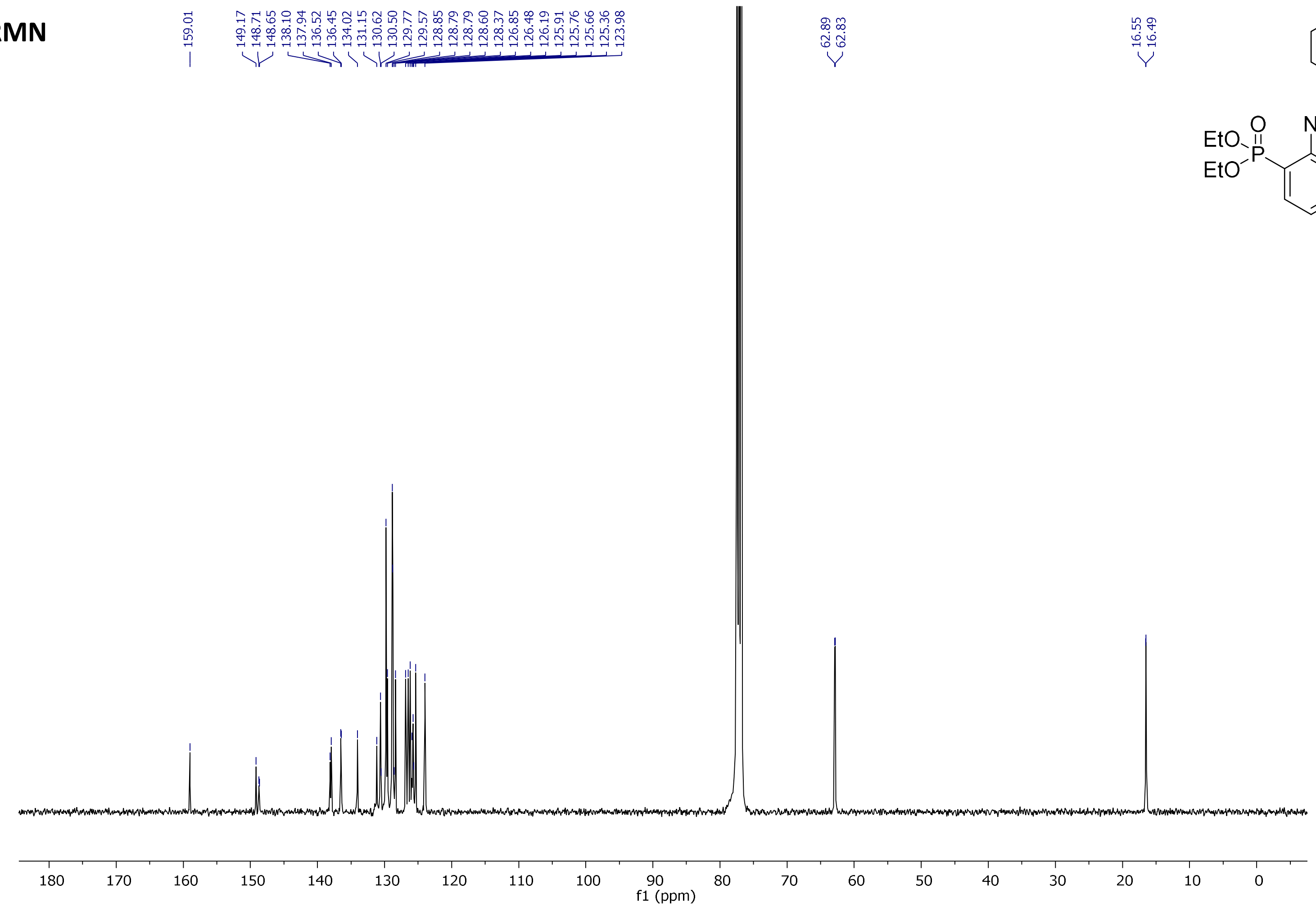

159.01
149.17
148.71
148.65
138.10
137.94
136.52
136.45
134.02
131.15
130.62
130.50
129.77
129.57
128.85
128.79
128.79
128.60
128.37
126.85
126.48
126.19
125.91
125.76
125.66
125.36
123.98
62.89
62.83
16.55
16.49
180 170 160 150 140 130 120 110 100 90 80 70 60 50 40 30 20 10 0
f1 (ppm)

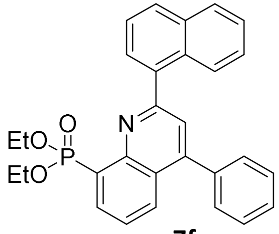

EtO
EtO
O
P
N

7f

^{31}P-RMN

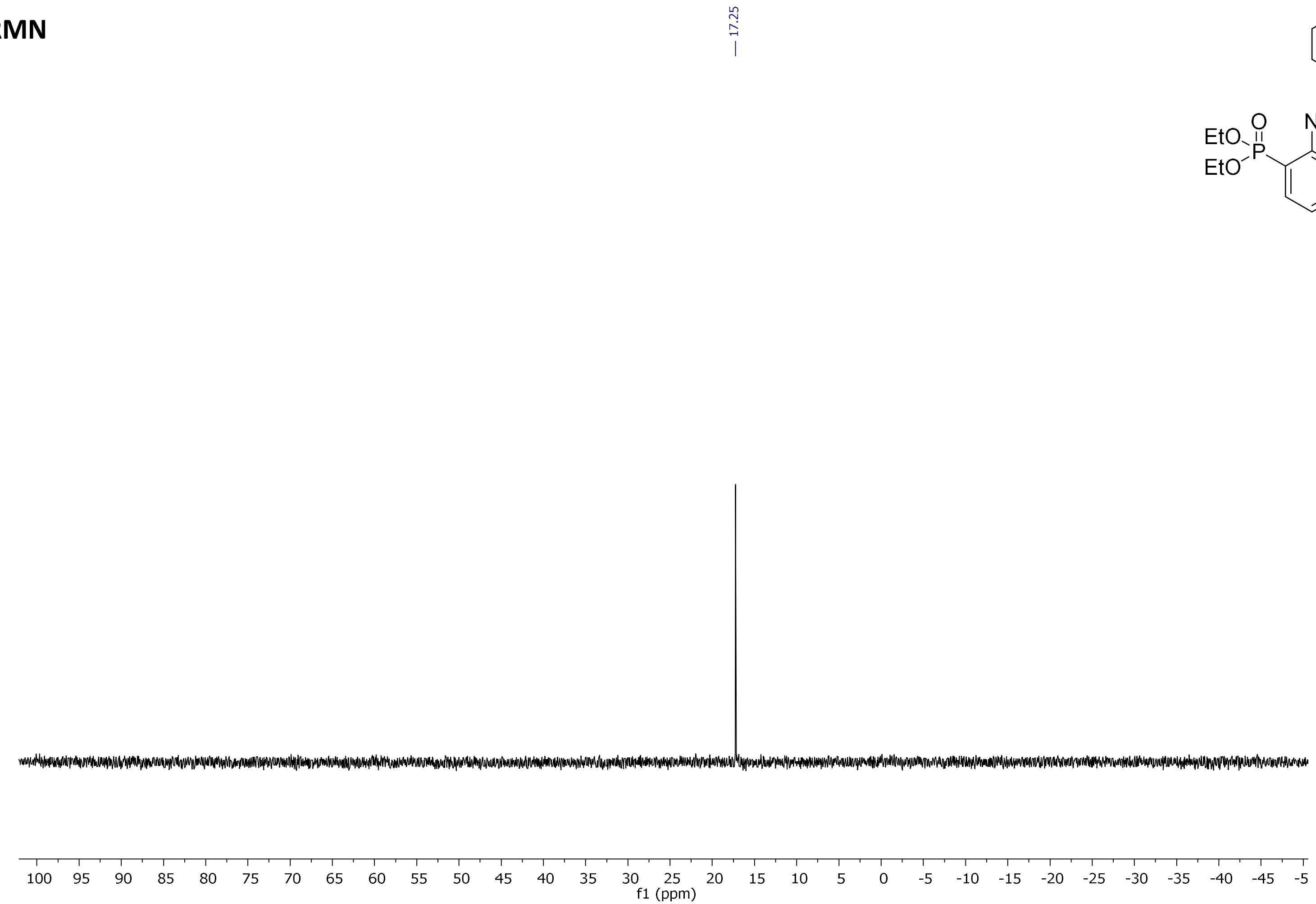

17.25
100 95 90 85 80 75 70 65 60 55 50 45 40 35 30 25 20 15 10 5 0 -5 -10 -15 -20 -25 -30 -35 -40 -45 -5
f1 (ppm)

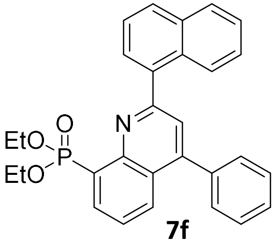

EtO
EtO
O
P
N

7f

1H-RMN

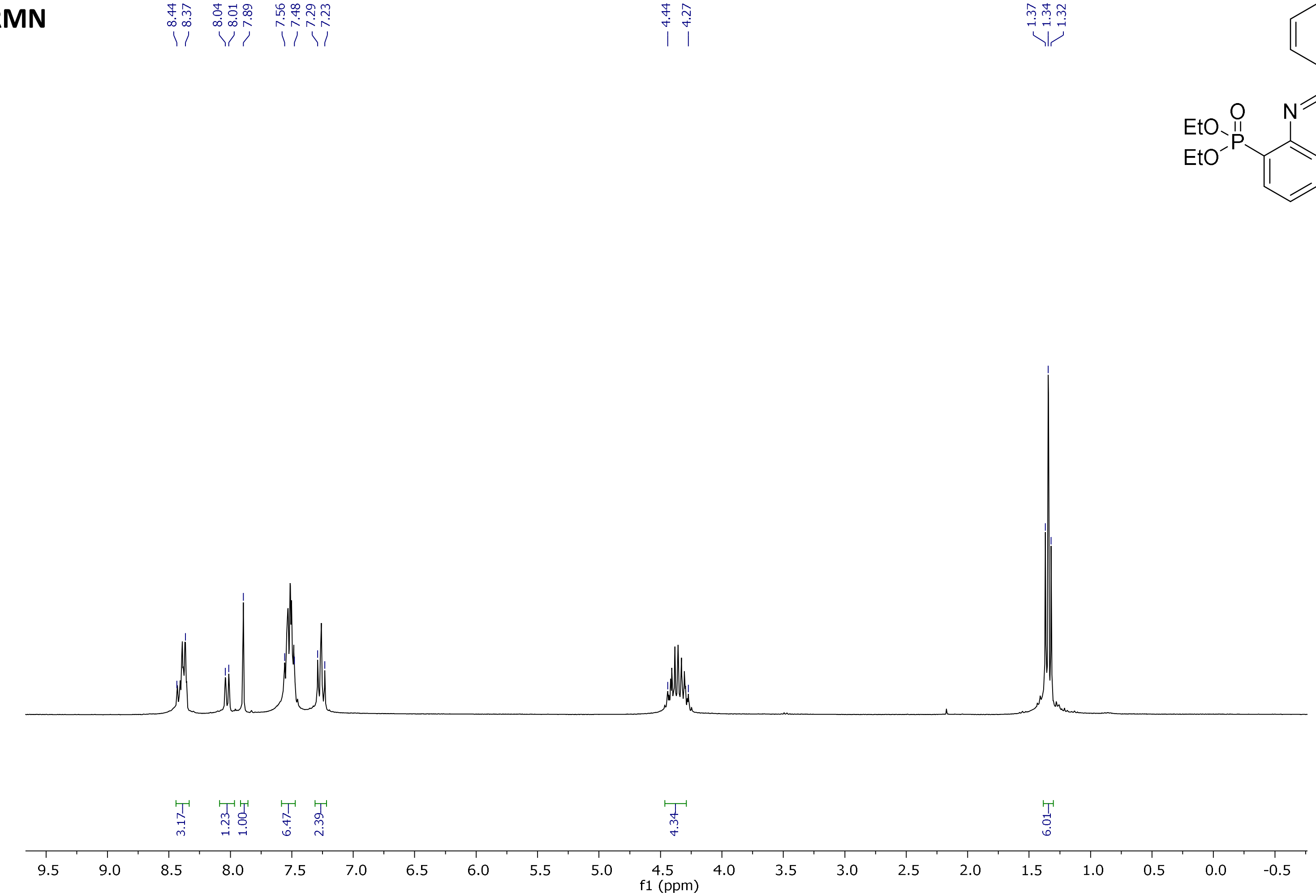
8.44
8.37
8.04
8.01
7.89
7.56
7.48
7.29
7.23
4.44
4.27
1.37
1.34
1.32
3.17
1.23
1.00
6.47
2.39
4.34
6.01
9.5 9.0 8.5 8.0 7.5 7.0 6.5 6.0 5.5 5.0 4.5 4.0 3.5 3.0 2.5 2.0 1.5 1.0 0.5 0.0 -0.5
f1 (ppm)

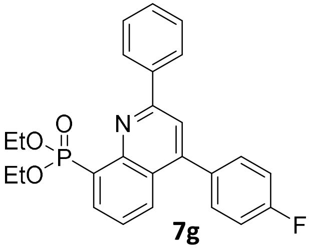
EtO
EtO
P
O
N
F

7g

^{13}C-RMN

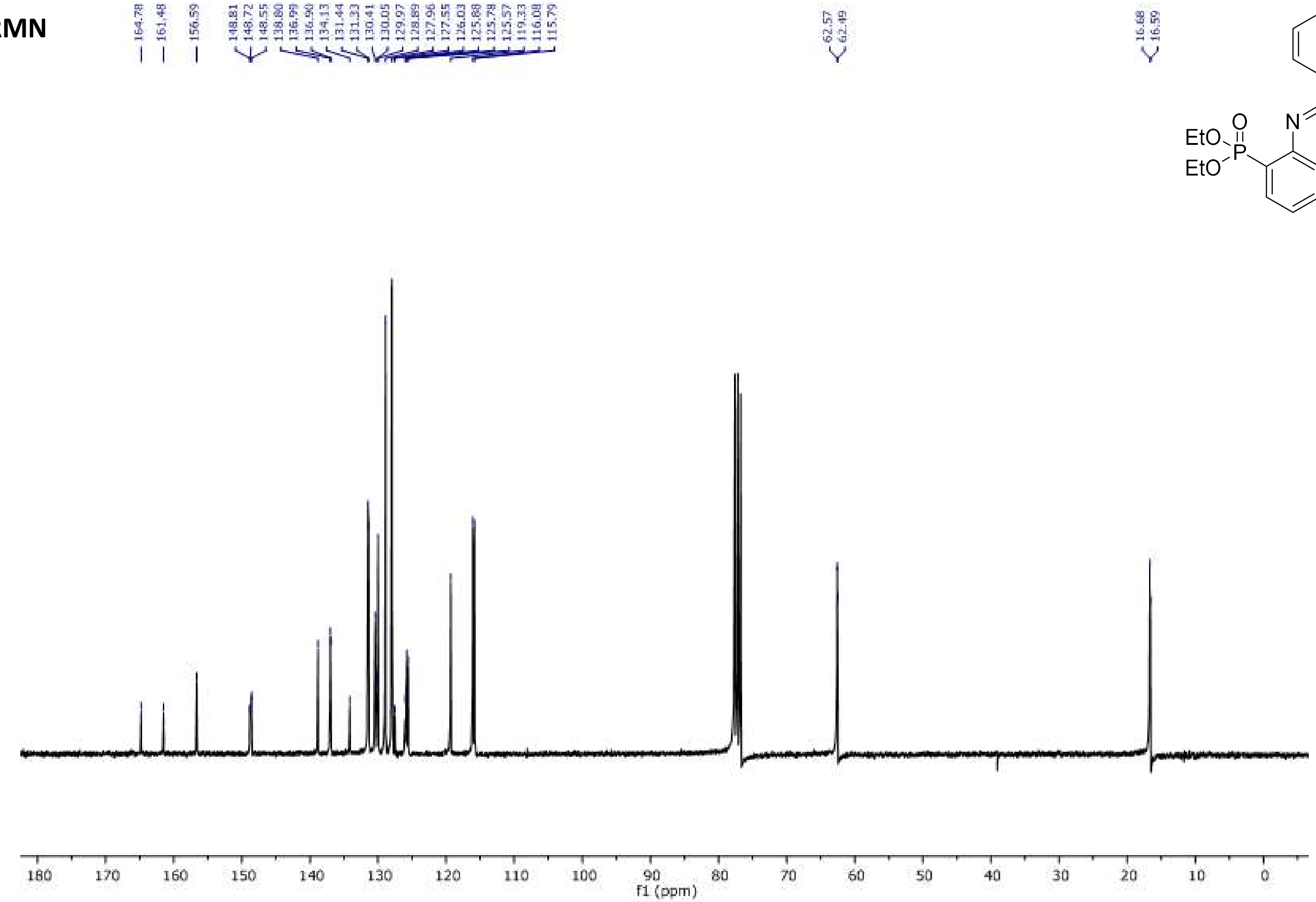
164.78
161.48
156.59
148.81
148.72
148.55
138.80
136.99
136.90
134.13
131.44
131.33
130.41
130.05
129.97
128.89
127.96
127.55
126.03
125.88
125.78
125.57
119.33
116.08
115.79
62.57
62.49
16.68
16.59
f1 (ppm)

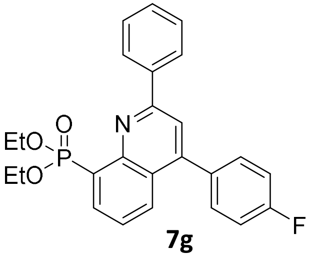
EtO
EtO
O
P
N
F

7g

^{31}P-RMN

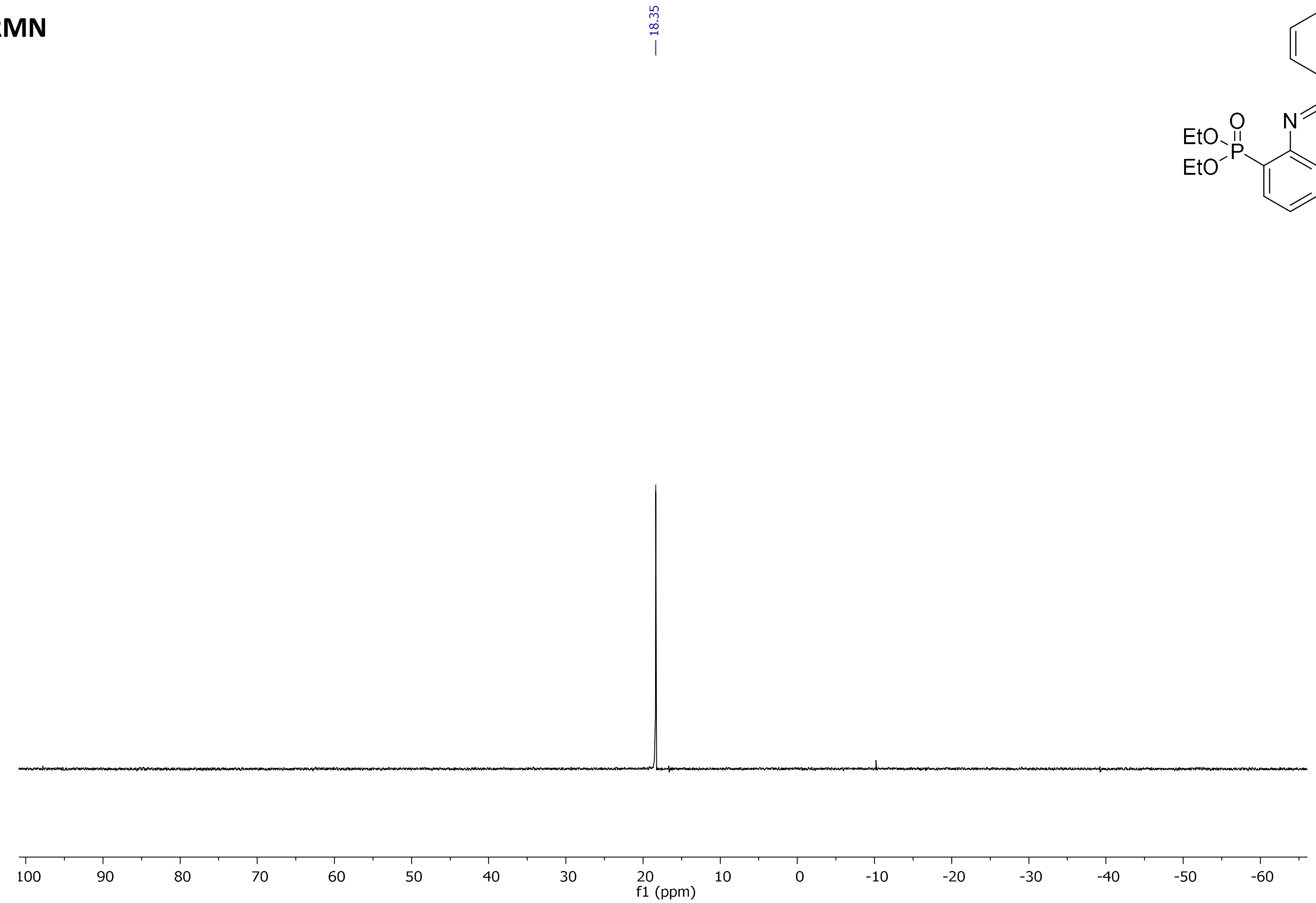

18.35
100
90
80
70
60
50
40
30
20
10
0
-10
-20
-30
-40
-50
-60
f1 (ppm)

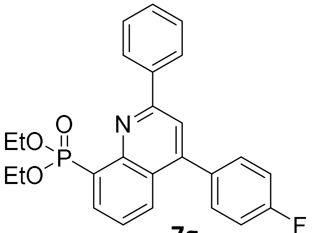

EtO
EtO
O
P
N
F

7g

^{19}F-RMN

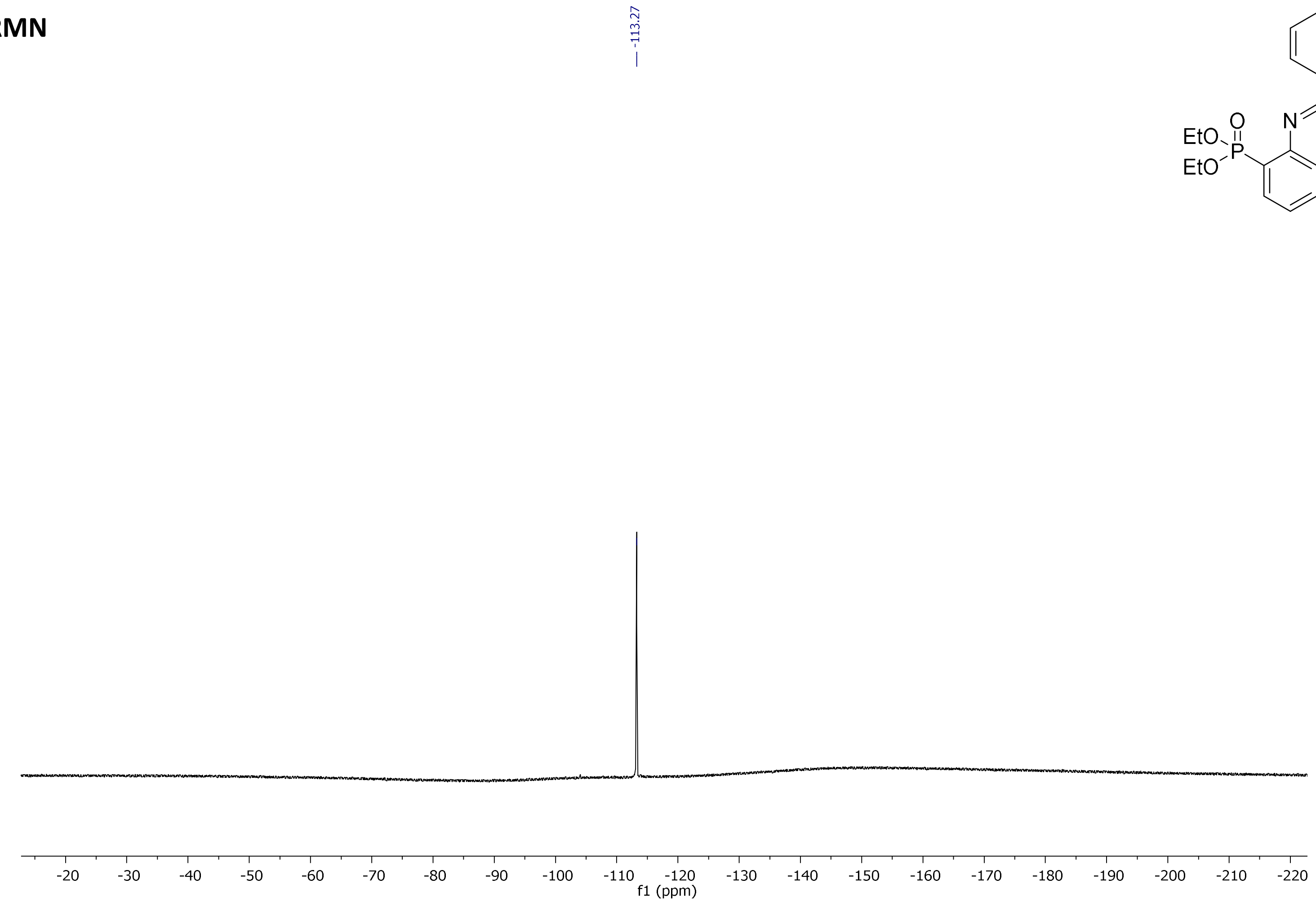

-113.27
-20
-30
-40
-50
-60
-70
-80
-90
-100
-110
-120
-130
-140
-150
-160
-170
-180
-190
-200
-210
-220
f1 (ppm)

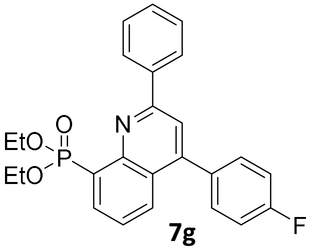

EtO
EtO
O
P
N
F

7g

^{1}H-RMN

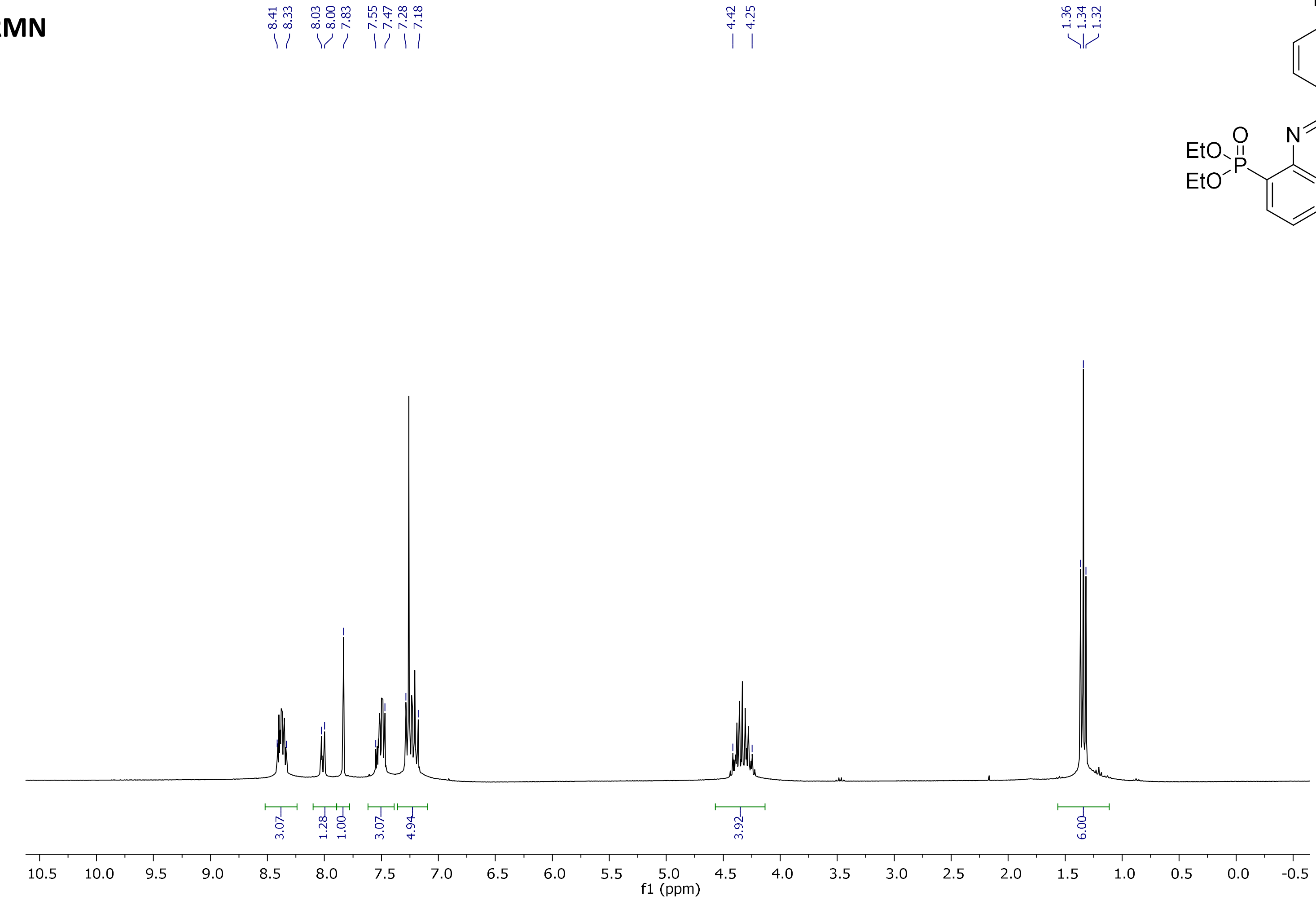

8.41
8.33
8.03
8.00
7.83
7.55
7.47
7.28
7.18
4.42
4.25
1.36
1.34
1.32
3.07
1.28
1.00
3.07
4.94
3.92
6.00
10.5 10.0 9.5 9.0 8.5 8.0 7.5 7.0 6.5 6.0 5.5 5.0 4.5 4.0 3.5 3.0 2.5 2.0 1.5 1.0 0.5 0.0 -0.5
f1 (ppm)

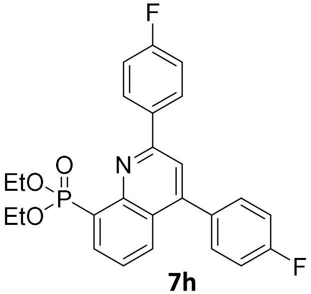

F
N
O
EtO
P
EtO
F

7h

^{13}C-RMN

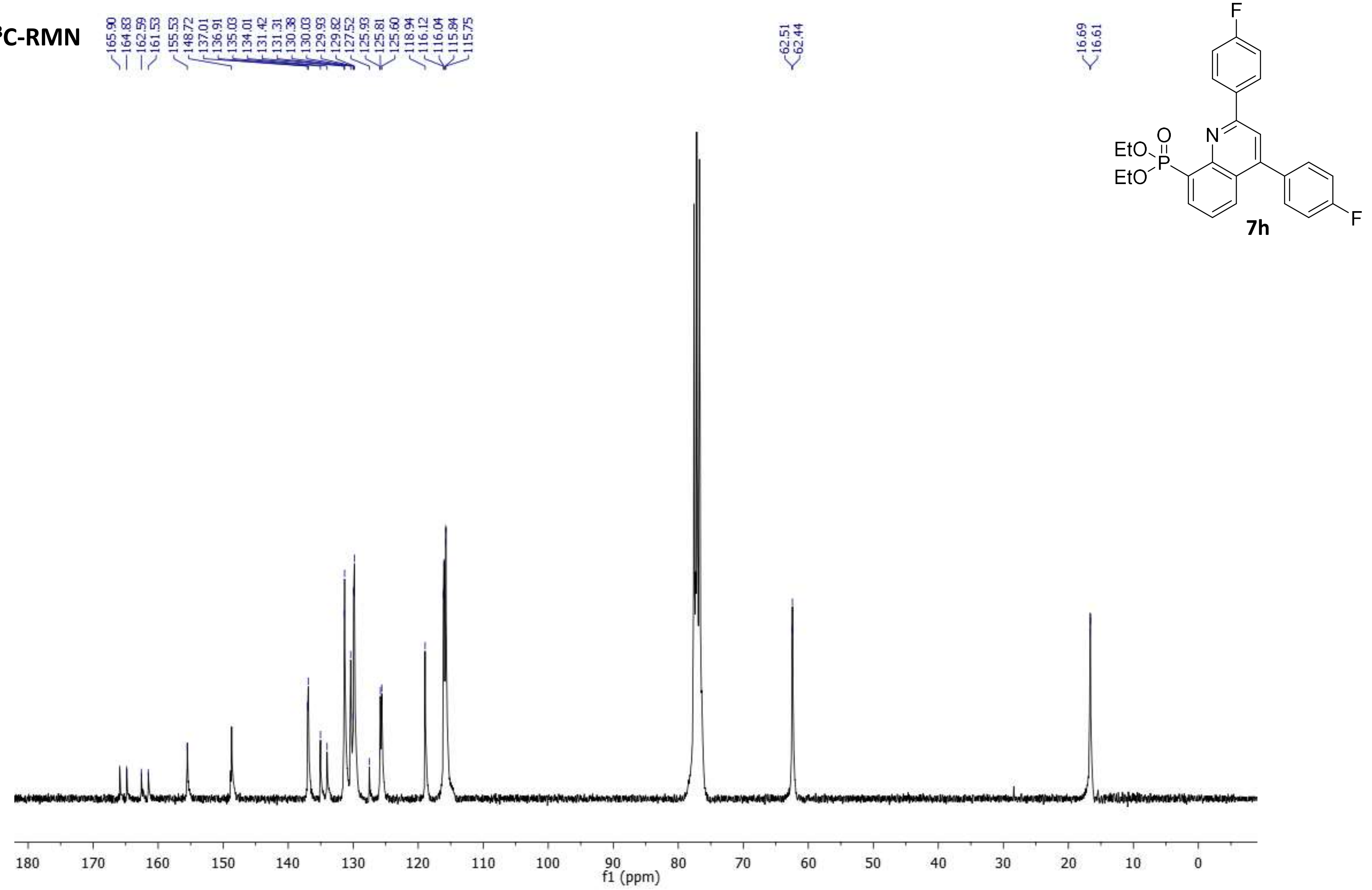
165.90
164.83
162.59
161.53
155.53
148.72
137.01
136.91
135.03
134.01
131.42
131.31
130.38
130.03
129.93
129.82
127.52
125.93
125.81
125.60
118.94
116.12
116.04
115.84
115.75
62.51
62.44
16.69
16.61
180
170
160
150
140
130
120
110
100
90
80
70
60
50
40
30
20
10
0
f1 (ppm)
F
O
N
EtO
P
EtO
F

7h

^{31}P-RMN

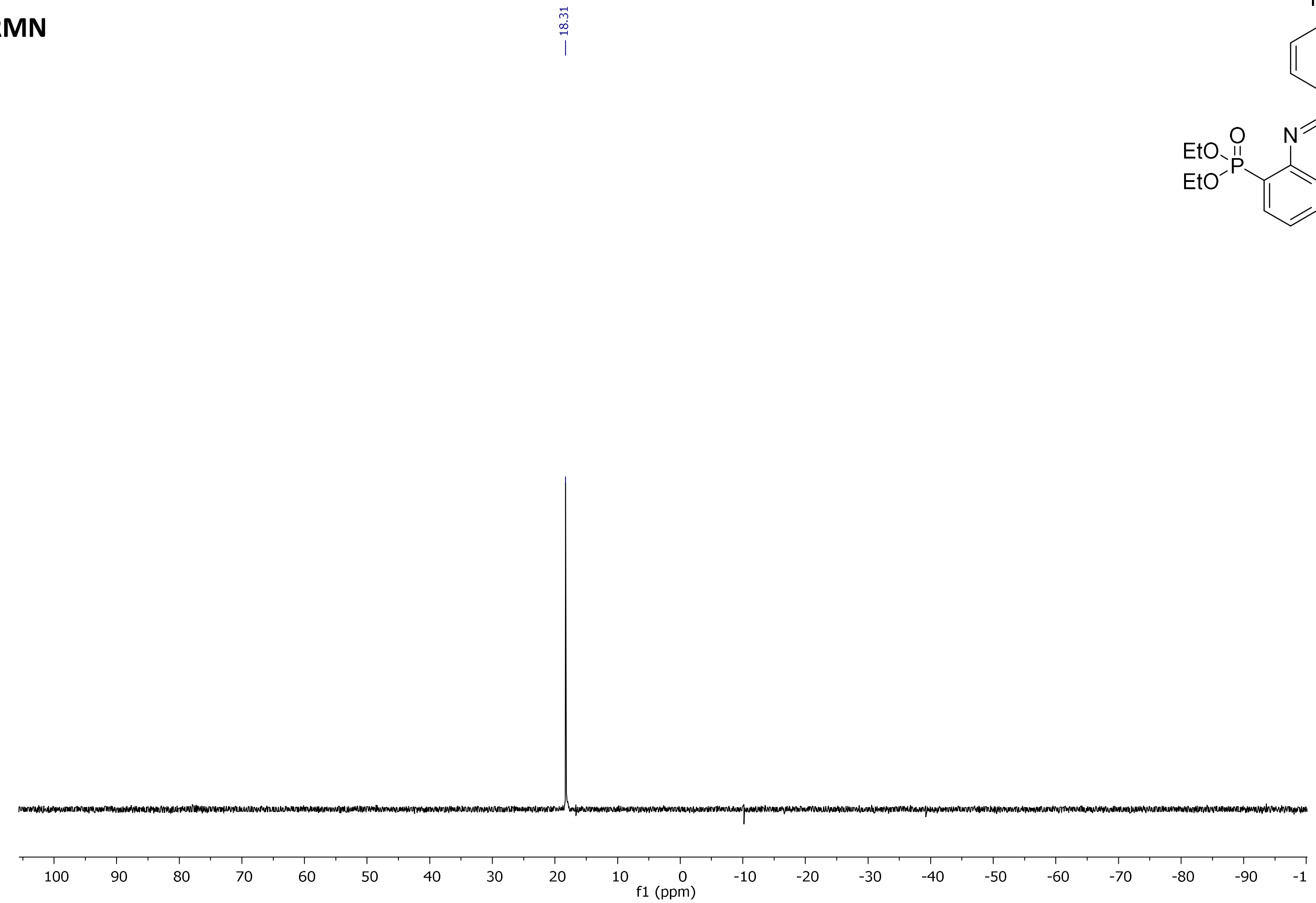

18.31
100
90
80
70
60
50
40
30
20
10
0
-10
-20
-30
-40
-50
-60
-70
-80
-90
-1
f1 (ppm)

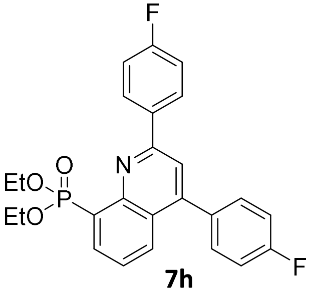

F
EtO
EtO
O
P
N
F

7h

^{19}F-RMN

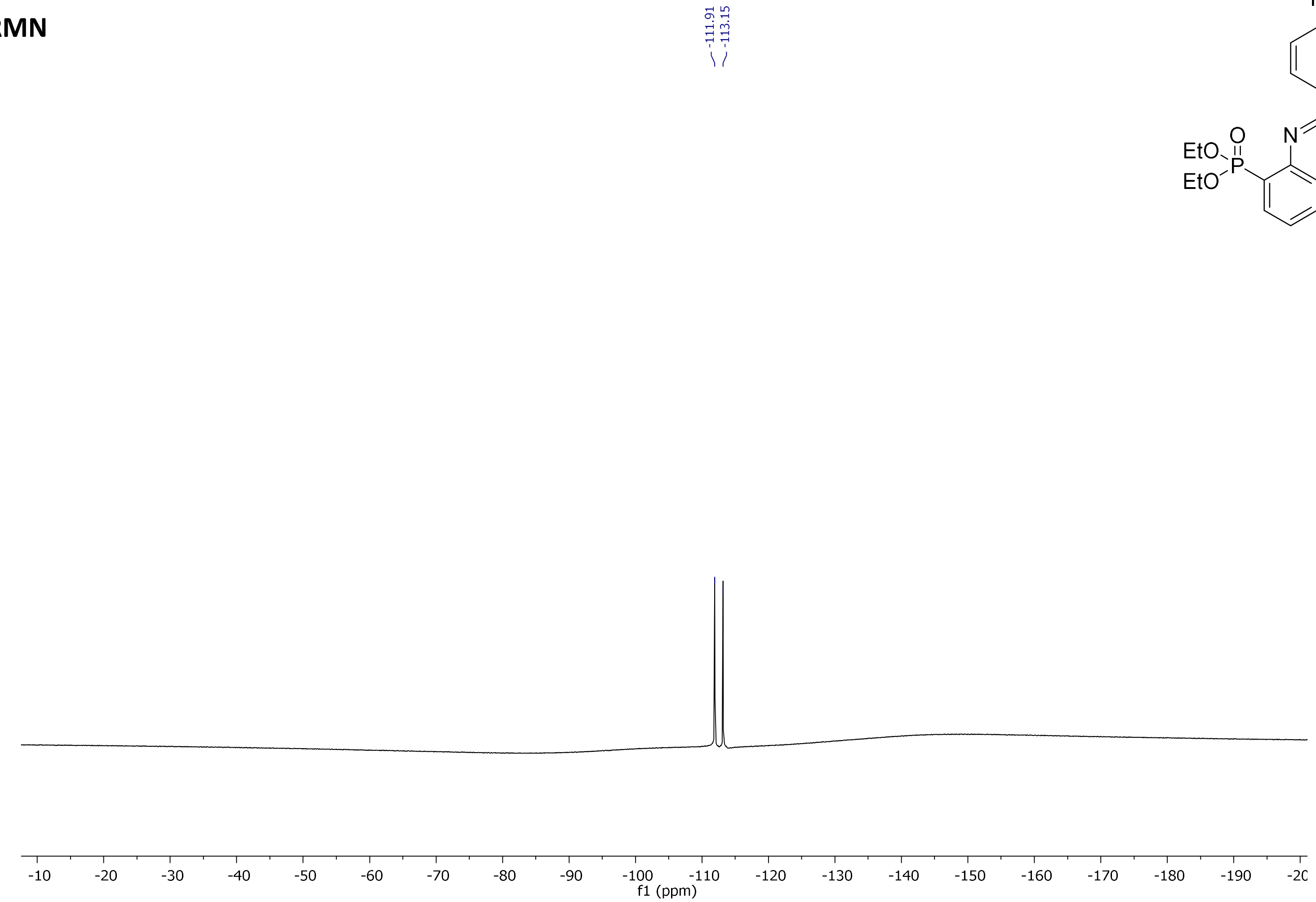

-111.91
-113.15
-10 -20 -30 -40 -50 -60 -70 -80 -90 -100 -110 -120 -130 -140 -150 -160 -170 -180 -190 -20
f1 (ppm)

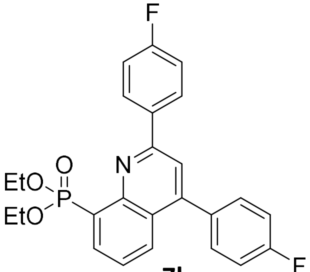

F
EtO
EtO
O
P
N
F

7h

^{1}H-RMN

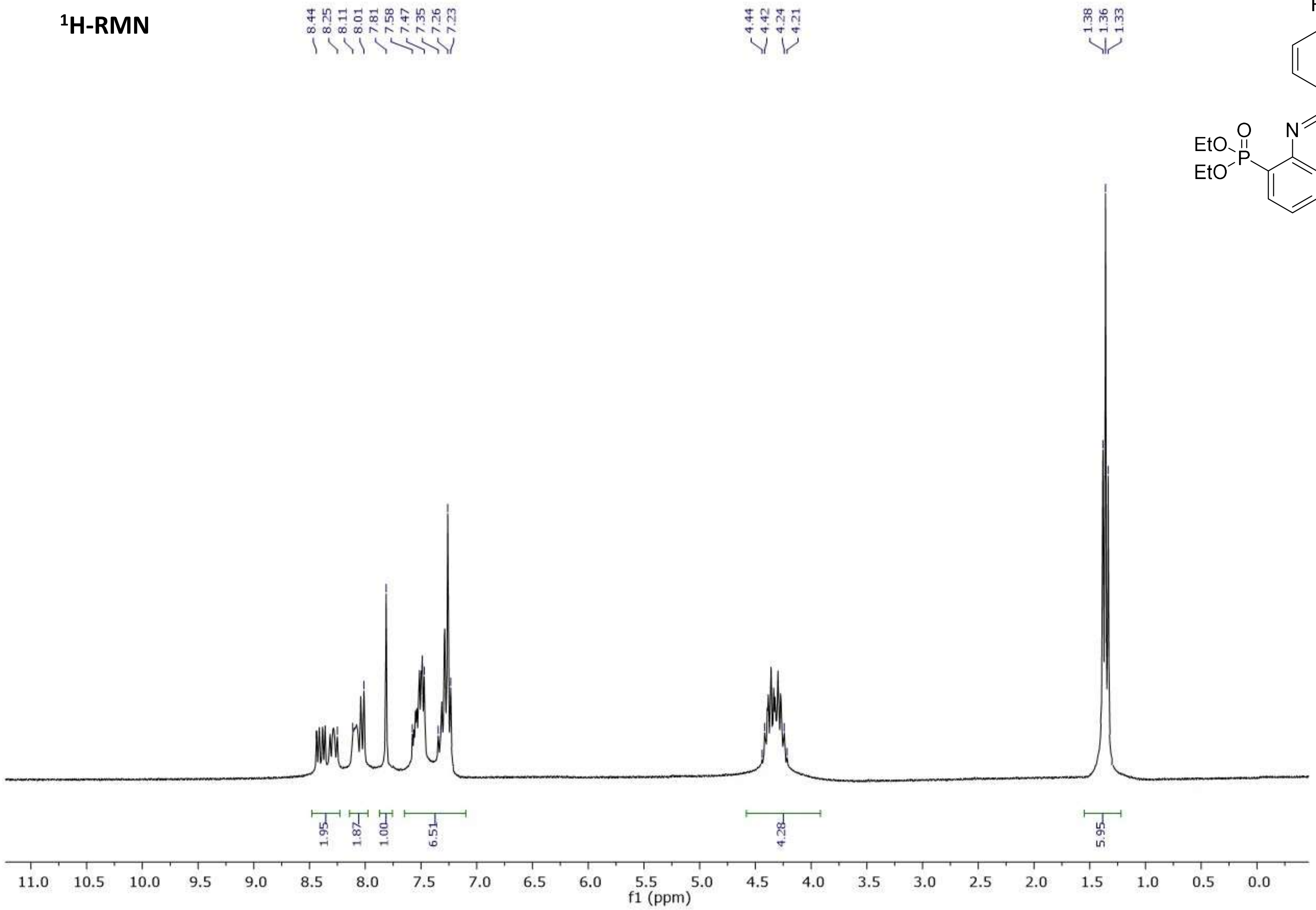

8.44
8.25
8.11
8.01
7.81
7.58
7.47
7.35
7.26
7.23
4.44
4.42
4.24
4.21
1.38
1.36
1.33
1.95
1.87
1.00
6.51
4.28
5.95
11.0 10.5 10.0 9.5 9.0 8.5 8.0 7.5 7.0 6.5 6.0 5.5 5.0 4.5 4.0 3.5 3.0 2.5 2.0 1.5 1.0 0.5 0.0
f1 (ppm)

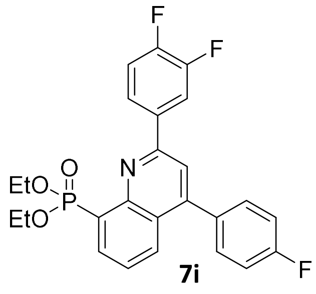

F
F
EtO
O
P
EtO
N
F

7i

^{13}C-RMN

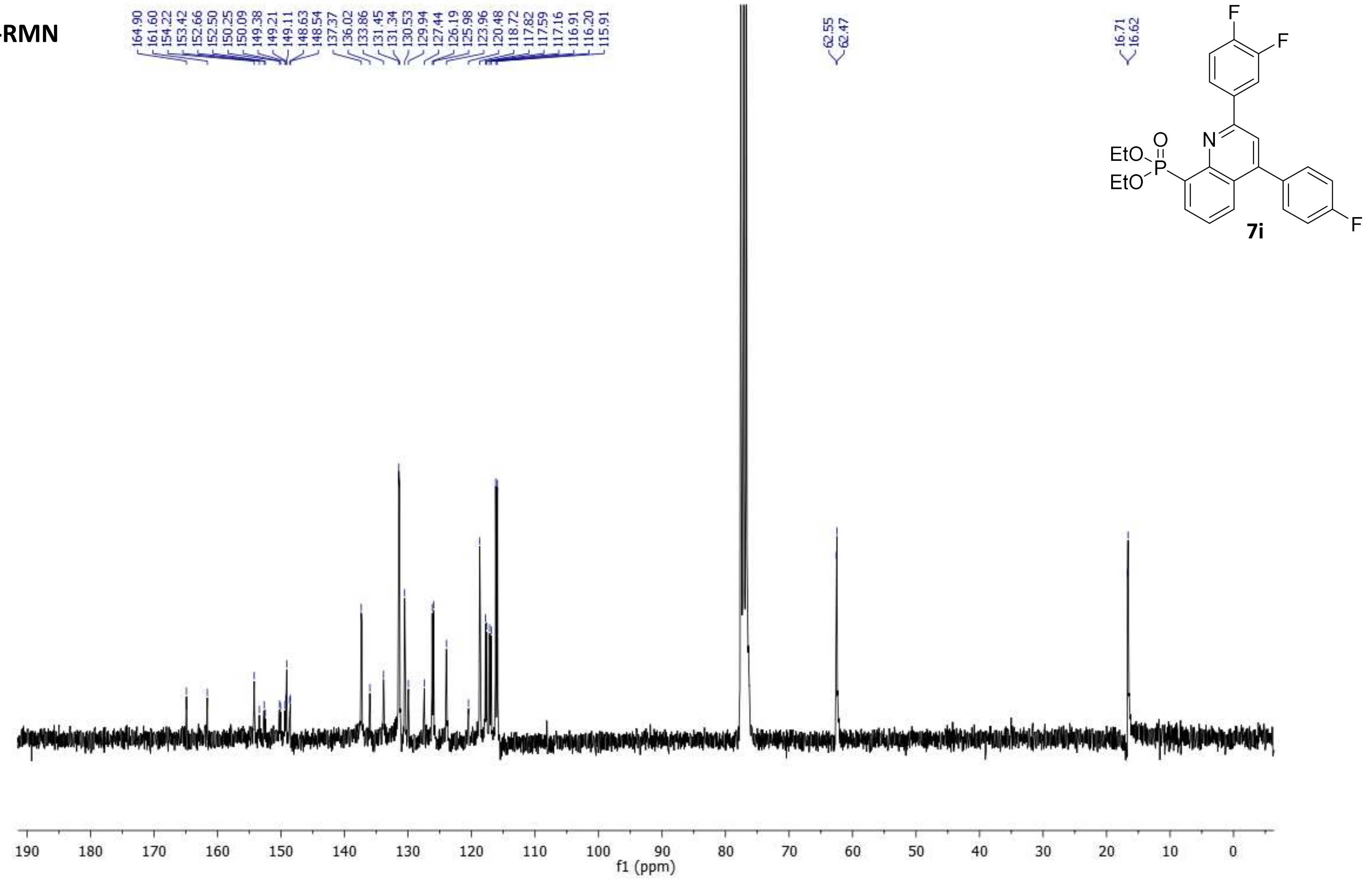

164.90
161.60
154.22
153.42
152.66
152.50
150.25
150.09
149.38
149.21
149.11
148.63
148.54
137.37
136.02
133.86
131.45
131.34
130.53
129.94
127.44
126.19
125.98
123.96
120.48
118.72
117.82
117.59
117.16
116.91
116.20
115.91
62.55
62.47
16.71
16.62
190 180 170 160 150 140 130 120 110 100 90 80 70 60 50 40 30 20 10 0
f1 (ppm)
F
F
N
EtO
O
P
EtO
F

7i

^{31}P-RMN

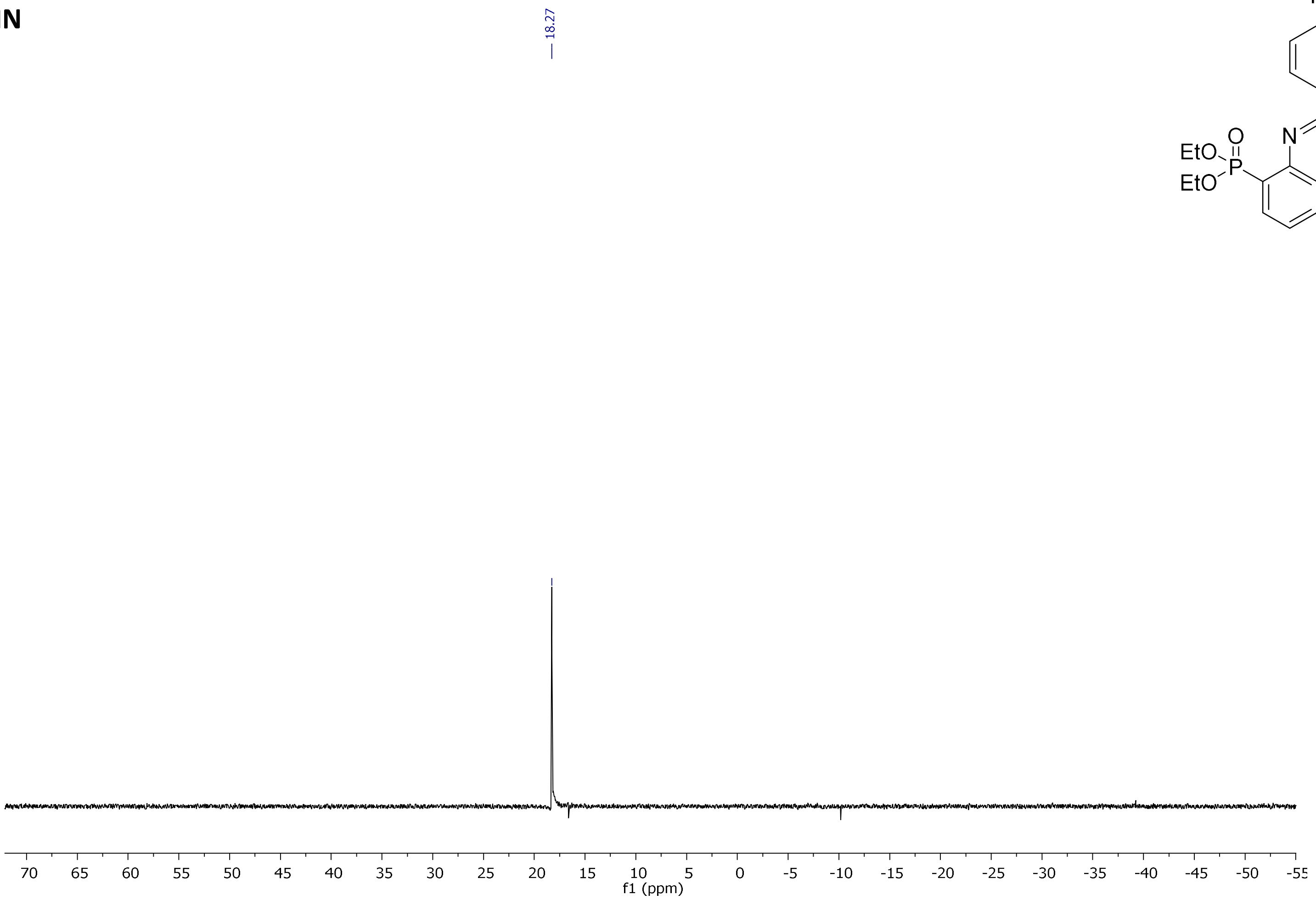

18.27
70
65
60
55
50
45
40
35
30
25
20
15
10
5
0
-5
-10
-15
-20
-25
-30
-35
-40
-45
-50
-55
f1 (ppm)

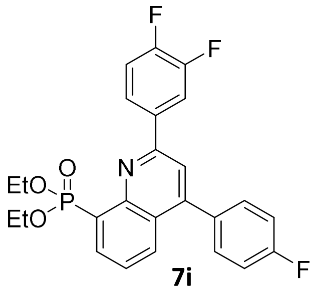

F
F
EtO
O
N
P
EtO
F

7i

^{19}F-RMN

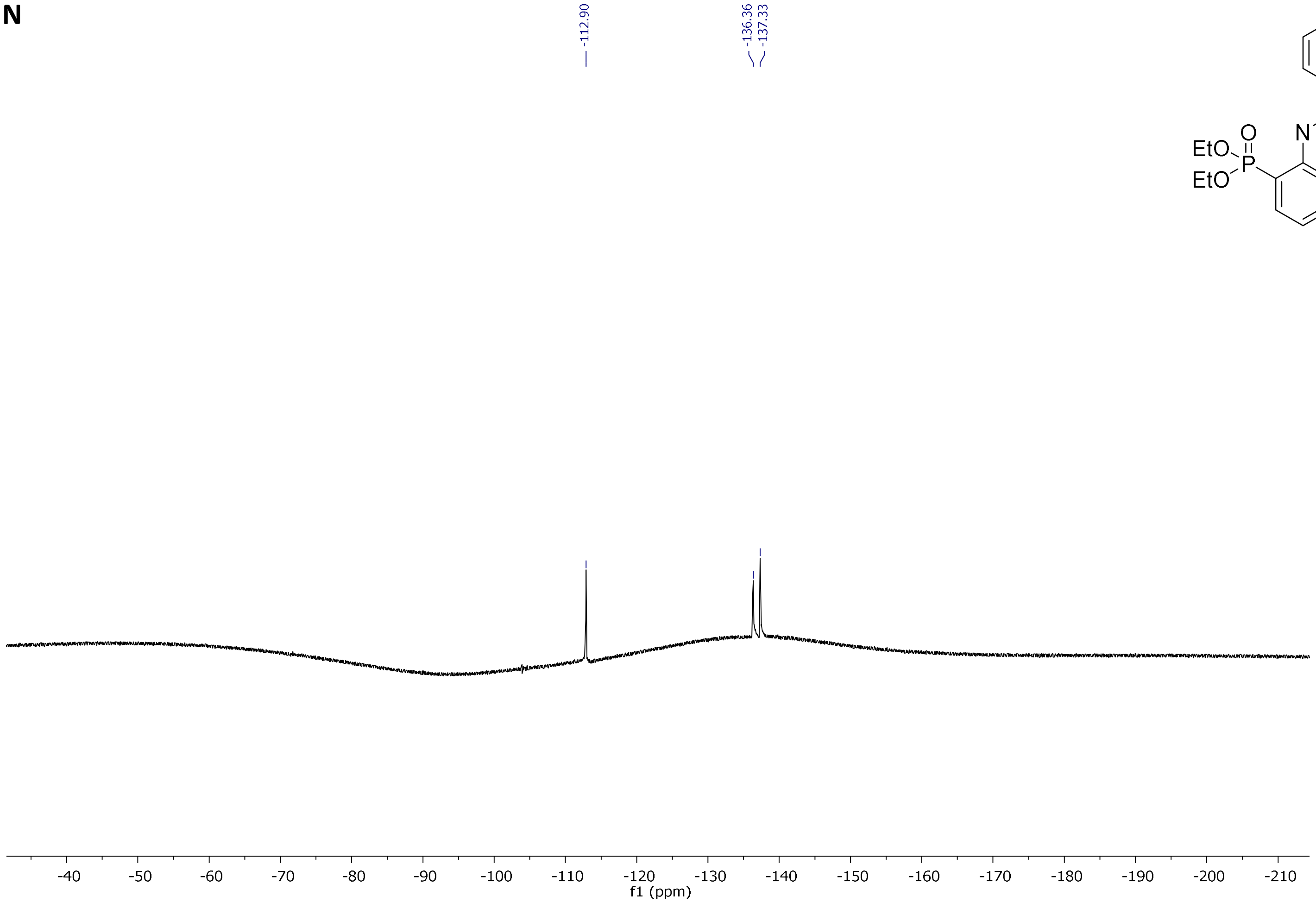

-112.90
-136.36
-137.33
-40 -50 -60 -70 -80 -90 -100 -110 -120 -130 -140 -150 -160 -170 -180 -190 -200 -210
f1 (ppm)

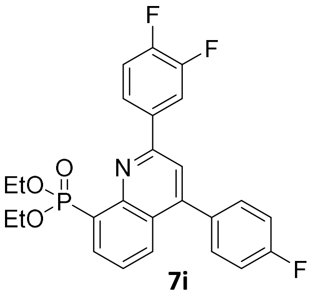

F
F
O
N
EtO
P
EtO
F

7i

^{1}H-RMN

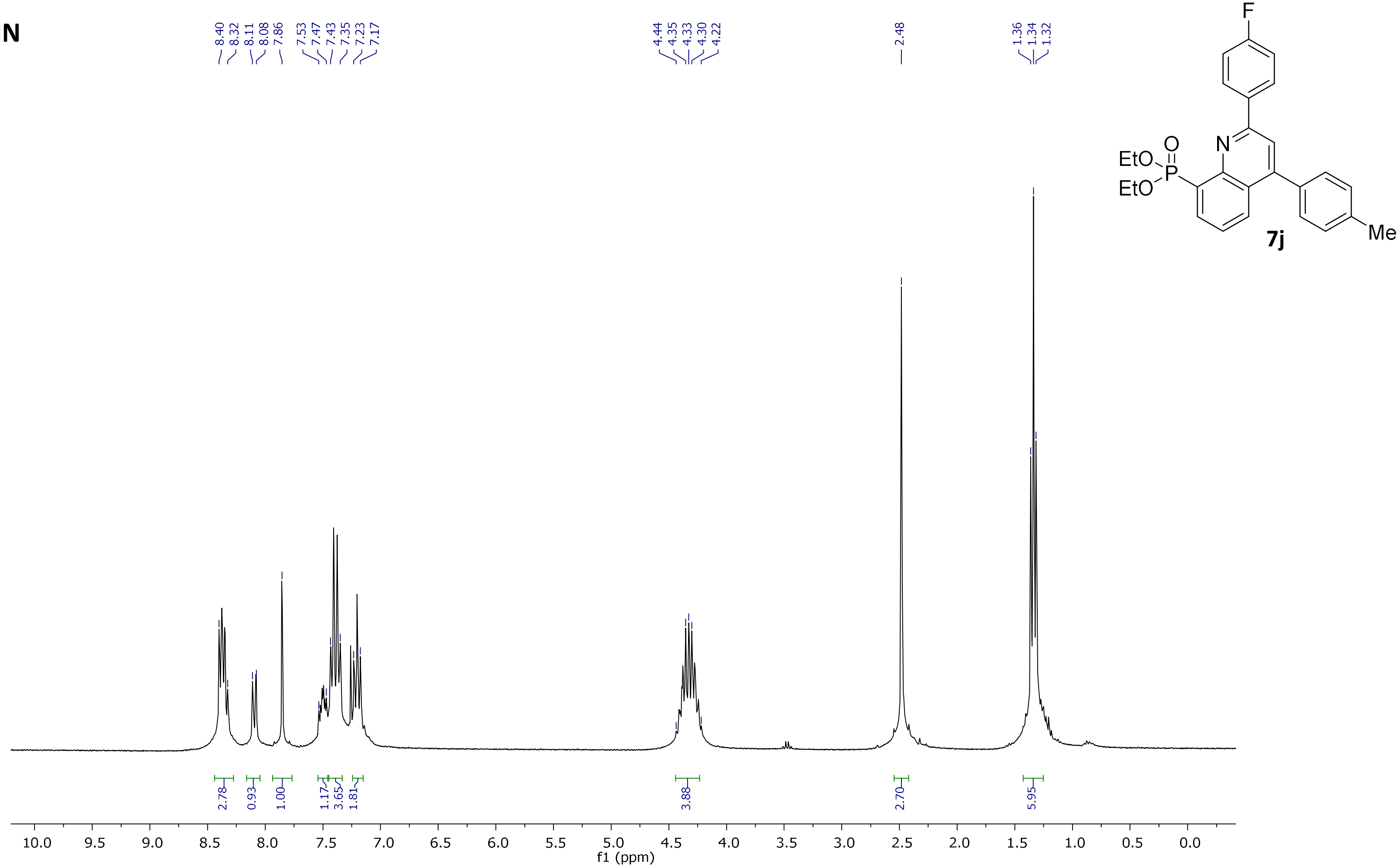

8.40
8.32
8.11
8.08
7.86
7.53
7.47
7.43
7.35
7.23
7.17
4.44
4.35
4.33
4.30
4.22
2.48
1.36
1.34
1.32
2.78
0.93
1.00
1.17
3.65
1.81
3.88
2.70
5.95
10.0
9.5
9.0
8.5
8.0
7.5
7.0
6.5
6.0
5.5
5.0
4.5
4.0
3.5
3.0
2.5
2.0
1.5
1.0
0.5
0.0
f1 (ppm)
F
N
EtO
EtO
O
P
Me

7j

^{13}C-RMN

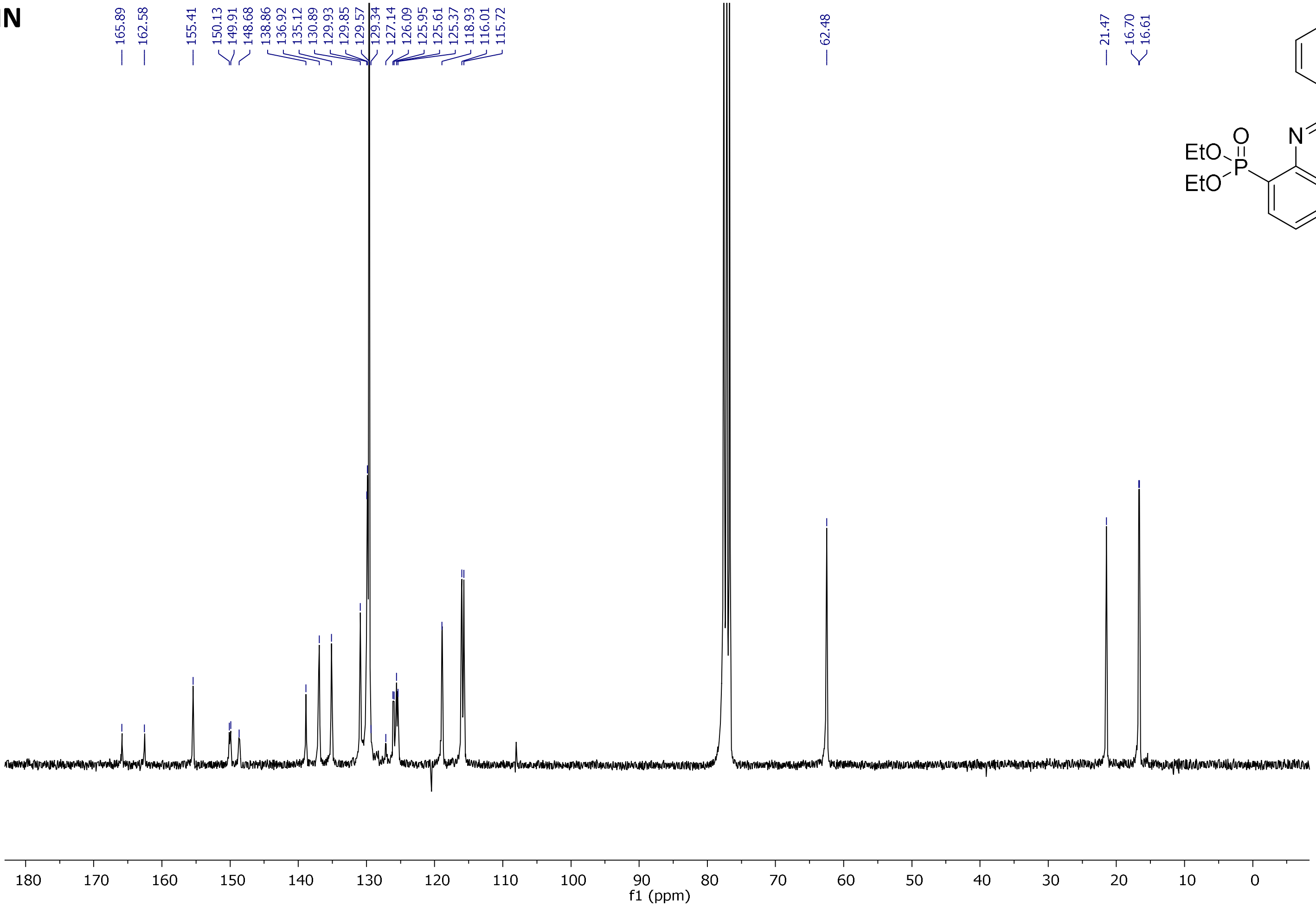

165.89
162.58
155.41
150.13
149.91
148.68
138.86
136.92
135.12
130.89
129.93
129.85
129.57
129.34
127.14
126.09
125.95
125.61
125.37
118.93
116.01
115.72
62.48
21.47
16.70
16.61
180 170 160 150 140 130 120 110 100 90 80 70 60 50 40 30 20 10 0
f1 (ppm)

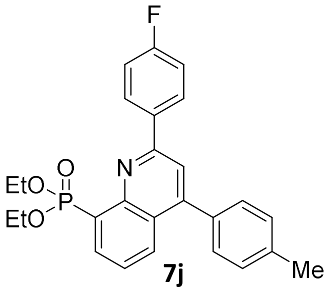

F
N
O
EtO
P
EtO
Me

7j

^{31}P-RMN

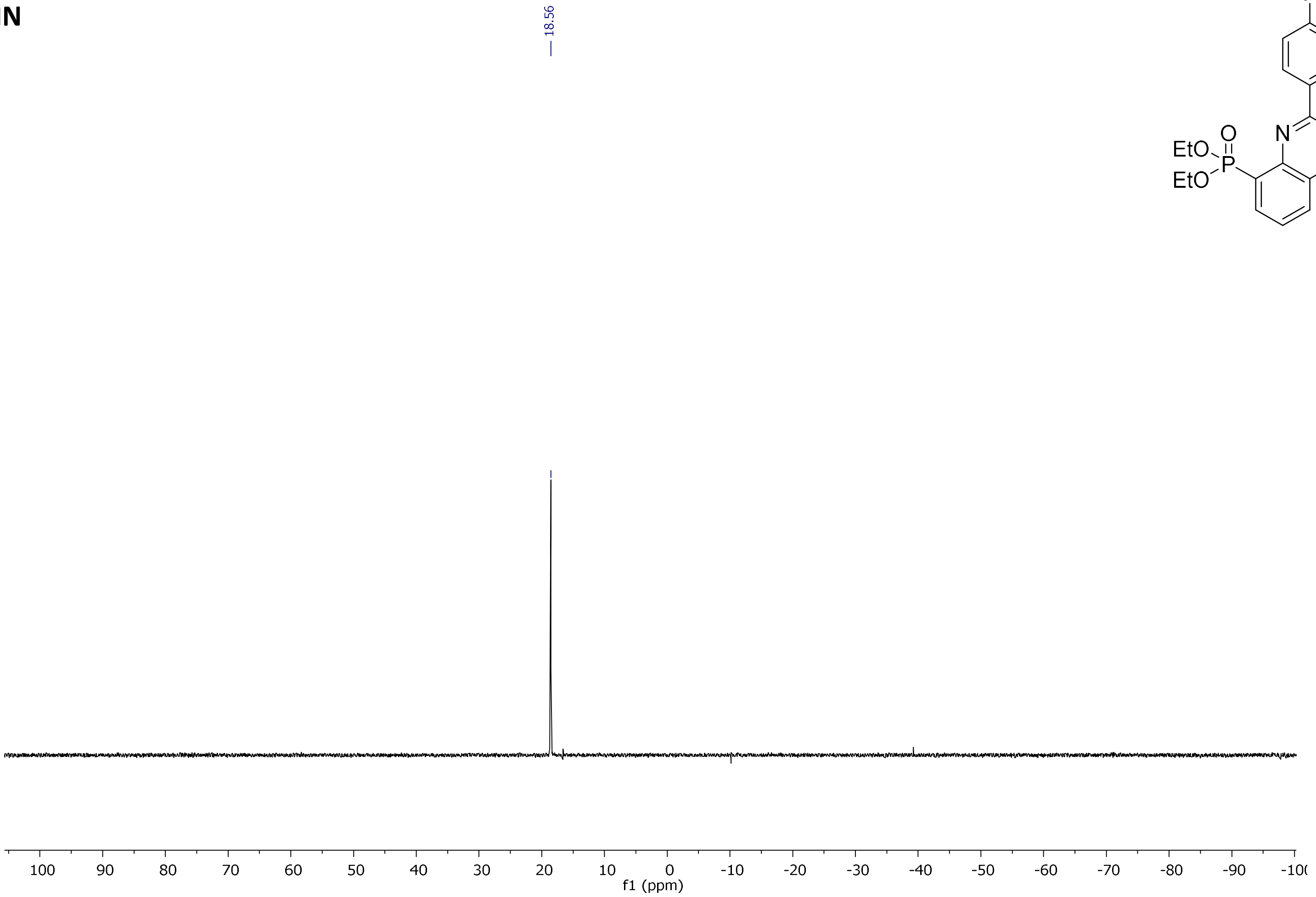

18.56
100
90
80
70
60
50
40
30
20
10
0
-10
-20
-30
-40
-50
-60
-70
-80
-90
f1 (ppm)

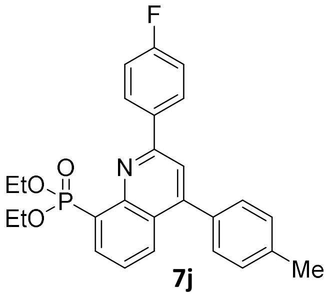

F
N
O
P
EtO
EtO
Me

7j

¹⁹F-RMN

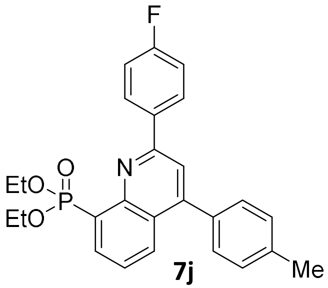

F
N
EtO
O
P
EtO
Me

7j

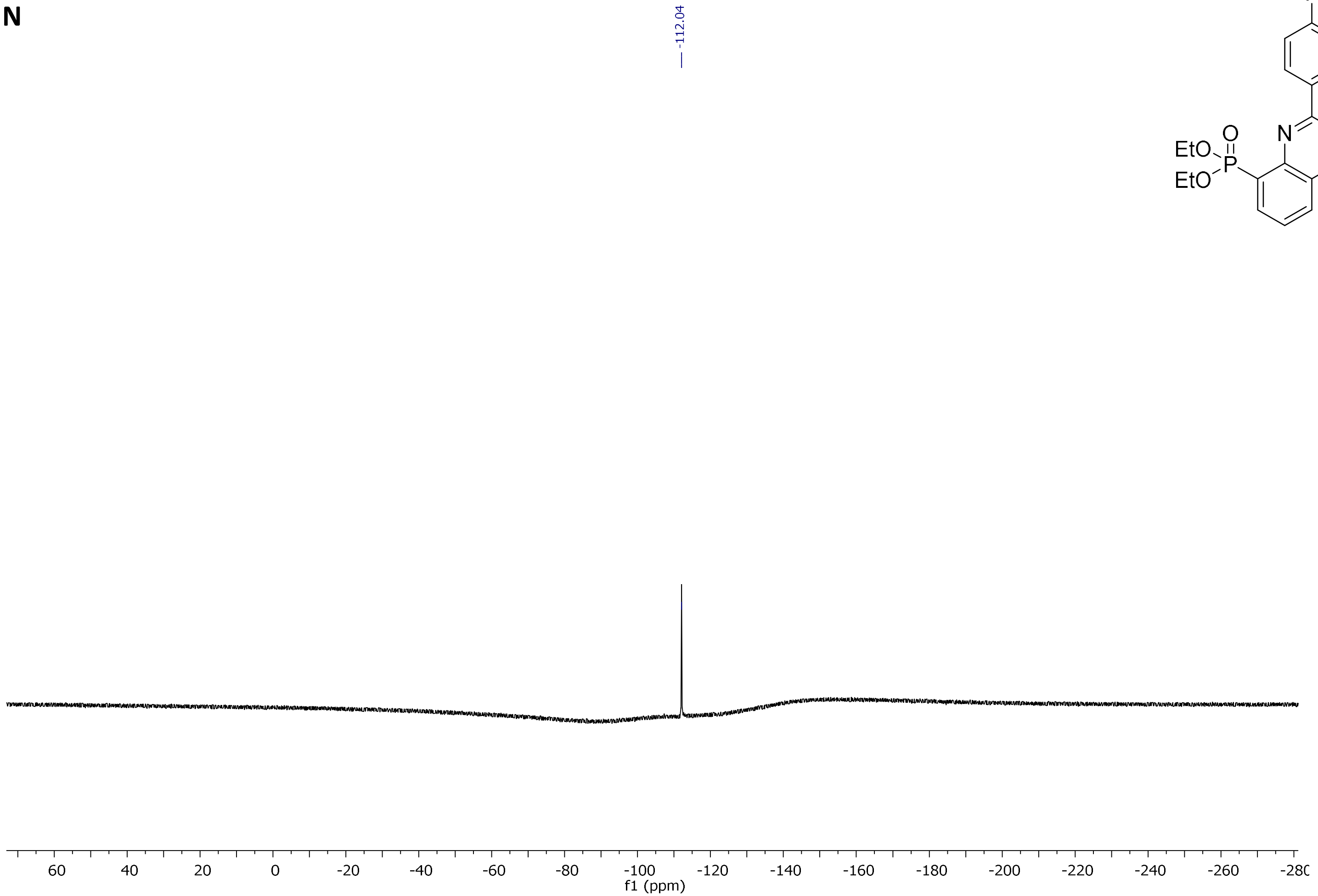

-112.04
60
40
20
0
-20
-40
-60
-80
-100
-120
-140
-160
-180
-200
-220
-240
-260
-280
f1 (ppm)

^{1}H-RMN

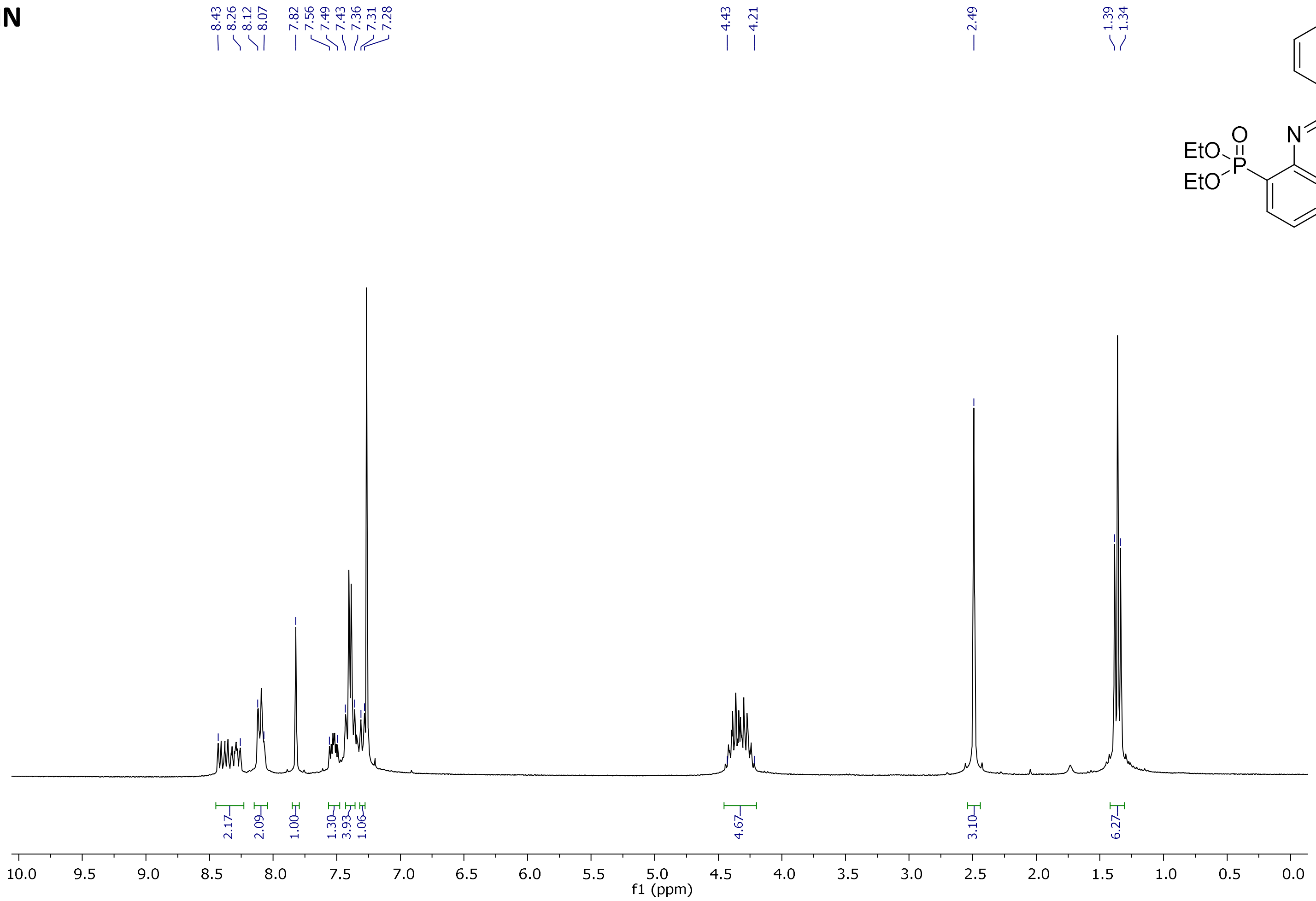
8.43
8.26
8.12
8.07
7.82
7.56
7.49
7.43
7.36
7.31
7.28
4.43
4.21
2.49
1.39
1.34
2.17
2.09
1.00
1.30
3.93
1.06
4.67
3.10
6.27
10.0 9.5 9.0 8.5 8.0 7.5 7.0 6.5 6.0 5.5 5.0 4.5 4.0 3.5 3.0 2.5 2.0 1.5 1.0 0.5 0.0
f1 (ppm)

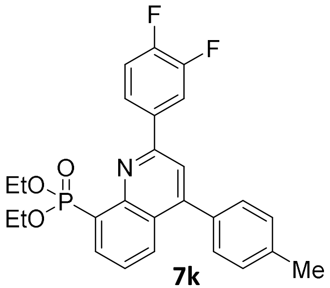
F
F
N
O
EtO
EtO
P
Me

7k

^{13}C-RMN

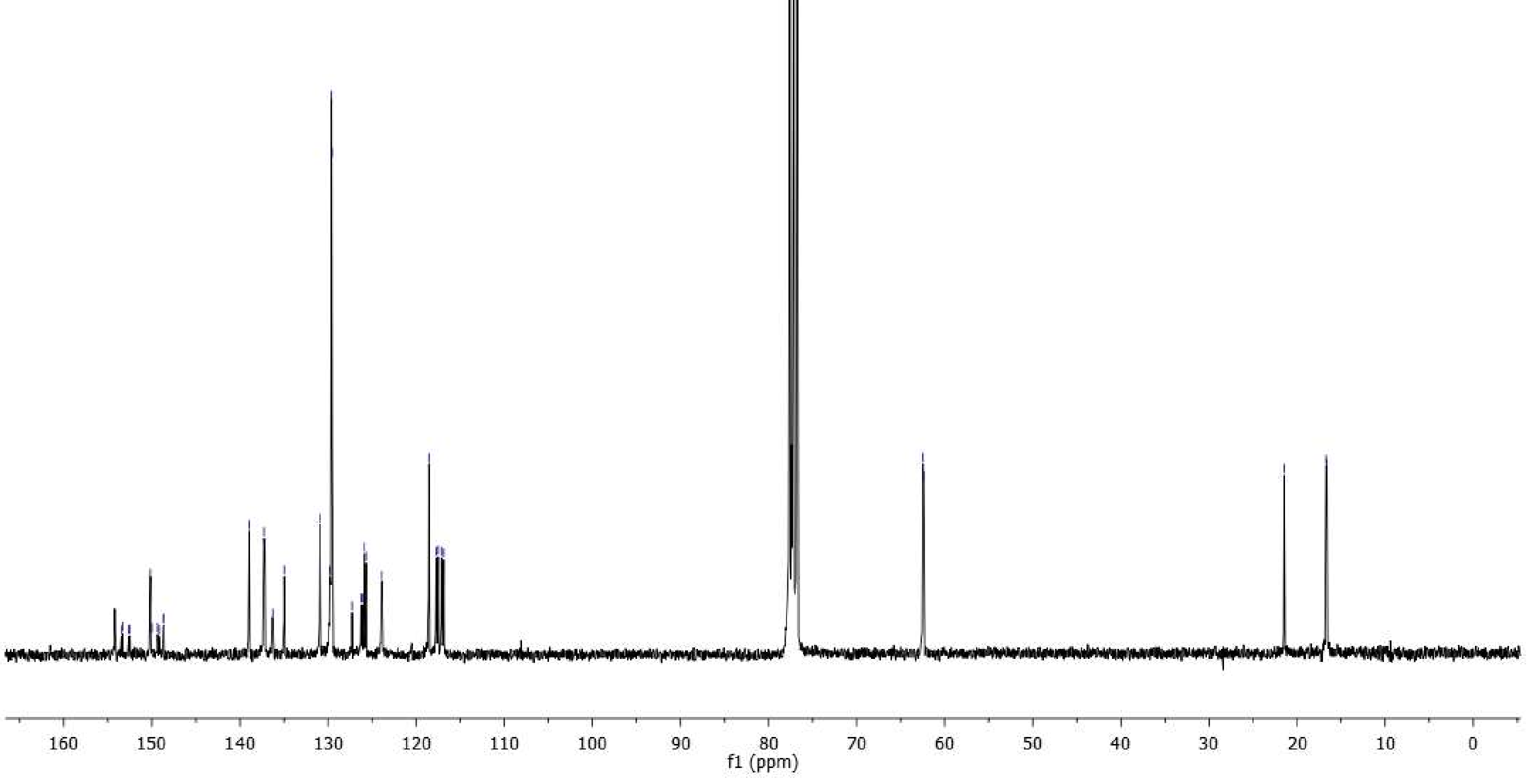
153.47
153.31
152.62
152.46
150.17
149.99
149.34
149.18
148.70
148.61
138.96
137.27
137.17
136.27
134.98
130.92
129.78
129.60
129.54
127.28
126.27
126.13
125.89
125.67
123.92
118.55
117.73
117.50
117.11
116.86
62.48
62.40
21.47
16.70
16.62
160
150
140
130
120
110
100
90
80
70
60
50
40
30
20
10
0
f1 (ppm)

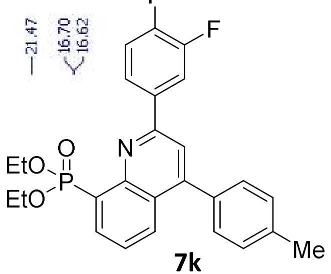
F
F
N
EtO
EtO
O
P
Me

7k

^{31}P-RMN

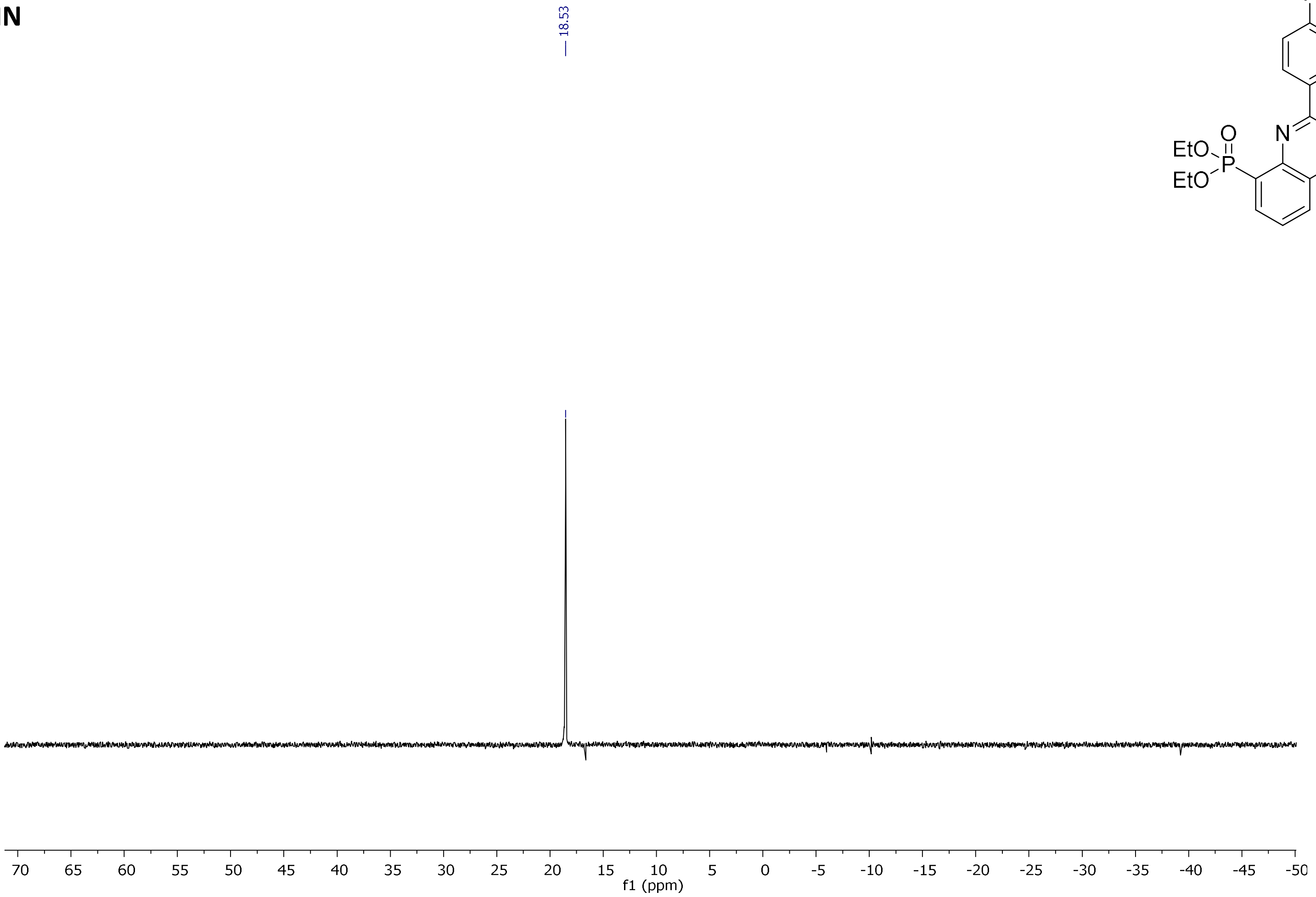

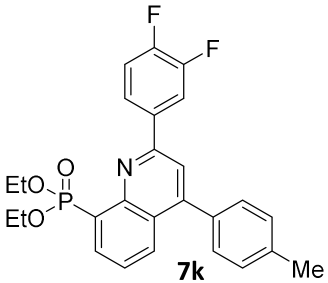

7k

^{19}F-RMN

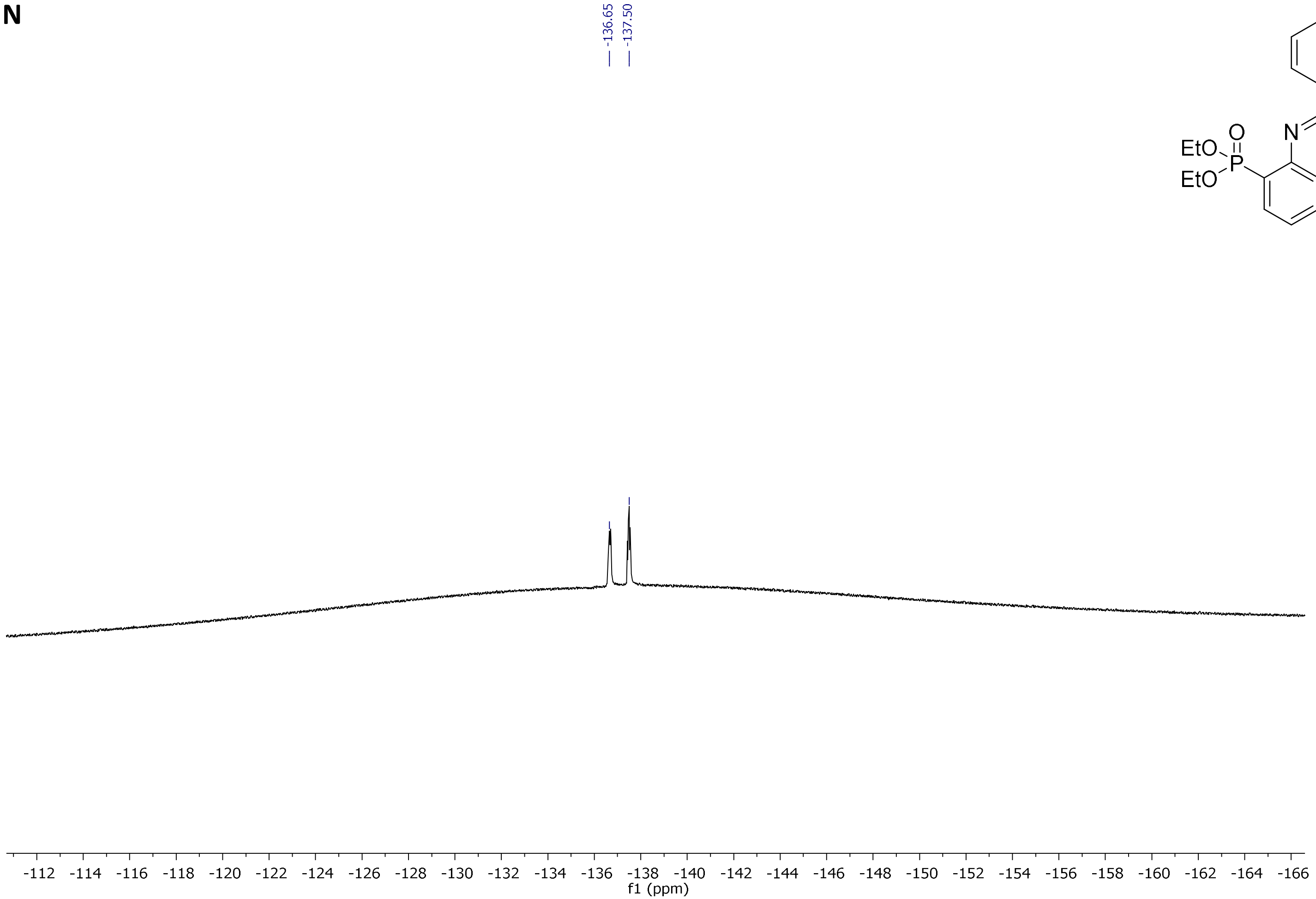

-136.65
-137.50
-112 -114 -116 -118 -120 -122 -124 -126 -128 -130 -132 -134 -136 -138 -140 -142 -144 -146 -148 -150 -152 -154 -156 -158 -160 -162 -164 -166
f1 (ppm)

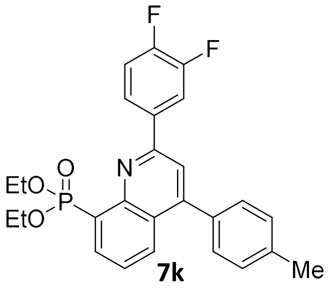

F
F
N
O
EtO
P
EtO
Me

7k

^{1}H-RMN

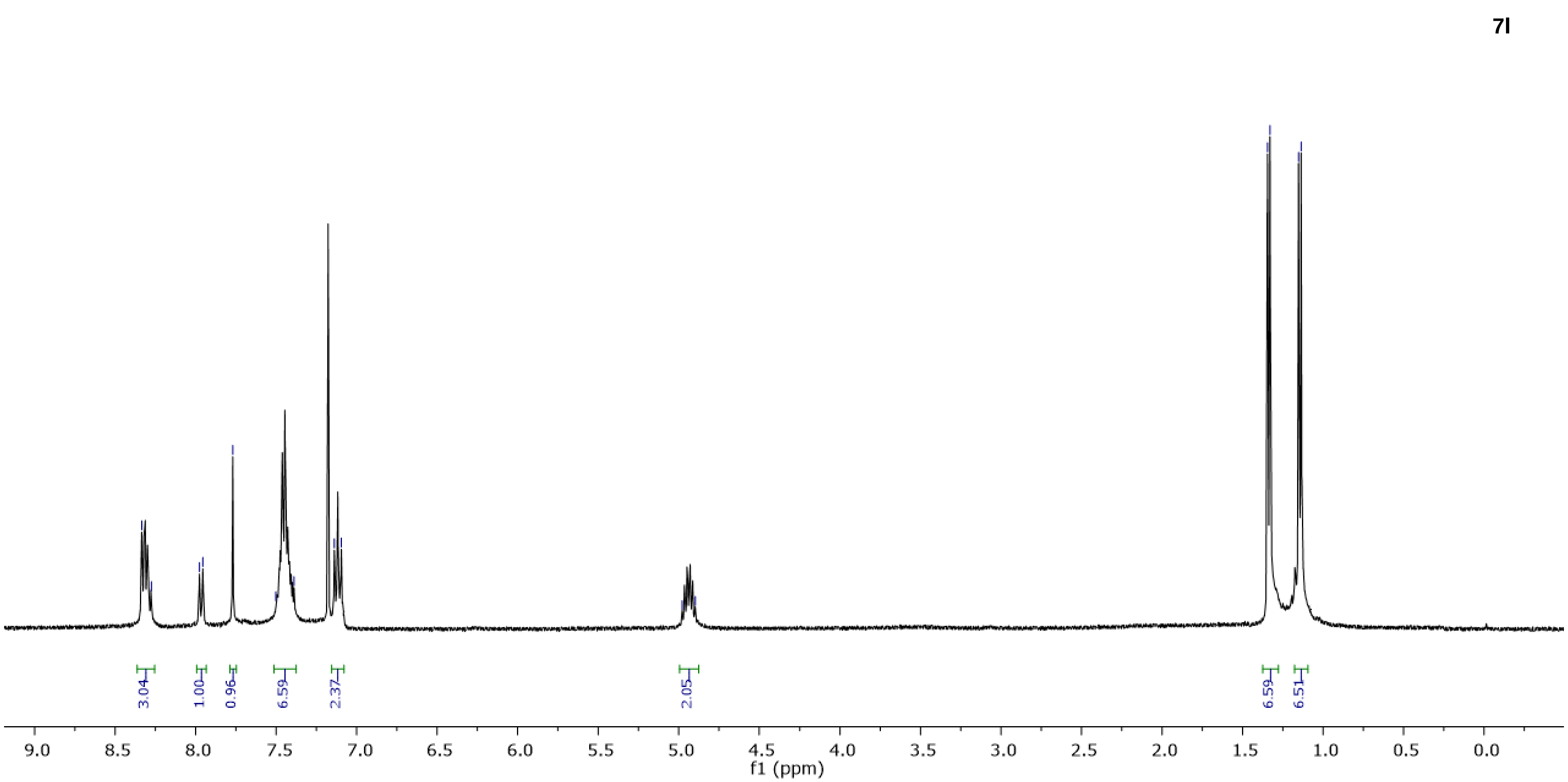

8.33
8.27
7.98
7.96
7.77
7.50
7.39
7.14
7.10
4.98
4.90
1.34
1.33
1.15
1.14
3.04
1.00
0.96
6.59
2.37
2.05
6.59
6.51
9.0 8.5 8.0 7.5 7.0 6.5 6.0 5.5 5.0 4.5 4.0 3.5 3.0 2.5 2.0 1.5 1.0 0.5 0.0
f1 (ppm)

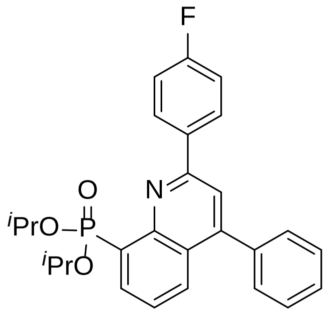

F
O
N
^{i}PrO–P
^{i}PrO

7l

^{13}C-RMN

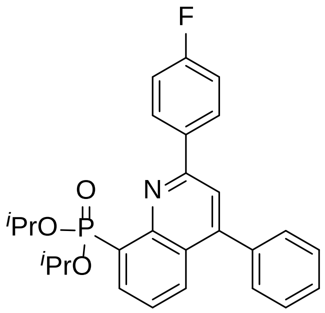
F
O
N
iPrO–P
iPrO

7l

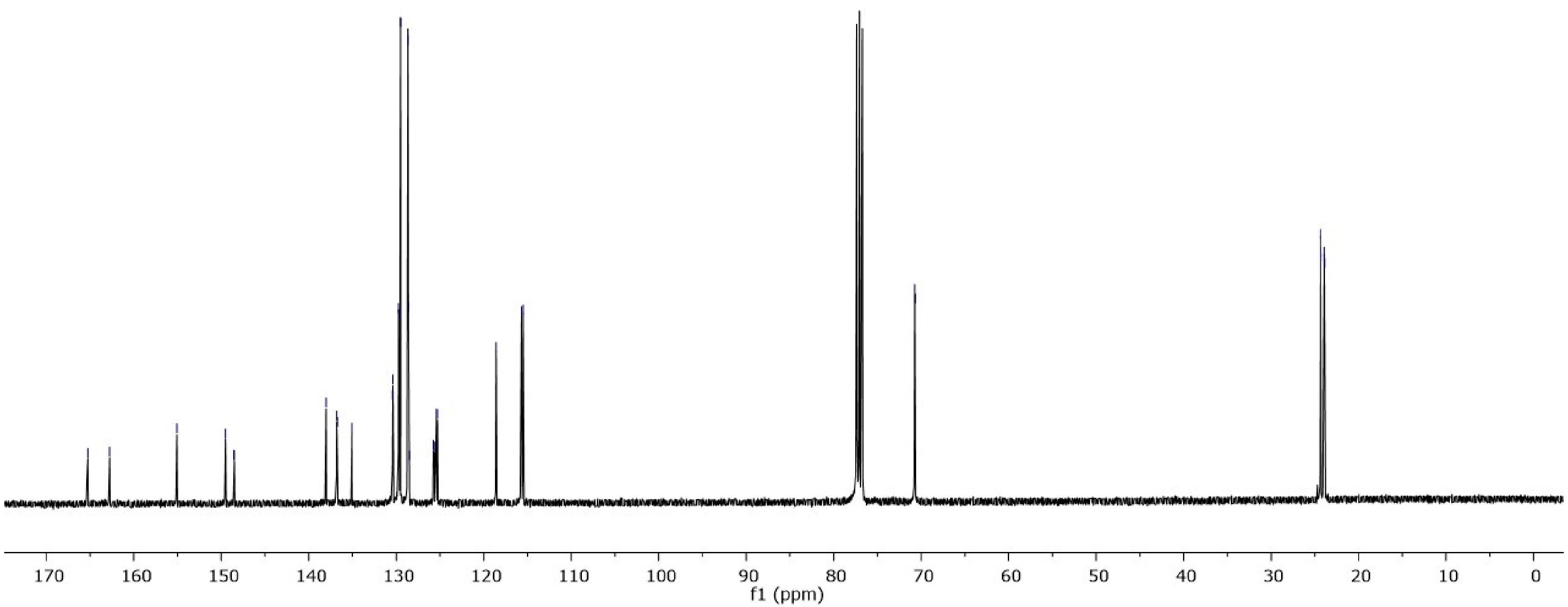
165.25
162.77
155.08
149.53
148.58
148.51
138.01
136.80
136.73
135.08
130.41
130.38
129.75
129.67
129.49
128.67
128.60
128.49
125.76
125.65
125.43
125.27
118.57
115.70
115.49
70.72
70.66
24.34
24.31
23.92
23.87
170 160 150 140 130 120 110 100 90 80 70 60 50 40 30 20 10 0
f1 (ppm)

^{31}P-RMN

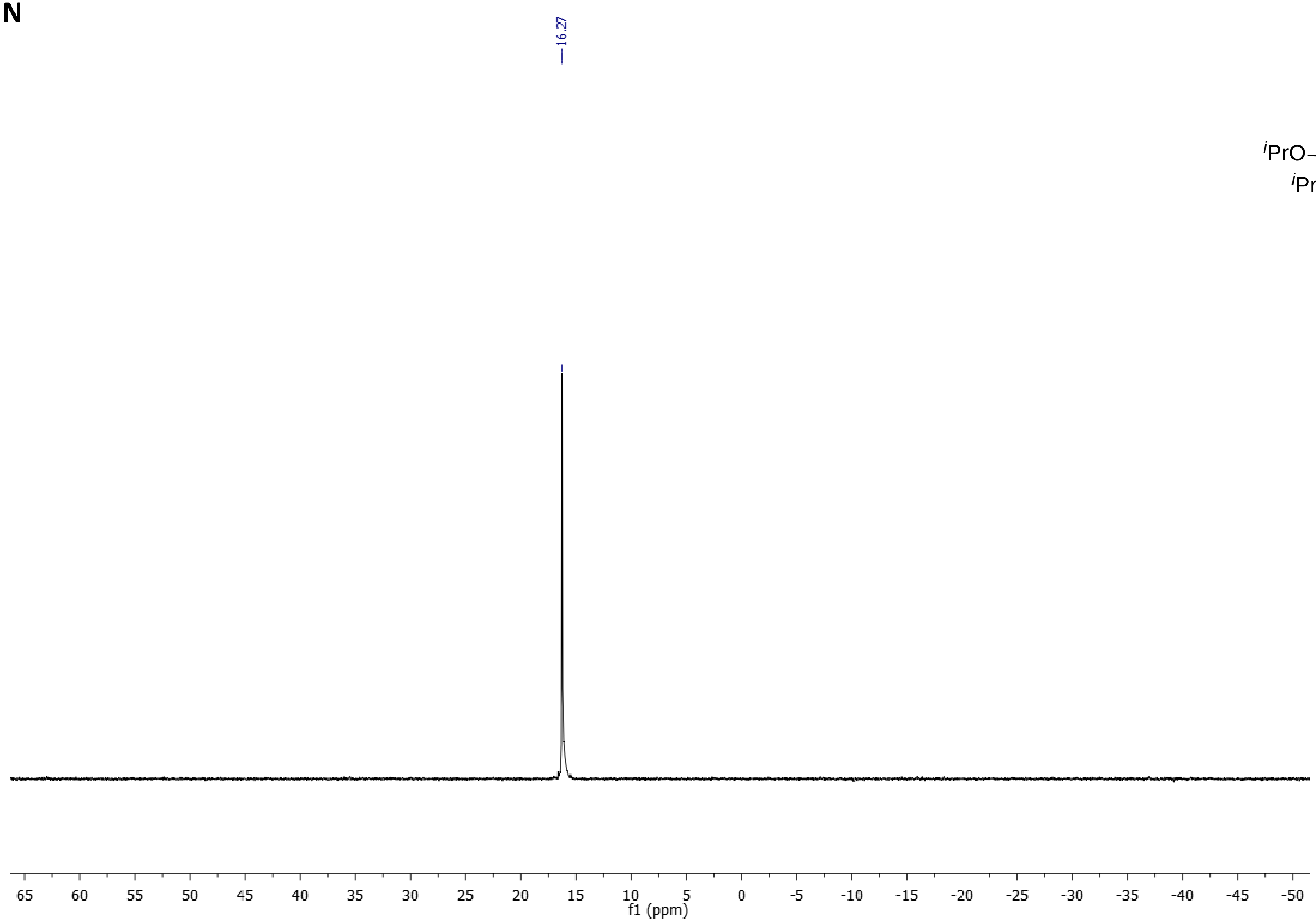

16.27
65
60
55
50
45
40
35
30
25
20
15
10
5
0
-5
-10
-15
-20
-25
-30
-35
-40
-45
-50
f1 (ppm)

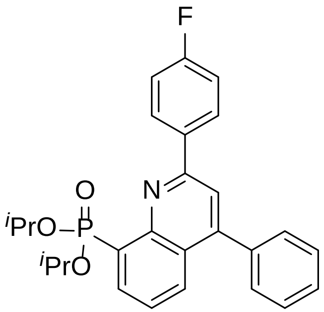

F
O
N
iPrO–P
iPrO

7l

^{19}F-RMN

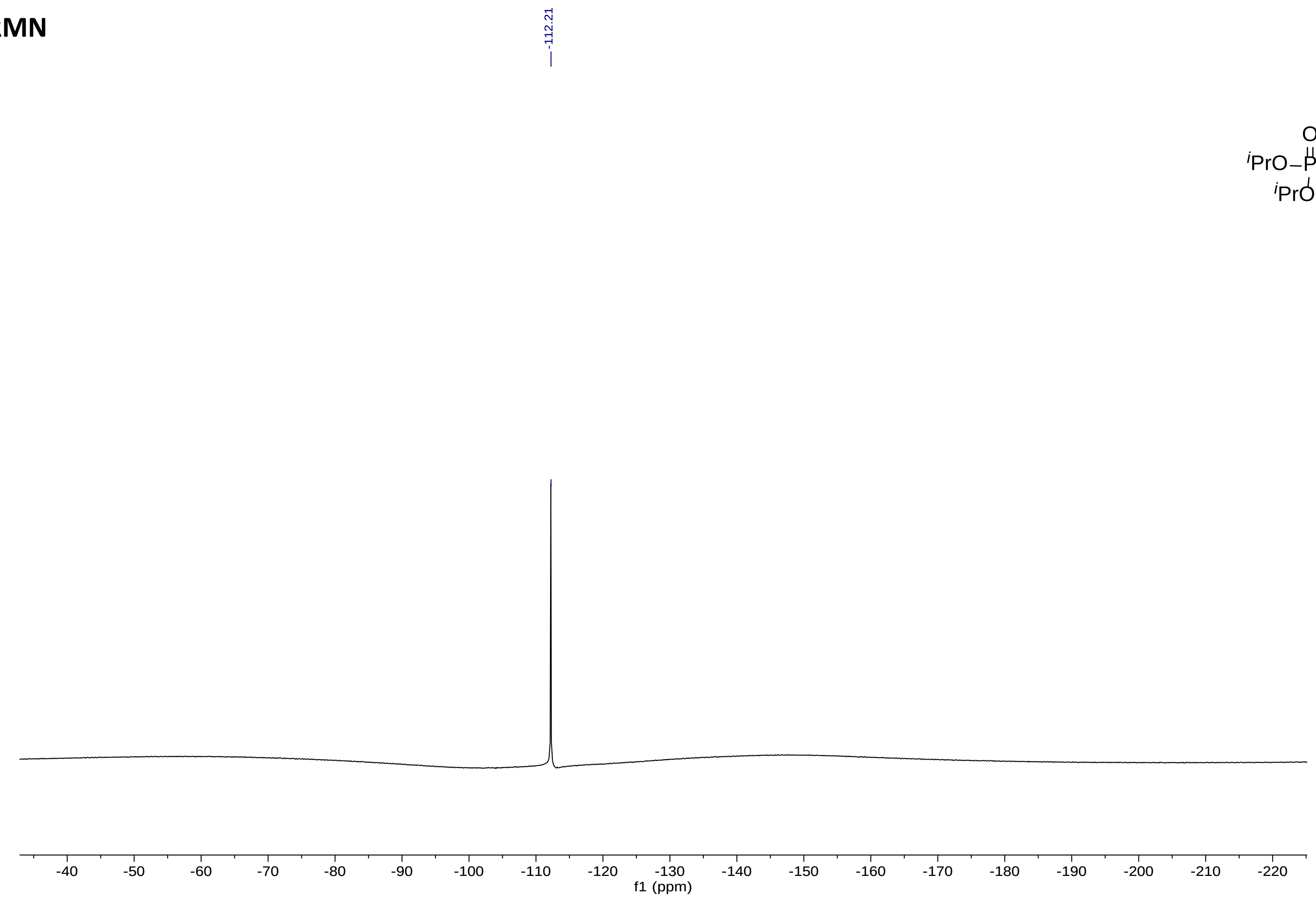

-112.21
-40
-50
-60
-70
-80
-90
-100
-110
-120
-130
-140
-150
-160
-170
-180
-190
-200
-210
-220
f1 (ppm)

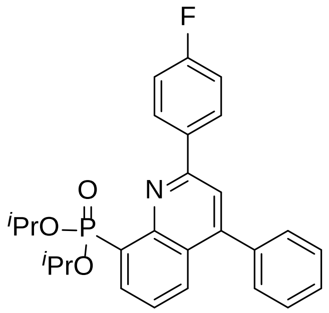

7l

¹H-RMN

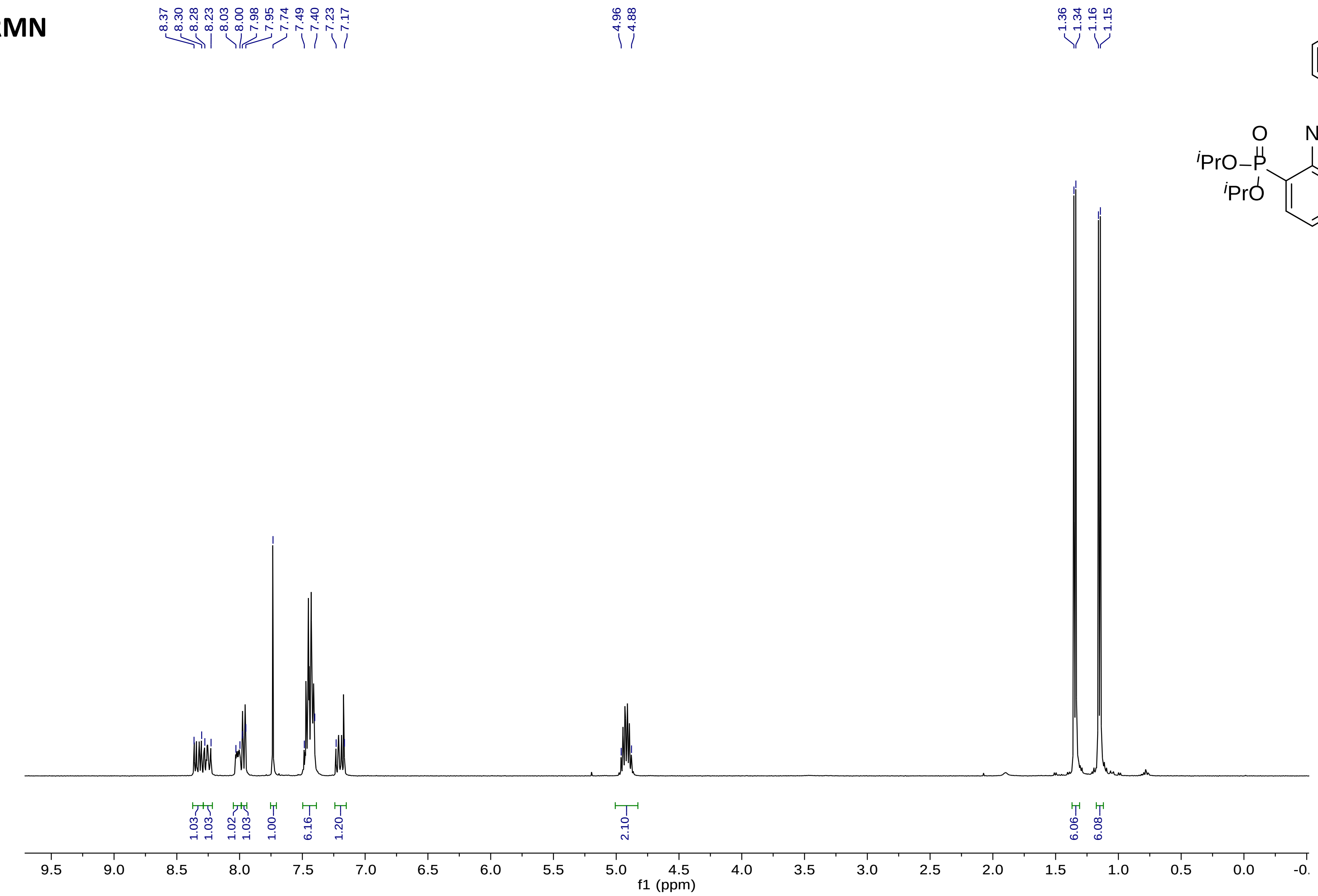
8.37
8.30
8.28
8.23
8.03
8.00
7.98
7.95
7.74
7.49
7.40
7.23
7.17
4.96
4.88
1.36
1.34
1.16
1.15
1.03
1.03
1.02
1.03
1.00
6.16
1.20
2.10
6.06
6.08
9.5
9.0
8.5
8.0
7.5
7.0
6.5
6.0
5.5
5.0
4.5
4.0
3.5
3.0
2.5
2.0
1.5
1.0
0.5
0.0
f1 (ppm)

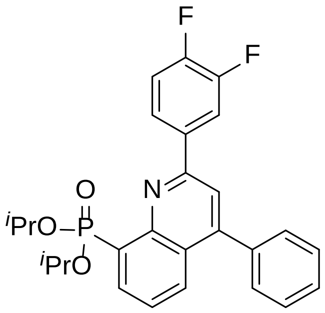
F
F
N
O
P
iPrO
iPrO

7m

^{13}C-RMN

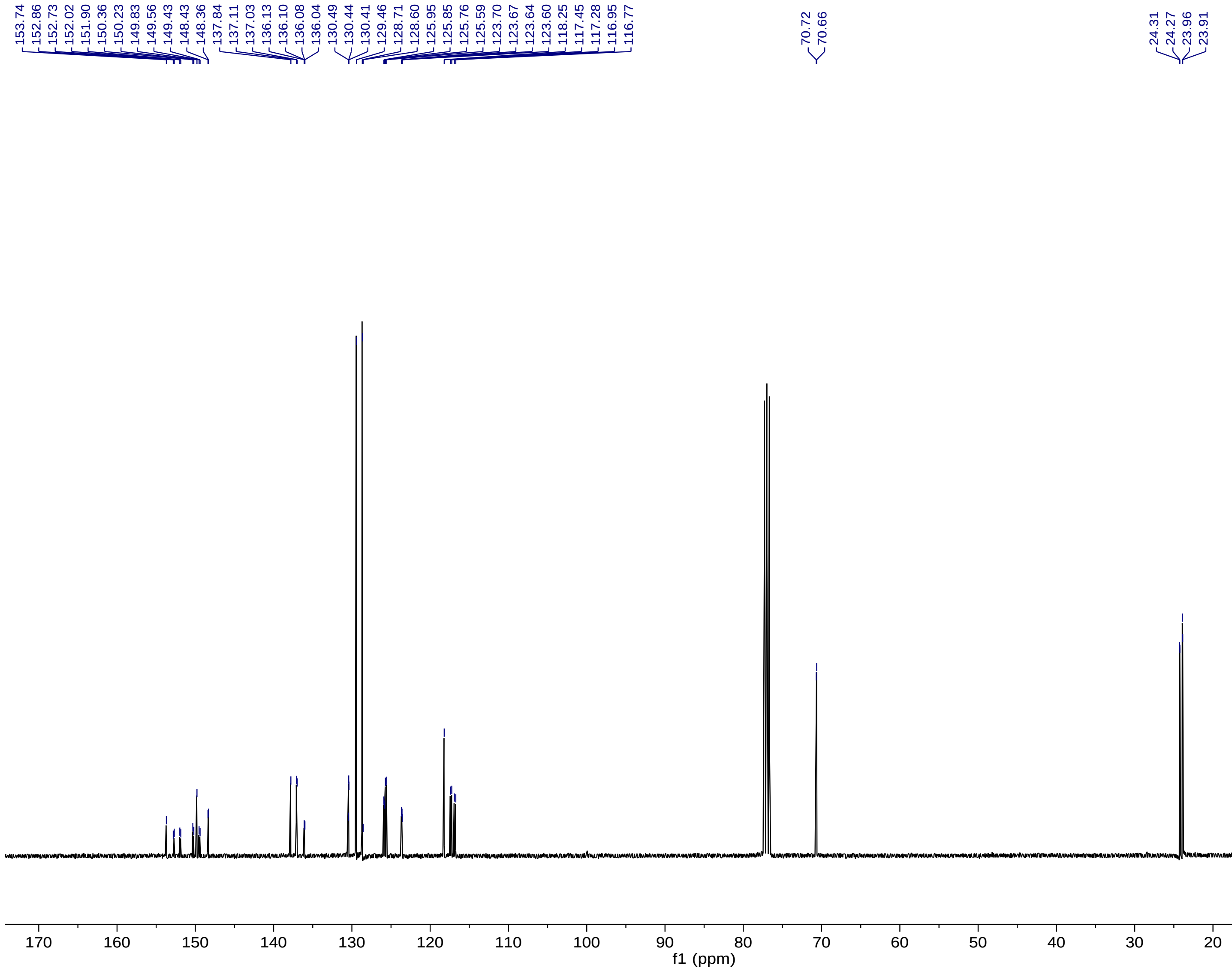
153.74
152.86
152.73
152.02
151.90
150.36
150.23
149.83
149.56
149.43
148.43
148.36
137.84
137.11
137.03
136.13
136.10
136.08
136.04
130.49
130.44
130.41
129.46
128.71
128.60
125.95
125.85
125.76
125.59
123.70
123.67
123.64
123.60
118.25
117.45
117.28
116.95
116.77
70.72
70.66
24.31
24.27
23.96
23.91
170
160
150
140
130
120
110
100
90
80
70
60
50
40
30
20
10
0
f1 (ppm)

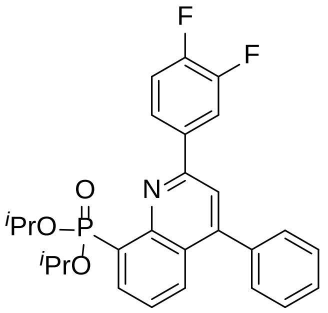
F
F
N
O
^{i}PrO
P
^{i}PrO

7m

^{31}P-RMN

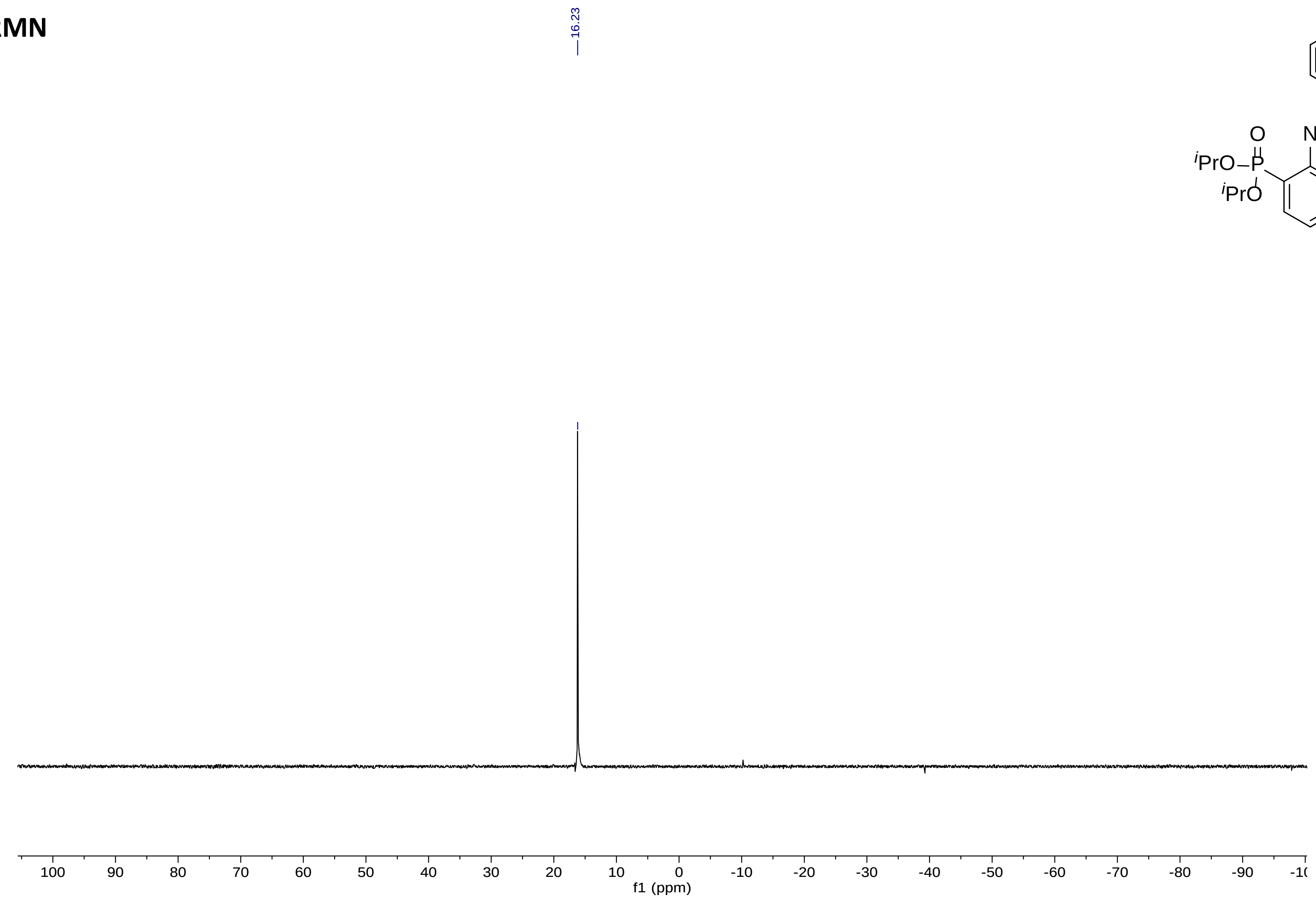
16.23
100 90 80 70 60 50 40 30 20 10 0 -10 -20 -30 -40 -50 -60 -70 -80 -90
f1 (ppm)

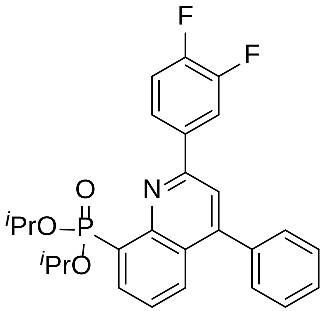
F
F
N
O
^{i}PrO–P
^{i}PrO

7m

^{19}F-RMN

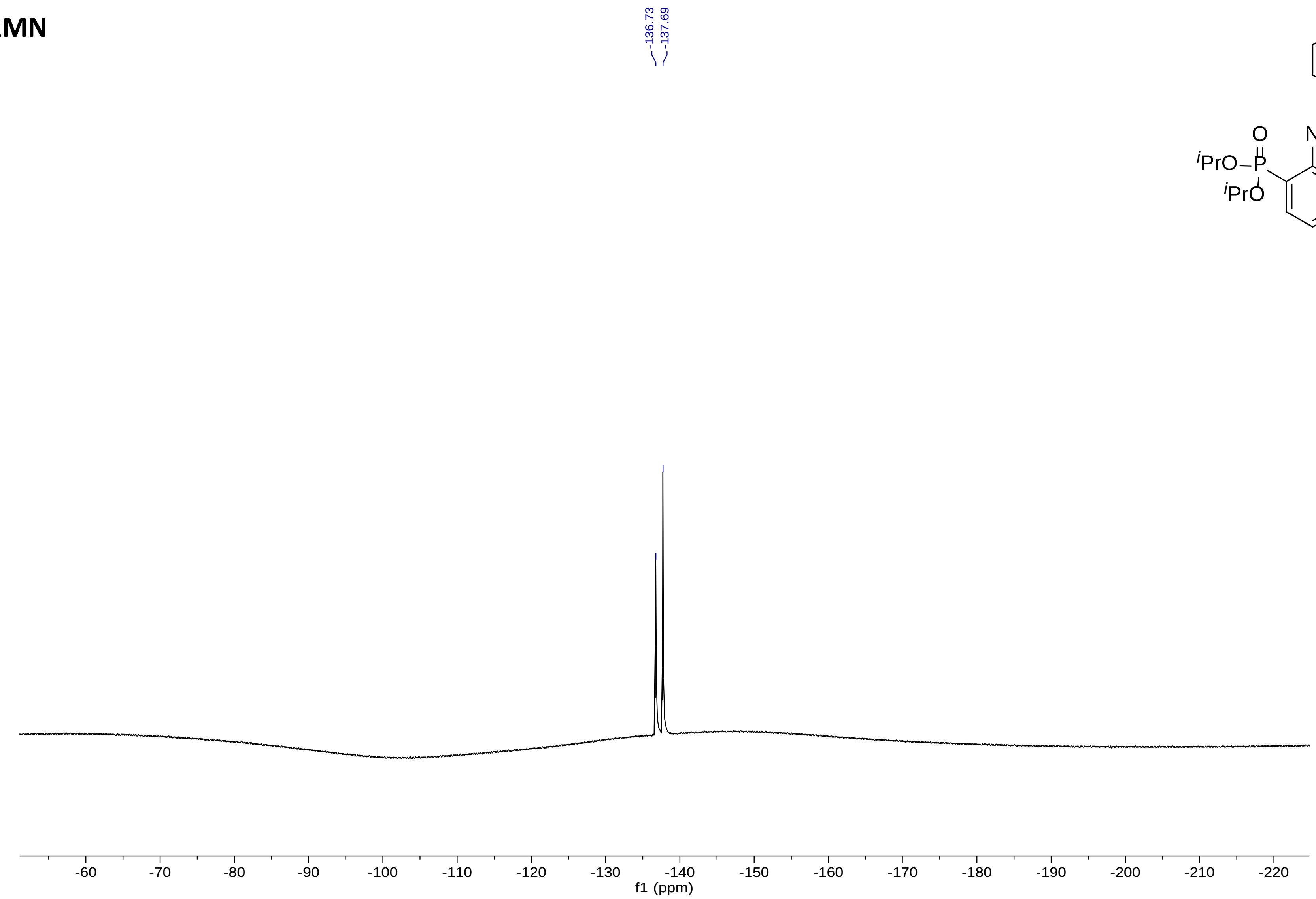

-136.73
-137.69
-60
-70
-80
-90
-100
-110
-120
-130
-140
-150
-160
-170
-180
-190
-200
-210
-220
f1 (ppm)

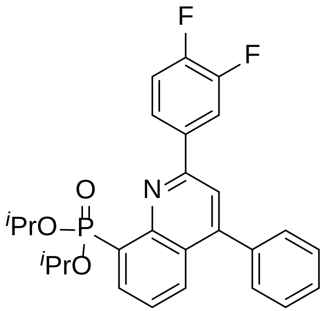

F
F
O
N
iPrO
P
iPrO

7m

^{1}H-RMN

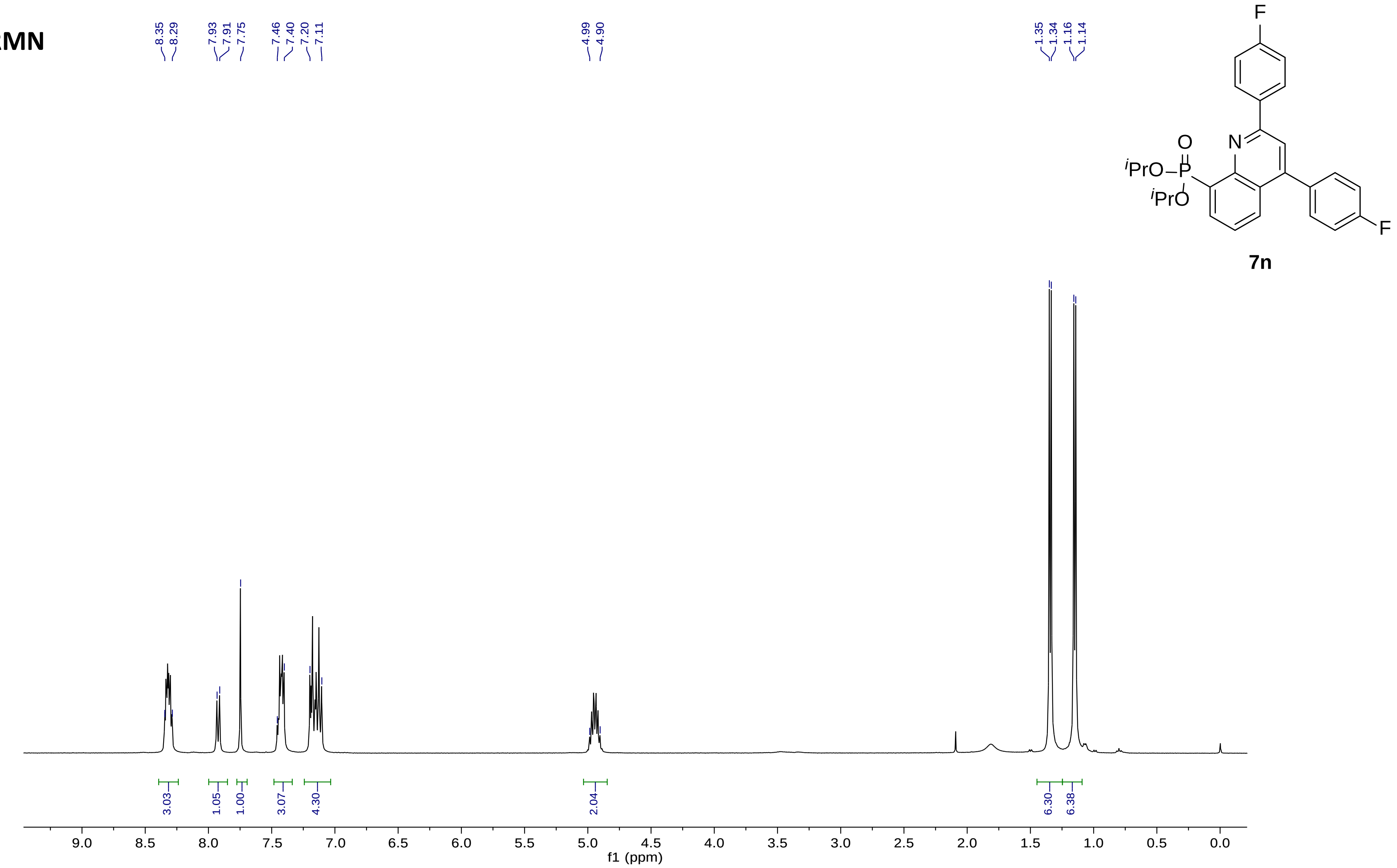

8.35
8.29
7.93
7.91
7.75
7.46
7.40
7.20
7.11
4.99
4.90
1.35
1.34
1.16
1.14
3.03
1.05
1.00
3.07
4.30
2.04
6.30
6.38
9.0
8.5
8.0
7.5
7.0
6.5
6.0
5.5
5.0
4.5
4.0
3.5
3.0
2.5
2.0
1.5
1.0
0.5
0.0
f1 (ppm)
F
O
N
iPrO
P
iPrO
F

7n

^{13}C-RMN

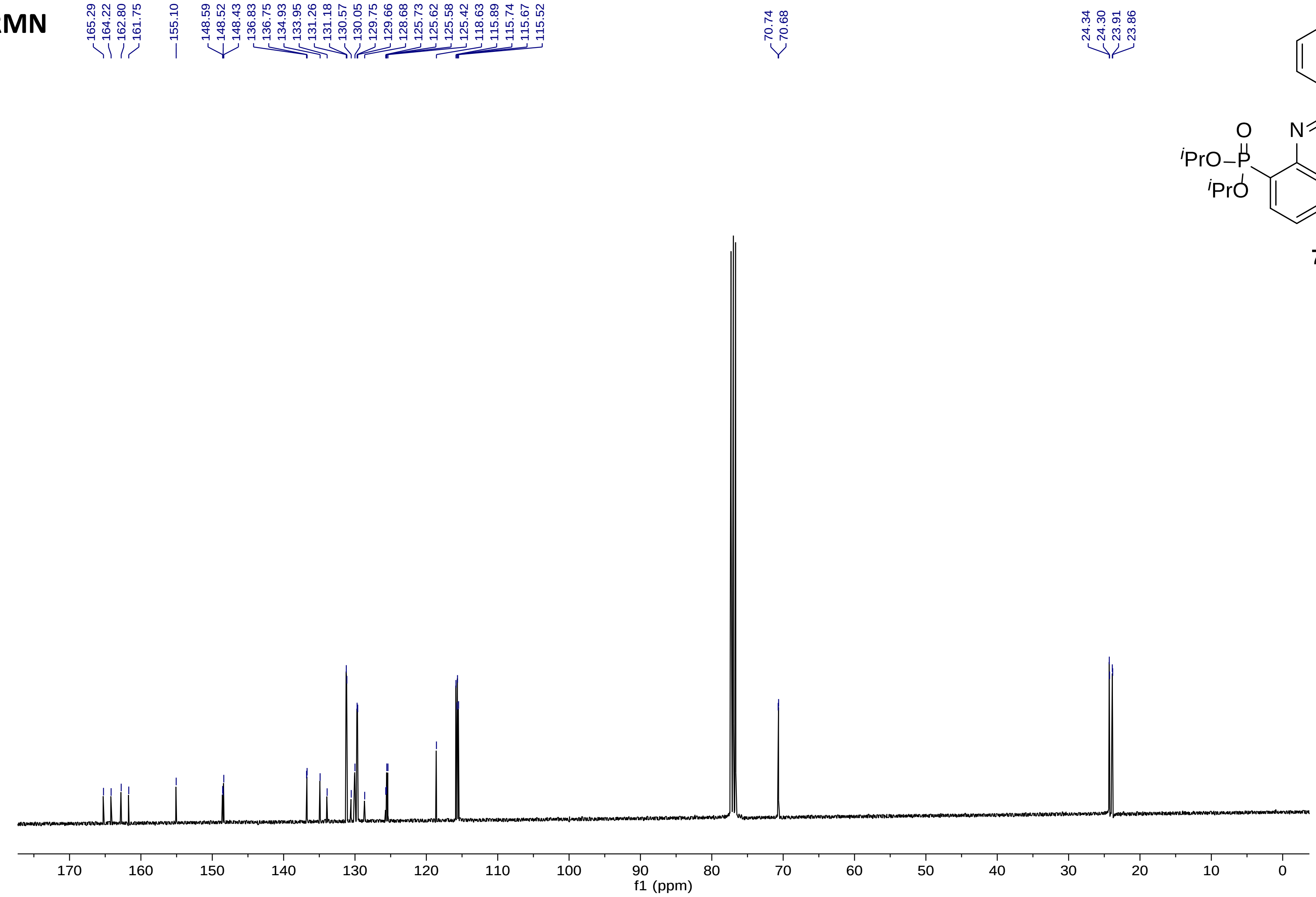
165.29
164.22
162.80
161.75
155.10
148.59
148.52
148.43
136.83
136.75
134.93
133.95
131.26
131.18
130.57
130.05
129.75
129.66
128.68
125.73
125.62
125.58
125.42
118.63
115.89
115.74
115.67
115.52
70.74
70.68
24.34
24.30
23.91
23.86
170 160 150 140 130 120 110 100 90 80 70 60 50 40 30 20 10 0
f1 (ppm)

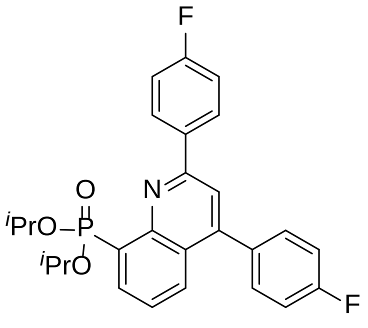
F
O
N
iPrO–P
iPrO
F

7n

^{31}P-RMN

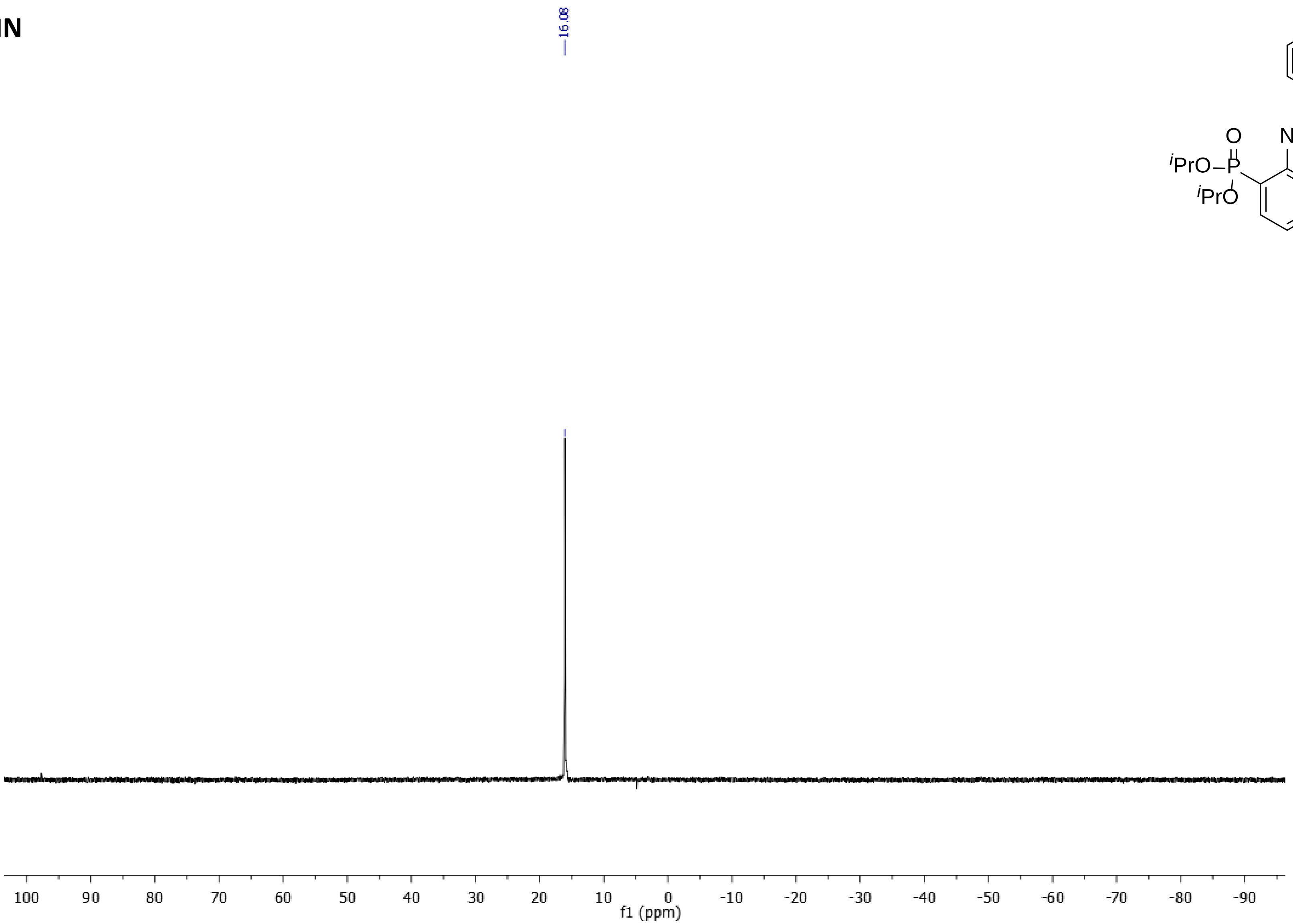
16.08
100
90
80
70
60
50
40
30
20
10
0
-10
-20
-30
-40
-50
-60
-70
-80
-90
f1 (ppm)

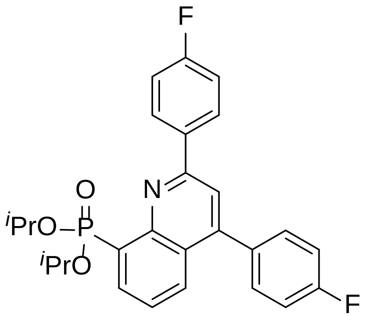
F
O
N
iPrO
P
iPrO
F

7n

^{19}F-RMN

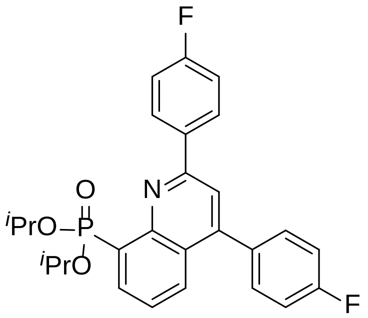

F
N
O
^{i}PrO–P
^{i}PrO
F

7n

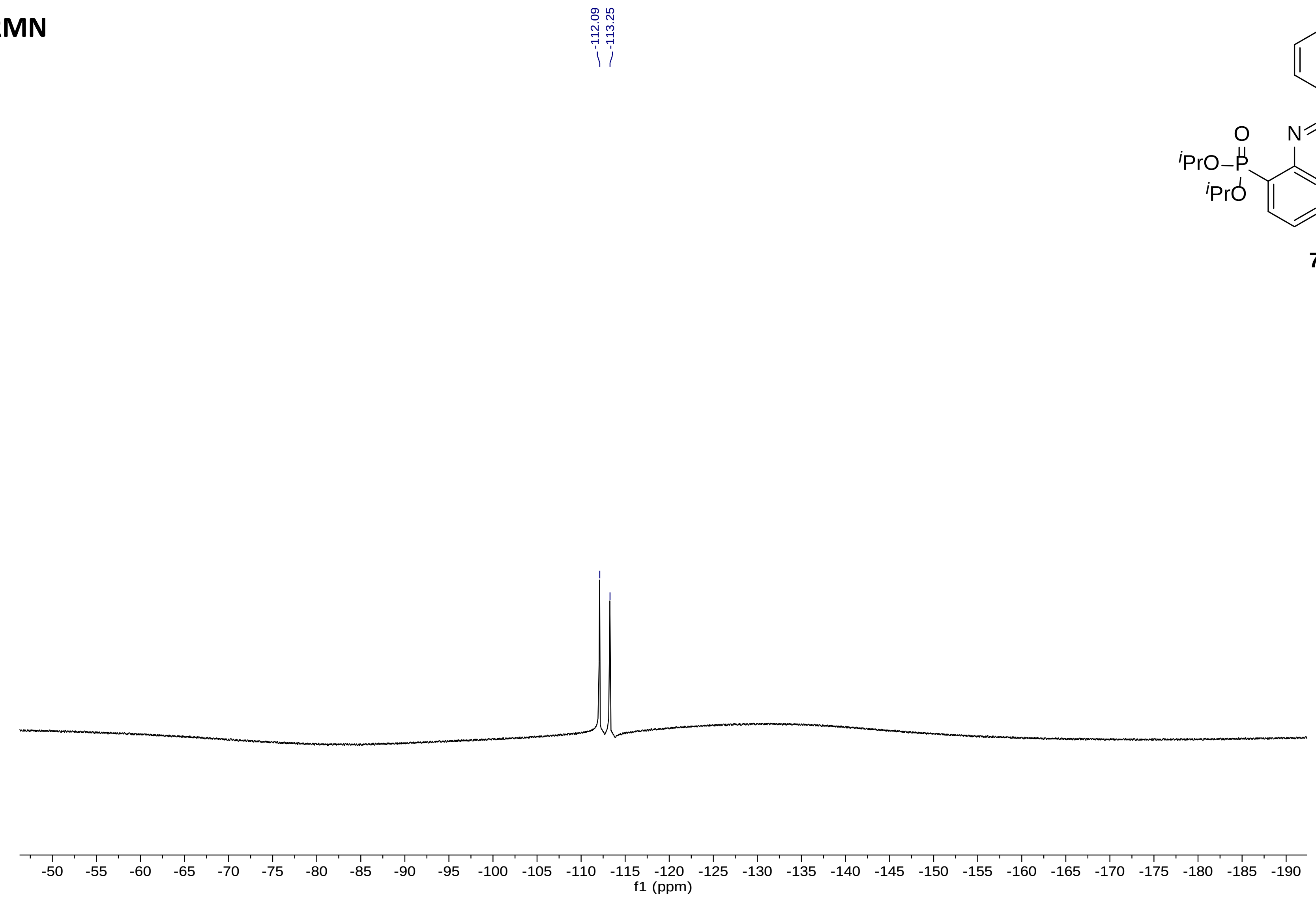

-112.09
-113.25
-50
-55
-60
-65
-70
-75
-80
-85
-90
-95
-100
-105
-110
-115
-120
-125
-130
-135
-140
-145
-150
-155
-160
-165
-170
-175
-180
-185
-190
f1 (ppm)

^{1}H-RMN

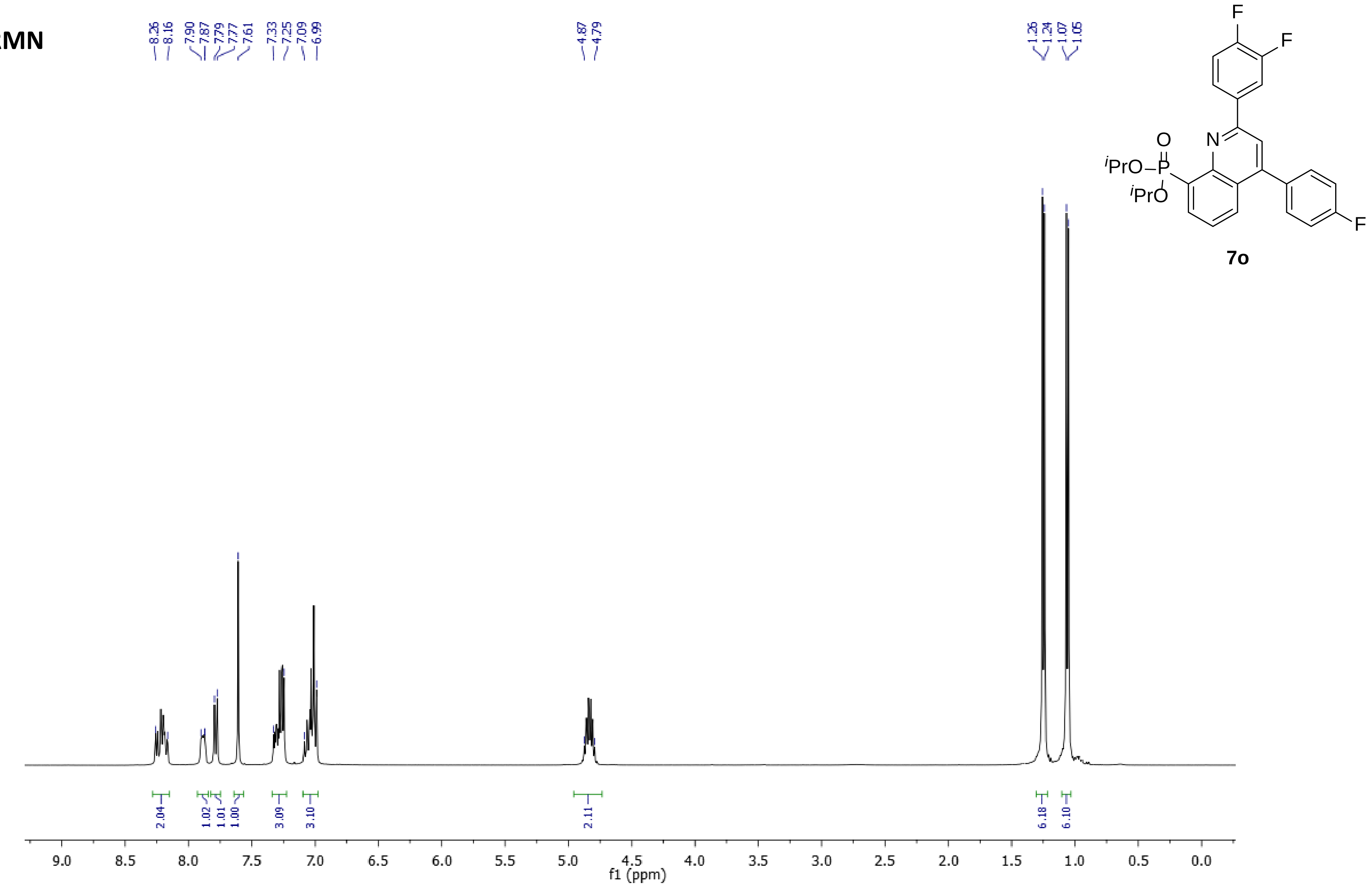
8.26
8.16
7.90
7.87
7.79
7.77
7.61
7.33
7.25
7.09
6.99
4.87
4.79
1.26
1.24
1.07
1.05
2.04
1.02
1.01
1.00
3.09
3.10
2.11
6.18
6.10
9.0
8.5
8.0
7.5
7.0
6.5
6.0
5.5
5.0
4.5
4.0
3.5
3.0
2.5
2.0
1.5
1.0
0.5
0.0
f1 (ppm)
F
F
N
O
^{i}PrO–P
^{i}PrO
F

7o

^{13}C-RMN

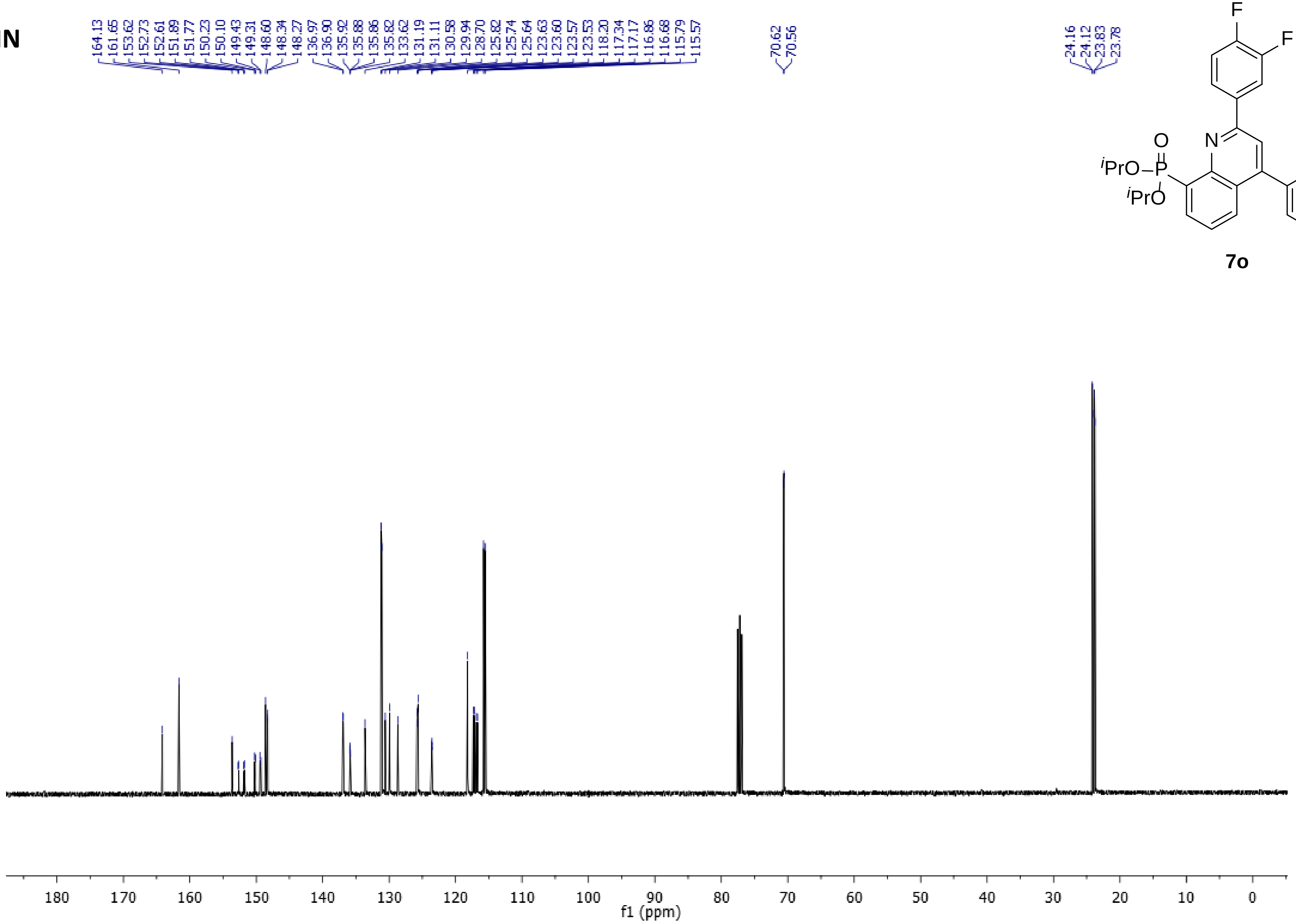
164.13
161.65
153.62
152.73
152.61
151.89
151.77
150.23
150.10
149.43
149.31
148.60
148.34
148.27
136.97
136.90
135.92
135.88
135.86
135.82
133.62
131.19
131.11
130.58
129.94
128.70
125.82
125.74
125.64
123.63
123.60
123.57
123.53
118.20
117.34
117.17
116.86
116.68
115.79
115.57
70.62
70.56
24.16
24.12
23.83
23.78
180
170
160
150
140
130
120
110
100
90
80
70
60
50
40
30
20
10
0
f1 (ppm)

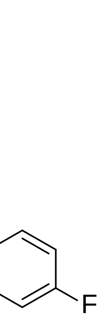
F
F
N
O
^{i}PrO
P
^{i}PrO
F

7o

^{31}P-RMN

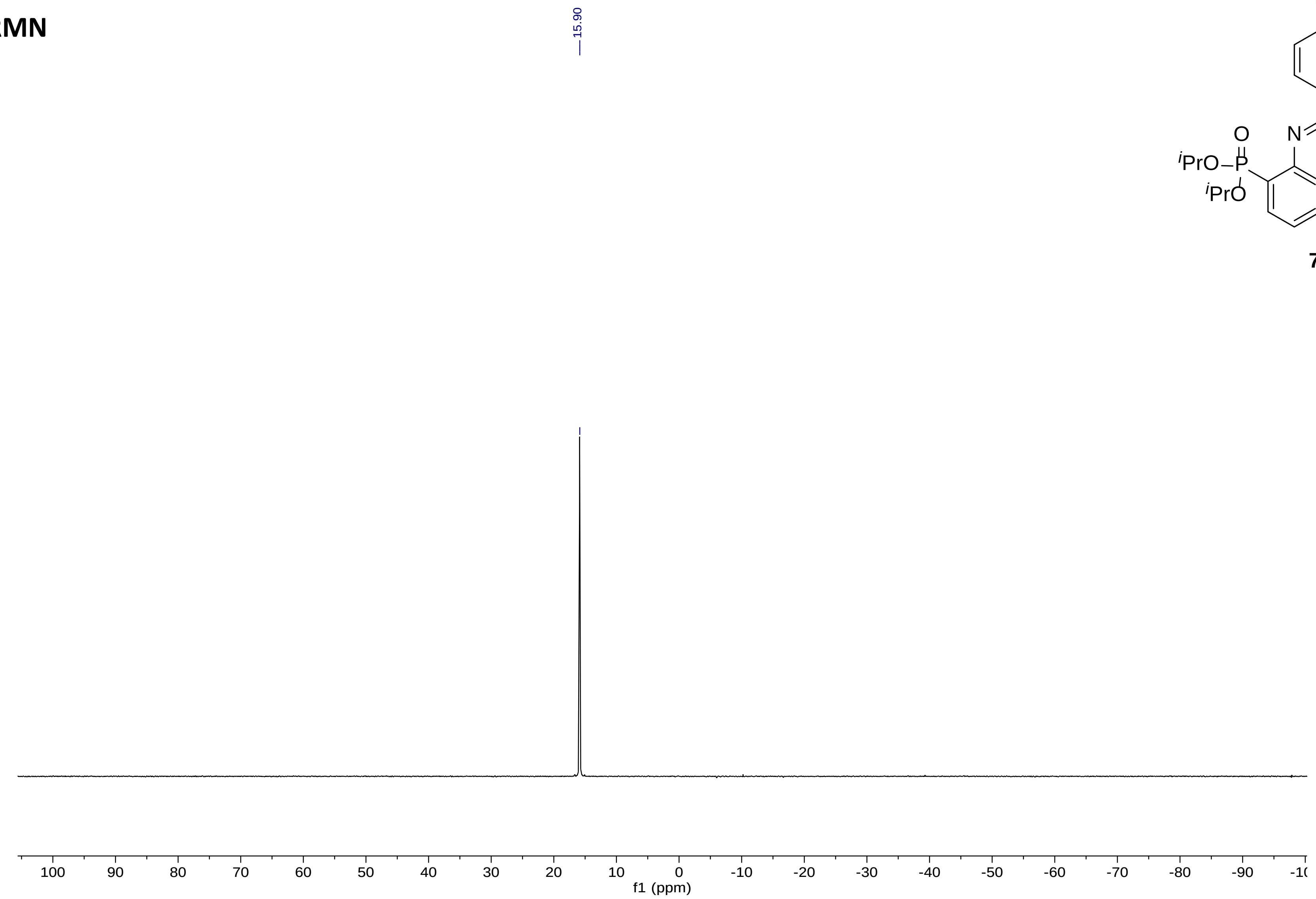
15.90
100
90
80
70
60
50
40
30
20
10
0
-10
-20
-30
-40
-50
-60
-70
-80
-90
f1 (ppm)

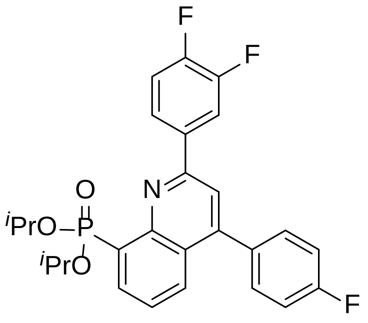
F
F
O
N
^{i}PrO–P
^{i}PrO
F

7o

^{19}F-RMN

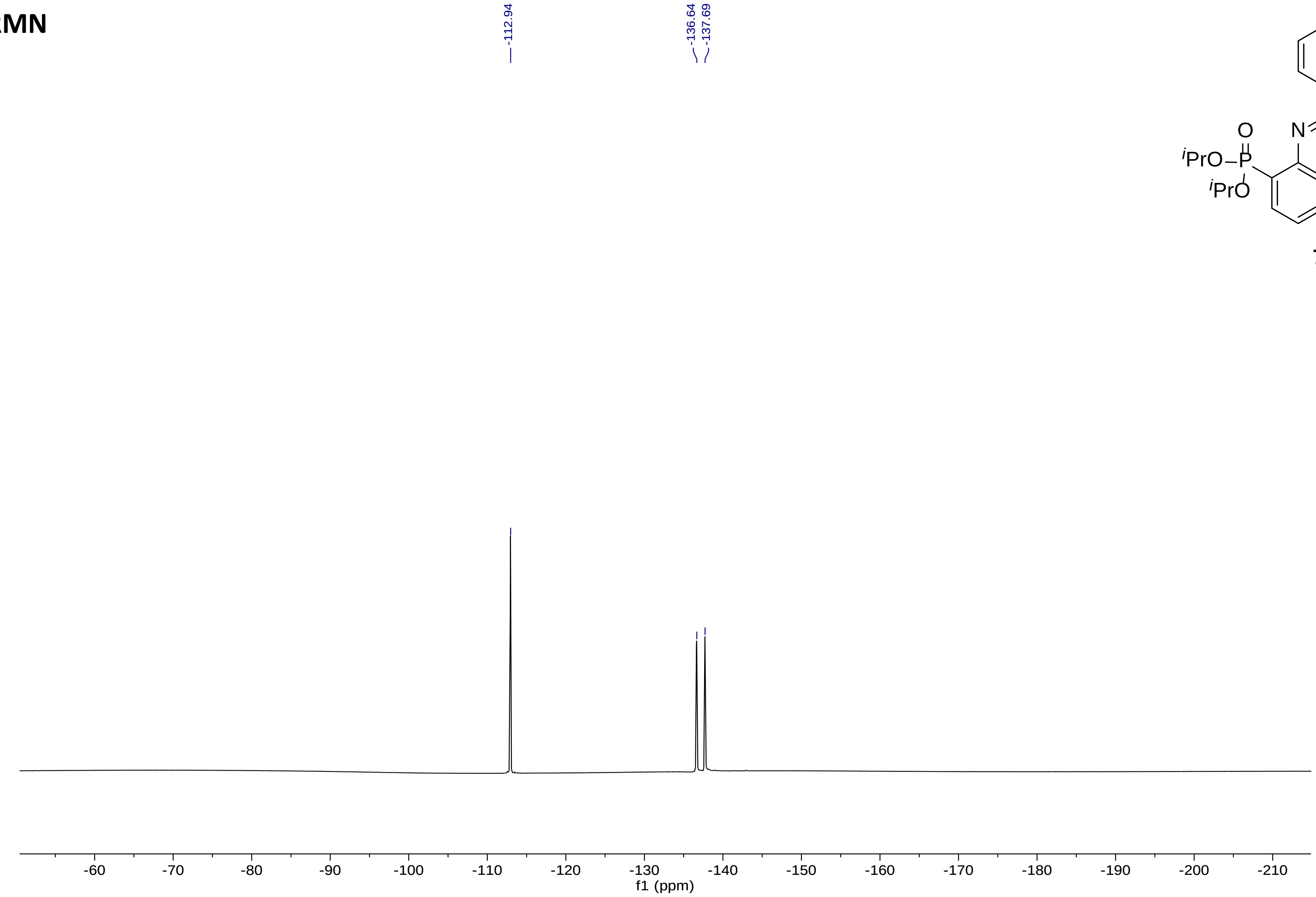

-112.94
-136.64
-137.69
-60
-70
-80
-90
-100
-110
-120
-130
-140
-150
-160
-170
-180
-190
-200
-210
f1 (ppm)

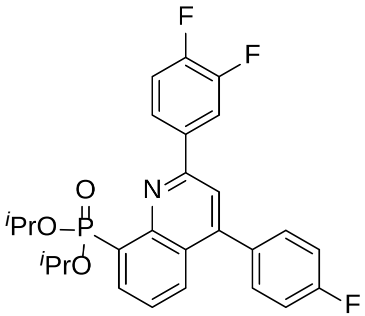

F
F
N
O
^{i}PrO–P
^{i}PrO
F

7o

^{1}H-RMN

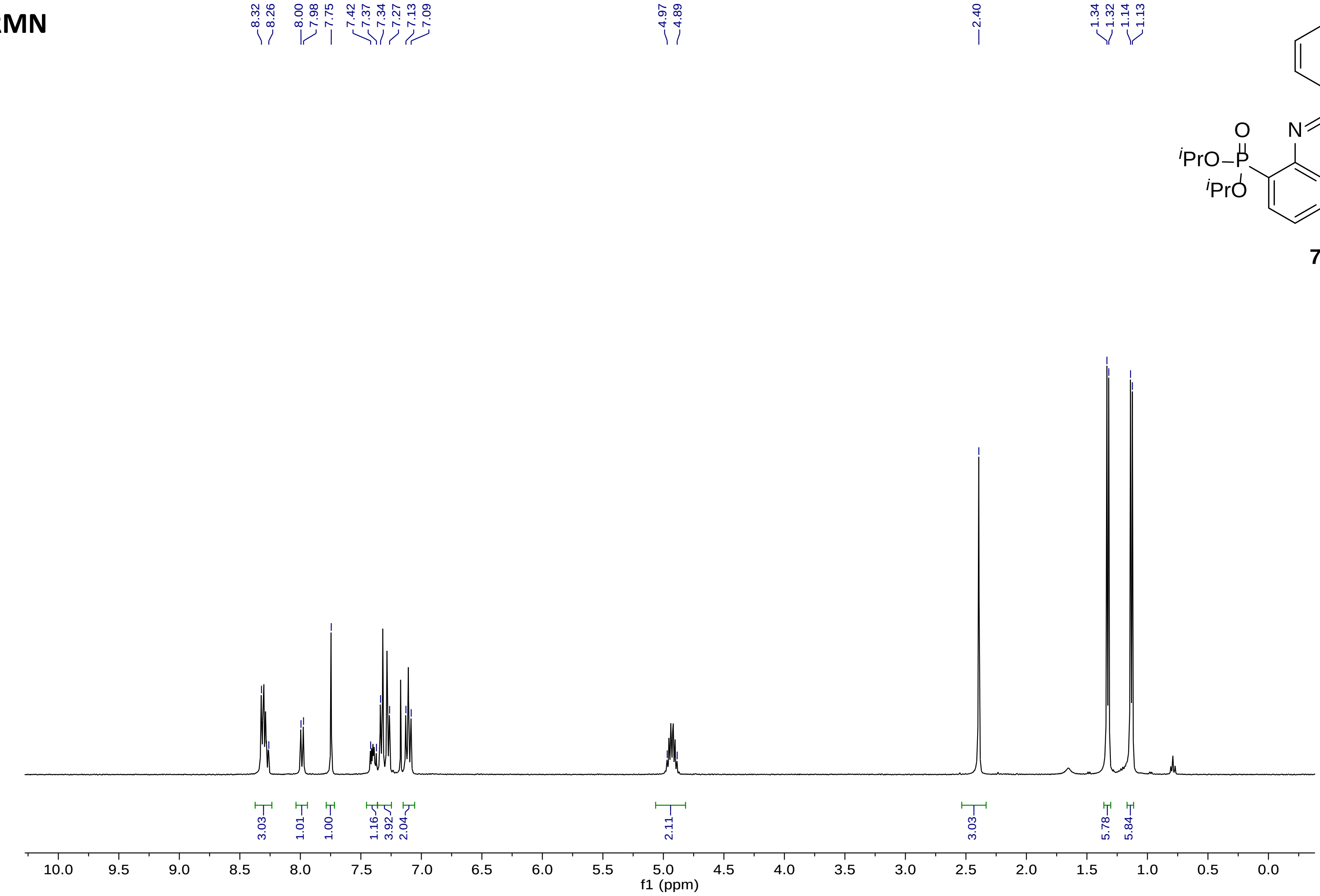
8.32
8.26
8.00
7.98
7.75
7.42
7.37
7.34
7.27
7.13
7.09
4.97
4.89
2.40
1.34
1.32
1.14
1.13
3.03
1.01
1.00
1.16
3.92
2.04
2.11
3.03
5.78
5.84
10.0
9.5
9.0
8.5
8.0
7.5
7.0
6.5
6.0
5.5
5.0
4.5
4.0
3.5
3.0
2.5
2.0
1.5
1.0
0.5
0.0
f1 (ppm)

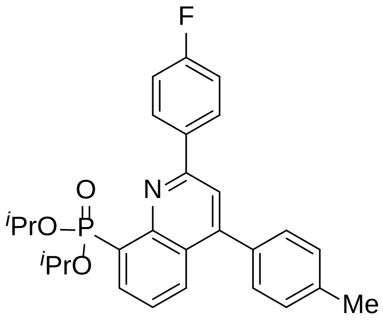
F
N
O
^{i}PrO
P
^{i}PrO
Me

7p

^{13}C-RMN

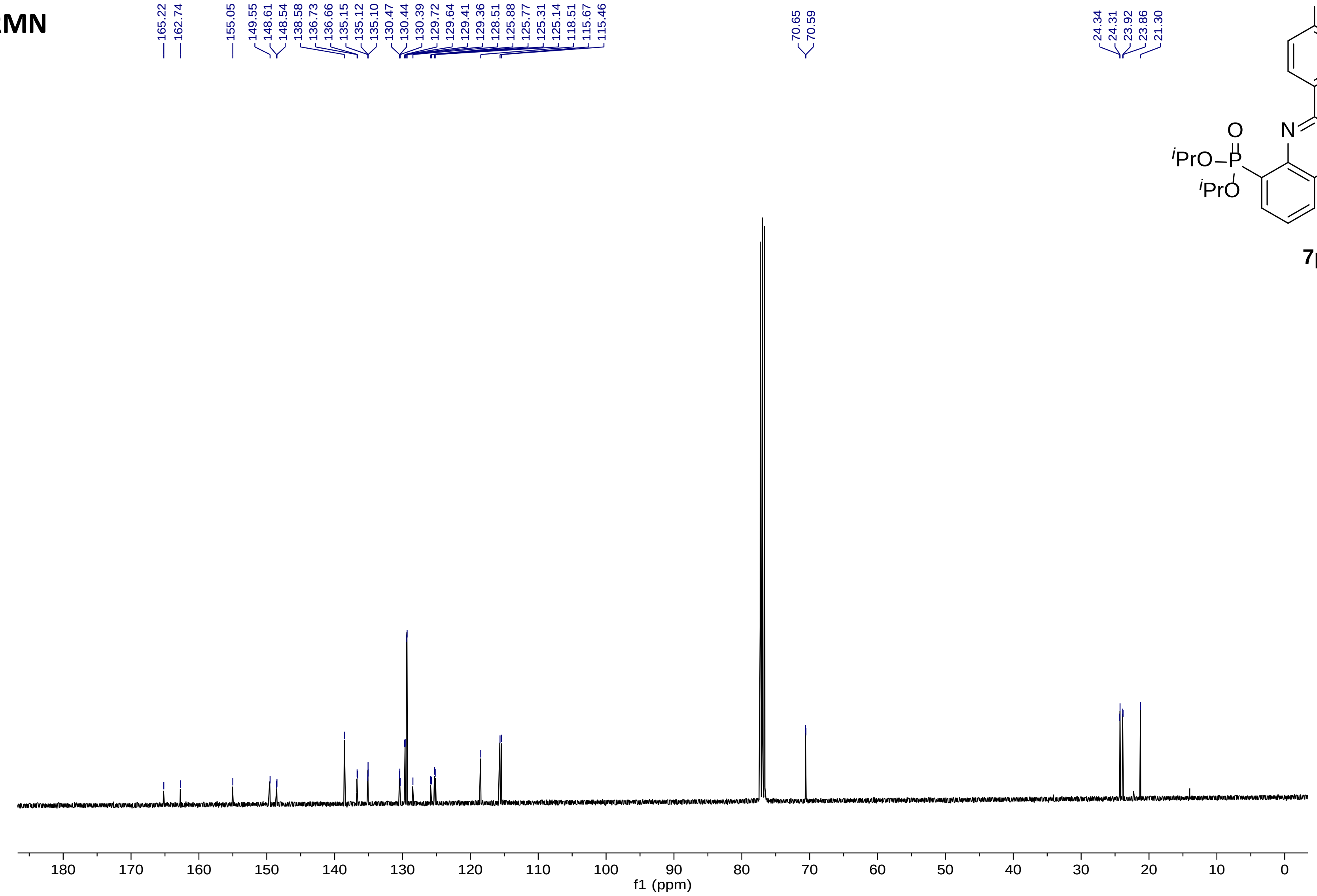

165.22
162.74
155.05
149.55
148.61
148.54
138.58
136.73
136.66
135.15
135.12
135.10
130.47
130.44
130.39
129.72
129.64
129.41
129.36
128.51
125.88
125.77
125.31
125.14
118.51
115.67
115.46
70.65
70.59
24.34
24.31
23.92
23.86
21.30
180
170
160
150
140
130
120
110
100
90
80
70
60
50
40
30
20
10
0
f1 (ppm)

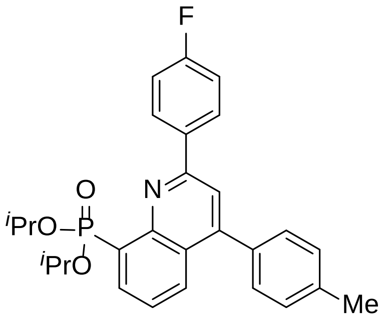

F
N
O
^{i}PrO–P
^{i}PrO
Me

7p

^{31}P-RMN

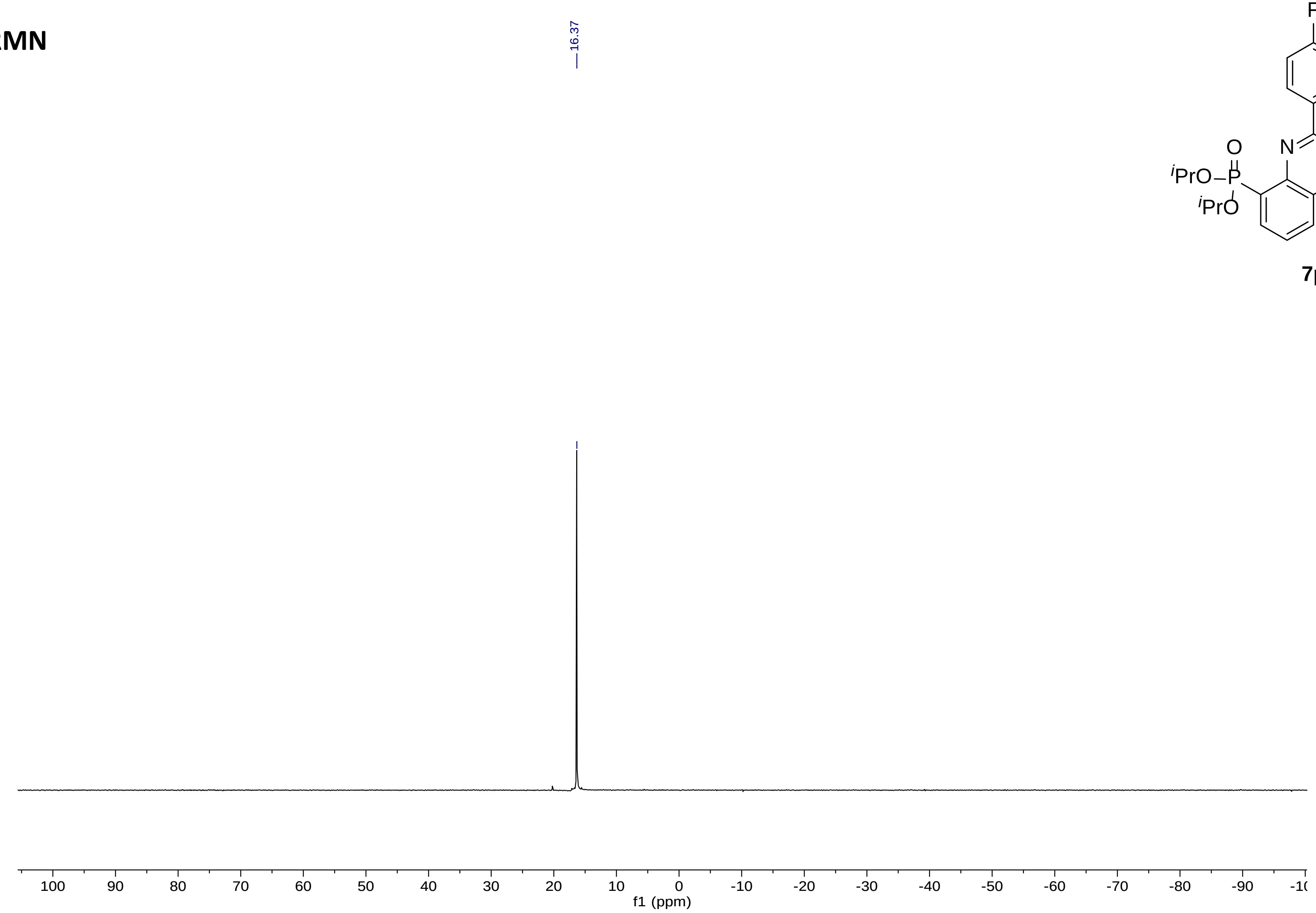
16.37
100
90
80
70
60
50
40
30
20
10
0
-10
-20
-30
-40
-50
-60
-70
-80
-90
f1 (ppm)

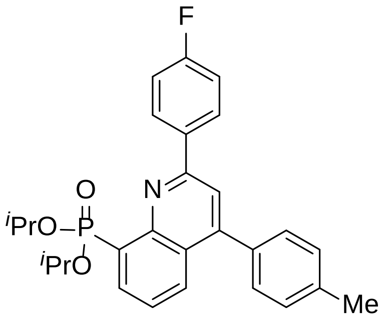
F
N
O
^{i}PrO
P
^{i}PrO
Me

7p

^{19}F-RMN

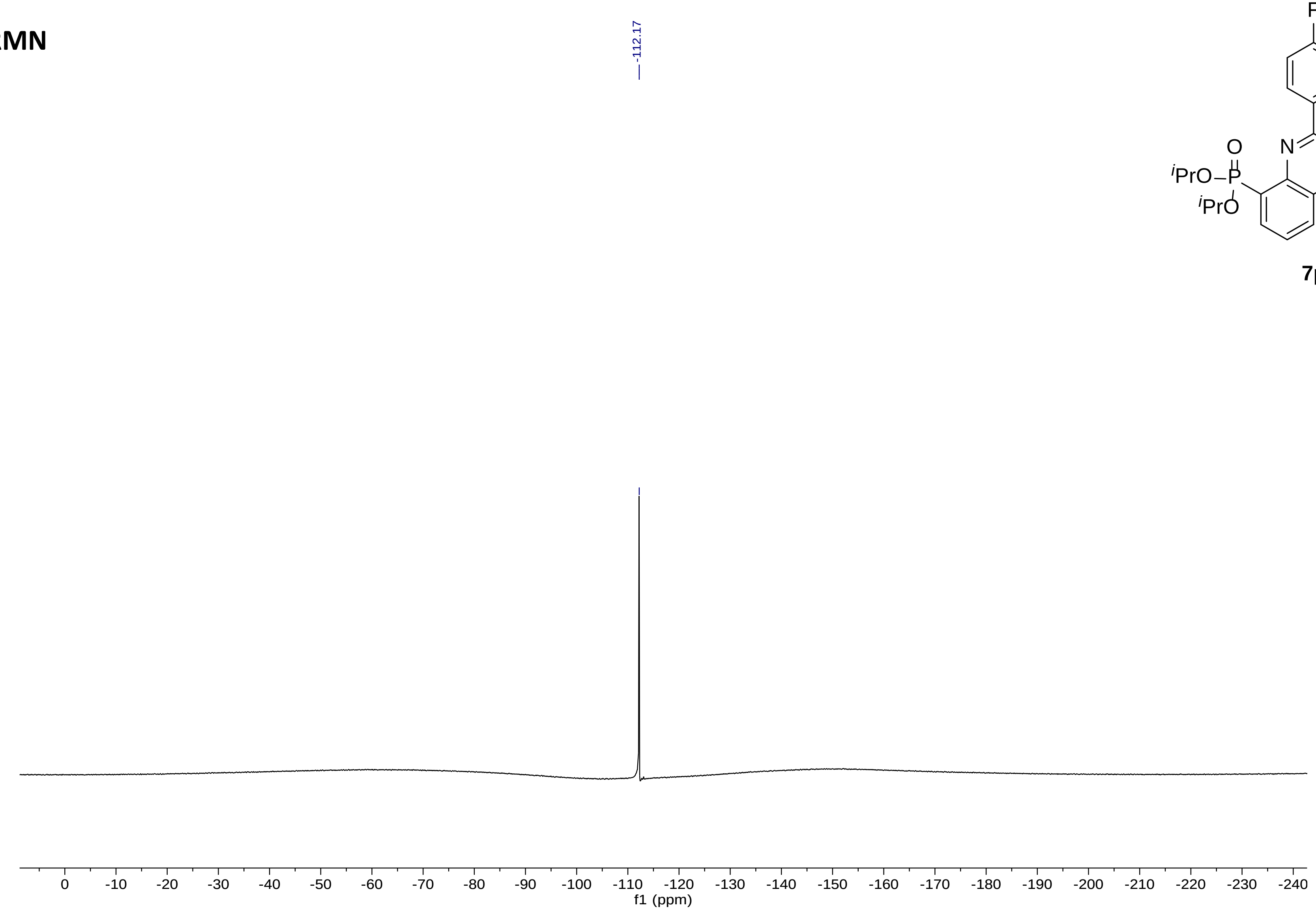

-112.17
0
-10
-20
-30
-40
-50
-60
-70
-80
-90
-100
-110
-120
-130
-140
-150
-160
-170
-180
-190
-200
-210
-220
-230
-240
f1 (ppm)

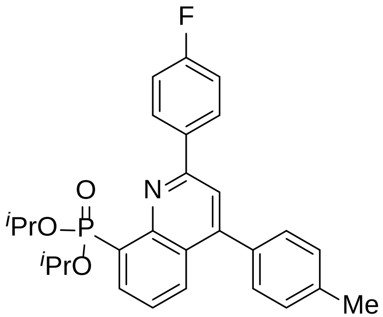

F
N
O
^{i}PrO
P
^{i}PrO
Me

7p

^{1}H-RMN

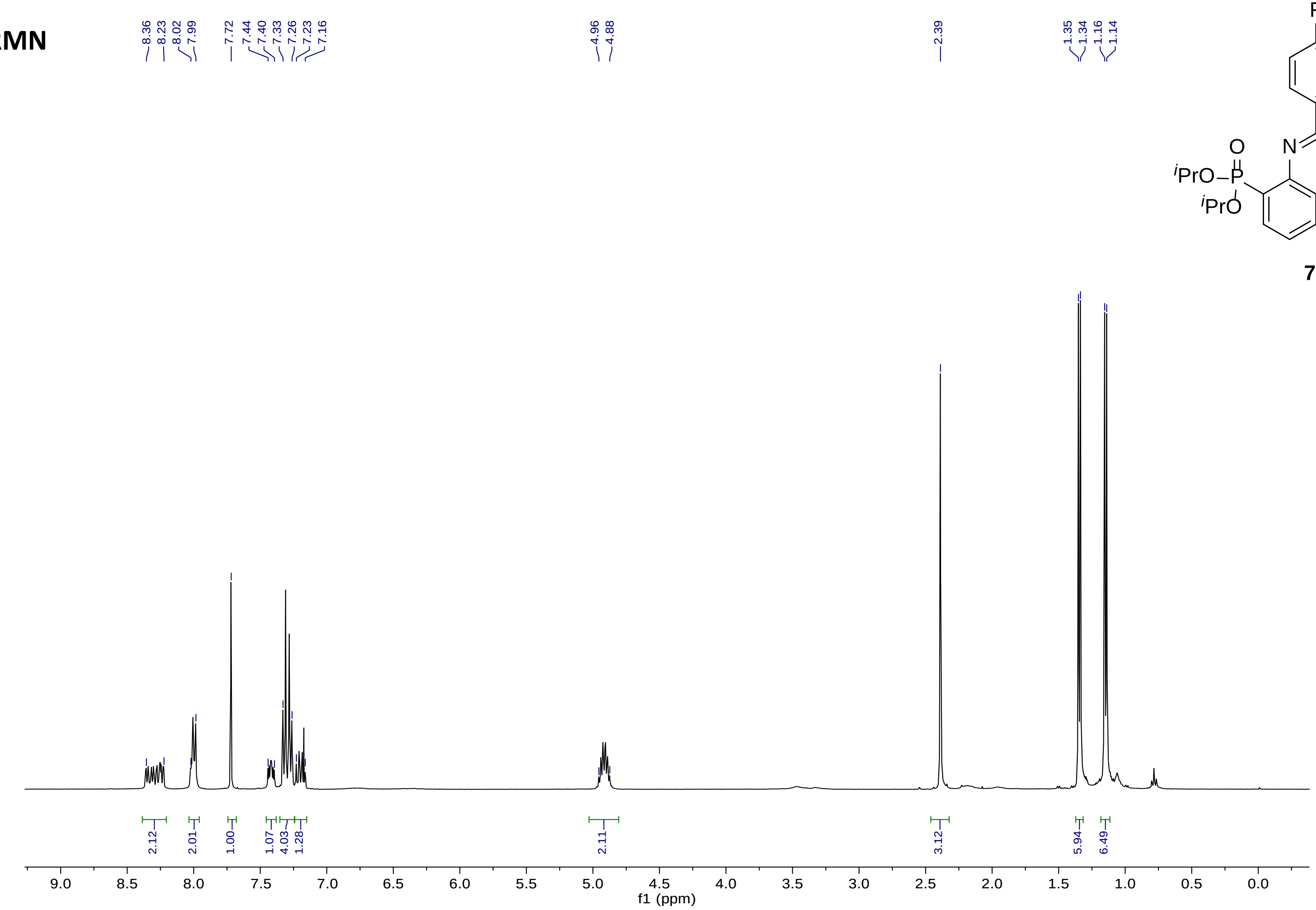

8.36
8.23
8.02
7.99
7.72
7.44
7.40
7.33
7.26
7.23
7.16
4.96
4.88
2.39
1.35
1.34
1.16
1.14
2.12
2.01
1.00
1.07
4.03
1.28
2.11
3.12
5.94
6.49
9.0
8.5
8.0
7.5
7.0
6.5
6.0
5.5
5.0
4.5
4.0
3.5
3.0
2.5
2.0
1.5
1.0
0.5
0.0
f1 (ppm)

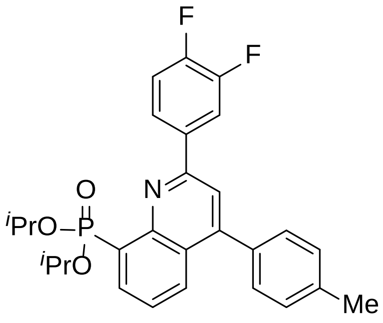

F
F
N
O
iPrO
P
iPrO
Me

7q

^{13}C-RMN

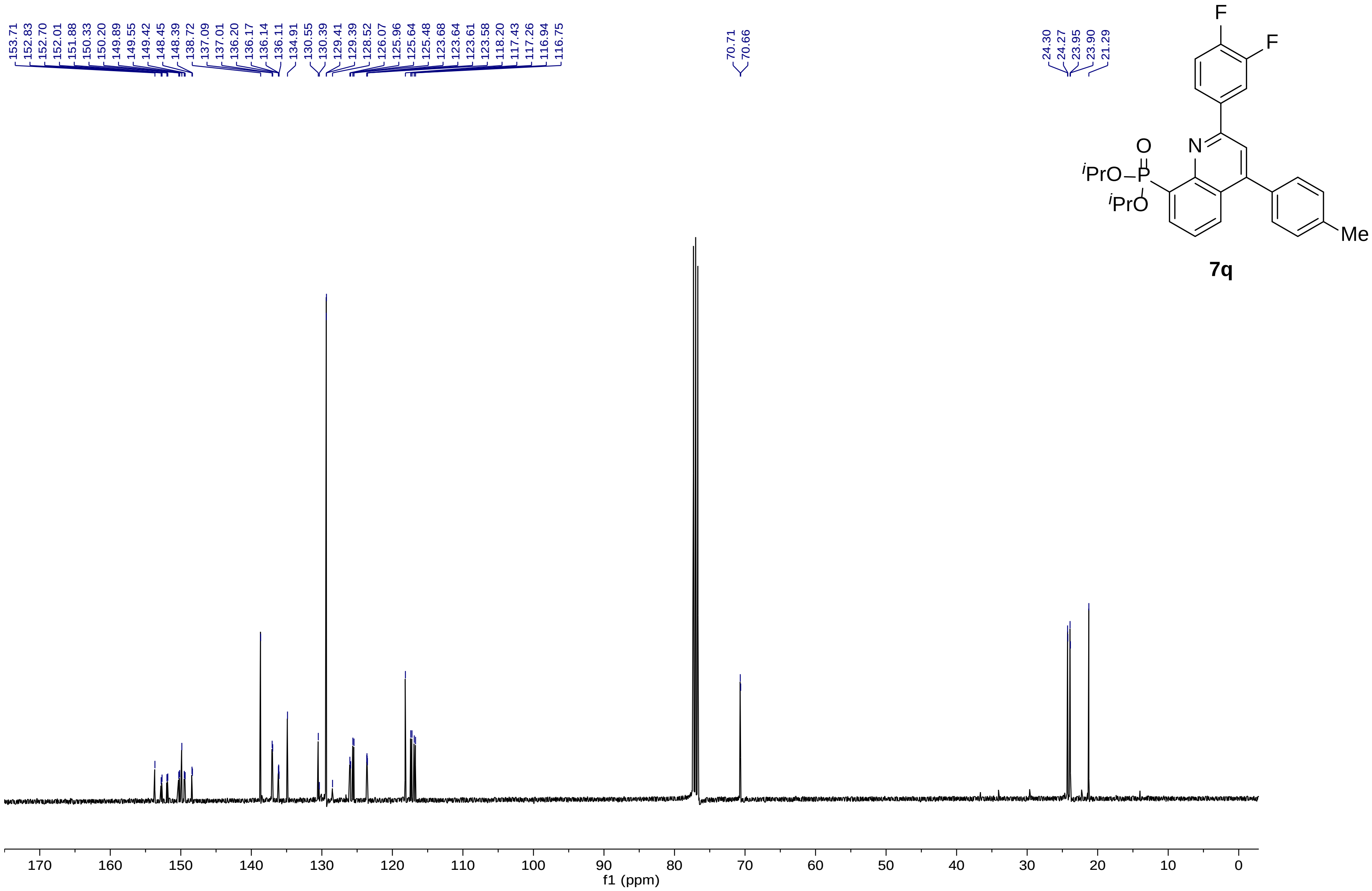
153.71
152.83
152.70
152.01
151.88
150.33
150.20
149.89
149.55
149.42
148.45
148.39
138.72
137.09
137.01
136.20
136.17
136.14
136.11
134.91
130.55
130.39
129.41
129.39
128.52
126.07
125.96
125.64
125.48
123.68
123.64
123.61
123.58
118.20
117.43
117.26
116.94
116.75
70.71
70.66
24.30
24.27
23.95
23.90
21.29
170 160 150 140 130 120 110 100 90 80 70 60 50 40 30 20 10 0
f1 (ppm)
F
F
N
O
^{i}PrO–P
^{i}PrO
Me

7q

^{31}P-RMN

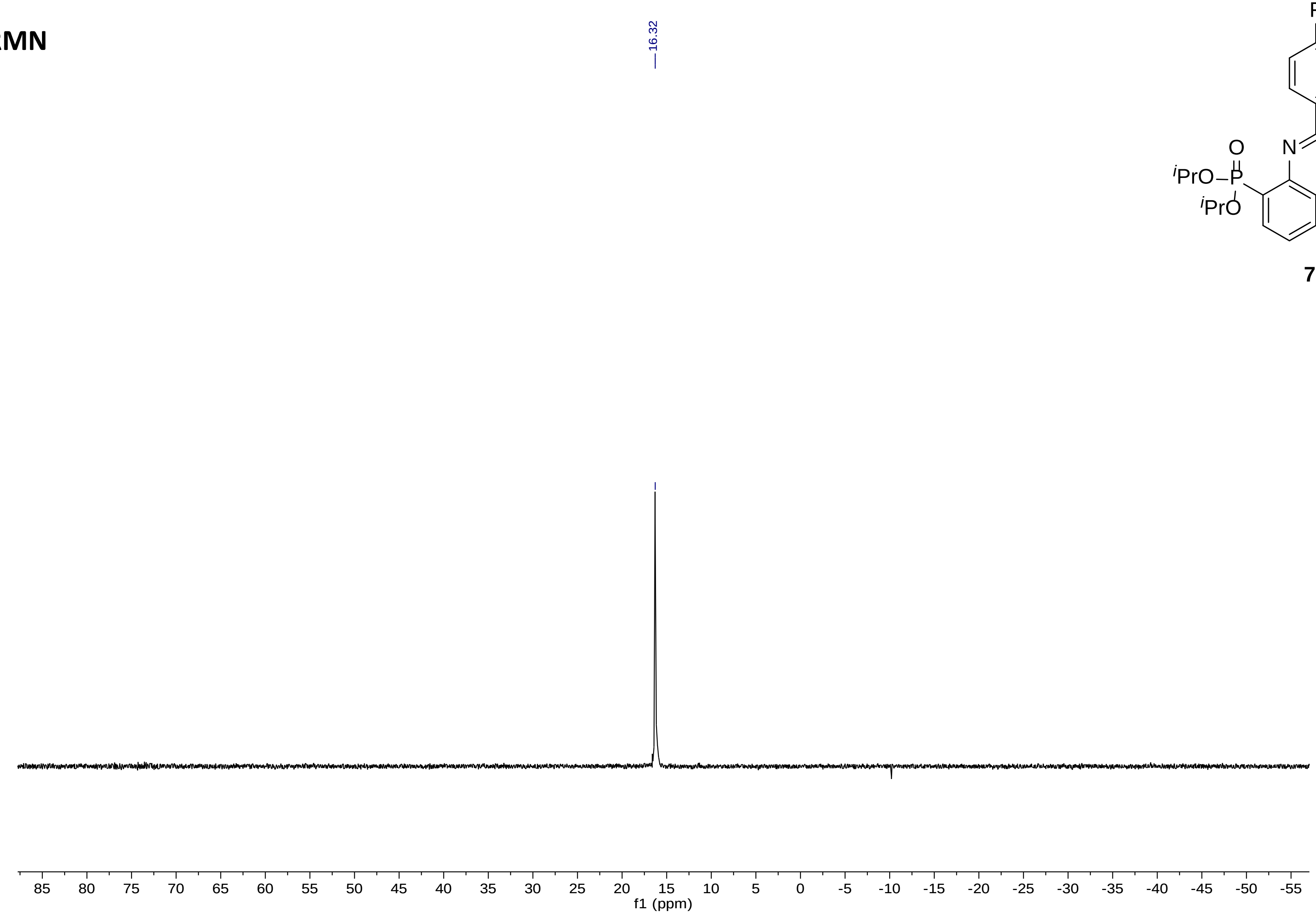
16.32
85
80
75
70
65
60
55
50
45
40
35
30
25
20
15
10
5
0
-5
-10
-15
-20
-25
-30
-35
-40
-45
-50
-55
f1 (ppm)

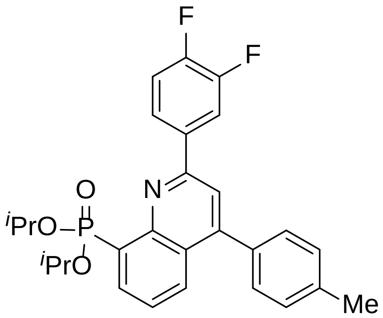
F
F
O
N
iPrO–P
iPrO
Me

7q

^{19}F-RMN

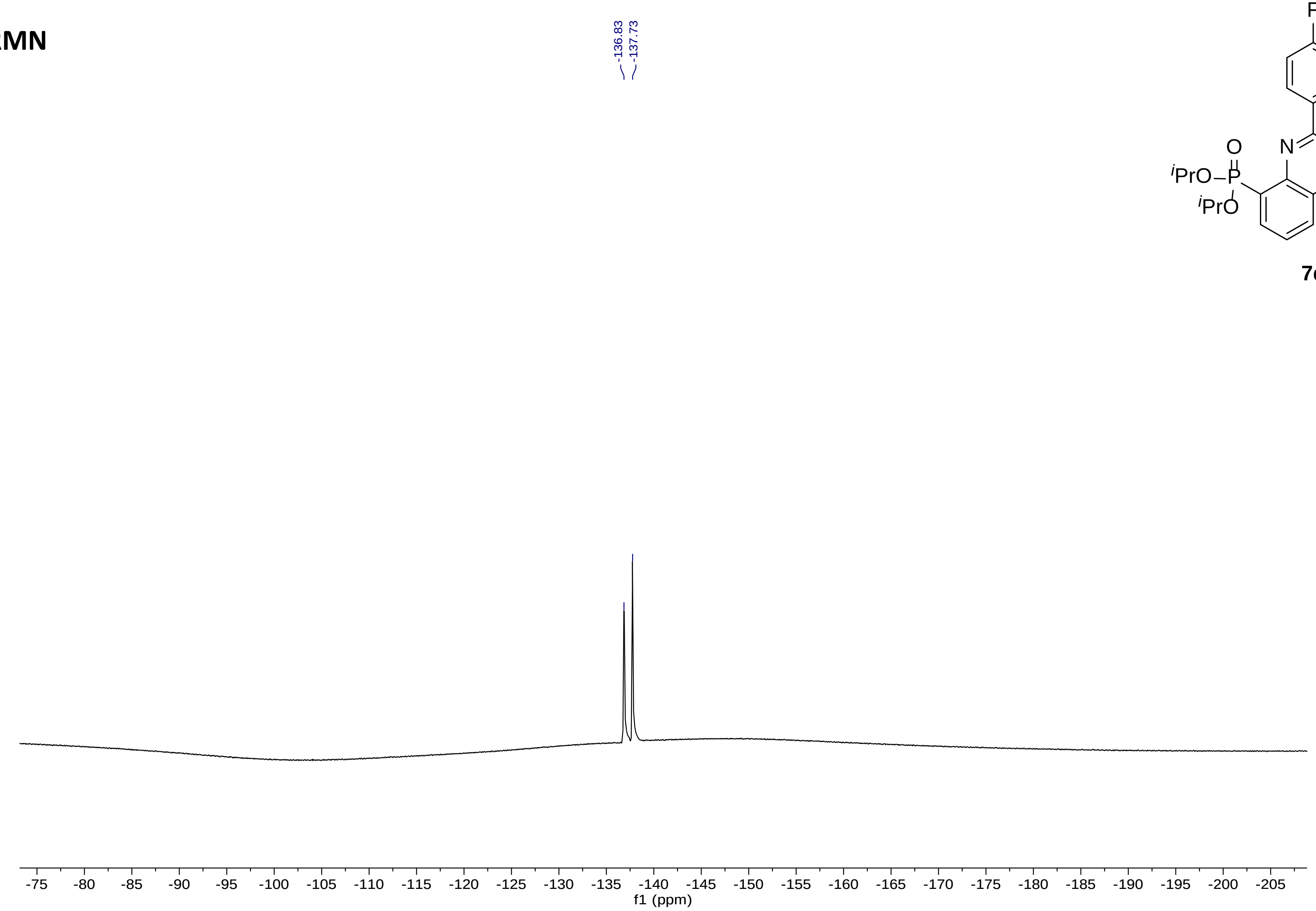

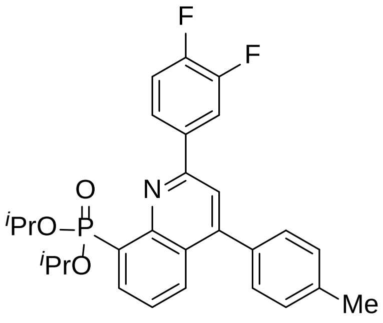

7q

^{1}H-RMN

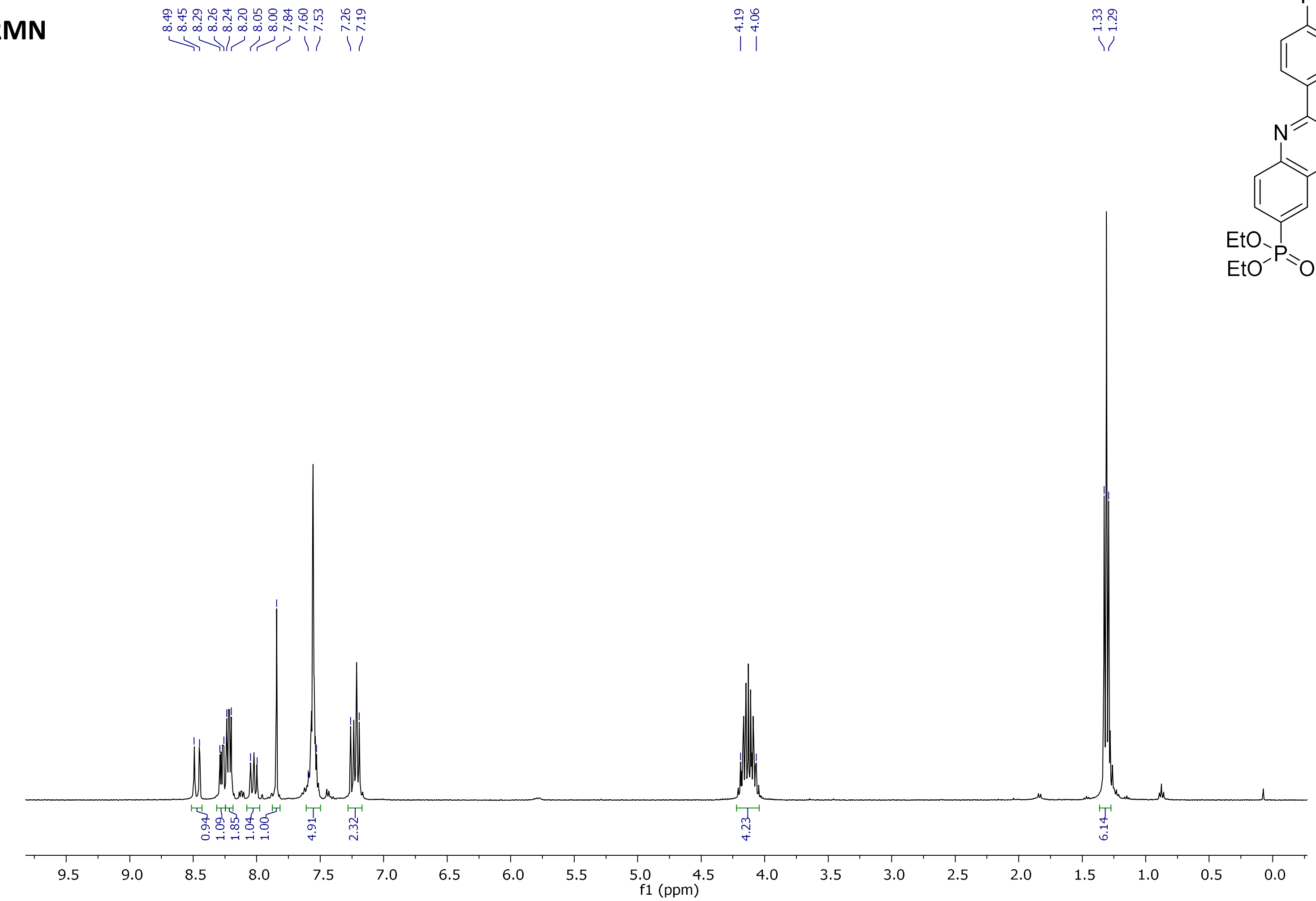

8.49
8.45
8.29
8.26
8.24
8.20
8.05
8.00
7.84
7.60
7.53
7.26
7.19
4.19
4.06
1.33
1.29
0.94
1.09
1.85
1.04
1.00
4.91
2.32
4.23
6.14
9.5
9.0
8.5
8.0
7.5
7.0
6.5
6.0
5.5
5.0
4.5
4.0
3.5
3.0
2.5
2.0
1.5
1.0
0.5
0.0
f1 (ppm)

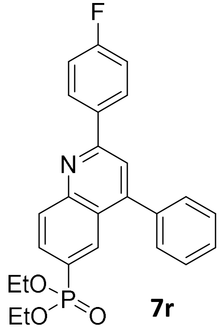

F
N
EtO
EtO
P
O

7r

^{13}C-RMN

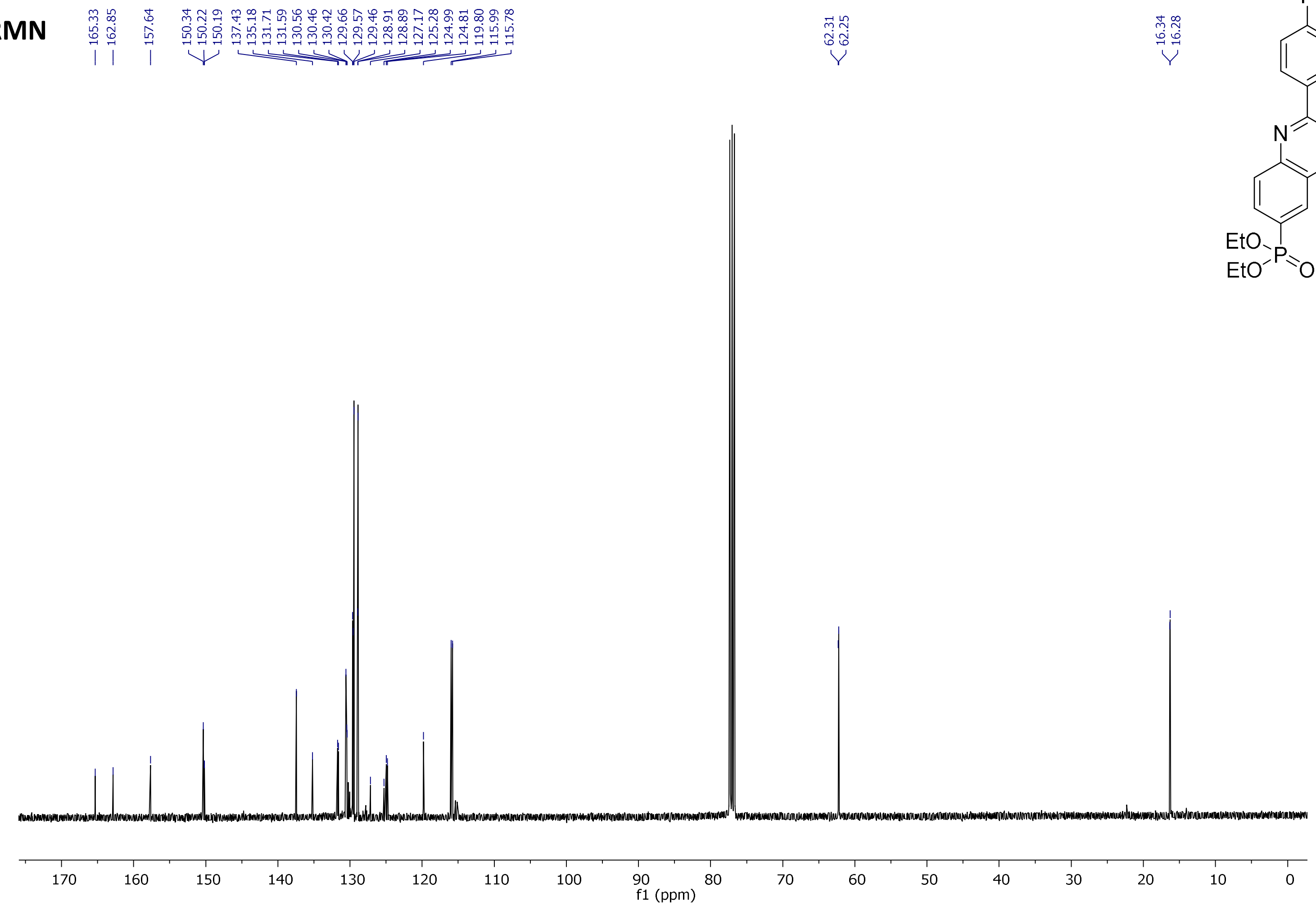
165.33
162.85
157.64
150.34
150.22
150.19
137.43
135.18
131.71
131.59
130.56
130.46
130.42
129.66
129.57
129.46
128.91
128.89
127.17
125.28
124.99
124.81
119.80
115.99
115.78
62.31
62.25
16.34
16.28
170
160
150
140
130
120
110
100
90
80
70
60
50
40
30
20
10
0
f1 (ppm)

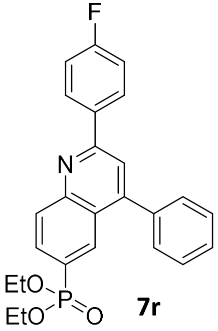
F
N
EtO
EtO
P
O

7r

^{31}P-RMN

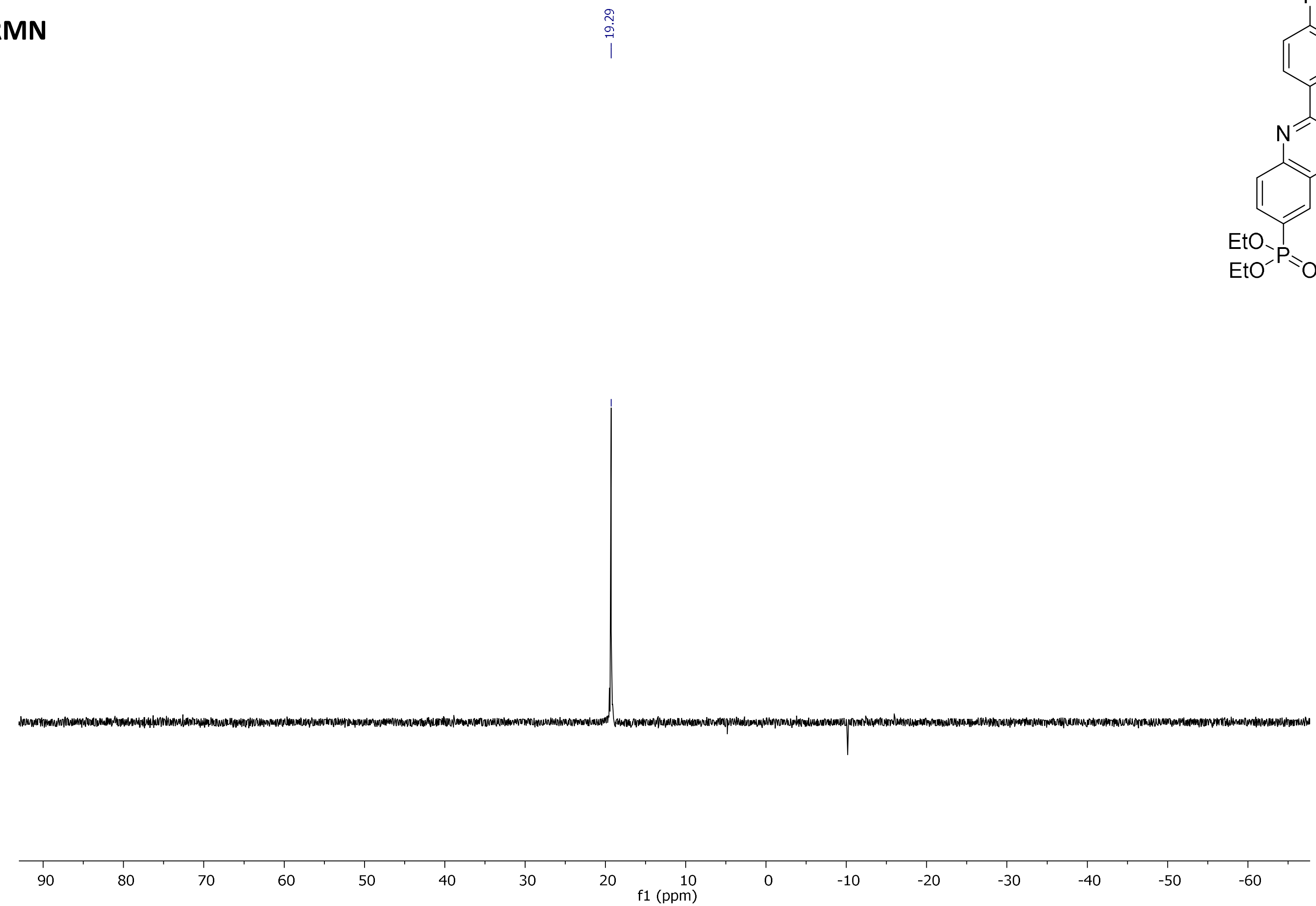

19.29
90
80
70
60
50
40
30
20
10
0
-10
-20
-30
-40
-50
-60
f1 (ppm)

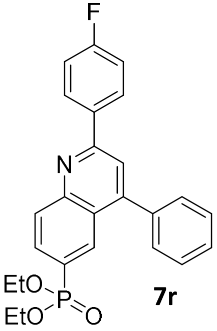

F
N
EtO
EtO
P
O

7r

^{19}F-RMN

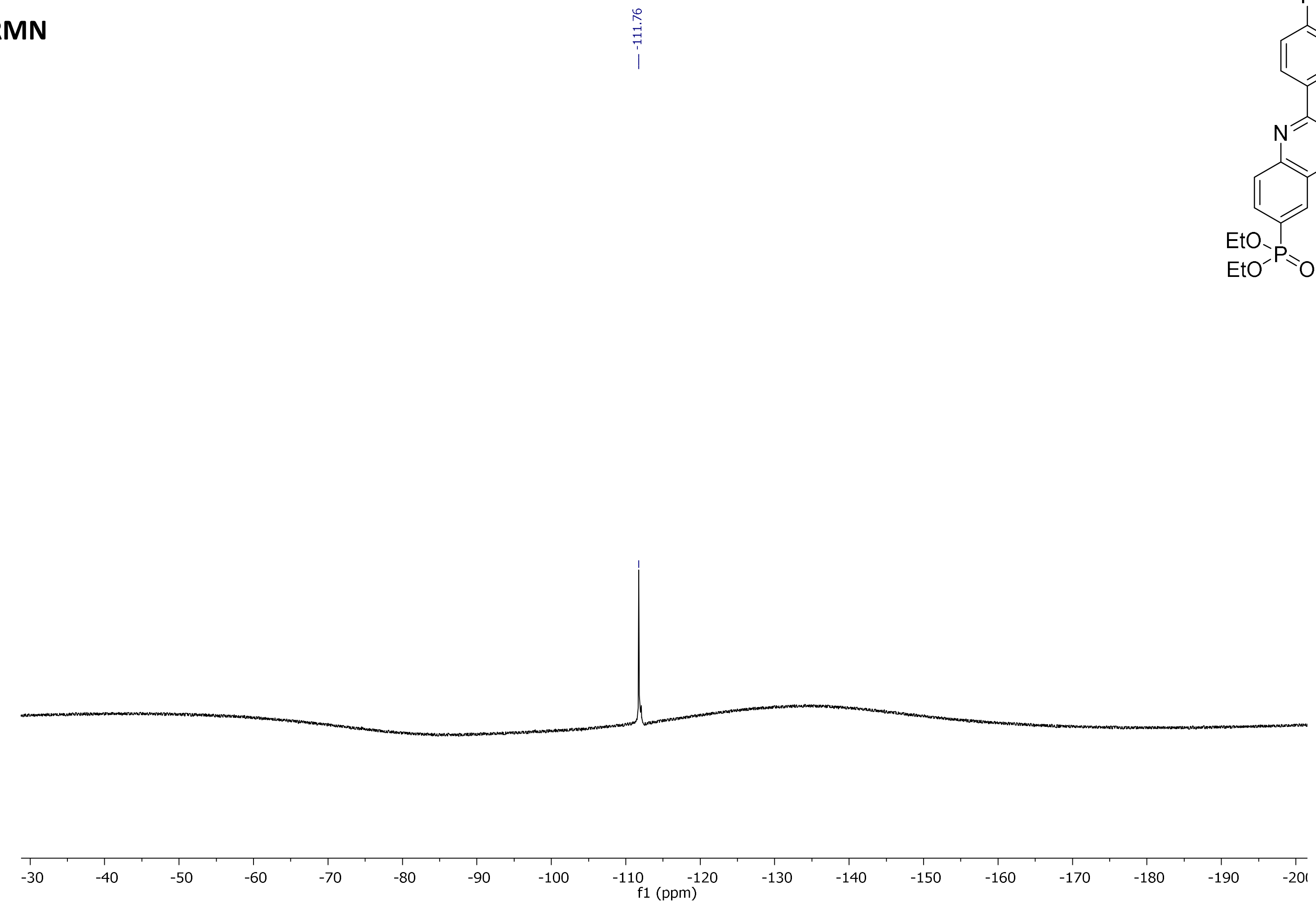
-111.76
-30
-40
-50
-60
-70
-80
-90
-100
-110
-120
-130
-140
-150
-160
-170
-180
-190
-200
f1 (ppm)

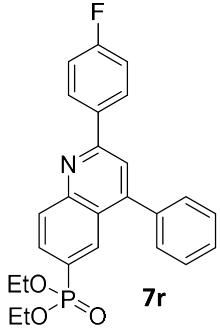
F
N
EtO
EtO
P
O

7r

^{1}H-RMN

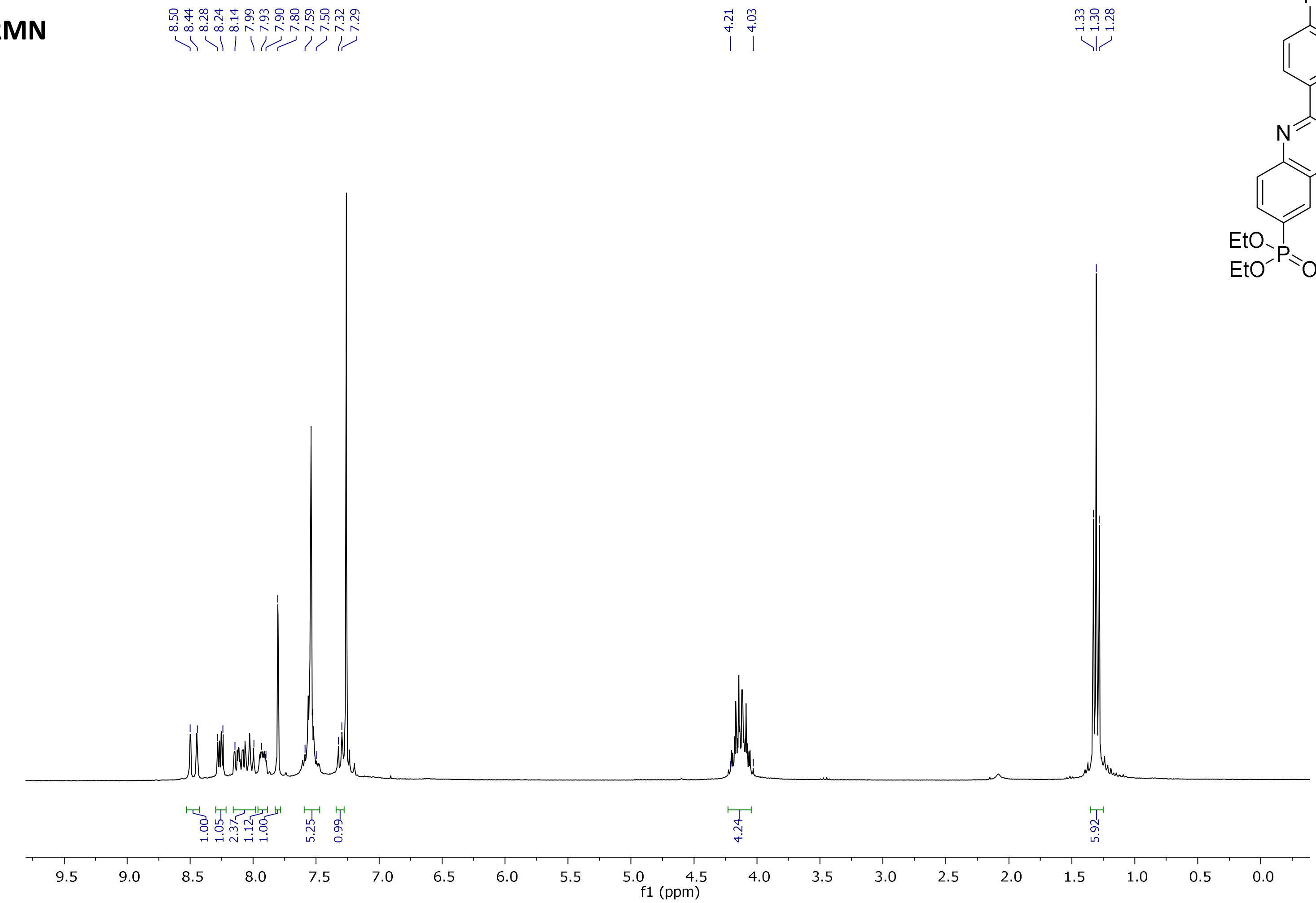
8.50
8.44
8.28
8.24
8.14
7.99
7.93
7.90
7.80
7.59
7.50
7.32
7.29
4.21
4.03
1.33
1.30
1.28
1.00
1.05
2.37
1.12
1.00
5.25
0.99
4.24
5.92
9.5
9.0
8.5
8.0
7.5
7.0
6.5
6.0
5.5
5.0
4.5
4.0
3.5
3.0
2.5
2.0
1.5
1.0
0.5
0.0
f1 (ppm)

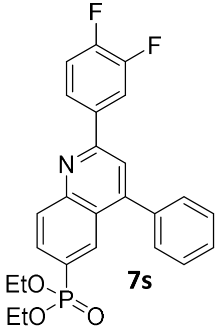
F
F
N
EtO
EtO
P
O

7s

^{13}C-RMN

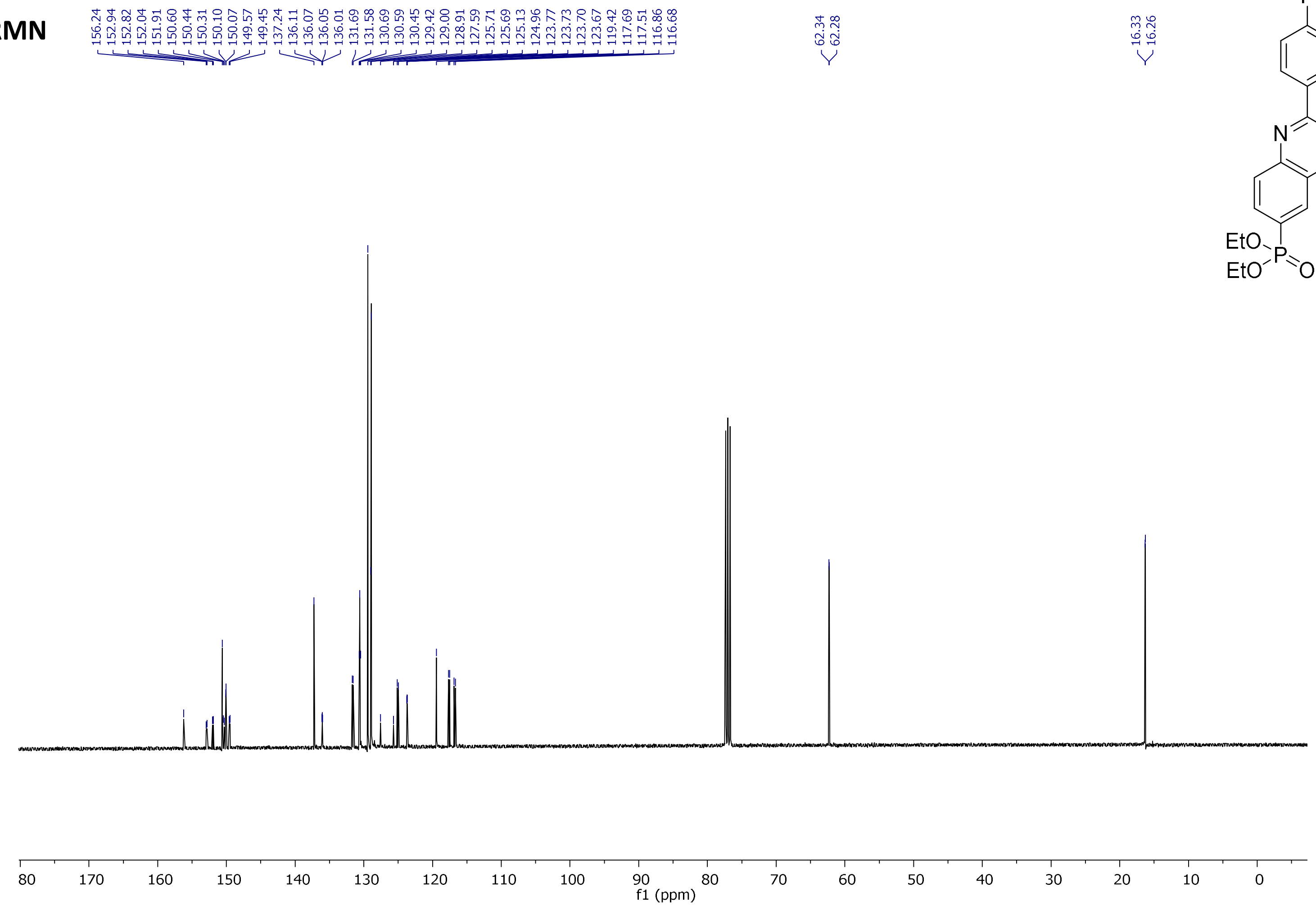
156.24
152.94
152.82
152.04
151.91
150.60
150.44
150.31
150.10
150.07
149.57
149.45
137.24
136.11
136.07
136.05
136.01
131.69
131.58
130.69
130.59
130.45
129.42
129.00
128.91
127.59
125.71
125.69
125.13
124.96
123.77
123.73
123.70
123.67
119.42
117.69
117.51
116.86
116.68
62.34
62.28
16.33
16.26
f1 (ppm)

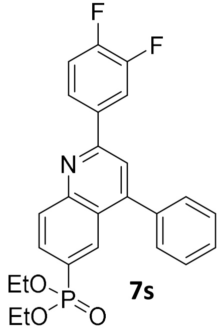
F
F
N
EtO
EtO
P
O

7s

^{31}P-RMN

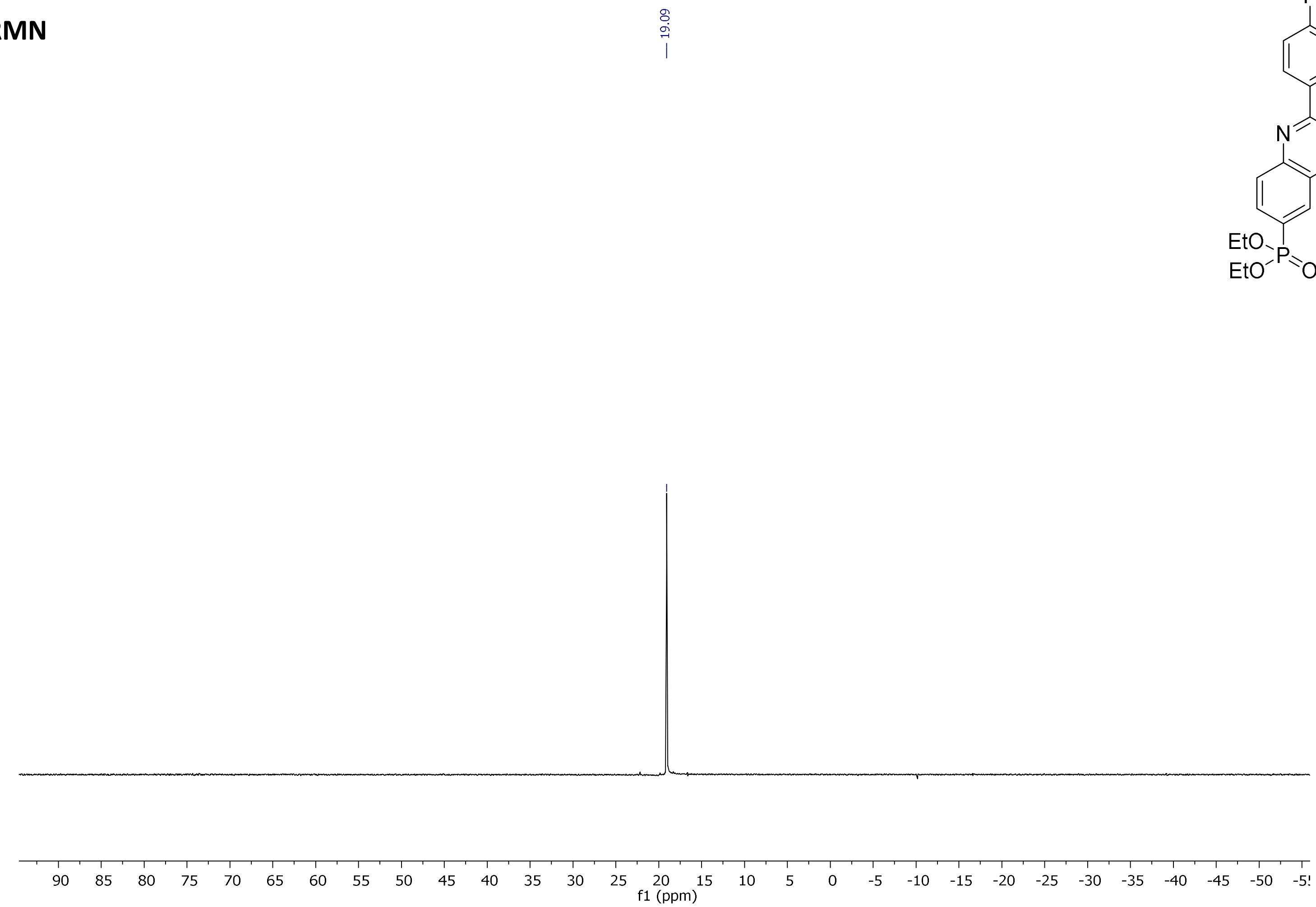

19.09
90 85 80 75 70 65 60 55 50 45 40 35 30 25 20 15 10 5 0 -5 -10 -15 -20 -25 -30 -35 -40 -45 -50
f1 (ppm)

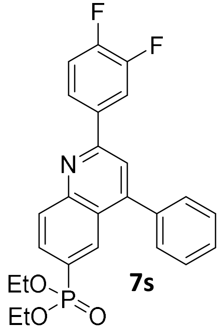

F
F
N
EtO
EtO
P
O

7s

^{19}F-RMN

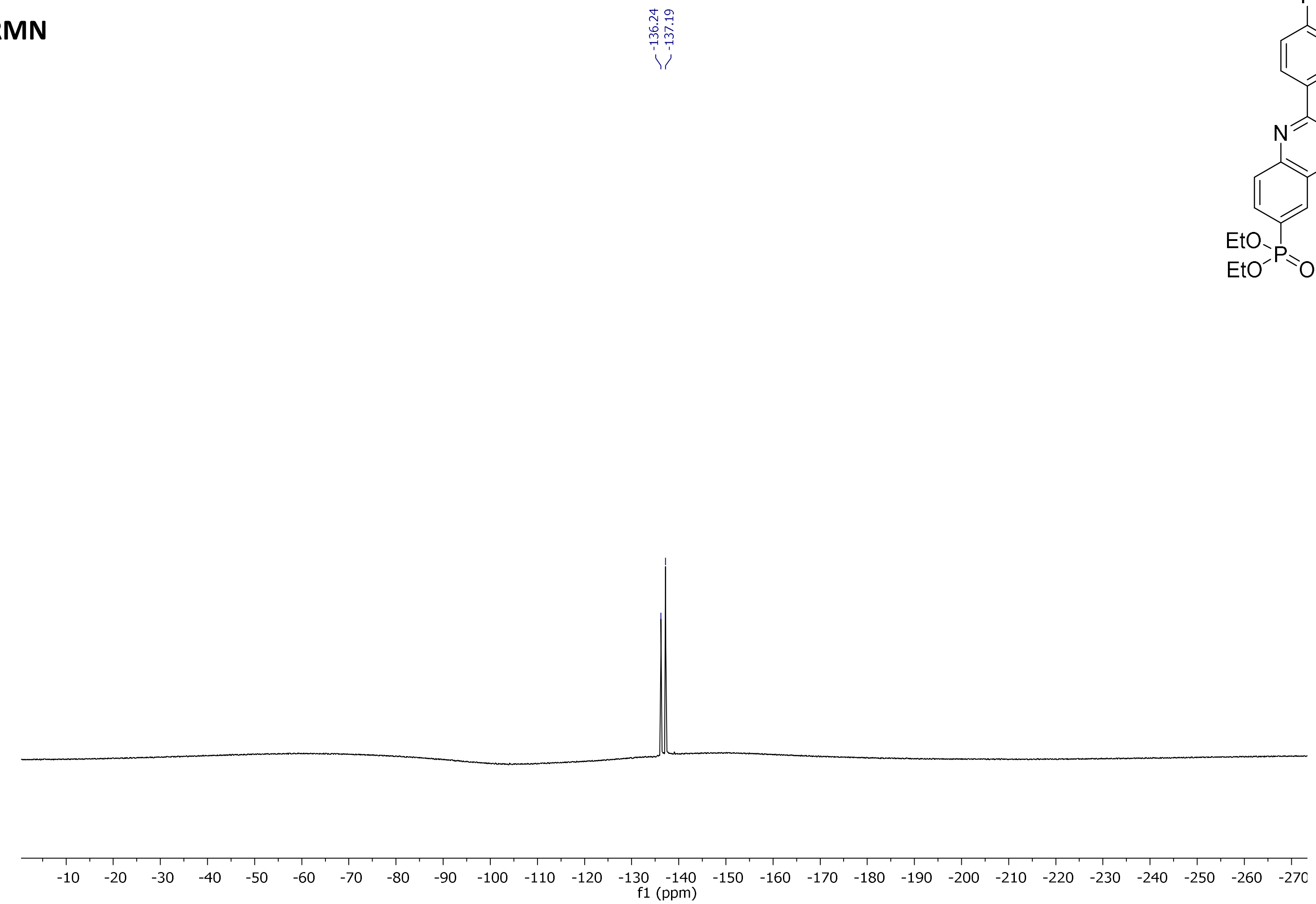

-136.24
-137.19
-10 -20 -30 -40 -50 -60 -70 -80 -90 -100 -110 -120 -130 -140 -150 -160 -170 -180 -190 -200 -210 -220 -230 -240 -250 -260 -270
f1 (ppm)

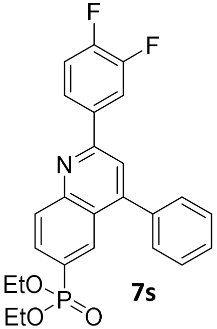

F
F
N
EtO
EtO
P
O

7s

^{1}H-RMN

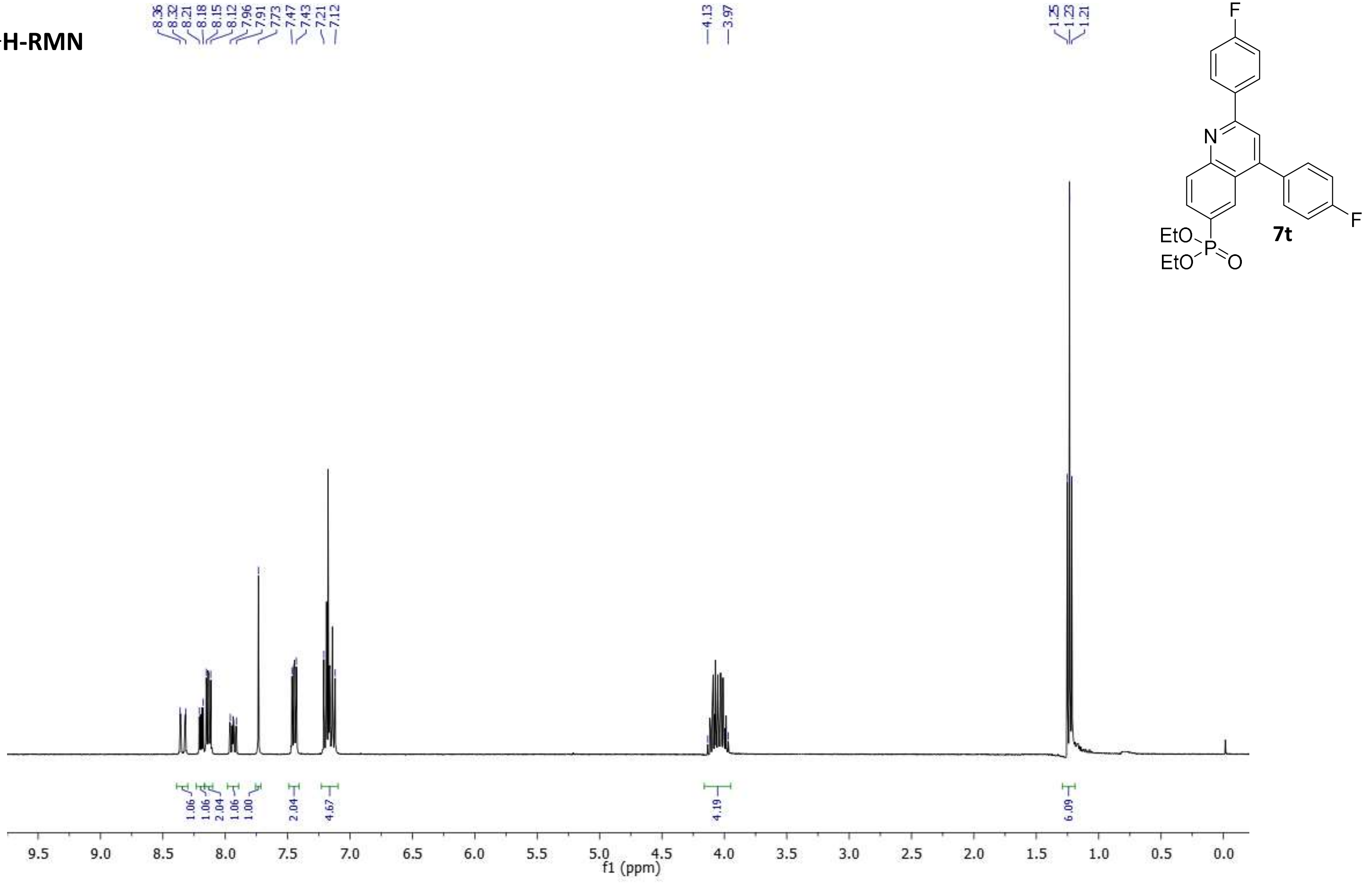
8.36
8.32
8.21
8.18
8.15
8.12
7.96
7.91
7.73
7.47
7.43
7.21
7.12
4.13
3.97
1.25
1.23
1.21
1.06
1.06
2.04
1.06
1.00
2.04
4.67
4.19
6.09
9.5
9.0
8.5
8.0
7.5
7.0
6.5
6.0
5.5
5.0
4.5
4.0
3.5
3.0
2.5
2.0
1.5
1.0
0.5
0.0
f1 (ppm)
F
N
F
EtO
EtO
P
O

7t

^{13}C-RMN

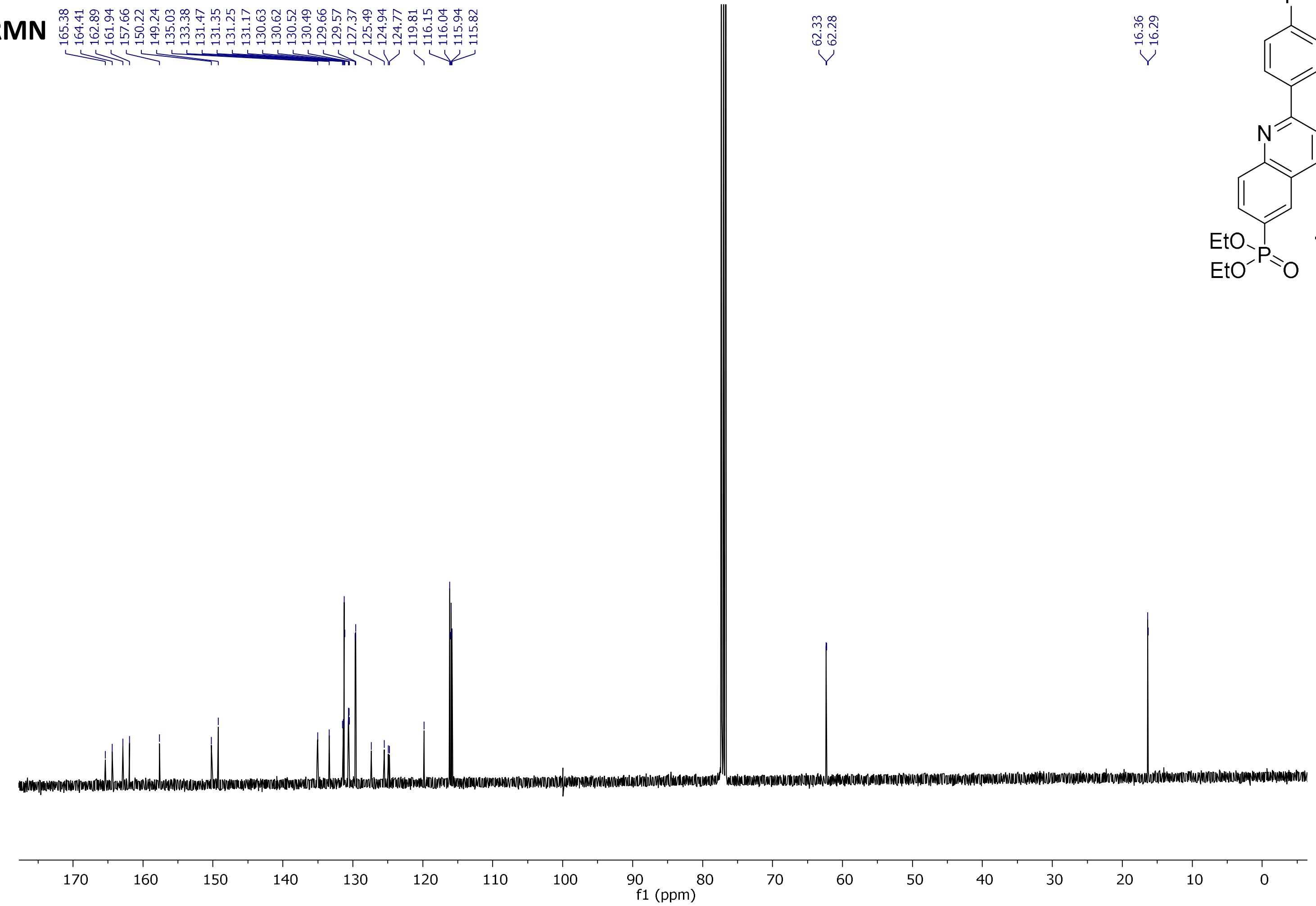
165.38
164.41
162.89
161.94
157.66
150.22
149.24
135.03
133.38
131.47
131.35
131.25
131.17
130.63
130.62
130.52
130.49
129.66
129.57
127.37
125.49
124.94
124.77
119.81
116.15
116.04
115.94
115.82
62.33
62.28
16.36
16.29
170
160
150
140
130
120
110
100
90
80
70
60
50
40
30
20
10
0
f1 (ppm)

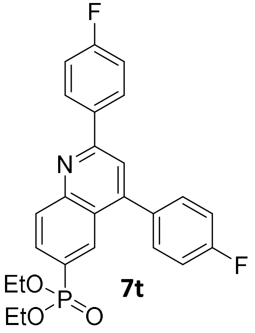
F
N
F
EtO
EtO
P
O

7t

^{31}P-RMN

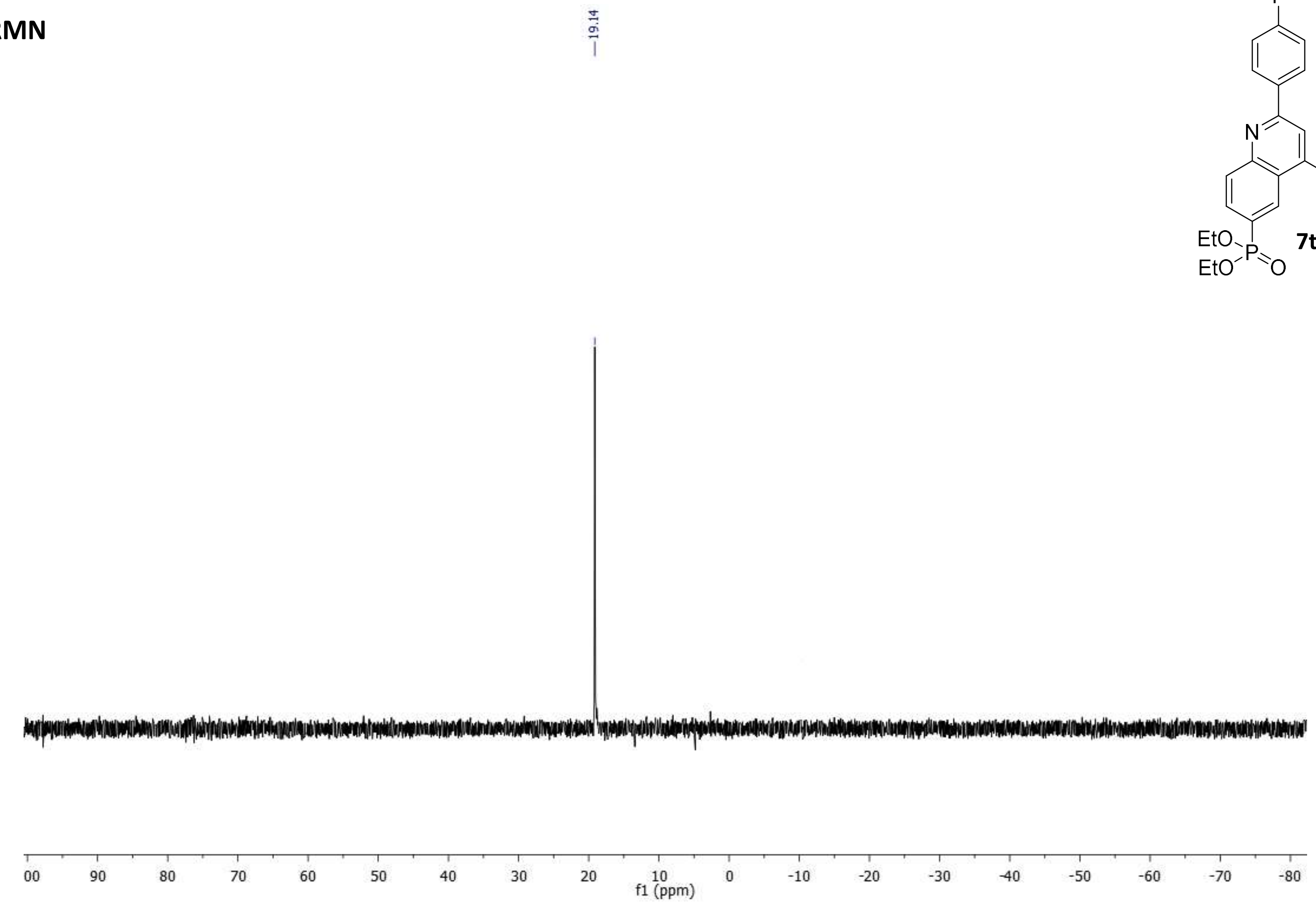

19.14
00
90
80
70
60
50
40
30
20
10
0
-10
-20
-30
-40
-50
-60
-70
-80
f1 (ppm)

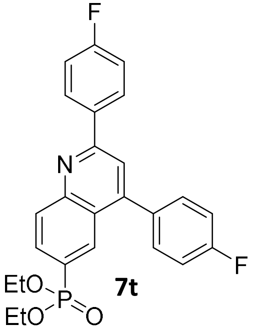

F
N
F
EtO
EtO
P
O

7t

^{19}F-RMN

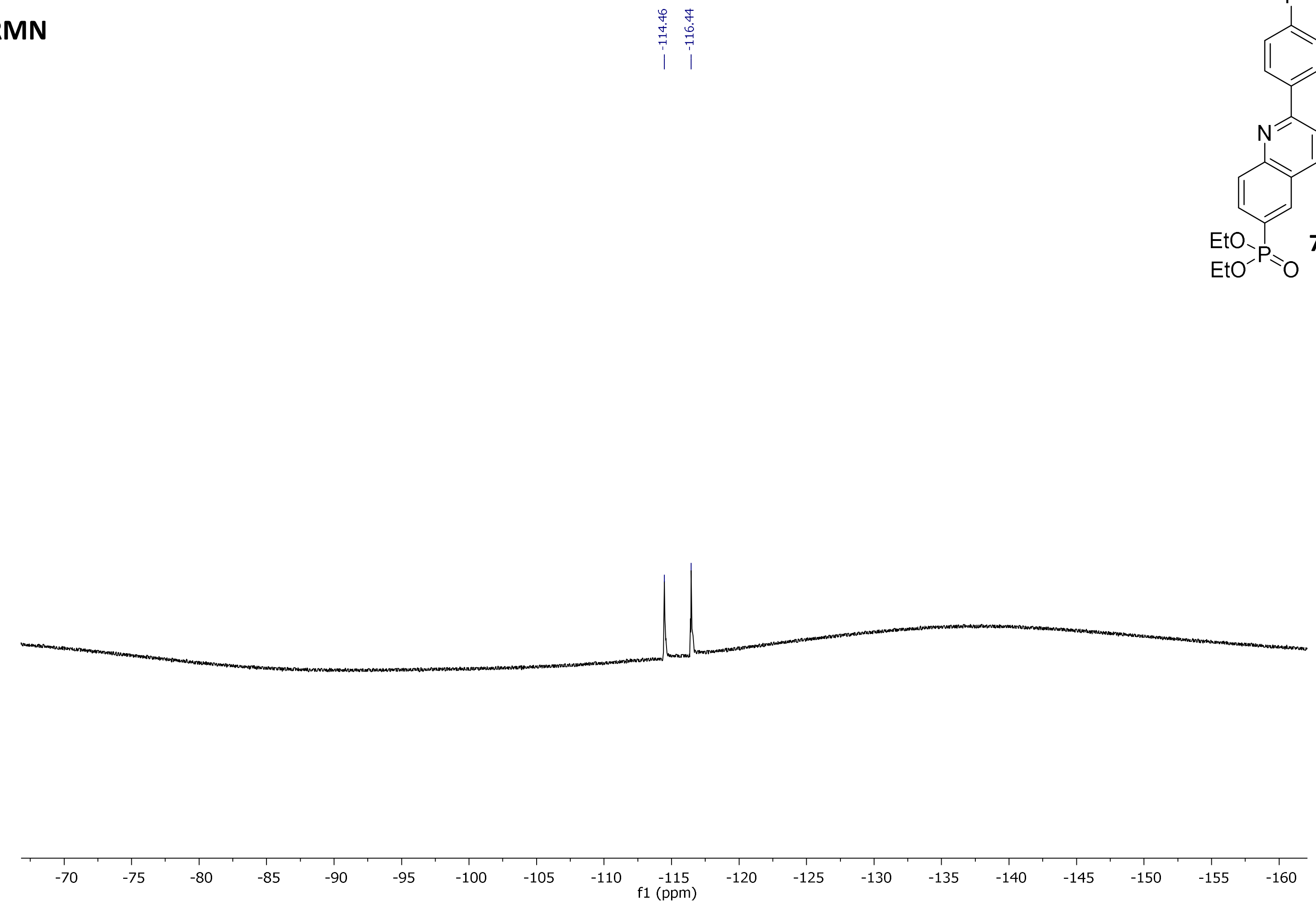

-114.46
-116.44
-70
-75
-80
-85
-90
-95
-100
-105
-110
-115
-120
-125
-130
-135
-140
-145
-150
-155
-160
f1 (ppm)

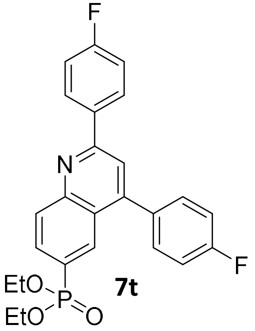

F
N
F
EtO
EtO
P
O

7t

^{1}H-RMN

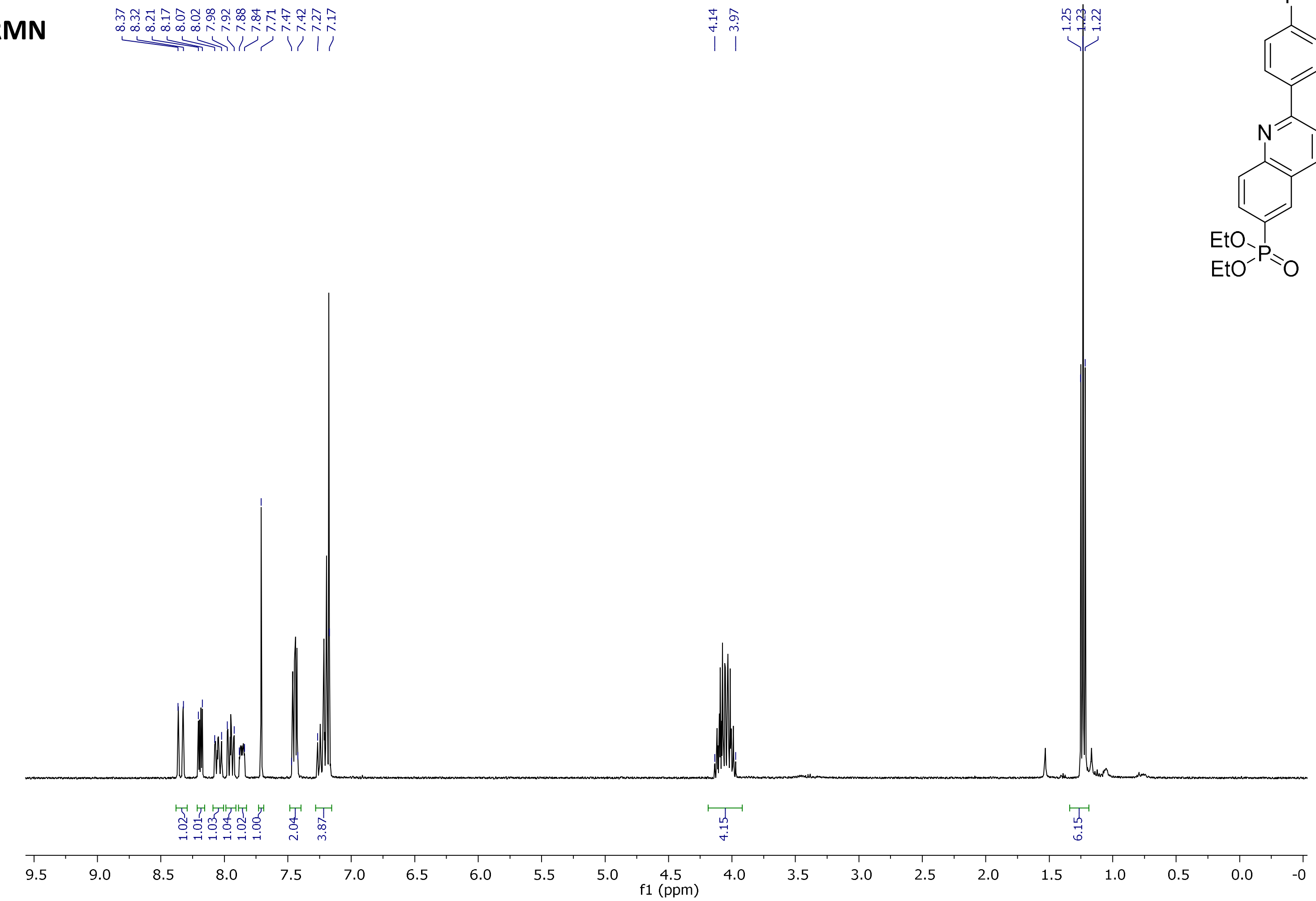

8.37
8.32
8.21
8.17
8.07
8.02
7.98
7.92
7.88
7.84
7.71
7.47
7.42
7.27
7.17
4.14
3.97
1.25
1.23
1.22
1.02
1.01
1.03
1.04
1.02
1.00
2.04
3.87
4.15
6.15
9.5
9.0
8.5
8.0
7.5
7.0
6.5
6.0
5.5
5.0
4.5
4.0
3.5
3.0
2.5
2.0
1.5
1.0
0.5
0.0
-0
f1 (ppm)

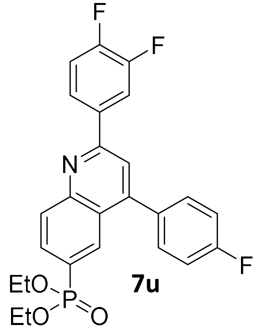

F
F
N
F
EtO
EtO
P
O

7u

^{13}C-RMN

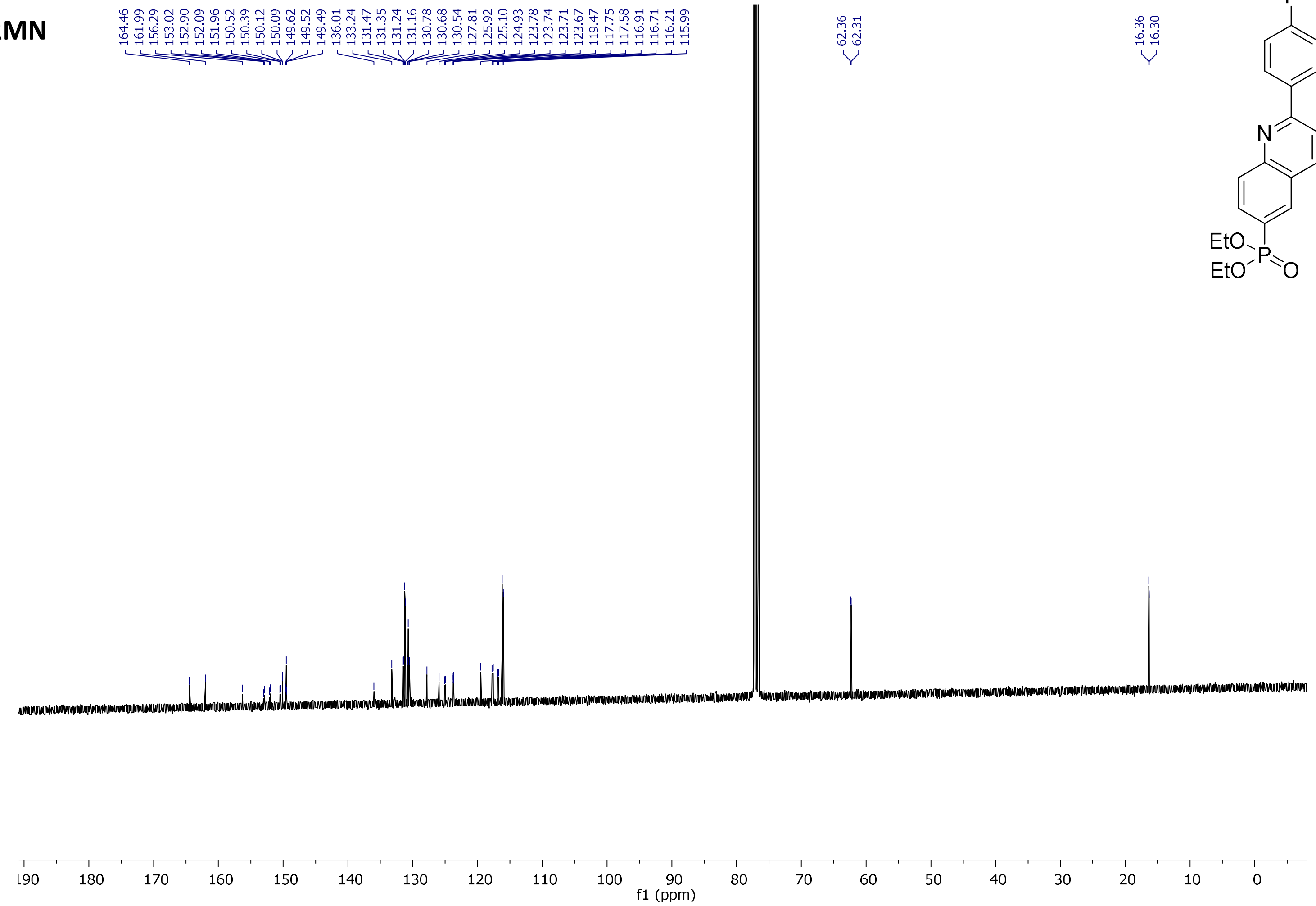
164.46
161.99
156.29
153.02
152.90
152.09
151.96
150.52
150.39
150.12
150.09
149.62
149.52
149.49
136.01
133.24
131.47
131.35
131.24
131.16
130.78
130.68
130.54
127.81
125.92
125.10
124.93
123.78
123.74
123.71
123.67
119.47
117.75
117.58
116.91
116.71
116.21
115.99
62.36
62.31
16.36
16.30
190 180 170 160 150 140 130 120 110 100 90 80 70 60 50 40 30 20 10 0
f1 (ppm)

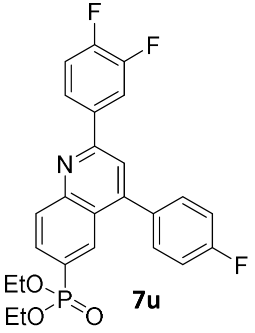
F
F
N
F
EtO
EtO
P
O

7u

^{31}P-RMN

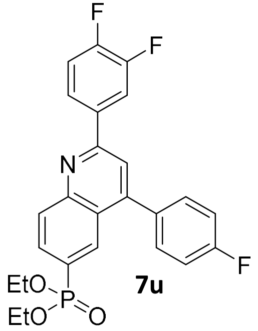

F
F
N
F
EtO
EtO
P
O

7u

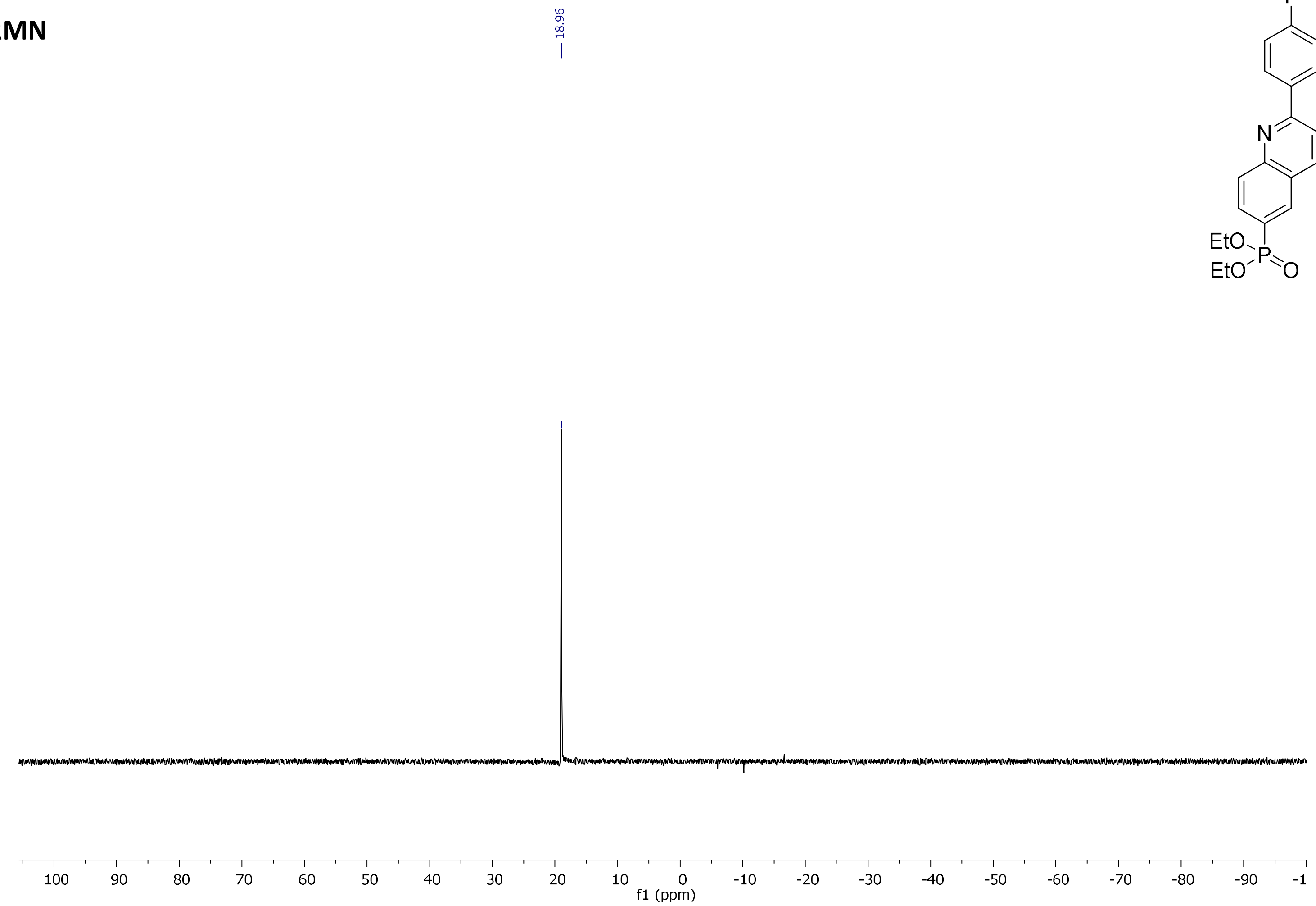

18.96
100
90
80
70
60
50
40
30
20
10
0
-10
-20
-30
-40
-50
-60
-70
-80
-90
-1
f1 (ppm)

^{19}F-RMN

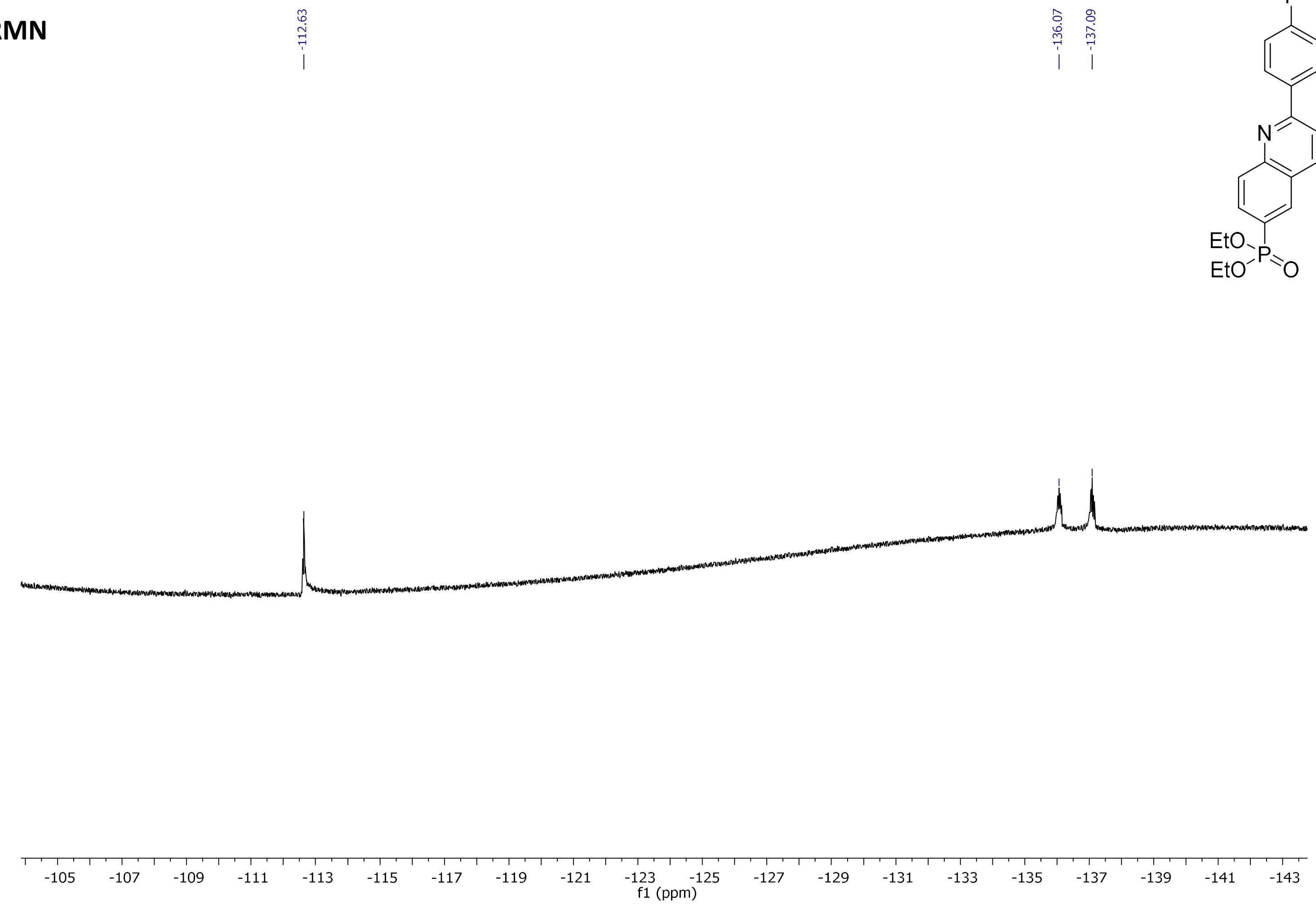

-112.63
-136.07
-137.09
-105
-107
-109
-111
-113
-115
-117
-119
-121
-123
-125
-127
-129
-131
-133
-135
-137
-139
-141
-143
f1 (ppm)

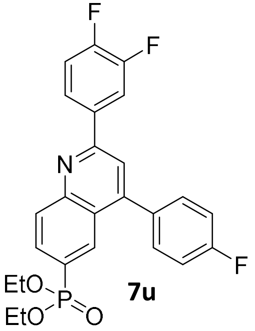

F
F
N
F
EtO
EtO
P
O

7u

¹H-RMN

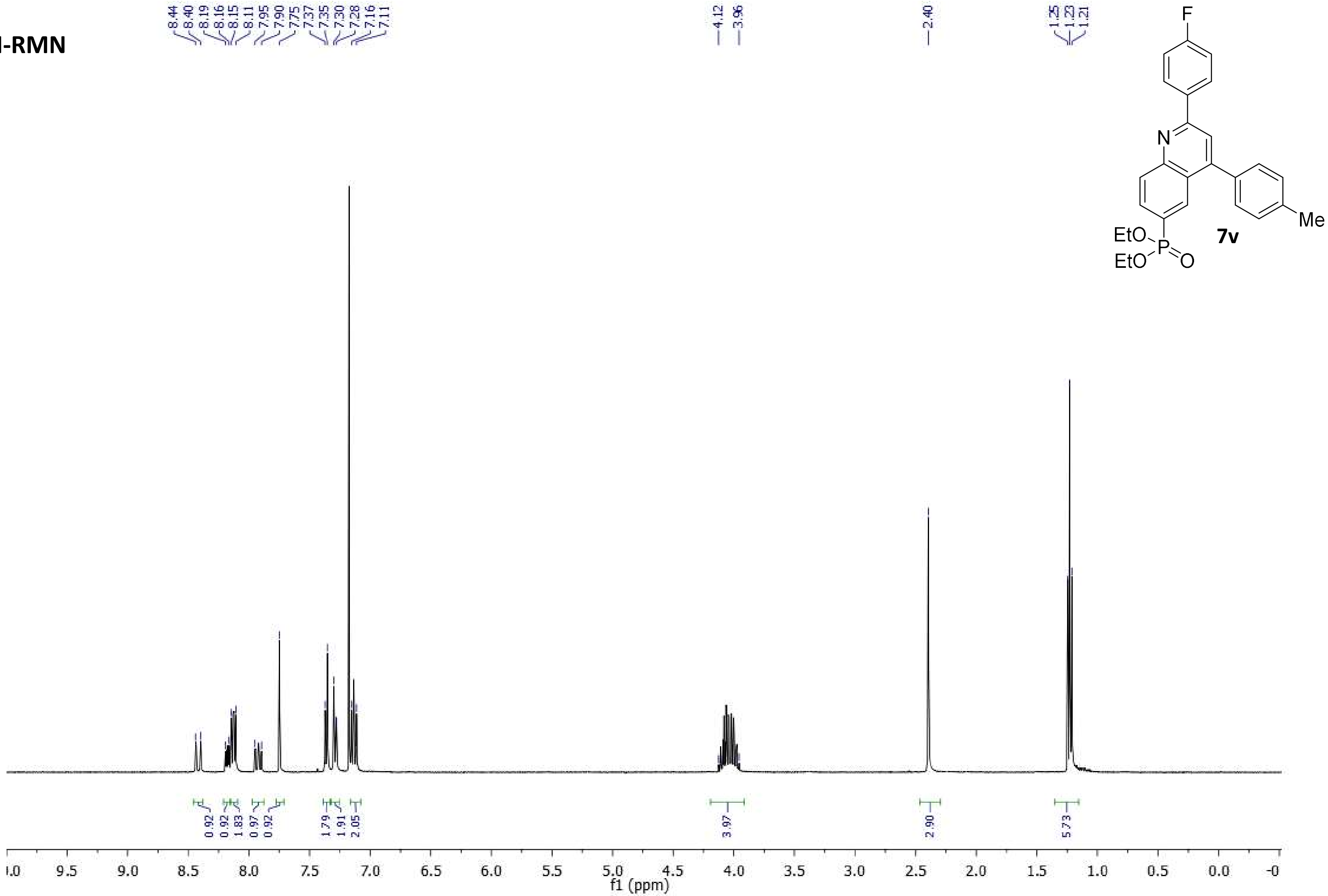

8.44
8.40
8.19
8.16
8.15
8.11
7.95
7.90
7.75
7.37
7.35
7.30
7.28
7.16
7.11
4.12
3.96
2.40
1.25
1.23
1.21
0.92
0.92
1.83
0.97
0.92
1.79
1.91
2.05
3.97
2.90
5.73
f1 (ppm)
F
N
Me
EtO
EtO
P
O

7v

^{13}C-RMN

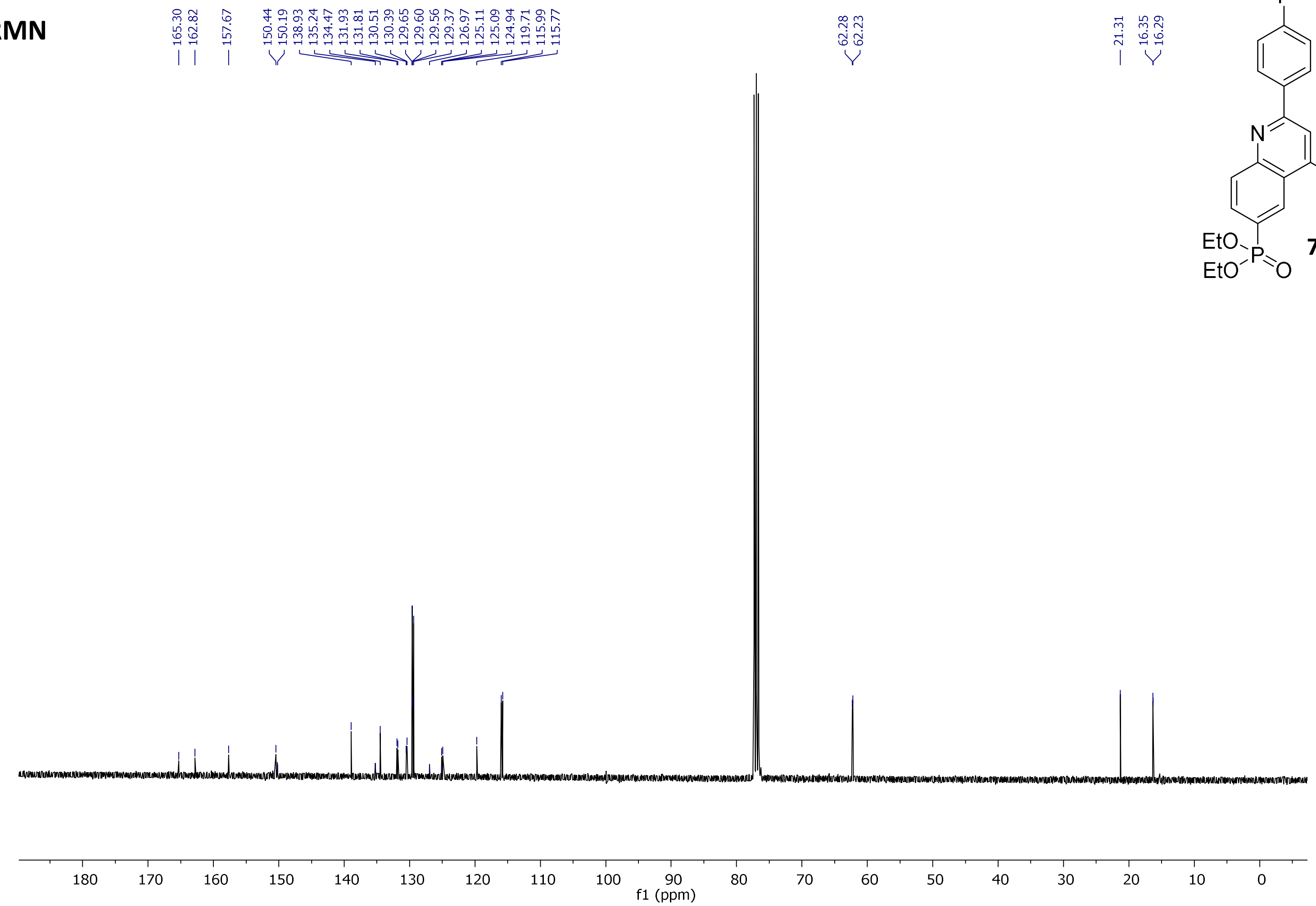

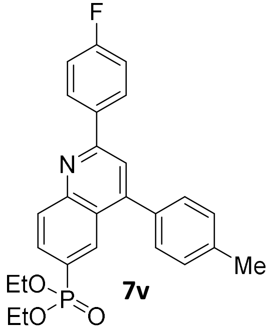

7v

^{31}P-RMN

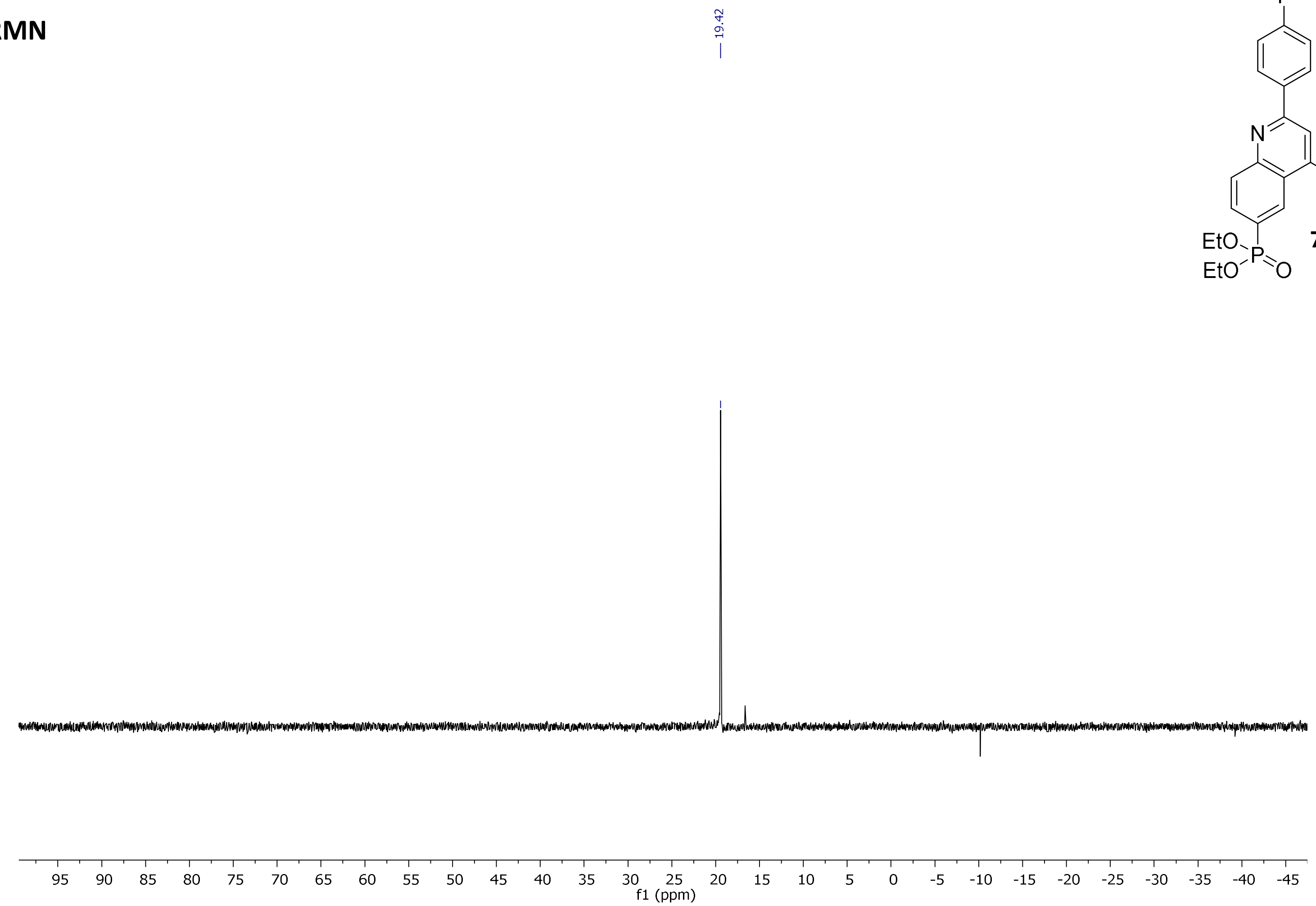
19.42
95
90
85
80
75
70
65
60
55
50
45
40
35
30
25
20
15
10
5
0
-5
-10
-15
-20
-25
-30
-35
-40
-45
f1 (ppm)

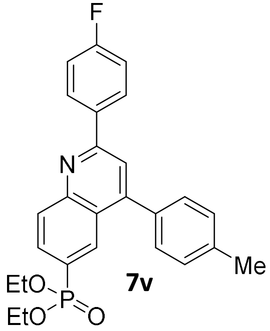
F
N
Me
EtO
EtO
P
O

7v

^{19}F-RMN

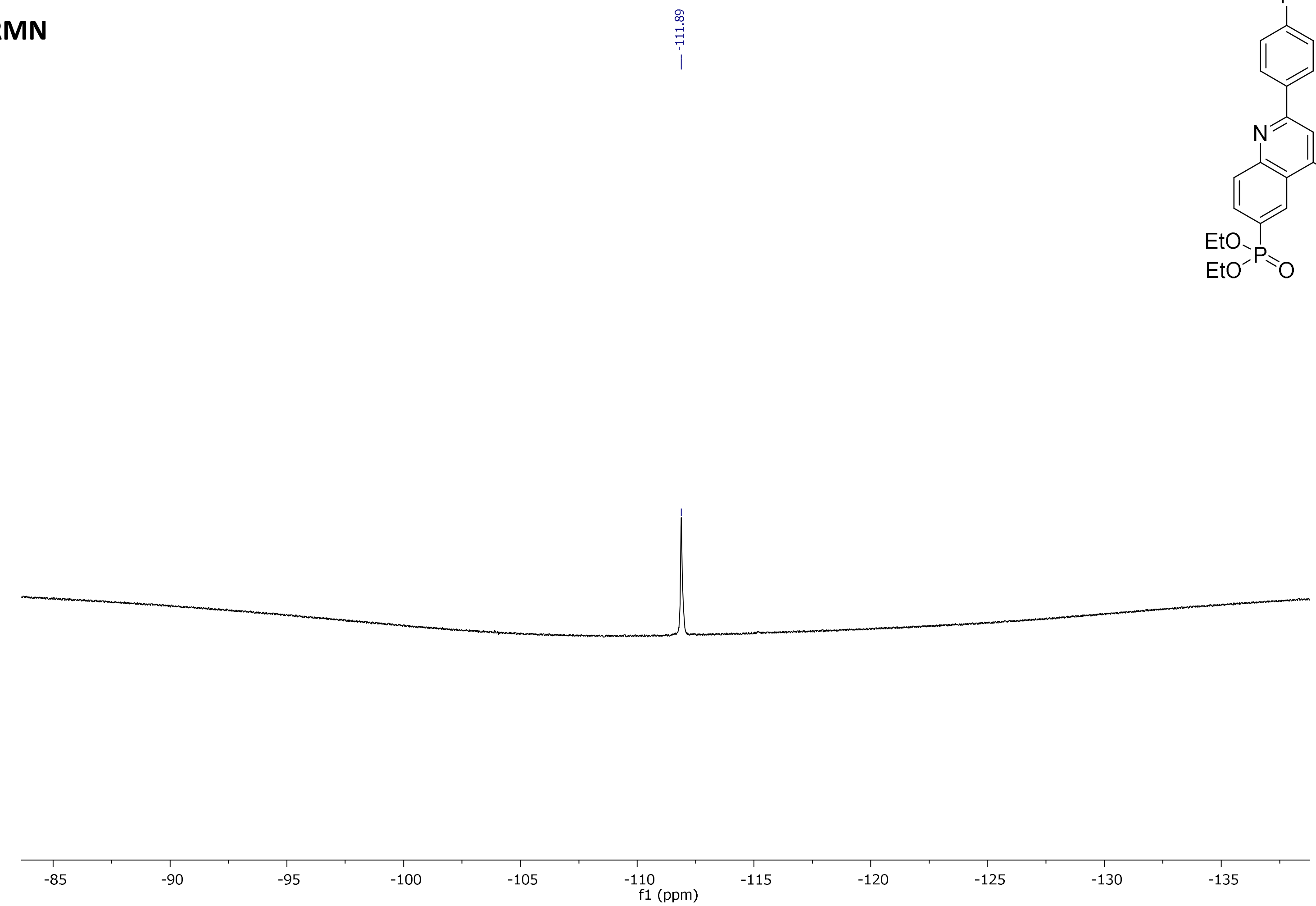
-111.89
-85
-90
-95
-100
-105
-110
-115
-120
-125
-130
-135
f1 (ppm)

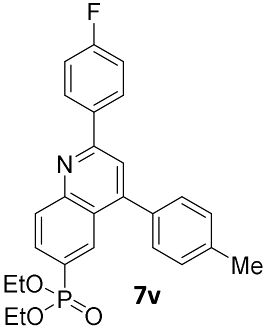
F
N
Me
EtO
EtO
P
O

7v

^{1}H-RMN

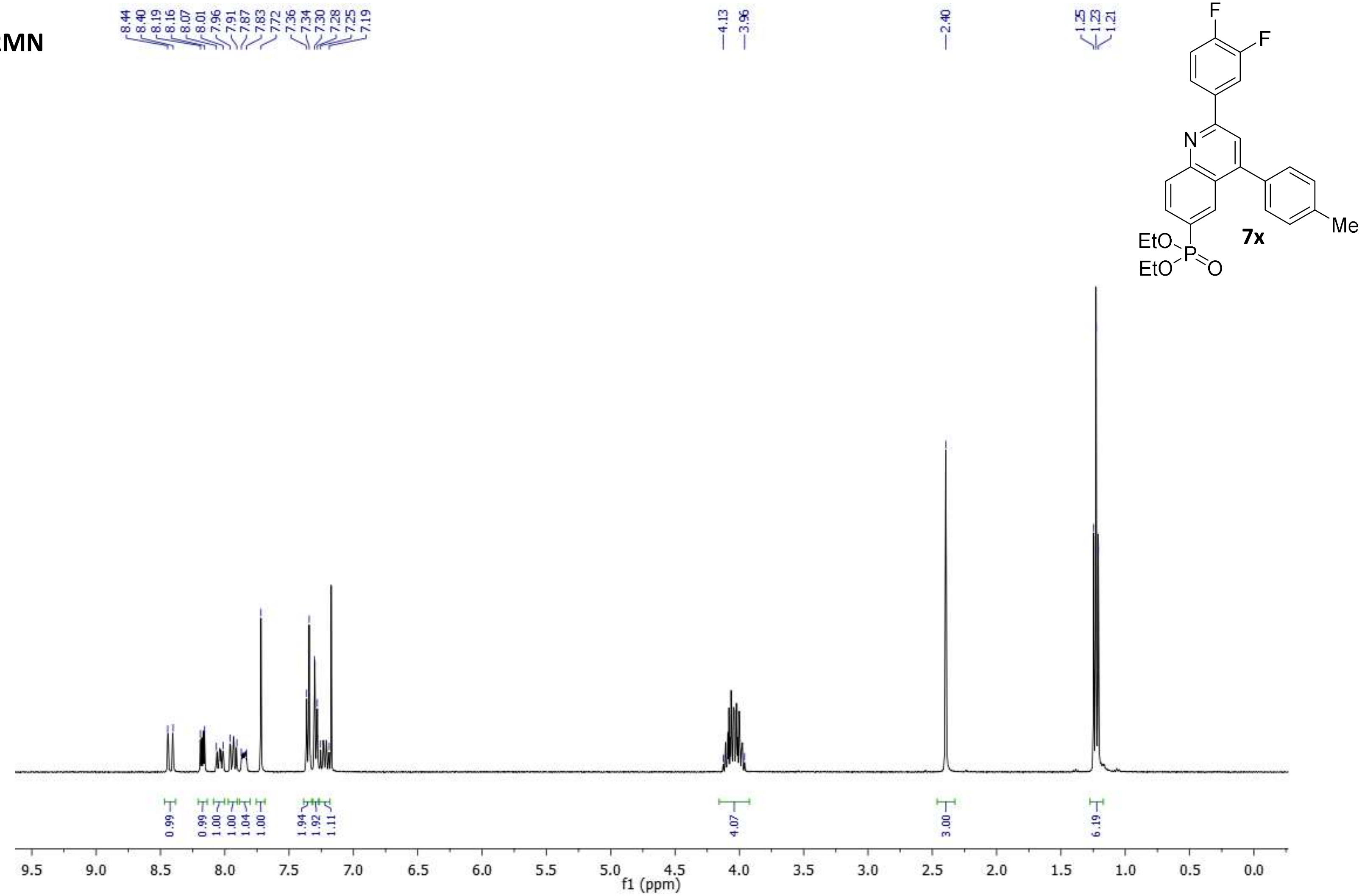

8.44
8.40
8.19
8.16
8.07
8.01
7.96
7.91
7.87
7.83
7.72
7.36
7.34
7.30
7.28
7.25
7.19
4.13
3.96
2.40
1.25
1.23
1.21
F
F
N
Me
EtO
EtO
P
O
7x
0.99
0.99
1.00
1.00
1.04
1.00
1.94
1.92
1.11
4.07
3.00
6.19
9.5
9.0
8.5
8.0
7.5
7.0
6.5
6.0
5.5
5.0
4.5
4.0
3.5
3.0
2.5
2.0
1.5
1.0
0.5
0.0
f1 (ppm)

^{13}C-RMN

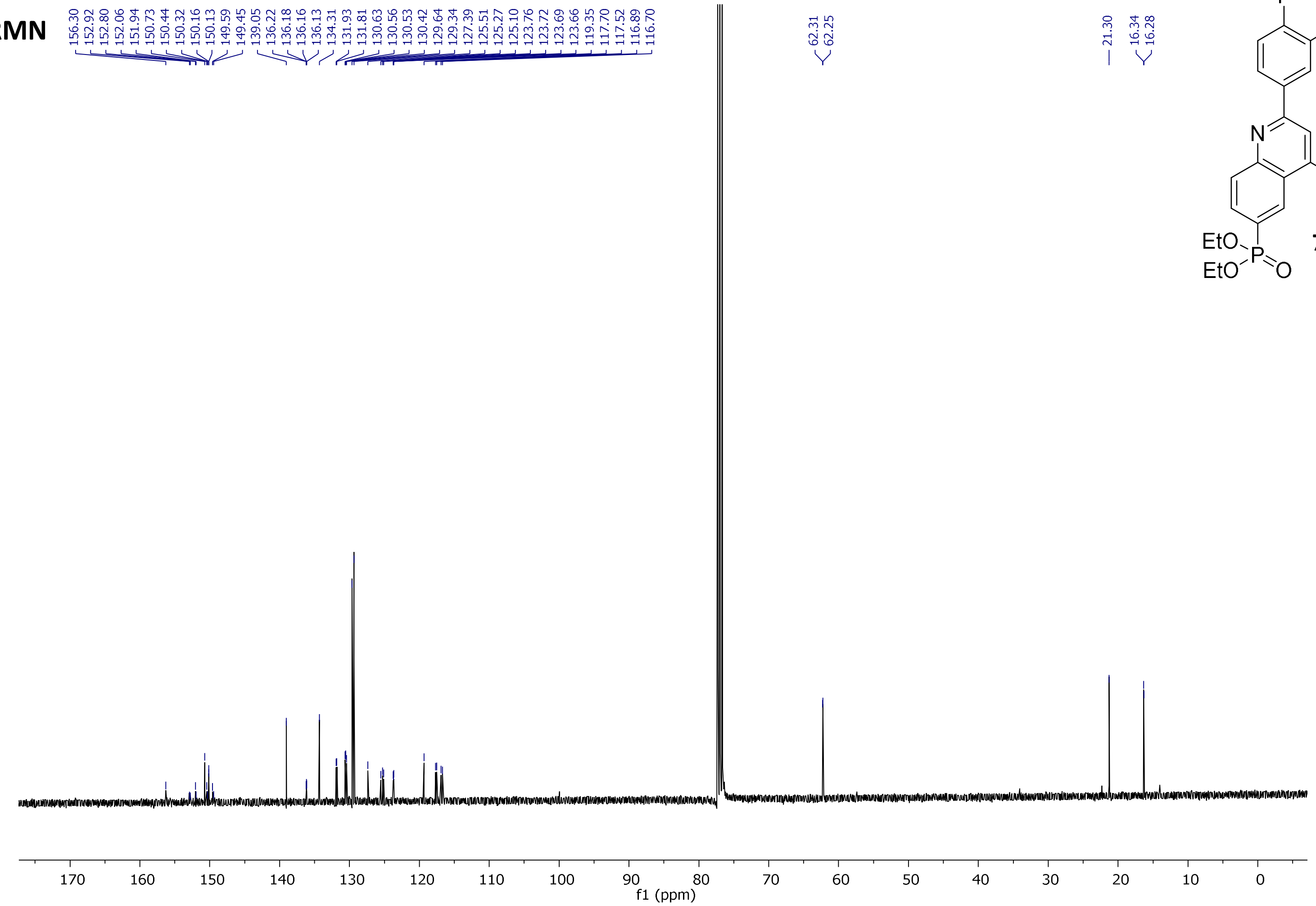
156.30
152.92
152.80
152.06
151.94
150.73
150.44
150.32
150.16
150.13
149.59
149.45
139.05
136.22
136.18
136.16
136.13
134.31
131.93
131.81
130.63
130.56
130.53
130.42
129.64
129.34
127.39
125.51
125.27
125.10
123.76
123.72
123.69
123.66
119.35
117.70
117.52
116.89
116.70
62.31
62.25
21.30
16.34
16.28
170 160 150 140 130 120 110 100 90 80 70 60 50 40 30 20 10 0
f1 (ppm)

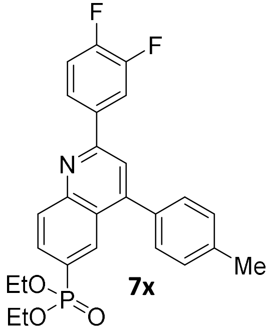
F
F
N
Me
EtO
EtO
P
O

7x

^{31}P-RMN

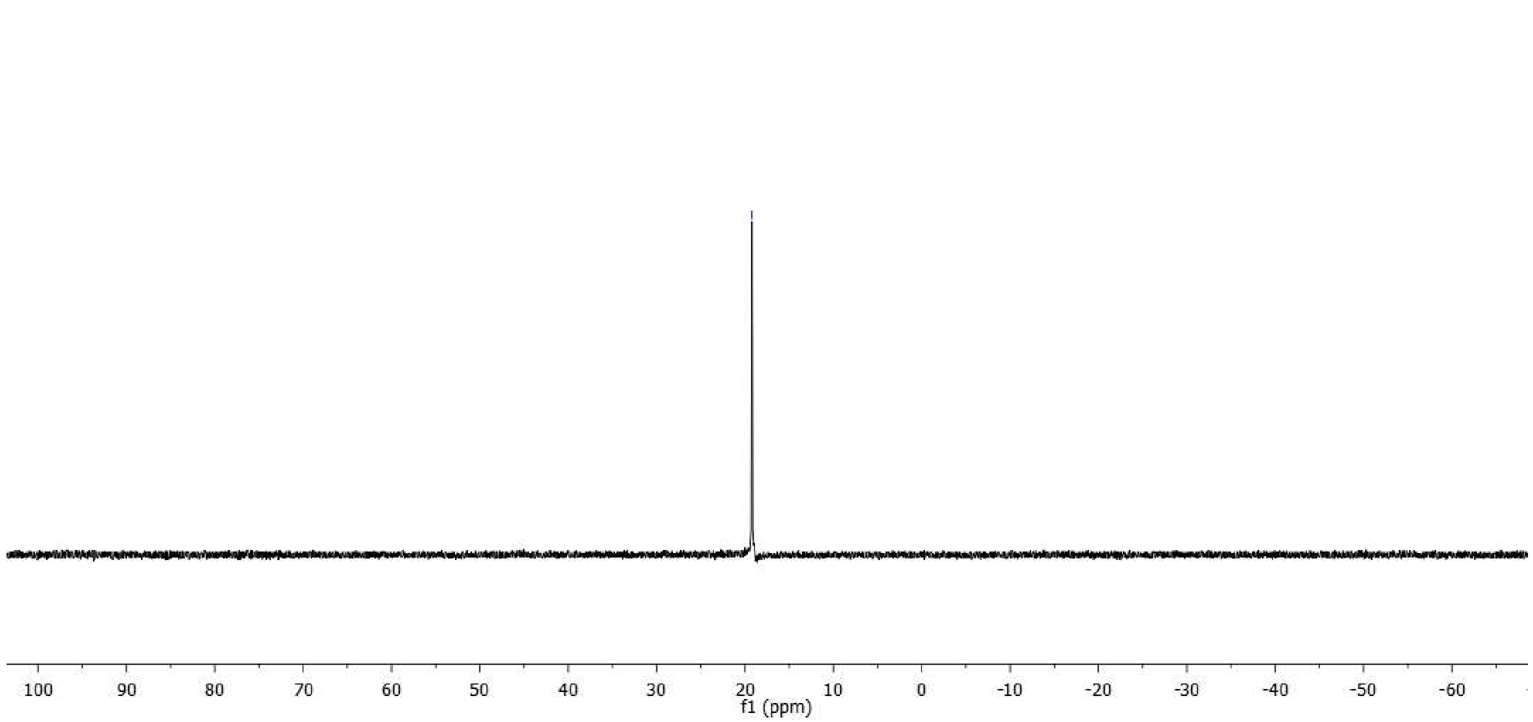

19.21
100
90
80
70
60
50
40
30
20
10
0
-10
-20
-30
-40
-50
-60
f1 (ppm)

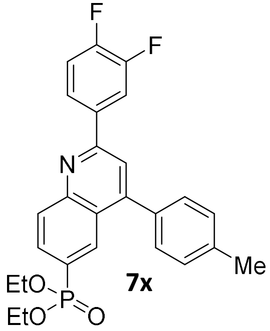

F
F
N
Me
EtO
EtO
P
O

7x

^{19}F-RMN

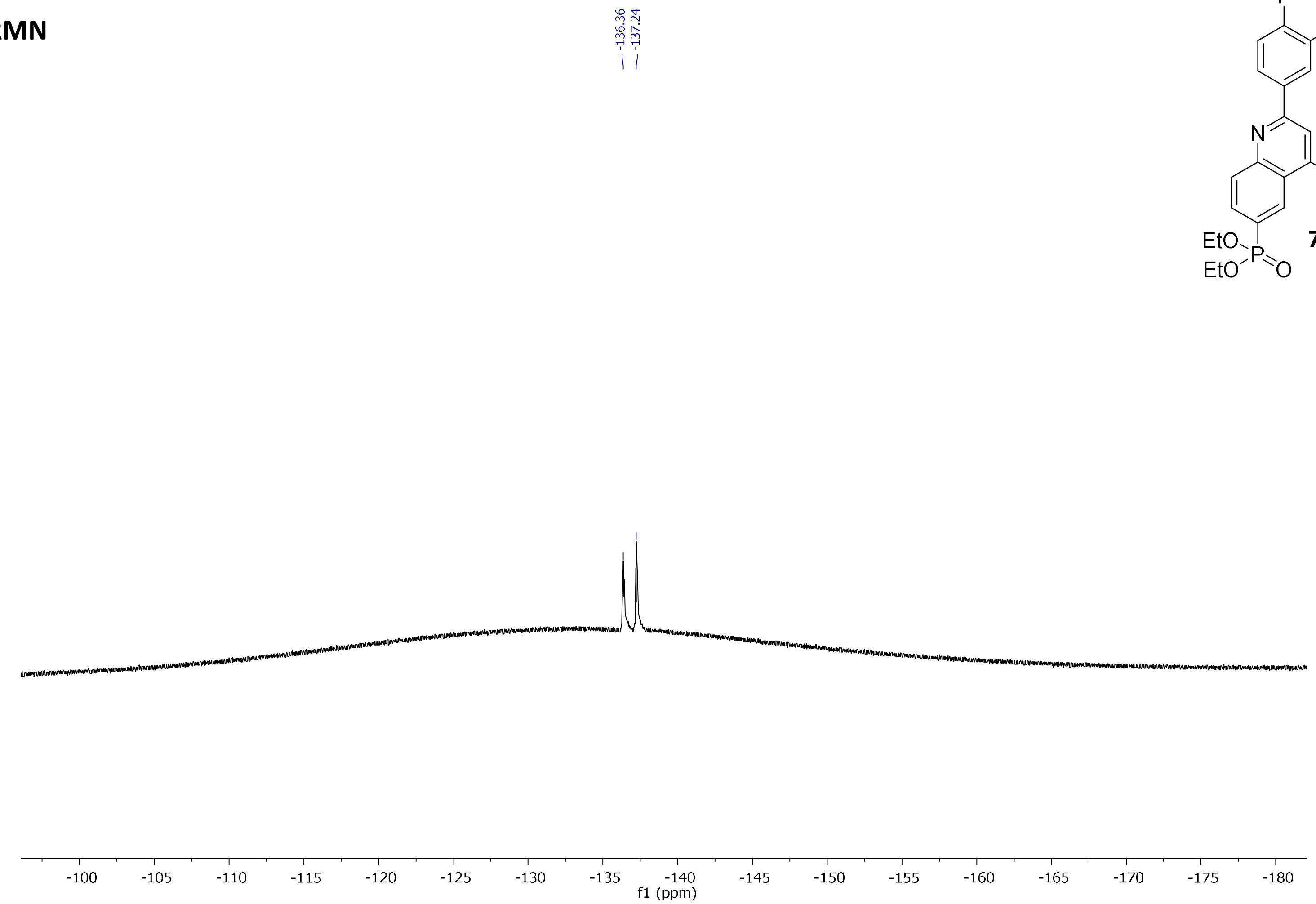

-136.36
-137.24
-100
-105
-110
-115
-120
-125
-130
-135
-140
-145
-150
-155
-160
-165
-170
-175
-180
f1 (ppm)

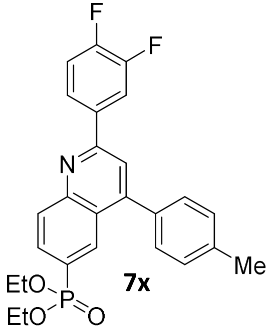

F
F
N
Me
EtO
EtO
P
O

7x

Table S1. Human TOP1B inhibitory activity of compounds **7**.

Entry	Comp.	PO(OR)$_2$	R^1	R^2	% Inhibition[a] 15 seconds	1 minute	3 minutes
1		CPT			++	++	-
2	**7a**	8-(PO(OEt)$_2$)	C_6H_5	H	+	+	+
3	**7b**	8-(PO(OEt)$_2$)	4-F-C_6H_4	H	++	++	+
4	**7c**	8-(PO(OEt)$_2$)	4-OMe-C_6H_4	H	-	-	-
5	**7d**	8-(PO(OEt)$_2$)	2-OMe-C_6H_4	H	+	+	-
6	**7e**	8-(PO(OEt)$_2$)	2-naphthyl	H	-	-	-
7	**7f**	8-(PO(OEt)$_2$)	1-naphthyl	H	+	+	-
8	**7g**	8-(PO(OEt)$_2$)	C_6H_5	F	+	+	-
9	**7h**	8-(PO(OEt)$_2$)	4-F-C_6H_4	F	+	+	-
10	**7i**	8-(PO(OEt)$_2$)	3,4-F_2-C_6H_3	F	+	+	+
11	**7j**	8-(PO(OEt)$_2$)	4-F-C_6H_4	Me	++	++	-
12	**7k**	8-(PO(OEt)$_2$)	3,4-F_2-C_6H_3	Me	++	++	+
13	**7l**	8-(PO(O*i*Pr)$_2$)	4-F-C_6H_4	H	+	-	-
14	**7m**	8-(PO(O*i*Pr)$_2$)	3,4-F_2-C_6H_3	H	+	+	-
15	**7n**	8-(PO(O*i*Pr)$_2$)	4-F-C_6H_4	F	++	++	+
16	**7o**	8-(PO(O*i*Pr)$_2$)	3,4-F_2-C_6H_3	F	+	+	+
17	**7p**	8-(PO(O*i*Pr)$_2$)	4-F-C_6H_4	Me	+	+	-
18	**7q**	8-(PO(O*i*Pr)$_2$)	3,4-F_2-C_6H_3	Me	+	+	-
19	**7r**	6-(PO(OEt)$_2$)	4-F-C_6H_4	H	-	-	-
20	**7s**	6-(PO(OEt)$_2$)	3,4-F_2-C_6H_3	H	-	-	-
21	**7t**	6-(PO(OEt)$_2$)	4-F-C_6H_4	F	+	+	-
22	**7u**	6-(PO(OEt)$_2$)	3,4-F_2-C_6H_3	F	+	+	+
23	**7v**	6-(PO(OEt)$_2$)	4-F-C_6H_4	Me	+	+	+
24	**7x**	6-(PO(OEt)$_2$)	3,4-F_2-C_6H_3	Me	-	-	-

[a] The activity of the compounds to inhibit TopI relaxation was expressed semiquantitatively as follows: Θ, no activity; + little activity; ++ similar to camptothecin, when activity.

Compound 7a

Column: chiralpak IC 0.4cm x 1 cm, DAIC 83311
Mobile phase: Heptane-Ethanol 90/10
Detector Wavelength: 280nm

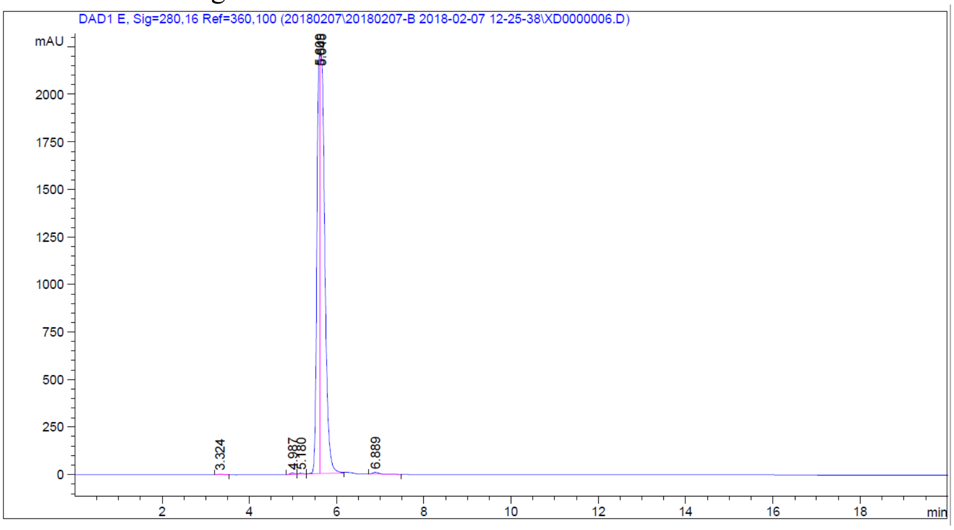

Signal 1: DAD1 E, Sig=280,16 Ref=360,100

Peak #	RetTime [min]	Type	Width [min]	Area [mAU*s]	Height [mAU]	Area %
1	3.324	BB	0.1554	21.27357	1.97944	0.0776
2	4.987	BV	0.1126	54.17192	7.47449	0.1976
3	5.180	VV	0.1152	38.09583	5.09647	0.1390
4	5.609	VV	0.0779	1.07977e4	2199.80566	39.3899
5	5.645	VB	0.1109	1.64224e4	2204.02148	59.9087
6	6.889	VB	0.1366	78.73672	9.13323	0.2872

Compound 7b

Column: chiralpak IC 0.4cm x 1 cm, DAIC 83311
Mobile phase: Heptane-Ethanol 90/10
Detector Wavelength: 280nm

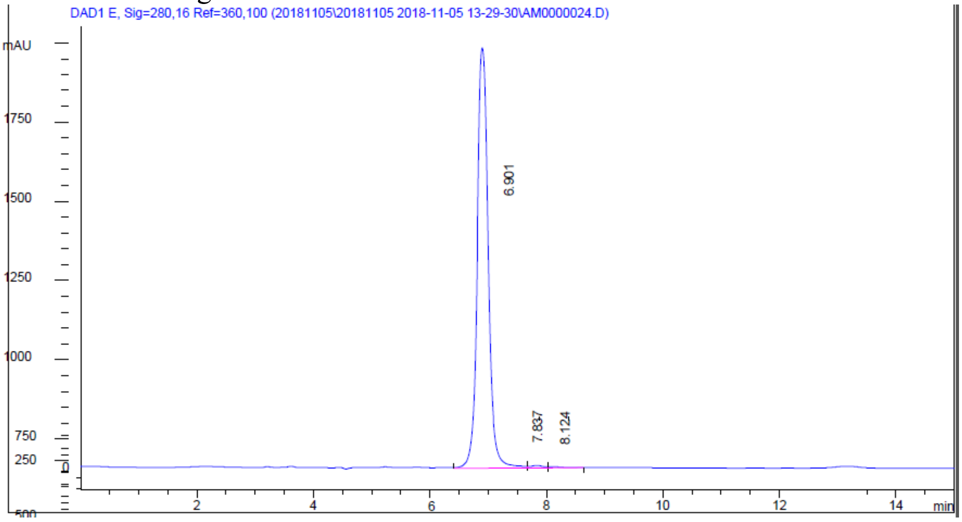

Signal 1: DAD1 E, Sig=280,16 Ref=360,100

Peak #	RetTime [min]	Type	Width [min]	Area [mAU*s]	Height [mAU]	Area %
1	6.901	BV	0.2010	2.52473e4	1924.86206	98.9606
2	7.837	VV	0.2091	171.33589	11.82178	0.6716
3	8.124	VB	0.2363	93.83268	5.63451	0.3678

Totals : 2.55124e4 1942.31835

Compound 7c

Column: chiralpak IC 0.4cm x 1 cm, DAIC 83311
Mobile phase: Heptane-Ethanol 90/10
Detector Wavelength: 280nm

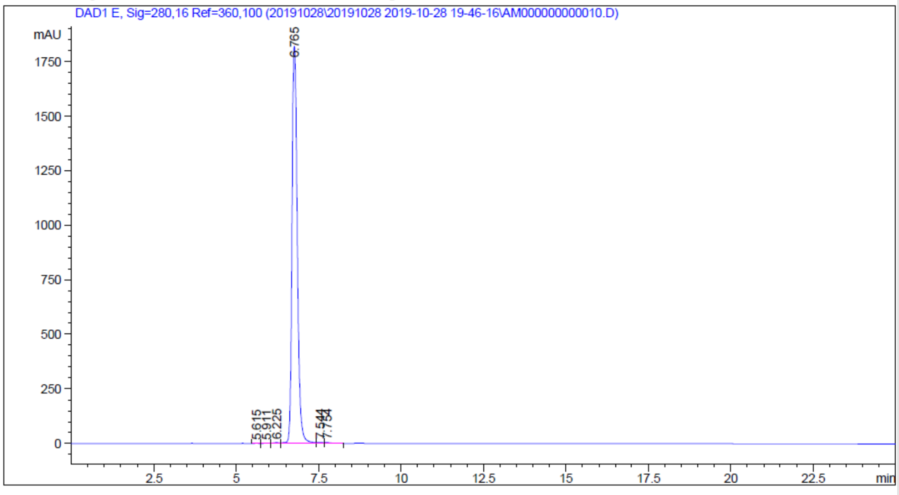

Signal 1: DAD1 E, Sig=280,16 Ref=360,100

Peak #	RetTime [min]	Type	Width [min]	Area [mAU*s]	Height [mAU]	Area %
1	5.615	BV	0.1071	9.86208	1.38510	0.0481
2	5.911	VV	0.1388	14.22149	1.55299	0.0693
3	6.225	VV	0.1800	51.03660	4.50994	0.2488
4	6.765	VV	0.1744	2.03161e4	1817.98633	99.0224
5	7.544	VV	0.1711	62.94007	5.28034	0.3068
6	7.754	VB	0.2072	62.50720	4.30991	0.3047

Totals : 2.05166e4 1835.02461

Compound 7d

Column: chiralpak IC 0.4cm x 1 cm, DAIC 83311
Mobile phase: Heptane-Ethanol 90/10
Detector Wavelength: 254nm

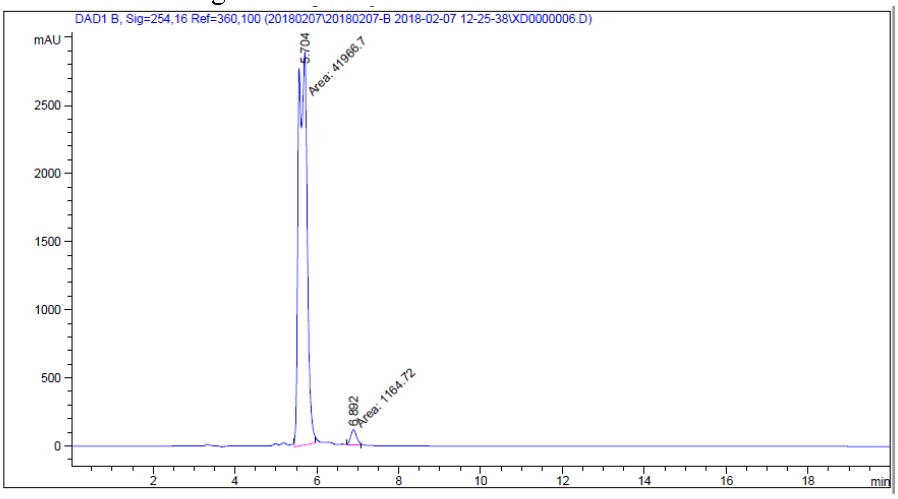

Signal 1: DAD1 B, Sig=254,16 Ref=360,100

Peak #	RetTime [min]	Type	Width [min]	Area [mAU*s]	Height [mAU]	Area %
1	5.704	MM	0.2426	4.19667e4	2882.74072	97.2996
2	6.892	MM	0.1720	1164.71875	112.84191	2.7004

Totals : 4.31314e4 2995.58263

Compound 7e

Column: chiralpak IC 0.4cm x 1 cm, DAIC 83311
Mobile phase: Heptane-Ethanol 90/10
Detector Wavelength: 280nm

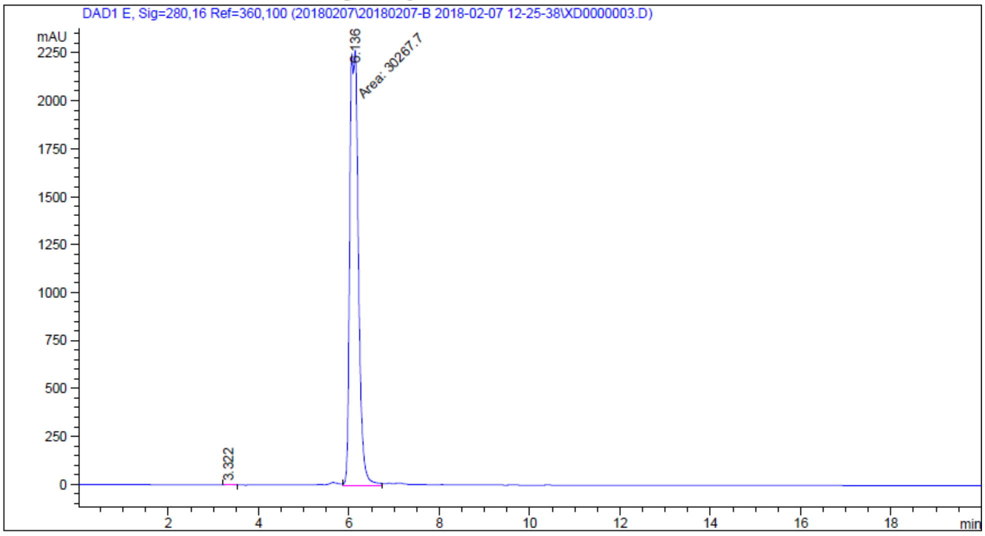

Signal 1: DAD1 E, Sig=280,16 Ref=360,100

Peak #	RetTime [min]	Type	Width [min]	Area [mAU*s]	Height [mAU]	Area %
1	3.322	BB	0.1607	28.21144	2.48114	0.0931
2	6.136	MM	0.2223	3.02677e4	2269.50781	99.9069
Totals :				3.02959e4	2271.98895	

Compound 7f

Column: chiralpak IC 0.4cm x 1 cm, DAIC 83311
Mobile phase: Heptane-Ethanol 90/10
Detector Wavelength: 280nm

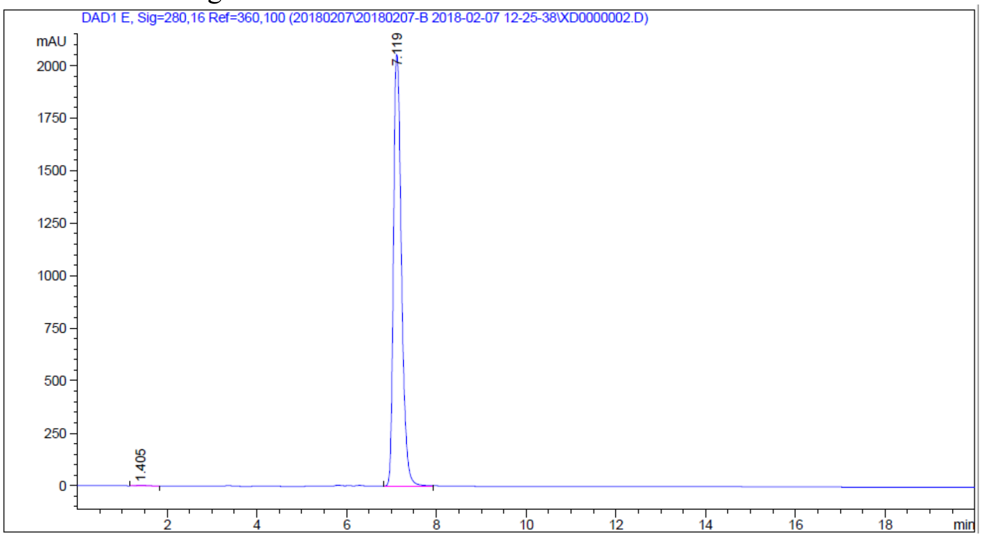

Signal 1: DAD1 E, Sig=280,16 Ref=360,100

Peak #	RetTime [min]	Type	Width [min]	Area [mAU*s]	Height [mAU]	Area %
1	1.405	BB	0.2174	58.99203	3.79465	0.2306
2	7.119	BB	0.1909	2.55275e4	2054.90747	99.7694
Totals :				2.55865e4	2058.70212	

Compound 7g

Column: chiralpak IC 0.4cm x 1 cm, DAIC 83311
Mobile phase: Heptane-Ethanol 90/10
Detector Wavelength: 230nm

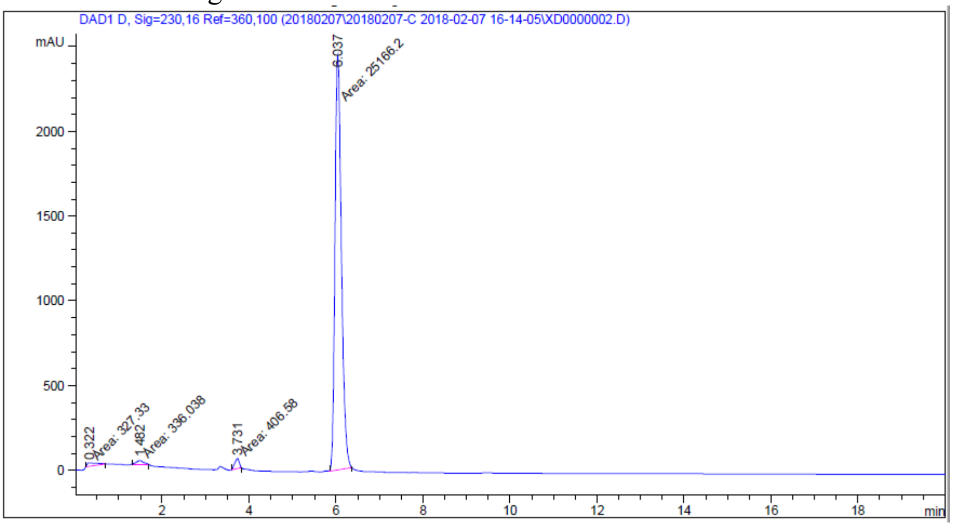

Signal 1: DAD1 D, Sig=230,16 Ref=360,100

Peak #	RetTime [min]	Type	Width [min]	Area [mAU*s]	Height [mAU]	Area %
1	0.322	MM	0.2512	327.33038	21.71726	1.2476
2	1.482	MM	0.2162	336.03784	25.90994	1.2808
3	3.731	MM	0.1149	406.57983	58.98366	1.5497
4	6.037	MM	0.1714	2.51662e4	2447.33716	95.9219

Totals : 2.62361e4 2553.94802

Compound 7h

Column: chiralpak IC 0.4cm x 1 cm, DAIC 83311
Mobile phase: Heptane-Ethanol 90/10
Detector Wavelength: 280nm

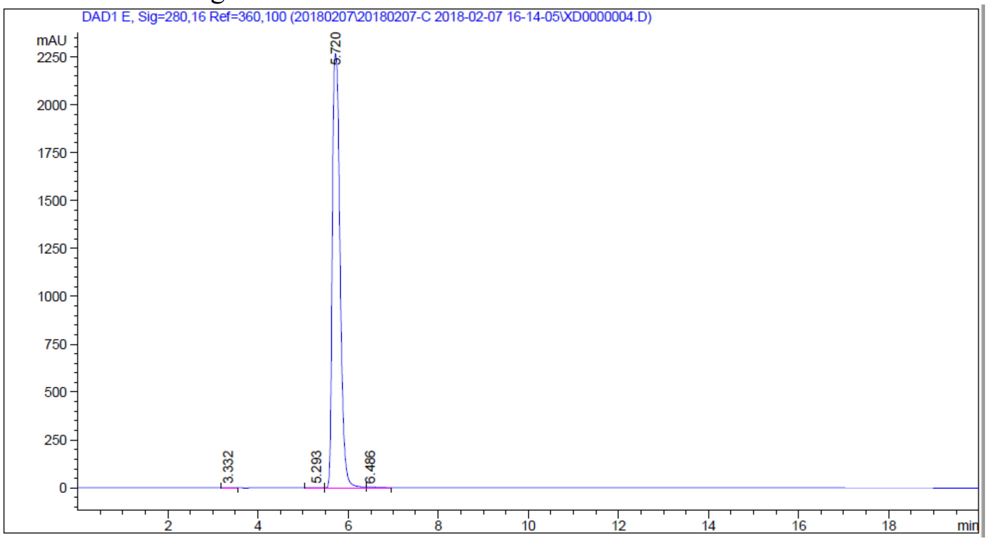

Signal 1: DAD1 E, Sig=280,16 Ref=360,100

Peak #	RetTime [min]	Type	Width [min]	Area [mAU*s]	Height [mAU]	Area %
1	3.332	BB	0.1778	17.05152	1.38368	0.0632
2	5.293	BV	0.1759	17.46941	1.39706	0.0647
3	5.720	VB	0.1884	2.68875e4	2265.86060	99.5986
4	6.486	BB	0.2575	73.83075	4.11165	0.2735

Totals : 2.69958e4 2272.75299

Compound 7i

Column: chiralpak IC 0.4cm x 1 cm, DAIC 83311
Mobile phase: Heptane-Ethanol 90/10
Detector Wavelength: 280nm

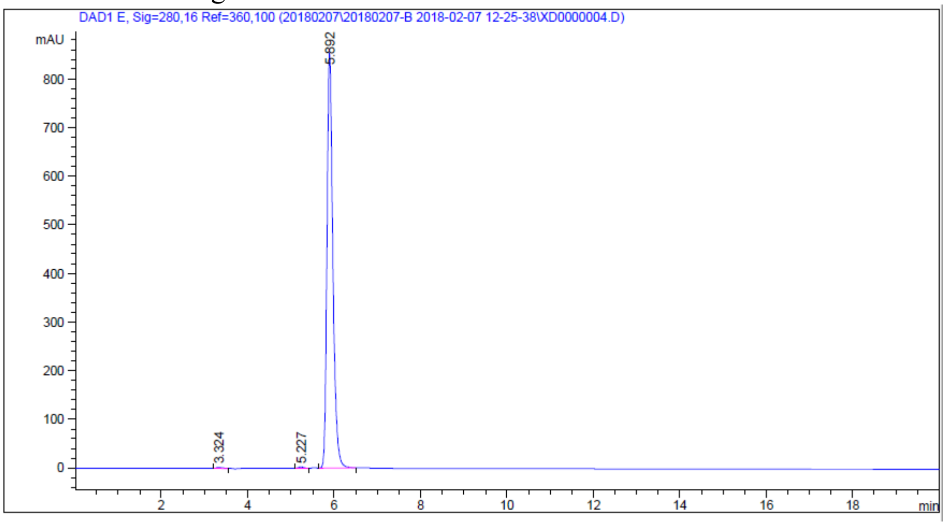

Signal 1: DAD1 E, Sig=280,16 Ref=360,100

Peak #	RetTime [min]	Type	Width [min]	Area [mAU*s]	Height [mAU]	Area %
1	3.324	BB	0.1547	20.58506	1.89519	0.2538
2	5.227	BV	0.1183	22.61293	2.92268	0.2788
3	5.892	VB	0.1443	8067.45117	853.68011	99.4674

Totals : 8110.64916 858.49799

Compound 7j

Column: chiralpak IC 0.4cm x 1 cm, DAIC 83311
Mobile phase: Heptane-Ethanol 90/10
Detector Wavelength: 254nm

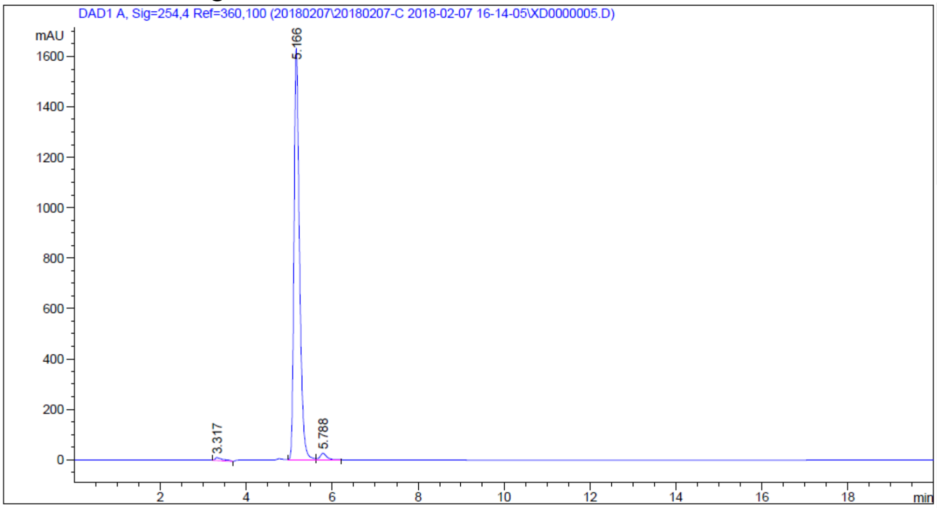

Signal 1: DAD1 A, Sig=254,4 Ref=360,100

Peak #	RetTime [min]	Type	Width [min]	Area [mAU*s]	Height [mAU]	Area %
1	3.317	BV	0.1833	149.74402	10.96568	1.0040
2	5.166	VV	0.1334	1.44782e4	1633.24390	97.0727
3	5.788	VB	0.1604	286.85782	26.07115	1.9233

Totals : 1.49148e4 1670.28074

Compound 7k

Column: chiralpak IC 0.4cm x 1 cm, DAIC 83311
Mobile phase: Heptane-Ethanol 90/10
Detector Wavelength: 280nm

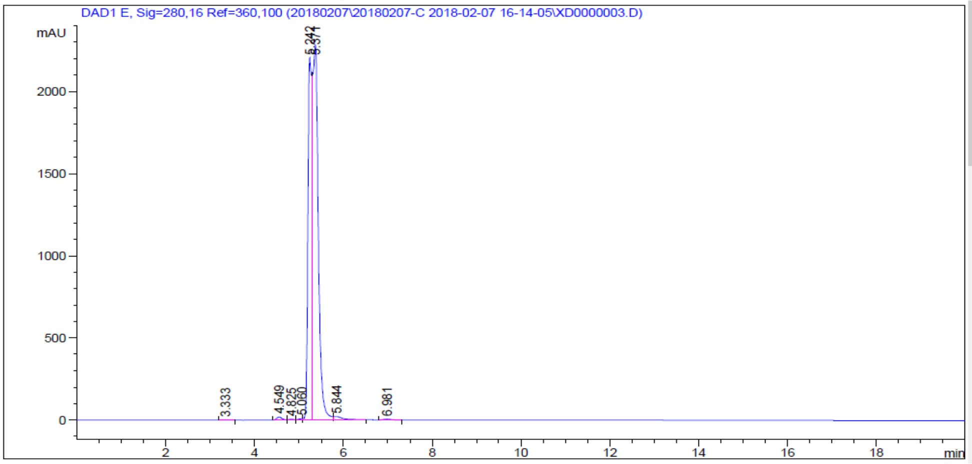

Signal 1: DAD1 E, Sig=280,16 Ref=360,100

Peak #	RetTime [min]	Type	Width [min]	Area [mAU*s]	Height [mAU]	Area %
1	3.333	BB	0.1634	15.12790	1.32290	0.0444
2	4.549	BV	0.1216	149.74399	18.66644	0.4395
3	4.825	VV	0.1173	41.81712	5.34407	0.1227
4	5.060	VV	0.1000	52.74845	7.88731	0.1548
5	5.242	VV	0.0807	1.22464e4	2209.17383	35.9405
6	5.371	VV	0.1262	2.12169e4	2286.79272	62.2671
7	5.844	VB	0.2075	298.00760	19.81295	0.8746
8	6.981	BB	0.1678	53.28969	4.86428	0.1564
Totals :				3.40740e4	4553.86451	

Compound 7l

Column: chiralpak IC 0.4cm x 1 cm, DAIC 83311
Mobile phase: Heptane-Ethanol 90/10
Detector Wavelength: 280nm

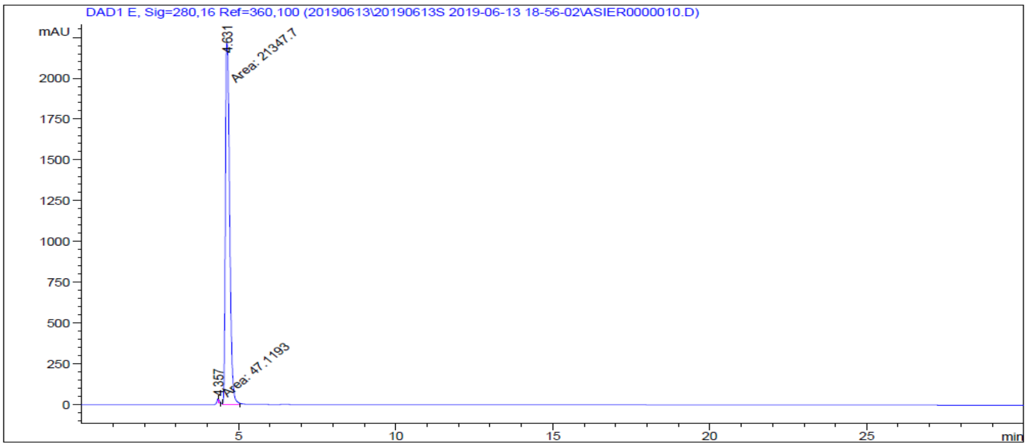

Signal 1: DAD1 E, Sig=280,16 Ref=360,100

Peak #	RetTime [min]	Type	Width [min]	Area [mAU*s]	Height [mAU]	Area %
1	4.357	MM	0.0345	47.11928	22.76258	0.2202
2	4.631	MM	0.1604	2.13477e4	2218.78882	99.7798
Totals :				2.13948e4	2241.55139	

Compound 7m

Column: chiralpak IC 0.4cm x 1 cm, DAIC 83311
Mobile phase: Heptane-Ethanol 90/10
Detector Wavelength: 280nm

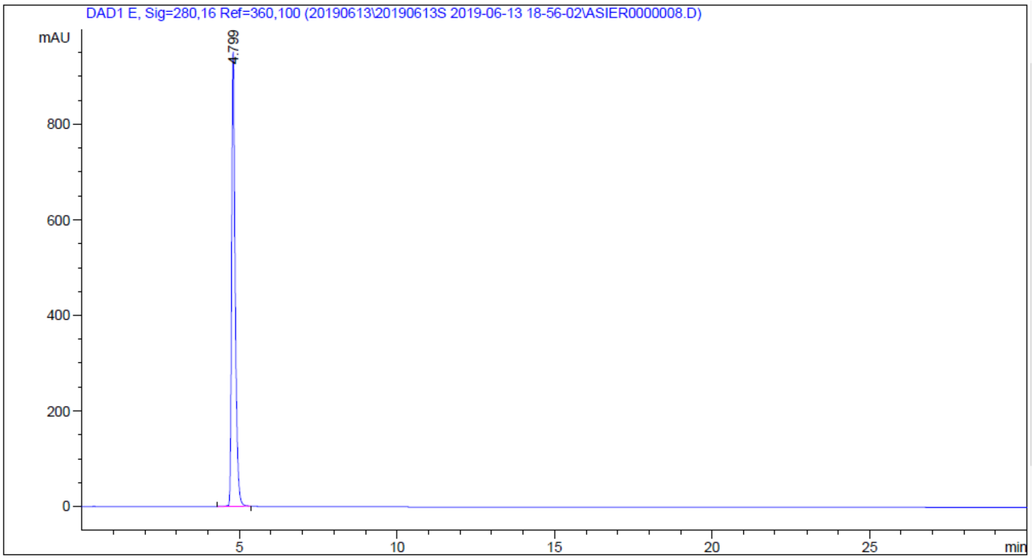

Signal 1: DAD1 E, Sig=280,16 Ref=360,100

Peak #	RetTime [min]	Type	Width [min]	Area [mAU*s]	Height [mAU]	Area %
1	4.799	BB	0.1211	7596.80078	952.29565	100.0000
Totals :				7596.80078	952.29565	

Compound 7n

Column: chiralpak IC 0.4cm x 1 cm, DAIC 83311
Mobile phase: Heptane-Ethanol 90/10
Detector Wavelength: 280nm

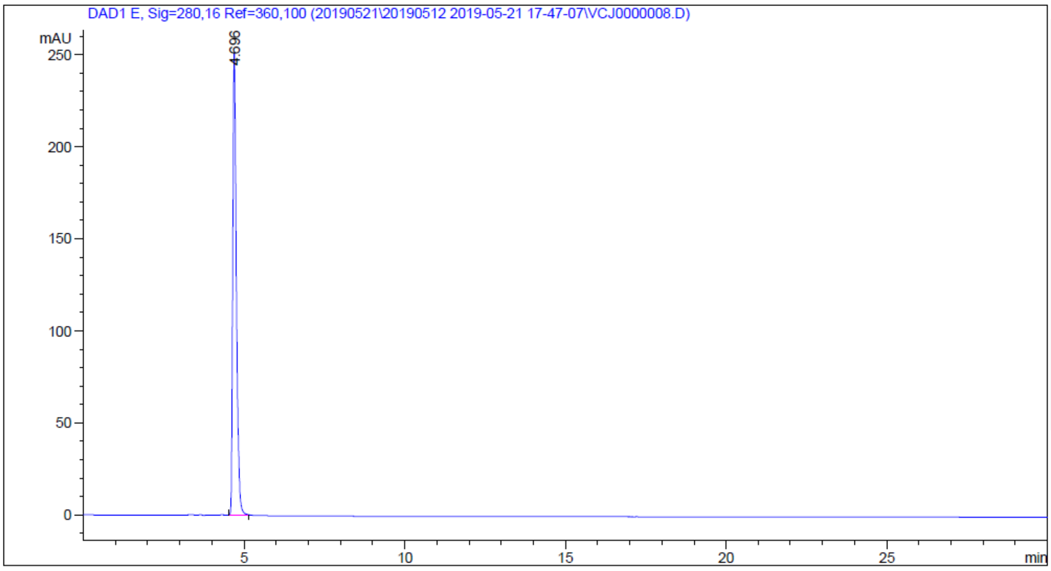

Signal 1: DAD1 E, Sig=280,16 Ref=360,100

Peak #	RetTime [min]	Type	Width [min]	Area [mAU*s]	Height [mAU]	Area %
1	4.696	BB	0.1168	1958.36707	251.73123	100.0000
Totals :				1958.36707	251.73123	

Compound 7o

Column: chiralpak IC 0.4cm x 1 cm, DAIC 83311
Mobile phase: Heptane-Ethanol 90/10
Detector Wavelength: 280nm

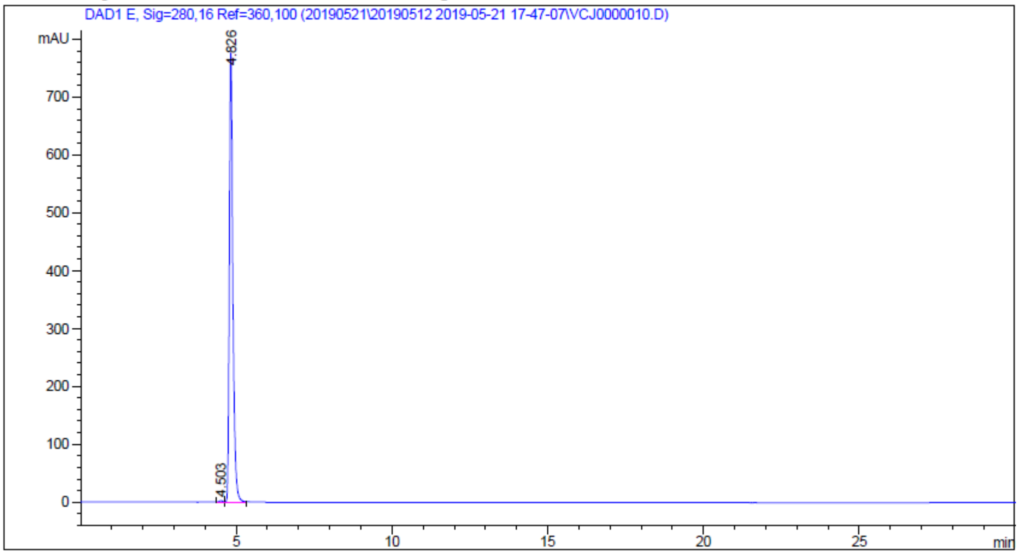

Signal 1: DAD1 E, Sig=280,16 Ref=360,100

Peak #	RetTime [min]	Type	Width [min]	Area [mAU*s]	Height [mAU]	Area %
1	4.503	BV	0.1072	17.63663	2.47254	0.2835
2	4.826	VB	0.1192	6202.64600	776.58984	99.7165
Totals :				6220.28263	779.06239	

Compound 7p

Column: chiralpak IC 0.4cm x 1 cm, DAIC 83311
Mobile phase: Heptane-Ethanol 90/10
Detector Wavelength: 280nm

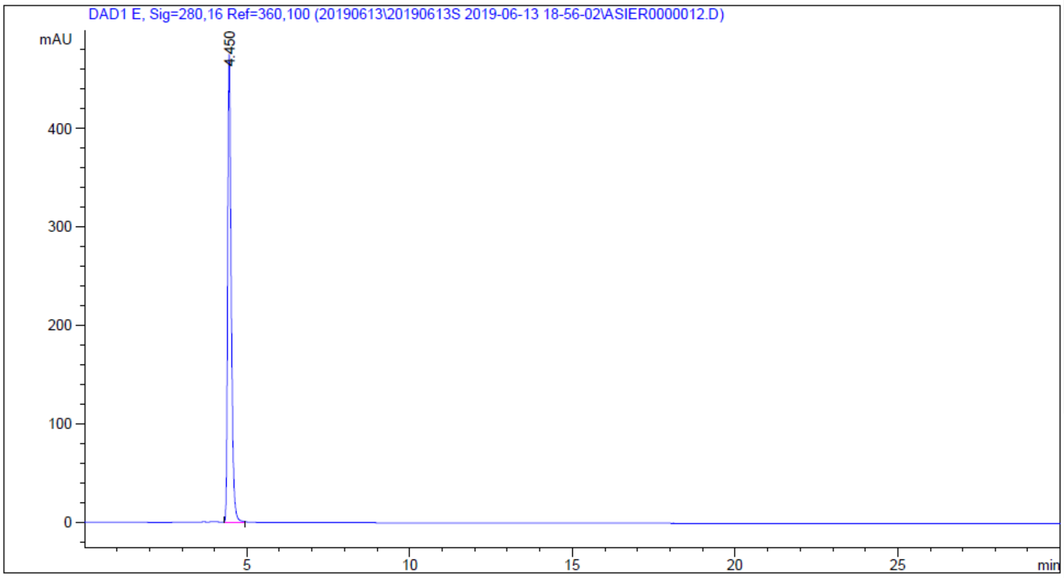

Signal 1: DAD1 E, Sig=280,16 Ref=360,100

Peak #	RetTime [min]	Type	Width [min]	Area [mAU*s]	Height [mAU]	Area %
1	4.450	BB	0.1111	3563.30615	477.03915	100.0000
Totals :				3563.30615	477.03915	

Compound 7q

Column: chiralpak IC 0.4cm x 1 cm, DAIC 83311
Mobile phase: Heptane-Ethanol 90/10
Detector Wavelength: 280nm

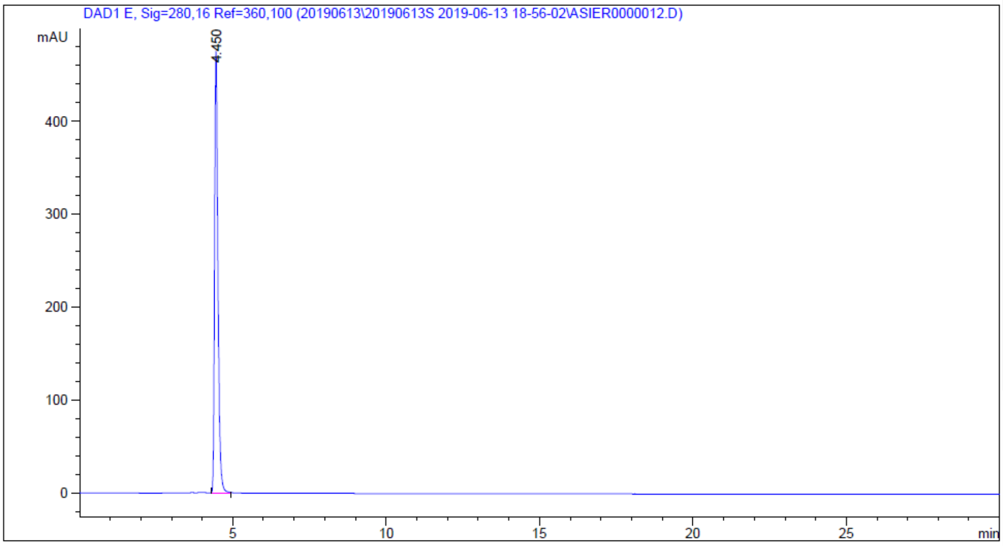

Signal 1: DAD1 E, Sig=280,16 Ref=360,100

Peak #	RetTime [min]	Type	Width [min]	Area [mAU*s]	Height [mAU]	Area %
1	4.450	BB	0.1111	3563.30615	477.03915	100.0000
Totals :				3563.30615	477.03915	

Compound 7r

Column: chiralpak IC 0.4cm x 1 cm, DAIC 83311
Mobile phase: Heptane-Ethanol 90/10
Detector Wavelength: 280nm

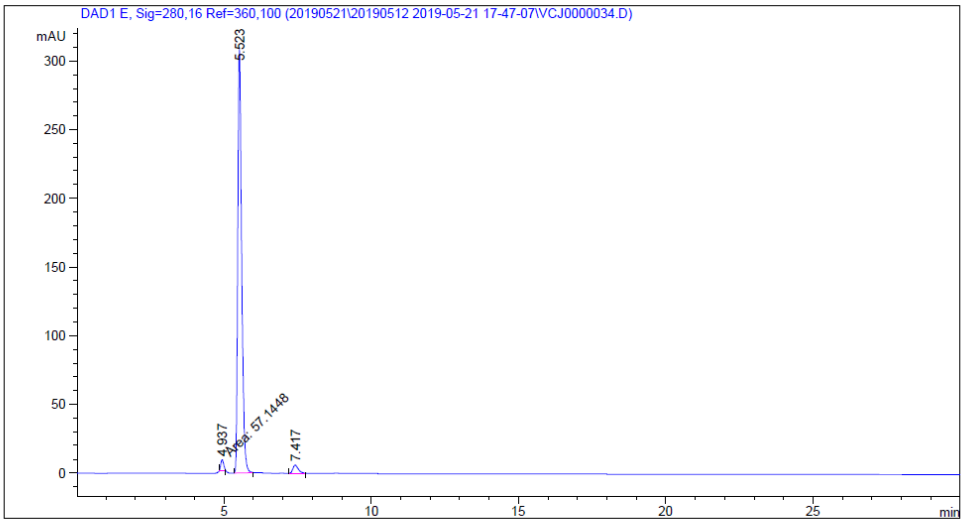

Signal 1: DAD1 E, Sig=280,16 Ref=360,100

Peak #	RetTime [min]	Type	Width [min]	Area [mAU*s]	Height [mAU]	Area %
1	4.937	MM	0.1139	57.14476	8.36224	1.9581
2	5.523	BB	0.1353	2784.66089	308.61548	95.4173
3	7.417	BB	0.1927	76.59584	6.01015	2.6246
Totals :				2918.40149	322.98788	

Compound 7s

Column: chiralpak IC 0.4cm x 1 cm, DAIC 83311
Mobile phase: Heptane-Ethanol 90/10
Detector Wavelength: 280nm

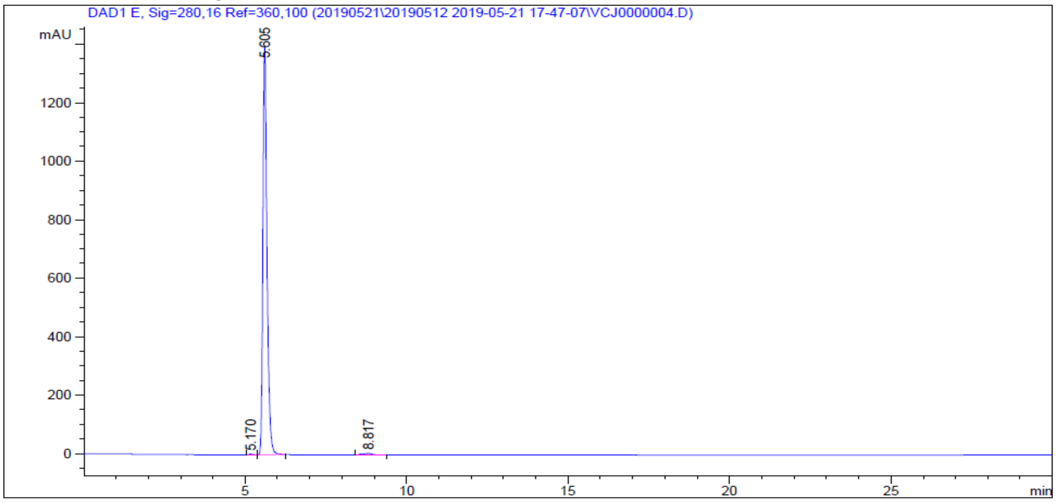

Signal 1: DAD1 E, Sig=280,16 Ref=360,100

Peak #	RetTime [min]	Type	Width [min]	Area [mAU*s]	Height [mAU]	Area %
1	5.170	BV	0.1328	19.47662	2.16818	0.1536
2	5.605	VB	0.1350	1.25486e4	1394.89685	98.9406
3	8.817	BB	0.2914	114.88801	5.41750	0.9058

Totals : 1.26830e4 1402.48252

Compound 7t

Column: chiralpak IC 0.4cm x 1 cm, DAIC 83311
Mobile phase: Heptane-Ethanol 90/10
Detector Wavelength: 330nm

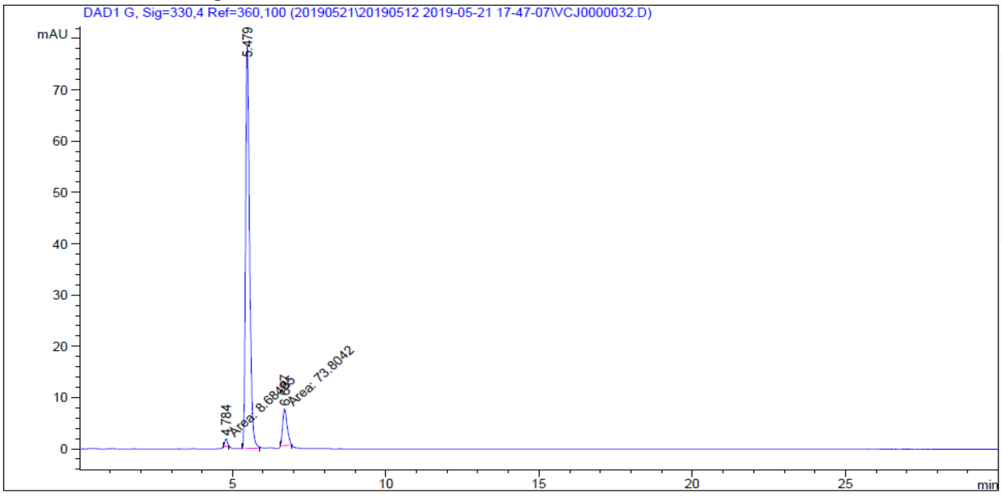

Signal 1: DAD1 G, Sig=330,4 Ref=360,100

Peak #	RetTime [min]	Type	Width [min]	Area [mAU*s]	Height [mAU]	Area %
1	4.784	MM	0.1007	8.68485	1.43806	1.1057
2	5.479	BB	0.1326	702.98444	78.42625	89.4982
3	6.697	MM	0.1717	73.80424	7.16324	9.3961

Totals : 785.47353 87.02755

Compound 7u

Column: chiralpak IC 0.4cm x 1 cm, DAIC 83311
Mobile phase: Heptane-Ethanol 90/10
Detector Wavelength: 273nm

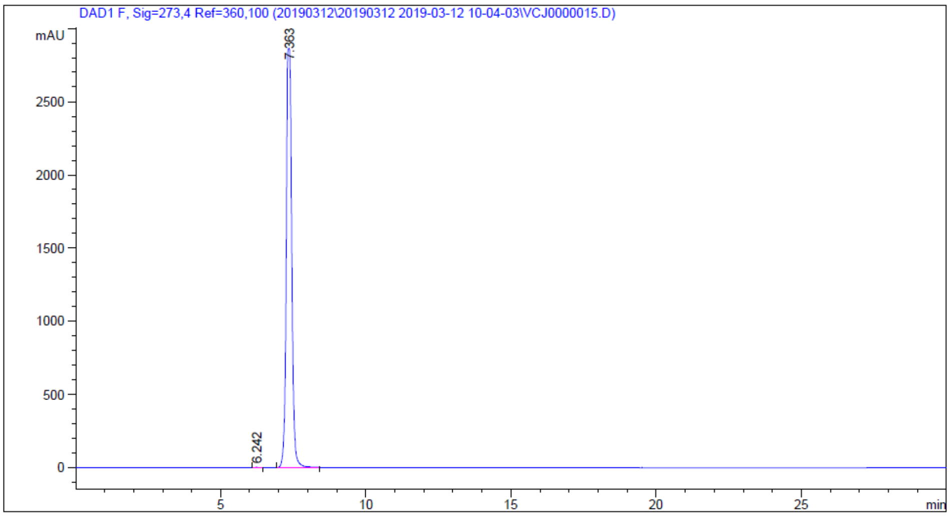

Peak #	RetTime [min]	Type	Width [min]	Area [mAU*s]	Height [mAU]	Area %
1	6.242	BB	0.1218	14.69139	1.86748	0.0390
2	7.363	BB	0.1776	3.76952e4	2862.90088	99.9610

Totals : 3.77099e4 2864.76836

Compound 7v

Column: chiralpak IC 0.4cm x 1 cm, DAIC 83311
Mobile phase: Heptane-Ethanol 90/10
Detector Wavelength: 280nm

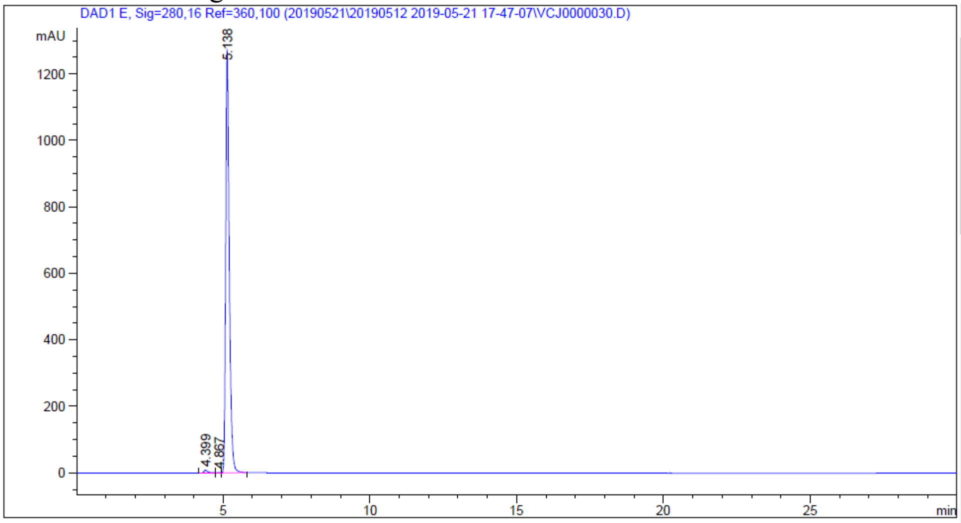

Signal 1: DAD1 E, Sig=280,16 Ref=360,100

Peak #	RetTime [min]	Type	Width [min]	Area [mAU*s]	Height [mAU]	Area %
1	4.399	BB	0.1375	77.25000	8.08078	0.6951
2	4.867	BV	0.1017	8.32403	1.25018	0.0749
3	5.138	VB	0.1310	1.10283e4	1274.22290	99.2300

Totals : 1.11139e4 1283.55386

Compound 7x

Column: chiralpak IC 0.4cm x 1 cm, DAIC 83311
Mobile phase: Heptane-Ethanol 90/10
Detector Wavelength: 280nm

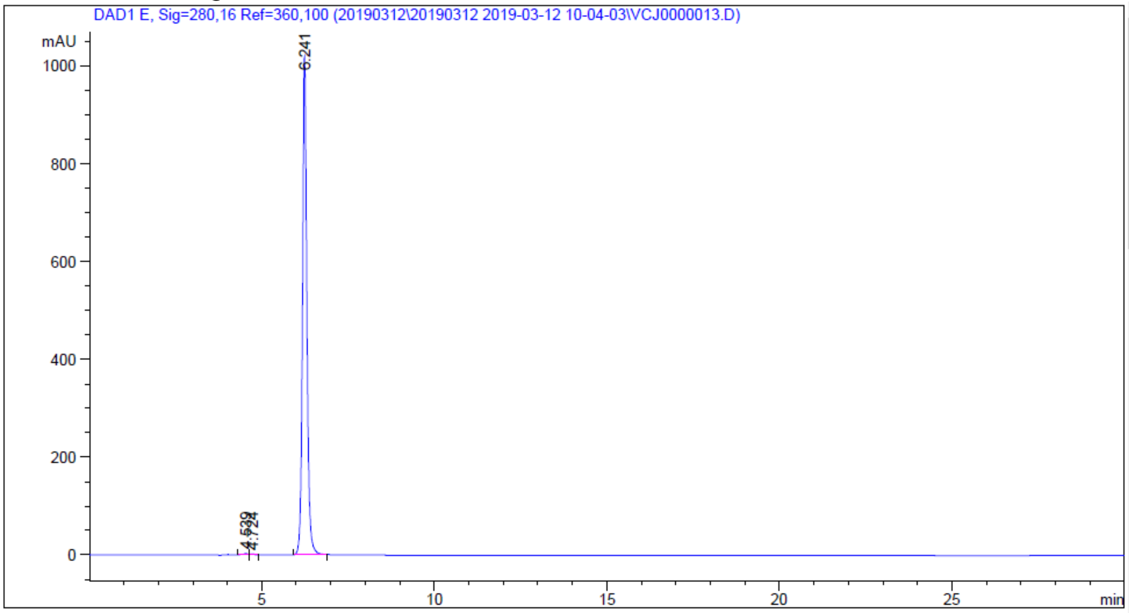

Signal 1: DAD1 E, Sig=280,16 Ref=360,100

Peak #	RetTime [min]	Type	Width [min]	Area [mAU*s]	Height [mAU]	Area %
1	4.539	BV	0.1375	29.96637	3.02759	0.3242
2	4.724	VB	0.1088	19.06075	2.62186	0.2062
3	6.241	BB	0.1353	9193.07715	1018.56769	99.4695

Totals : 9242.10427 1024.21714

Relaxation assay (LTopIB)

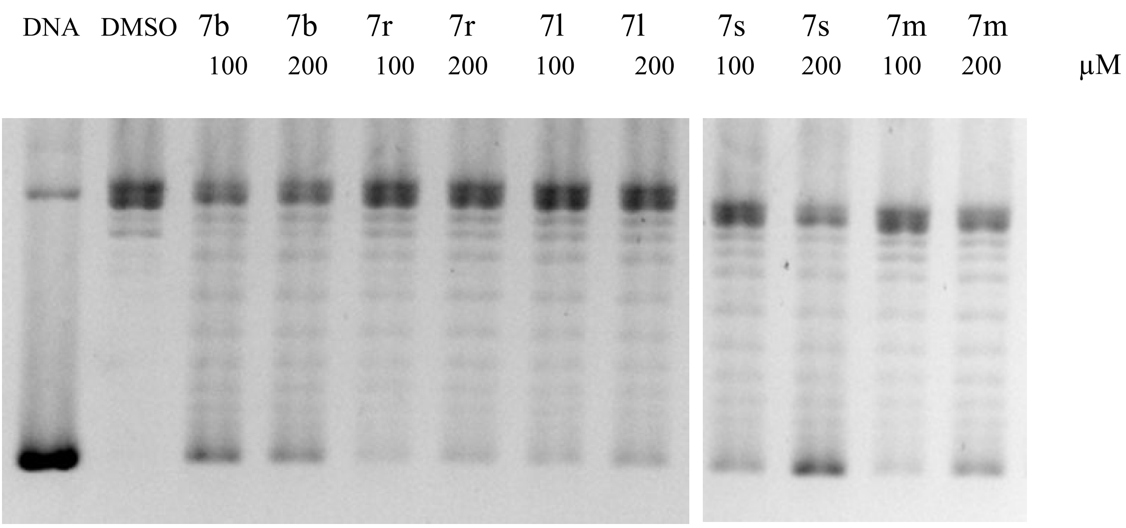

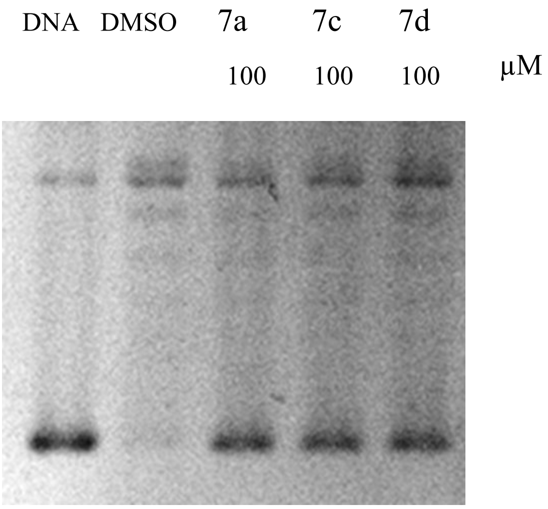

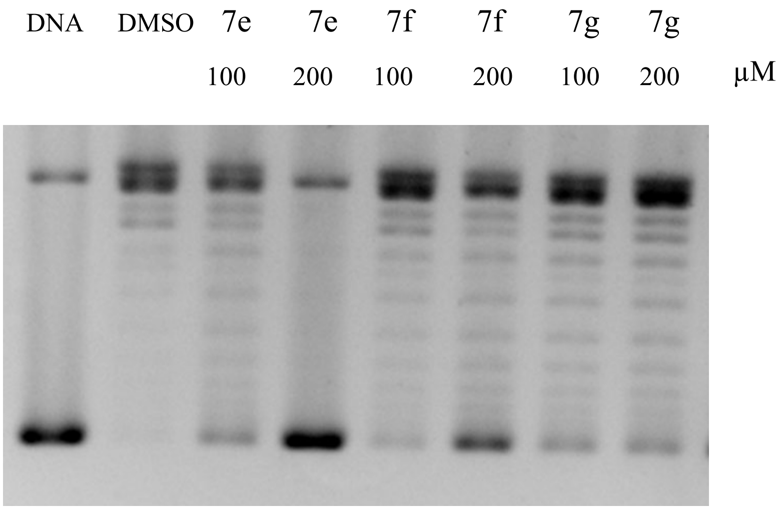

Relaxation assay (LTopIB)

DNA DMSO 7h 7h 7t 7t 7n 7n 7i 7i 7u 7u 7o 7o

100 200 100 200 100 200 100 200 100 200 100 200 μM

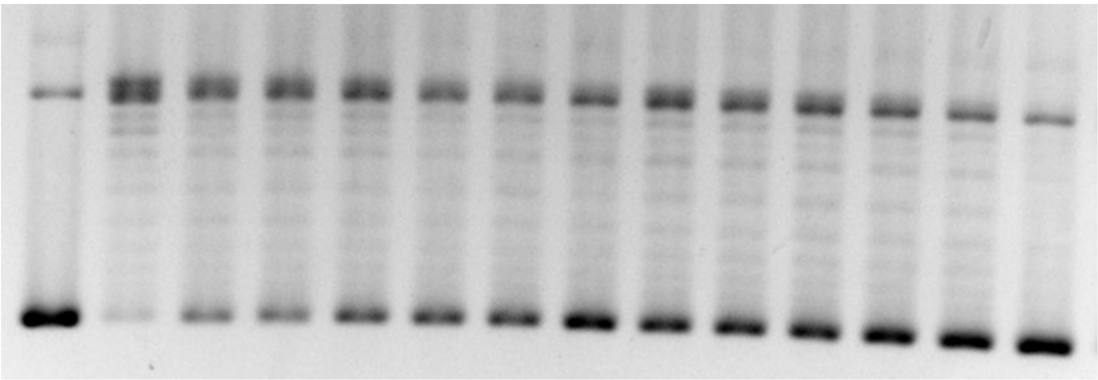

DNA DMSO 7j 7j 7v 7v 7p 7p 7k 7k 7x 7x 7q 7q

100 200 100 200 100 200 100 200 100 200 100 200 μM

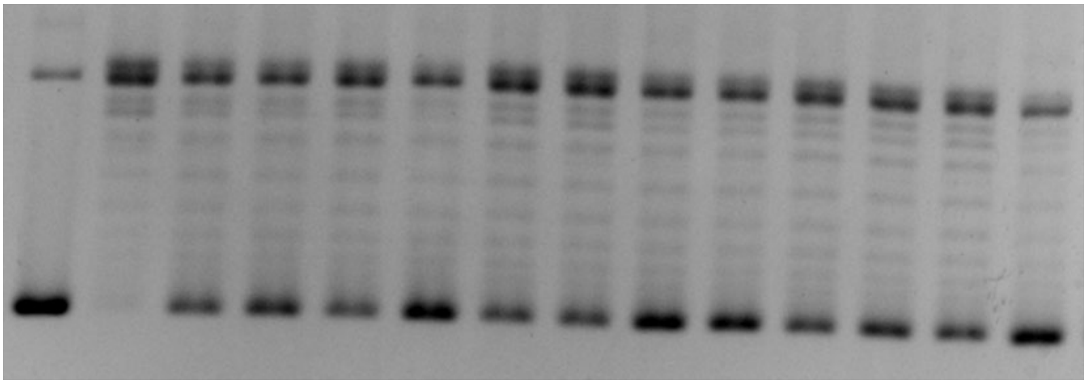

Supplement: Supplementary file 1 [file pharmaceuticals-14-00784-s001.zip › pharmaceuticals-1318130-supplementary.pdf]
